# Radical Scavenging Potential of the Phenothiazine Scaffold: A Computational Analysis

**DOI:** 10.1002/cmdc.202100546

**Published:** 2021-10-15

**Authors:** Marco Dalla Tiezza, Trevor A. Hamlin, F. Matthias Bickelhaupt, Laura Orian

**Affiliations:** ^1^ Dipartimento di Scienze Chimiche Università degli Studi di Padova Via Marzolo 1 35131 Padova Italy; ^2^ Department of Theoretical Chemistry Amsterdam Institute of Molecular and Life Sciences (AIMMS) Amsterdam Center for Multiscale Modeling (ACMM) Vrije Universiteit Amsterdam De Boelelaan 1083 1081 HV Amsterdam The Netherlands; ^3^ Institute for Molecules and Materials (IMM) Radboud University Heyendaalseweg 135 6525 AJ Nijmegen The Netherlands

**Keywords:** Antioxidants, Density functional calculations, Radical scavenging, Sulfur, Selenium

## Abstract

The reactivity of phenothiazine (**PS**), phenoselenazine (**PSE**), and phenotellurazine (**PTE**) with different reactive oxygen species (ROS) has been studied using density functional theory (DFT) in combination with the QM‐ORSA (Quantum Mechanics‐based Test for Overall Free Radical Scavenging Activity) protocol for an accurate kinetic rate calculation. Four radical scavenging mechanisms have been screened, namely hydrogen atom transfer (HAT), radical adduct formation (RAF), single electron transfer (SET), and the direct oxidation of the chalcogen atom. The chosen ROS are HO^.^, HOO^.^, and CH_3_OO^.^. **PS**, **PSE**, and **PTE** exhibit an excellent antioxidant activity in water regardless of the ROS due to their characteristic diffusion‐controlled regime processes. For the HO^.^ radical, the primary active reaction mechanism is, for all antioxidants, RAF. But, for HOO^.^ and CH_3_OO^.^, the dominant mechanism strongly depends on the antioxidant: HAT for **PS** and **PSE**, and SET for **PTE**. The scavenging efficiency decreases dramatically in lipid environment and remains only significant (via RAF) for the most reactive radical (HO^.^). Therefore, **PS**, **PSE**, and **PTE** are excellent antioxidant molecules, especially in aqueous, physiological environments where they are active against a broad spectrum of harmful radicals. There is no advantage or significant difference in the scavenging efficiency when changing the chalcogen since the reactivity mainly derives from the amino hydrogen and the aromatic sites.

## Graphical abstract

**Phenothiazine and its derivatives** are well‐known antipsychotic, antiemetic, and sedative drugs. Like reported for other psychoactive substances, we investigate their effective radical scavenging capability. The scavenging potential of phenothiazine, phenoselenazine, and phenotellurazine against three common harmful ROSs and in two different media are investigated *in silico*. Our results indicate that they indeed possess high antioxidant efficiency combined to their primary drug function.

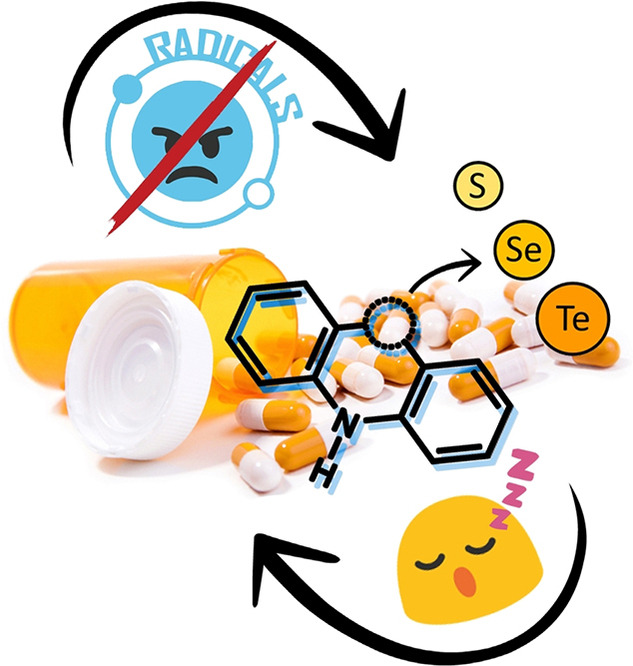

## Introduction

Oxidative stress is a pathological condition due to an unbalanced (too high) concentration of highly oxidant species in the cell, like peroxides and harmful radicals, which can react with phospholipids, proteins, and nucleic acids, impairing their function.[^1^, ^2^, ^3^, ^4^, ^5^, ^6^] The antioxidant defense system cannot efficiently maintain the redox equilibrium inside the cell, so that its components are irreversibly damaged. Oxidative stress is found in numerous diseases of different severity, from inflammatory processes to diabetes, cardiovascular and autoimmune diseases, cancer, and neurodegenerative diseases.[^7^, ^8^, ^9^, ^10^, ^11^] Oxidative stress also accompanies several critical mental disorders, like depression, schizophrenia, and even certain addictions. This is not surprising because the brain is particularly vulnerable to oxidative stress due to its large oxygen consumption. It is not clear whether oxidative stress is a cause or a consequence of the pathological condition and, for this reason, no treatment can be exclusively tailored to fight oxidative stress. Nevertheless, there is clinical evidence that a regular intake of antioxidant dietary supplements has beneficial effects on the efficacy and patient outcome of therapeutic approaches.

It has been recently reported that some well‐known psychotropic drugs possess antioxidant activity as radical scavengers.^[12]^ Zolpidem, a diffuse hypnotic, is more efficient than melatonin in quenching hydroxyl and alkoxyl radicals,^[13]^ while fluoxetine, also known under the commercial name *Prozac*, is the molecule that has revolutionized the approach to depression treatment and possesses a discrete antioxidant capacity but rather exerts this added function indirectly, by increasing the levels of free serotonin, a strong radical scavenger.^[14]^ Based on these examples, the administration of these drugs may have beneficial effects, adding value to the therapeutic approach. These results, which stem originally from clinical observation, have been rationalized at chemical level using *in silico* approaches. Despite the highly complex physiological environment, molecular studies on the antioxidant capacity of a substance are a valuable first approach to *in vivo* and clinical testing. One of the most important advantages of *in silico* studies (rather than *in vitro* ones) is the possibility of screening a large number of molecules at a reduced time and price. In addition, a detailed computational analysis, carried out at an accurate level of theory, allows to rationalize the results, thus providing information and guidelines for designing more efficient antioxidants.

**Scheme 1:**
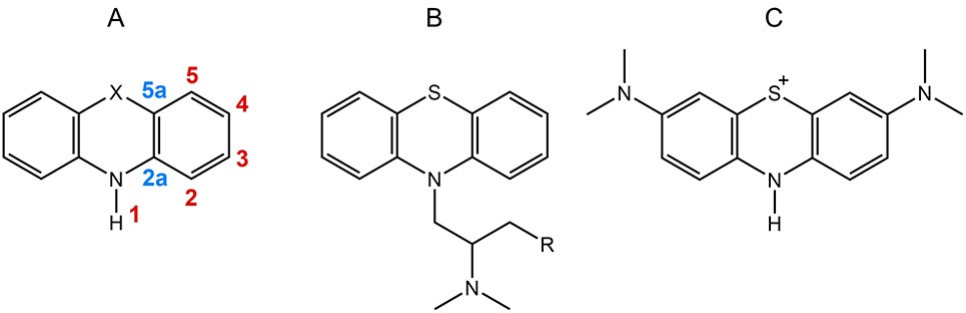
A) Phenothiazine (PS, X=S), phenoselenazine (PSE, X=Se), phenotellurazine (PTE, X=Te), B) promethazine (R=H), chlorpromethazine (R=Cl), C) methylene blue. The key reactive sites are shown in red and blue.

In this work, we have quantum chemically studied the antioxidant potential of the scaffold of a very important class of antipsychotic drugs, i. e. phenothiazines (Scheme 1, **PS**/**PSE**/**PTE**), using density functional theory (DFT). Different derivatives of these heterocyclic compounds find application in various medical fields, as antihistaminics (promethazine, Scheme 1, A), sedatives (chlorpromethazine, Scheme 1, B), anthelminthics. An important derivative is methylene blue (Scheme 1, C), which was first synthesized in 1876 and used by Ehlrich to distinguish bacteria, among which the malaria pathogen. Ehrlich proposed to use methylene blue in the treatment of malaria and, after testing, it was used for this purpose till the Second World War. Recently, it has been proposed again for malaria treatment^[15]^ due to its low cost and as attempt to combat drug resistance.^[16]^

The phenothiazine scaffold represents the ideal parent compound from which the activity can be regulated by different substituents. For example, in the phenothiazine derivatives used as antipsychotics, the sedative effects as well as the extrapyramidal side effects, can be modulated by using different substituents at nitrogen. Since we are interested in the antioxidant potential of this class of compounds, we have introduced several chemical modifications, replacing sulfur (in **PS**) with the heavier selenium and tellurium, generating phenoselenazine (**PSE**) and phenotellurazine (**PTE**), respectively. Organoselenium compounds are well‐known antioxidants, mimicking the enzymatic activity of glutathione peroxidases (GPx) and tellurium analogs[^17^, ^18^, ^19^, ^20^] are cautiously being tested for their enhanced capacity of reducing hydroperoxides, as also predicted by computational studies.[^21^, ^22^] **PSE** and **PTE** were designed as ideal “tandem” antioxidants, which can act as radical scavengers via different mechanisms, as well as GPx mimics, which can efficiently reduce H_2_O_2_ and hydroperoxides to water and alcohols, respectively, as recently proposed for selenium derivatives of fluoxetine.[^23^, ^24^]

## Materials and Methods

All the density functional theory (DFT)[^25^, ^26^] calculations have been carried out with the Gaussian 16 rev. C.01 software.^[27]^ The used exchange‐correlation (XC) functional is the M06‐2X, a hybrid meta‐GGA functional developed by Yan and Truhlar.^[28]^ It contains 54 % of exact Hartree‐Fock (HF) exchange and has been developed to give excellent results for main group thermochemistry. This has been used in combination with the following basis sets: the 6‐311++G(d,p) basis set for H, C, N, O, and S atoms, and the cc‐pVTZ for the Se and Te.[^29^, ^30^, ^31^, ^32^] A properly selected effective core potential (ECP) is also necessary for the heaviest chalcogen (Te). The former is a Pople split‐valence triple‐ζ Gaussian‐type orbitals (GTO) basis set with two polarization functions (1 additional set of d orbitals on heavy atoms and one set of p orbitals on hydrogen). The cc‐pVTZ instead is a Dunning's correlation‐consistent triple‐ζ basis set. The optimized structures of minima and transition state have been computed both in gas‐phase and in solvent. In the latter case, we have used the solvation model based on density (SMD) to emulate the physiological conditions (water) and the lipidic environment (pentyl ethanoate).^[33]^ Stationary‐point geometries have been subsequently verified with a vibrational analysis in order to assess the correct nature of the points located on the potential energy surface (PES): all normal modes of the minima have real frequencies, and, in the case of transition states, there is one normal mode associated to a single imaginary frequency which is associated with the reaction.

The aforementioned level of theory [(SMD)‐M06‐2X/6‐311++G(d,p), cc‐pVTZ(‐PP)] is compatible with the QM‐ORSA protocol used to calculate the overall antioxidant capability of the analyzed molecules.[^34^, ^35^, ^36^, ^37^, ^38^] This allows direct comparison to other antioxidant systems described in the literature, if necessary. The method consists firstly in evaluating the barrier for a given reaction with a canonical TS minimization on a first‐order saddle point. In the case of an electron transfer, the Gibbs free energy of activation is calculated according to the Marcus theory.[^39^, ^40^] Two thermal corrections are applied: the first one [Eq. (1)] is the conversion from the gas phase (1 atm, 298.15 K) to the condensed standard state (1 M, 298.15 K); the second one [Eq. (2)] is used to take into account the solvent cage effects:

(1) $${\Delta G}^{1M}={\Delta G}^{1atm}-RT\;ln\left(V_{M}\right)$$

(2) $${\Delta G}^{sol}≅{\Delta G}^{gas}-RT\;\left\{ln\left[n10^{2(n-1)}\right]-\left(n-1\right)\right\}$$

where $V_{M}$ is the molar volume and n the total of reactants moles. The latter equation [Eq. (2)] is intended to better estimate the reduced entropy loss for a transition state formation due to the solvation effects. Ignoring these two corrections can lead to a substantial underestimation of the final kinetic rate constants (up to 1800 times). The rate constants (k ) have been calculated within the Transition State Theory (TST) model with the Eyring–Polanyi equation [Eq. 3].[^41^, ^42^]

(3) $$k=\kappa \left(T\right)\frac{k_{B}T}{h}e^{-\frac{{\Delta G}^{\neq }}{RT}}$$

where $\kappa \left(T\right)$ is the Wigner transmission coefficient [Eq. (4)] used to include the one‐dimensional quantum tunneling effect.^[43]^ For some reactions that involves displacements of light atoms, i. e. HAT, the tunneling correction turned out to be a crucial factor in order to avoid underestimation of the total rate constant.^[44]^

(4) $$\kappa \left(T\right)=1+\frac{1}{24}\left[\frac{h\left|im\left(\nu^{\neq }\right)\right|}{k_{B}T}\right]$$

However, this is not the final rate constant because many reactions are so fast that the process is limited by diffusion and, the sole thermal rate constant, is no longer a good prediction of the real reaction rate. To solve this issue, the Smoluchowski equation for steady‐state solutions [Eq. (5)] in combination with the Stokes‐Einstein equation [Eq. (6)] has been used to calculate the diffusion rate constant ($k_{D}$ ):

(5) $$k_{D}=4\pi R_{AB}D_{AB}N_{A}$$

(6) $$D_{A\;or\;B}=\frac{k_{B}T}{6\pi \eta a_{A\;or\;B}}$$

where $R_{AB}$ is the reaction distance, $D_{AB}$ is the mutual diffusion coefficient of the ROS (A) and the scavenger (B), η is the solvent viscosity, $a_{A\;}$ and $a_{B}$ is the Stokes radius of A and B, respectively. Then, according to the Collins−Kimball theory [Eq. (7)],^[45]^ both the thermal and diffusion rate constants are coupled to form the total rate coefficients.

(7) $$k^{app}=\frac{k_{D}k}{k_{D}+k}$$

The branching ratios (Γ) have been calculated as well [Eq. (8)], and they represent the percentual contribution of a single mechanism to the overall antioxidant activity.

(8) $$\Gamma_{i}=\frac{100\;k_{i}^{app}}{\sum_{i}^{N}\sigma_{i}k_{i}^{app}}$$

## Results and Discussion

Three different mechanisms of radical scavenging, i. e. Hydrogen Atom Transfer (HAT), Radical Adduct Formation (RAF), and Single Electron Transfer (SET), have been analyzed assuming that they are the possible mechanisms through which ROS quenching occurs. Based on molecular symmetry, there are seven non‐equivalent active sites on the phenothiazine and its derivatives (Scheme 1): 1 active site is present on the only amino nitrogen (1); 4 active sites are aromatic carbon atoms (2, 3, 4, 5); and 2 active sites are junction carbon atoms (2a and 5a). The energetics of the three mechanisms will be described and analyzed in detail in the following paragraphs.

### Hydrogen Atom Transfer (HAT)

**Scheme 2:**
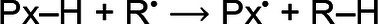
HAT mechanism where Px=PS, PSE, PTE and R^.^=HO^.^, HOO^.^, CH_3_OO^.^ (see also Scheme 1).

Hydrogen atom transfer (HAT) is the most relevant mechanism for radical quenching. This elementary process is shown in Scheme 2.

**Figure 1:**
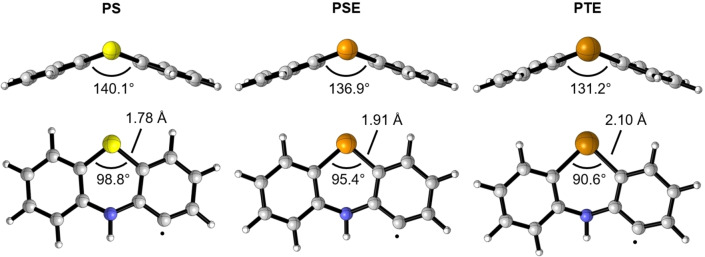
Minimum energy structure of **PS**, **PSE**, and **PTE** radicals (H abstracted from site 2) in the gas phase, computed at M06‐2X/6‐311++G(d,p), cc‐pVTZ(‐PP).

On the phenothiazine scaffold, there are 5 positions from which H^.^ can be abstracted (sites 1, 2, 3, 4, 5, Scheme 1). In all cases, the most exergonic reaction involves the most acidic hydrogen, i. e., the amino hydrogen. The reaction Gibbs free energies associated with HAT from the aromatic sites (sites 2, 3, 4, 5, Scheme 1) are mutually similar but all much less favorable than HAT from the amino group (site 1). Only in the case of the hydroxyl radical, HAT from the aromatic sites remains exergonic. As soon as peroxyl radicals are involved, HAT from the aromatic sites becomes highly endergonic and thus thermodynamically inaccessible (Table 1). The relative thermodynamic viability of the various HAT pathways, as quantified by ΔΔ*G*_r_(site)=Δ*G*_r_(site)−Δ*G*_r_(site 1) is barely affected (variations of only few kcal mol^−1^) when changing radicals, chalcogens and medium and spans from 27 to 32 kcal mol^−1^ (Figure 1).

**Table 1:** Gibbs free energy of reaction (Δ*G*_r_, in kcal mol^−1^) for the hydrogen atom transfer (HAT).^[a]^

		Δ*G*_r_ in water			Δ*G*_r_ in lipid		
ROS	Site	S	Se	Te	S	Se	Te
HO^.^	1	−39.9	−39.2	−37.4	−37.6	−36.7	−34.7
	2	−7.4	−7.7	−8.1	−5.7	−6.0	−6.4
	3	−8.5	−8.6	−8.6	−6.4	−6.6	−6.5
	4	−7.8	−8.0	−8.1	−5.7	−5.9	−6.0
	5	−8.1	−8.8	−9.8	−6.1	−6.8	−7.8
HOO^.^	1	−7.6	−7.0	−5.2	−5.1	−4.1	−2.1
	2	24.8	24.5	24.1	26.9	26.5	26.2
	3	23.8	23.6	23.6	26.1	26.0	26.0
	4	24.5	24.2	24.2	26.8	26.6	26.6
	5	24.1	23.4	22.4	26.4	25.8	24.7
CH_3_OO^.^	1	−6.3	−5.6	−3.8	−3.4	−2.4	−0.4
	2	26.2	25.8	25.4	28.6	28.2	27.8
	3	25.1	25.0	25.0	27.8	27.7	27.7
	4	25.8	25.6	25.5	28.5	28.3	28.3
	5	25.5	24.8	23.8	28.1	27.5	26.4

[a] Computed at (SMD)‐M06‐2X/6‐311++G(d,p), cc‐pVTZ(‐PP). For details, see Materials and Methods.

From a thermodynamic point of view, HAT from the amino group is the favored pathway for all the substrates (**PS**, **PSE**, and **PTE**), attacking radicals and under any physiological condition (different medium). For this reason, the potential energy surface (PES) has been thoroughly analyzed for this site.

The HO^.^ radical is the most reactive one among our model radicals and the only one that shows exergonic HAT reactions for all sites of the three studied scaffolds. When considering the reactivity toward HOO^.^ and CH_3_OO^.^ radicals, HAT becomes prohibitively endergonic for the aromatic sites and the only possible pathway that remains is the H abstraction from the amino site (site 1). The aromatic sites are always less reactive than the amino site and they all have similar reaction energies. Nearly no variation emerges when changing the chalcogen but, in general, the most efficient system is **PS**, followed by **PSE**, and **PTE**.

The process in the polar environment is completely barrierless regardless of the radical (Table 2); no effect of the chalcogen is found. The absence of any noticeable reaction barrier has been confirmed by the strictly decreasing minimum energy reaction path connecting the reactants to the products via nudged elastic band (NEB) calculation as well as by other authors.^[46]^ However, in lipid media, the barriers are appreciable: they are rather small for HO^.^ but increase significantly for HOO^.^ and CH_3_OO^.^ (from 18 kcal mol^−1^ to almost 20 kcal mol^−1^, depending on the chalcogen).

**Table 2:** Gibbs free energy of activation (Δ*G* ^≠^, in kcal mol^−1^) for hydrogen atom transfer (HAT) from site 1.

	Δ*G* ^≠^ in water			Δ*G* ^≠^ in lipid		
ROS	S	Se	Te	S	Se	Te
HO^.^	0.0	0.0	0.0	6.3	6.2	6.1
HOO^.^	0.0	0.0	0.0	17.9	18.2	19.0
CH_3_OO^.^	0.0	0.0	0.0	18.7	19.0	19.6

[a] Computed at (SMD)‐M06‐2X/6‐311++G(d,p), cc‐pVTZ(‐PP). For details, see Materials and Methods.

### Radical Adduct Formation (RAF)

**Scheme 3:**
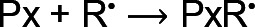
RAF mechanism where Px=PS, PSE, PTE and R^.^=HO^.^, HOO^.^, CH_3_OO^.^ (see also Scheme 1).

The Radical Adduct Formation (RAF) is another important mechanism that leads to the formation of a single adduct as a product (Scheme 3).

In the case of the phenothiazine scaffold, this reaction mechanism is rather essential because the HAT mechanism is not well suited for the aromatic positions since the hydrogen transfer from the benzene rings (to a ROS) disrupts the ring aromaticity. Furthermore, the RAF mechanism may involve up to 6 sites (2a, 2, 3, 4, 5, 5a) on each aromatic ring instead of only 4 (in HAT).

From a thermodynamic point of view, sites 5a and 2 show the most exergonic reactions (Table 3). On the other hand, the three worst sites are 5 and 3. Sites 2a and 4 show intermediate thermodynamic feasibility. This trend holds true for all the studied antioxidants (**PS**, **PSE**, **PTE**) and for all the radicals.

**Table 3:** Gibbs free energy of reaction (Δ*G*_r_, in kcal mol^−1^) for radical adduct formation (RAF).^[a]^

		Δ*G*_r_ in water			Δ*G*_r_ in lipid		
ROS	Site	S	Se	Te	S	Se	Te
HO^.^	2a	−9.8	−9.4	−8.6	−9.0	−8.4	−7.4
	2	−10.1	−10.3	−10.5	−10.4	−10.8	−10.9
	3	−8.3	−8.2	−7.6	−8.2	−8.1	−7.2
	4	−9.9	−10.2	−10.2	−9.7	−9.9	−9.6
	5	−8.1	−8.2	−8.6	−7.8	−8.1	−8.5
	5a	−13.4	−13.2	−38. ⋅7^[b]^	−12.6	−12.7	−39.8^[b]^
HOO^.^	2a	15.6	15.8	16.4	17.6	18.1	19.1
	2	15.4	14.9	14.5	16.5	16.4	15.8
	3	16.3	16.1	17.3	18.0	18.1	19.0
	4	14.8	14.3	14.9	16.8	16.4	16.7
	5	17.1	16.7	16.5	18.8	18.5	18.0
	5a	12.3	11.9	−9.1^[b].^	14.3	13.5	−8.8^[b]^
CH_3_OO^.^	2a	18.4	18.7	19.7	21.6	21.9	23.2
	2	17.9	17.3	17.1	19.9	19.5	19.6
	3	19.0	19.3	19.9	21.6	21.7	22.2
	4	17.4	17.3	17.6	20.2	20.2	20.3
	5	19.6	19.4	19.3	22.3	21.9	21.3
	5a	14.6	14.2	−6.7^[b].^	17.1	16.6	−5.3^[b]^

[a] Computed at (SMD)‐M06‐2X/6‐311++G(d,p), cc‐pVTZ(‐PP). For details, see Materials and Methods. [b] Reaction leads to central ring opening; original antioxidant structure is therefore no longer recoverable.

Also, in this case, the most reactive radical is HO^.^: the reactions are highly exergonic for all sites and for all the three chalcogens and media. HOO^.^ and CH_3_OO^.^ show similar reactivity but all the involved reactions are thermodynamically disfavored, regardless of the chalcogens and media. The trend found for Δ*G*_r_ is recovered for Δ*G* ^≠^ as well (Table 4): the barriers associated to the different sites roughly follow the previously described trend in reaction energies: the smaller barriers are calculated for sites 5a and 2a, the highest ones for sites 5 and 3.

**Table 4:** Gibbs free energy of activation (Δ*G* ^≠^, in kcal mol^−1^) for radical adduct formation (RAF).^[a]^

		Δ*G* ^≠^ in water			Δ*G* ^≠^ in lipid		
ROS	Site	S	Se	Te	S	Se	Te
HO^.^	2a	4.8	4.5	5.1	9.2	9.5	10.0
	2	6.0	5.6	6.0	8.1	7.8	8.0
	3	8.1	7.5	8.3	10.4	10.3	10.5
	4	4.8	4.8	5.4	8.4	8.4	8.5
	5	9.1	8.9	8.8	10.7	10.4	9.7
	5a	4.2	4.5	4.9^[b]^	8.5	8.6	9.1^[b]^
HOO^.^	2a	22.6	22.2	22.8	25.9	26.4	26.6
	2	23.2	22.9	22.6	25.6	25.5	25.4
	3	24.7	24.3	25.1	27.4	27.3	27.6
	4	21.9	21.8	21.8	25.5	25.2	25.3
	5	25.7	25.7	25.8	28.1	27.9	27.7
	5a	21.4	21.1	21.6^[b]^	25.4	25.3	25.5^[b]^
CH_3_OO^.^	2a	25.0	24.9	25.3	29.8	29.9	30.6
	2	25.1	24.9	23.7	28.7	28.5	27.7
	3	27.0	26.9	27.2	30.2	30.6	30.9
	4	25.0	24.4	24.7	29.4	29.1	29.1
	5	28.3	28.2	28.0	31.6	31.5	31.4
	5a	23.5	23.7	23.7^[b].^	28.3	28.4	28.9^[b]^

[a] Computed at (SMD)‐M06‐2X/6‐311++G(d,p), cc‐pVTZ(‐PP). For details, see Materials and Methods. [b] Reaction leads to central ring opening; original antioxidant structure is therefore no longer recoverable.

Furthermore, the lowest barriers are computed for HO^.^, followed by HOO^.^ and CH_3_OO^.^. Like in the HAT mechanism, the differences in terms of Δ*G*_r_ and Δ*G* ^≠^ between the polar and apolar solvent are not particularly pronounced because the products of both processes are neither charged nor highly polarized. Hence, there is no additional stabilization of products rather than reagents due to the solvation effect. However, a general trend is noticed because in lipid media, the reactions are slightly more endergonic and the barriers tend to be higher.

It is worth to notice that in **PTE**, the attack of the ROS to site 5a is not possible: the process involves the opening of the ring and the subsequent and irreversible loss of the original antioxidant molecule. This happens with all the three screened radicals and in both solvents.

### Single Electron Transfer (SET)

**Scheme 4:**
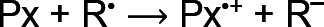
SET mechanism where Px=PS, PSE, PTE and R^.^=HO^.^, HOO^.^, CH_3_OO^.^.

The Single Electron Transfer (SET) is the only mechanism that doesn't require any nuclear displacements and, for this reason, the canonical minimization procedure used for the transition state localization is impracticable. However, Marcus theory is well suited for this purpose. The mechanism is reported in Scheme 4.

In gas phase, this mechanism is highly unlikely due to the large positive Δ*G*_r_ values (in the best‐case scenario, i. e. **PS**+OH^.^, the reaction is neatly endergonic and Δ*G*_r_ exceeds 120 kcal mol^−1^). The reason can be ascribed to the formation of highly destabilized products, i. e. charged radical species. Thus, the overall contribution of SET to scavenging activity in gas phase and in lipid media (or in any other non‐polar solvent) is negligible.

However, this reaction mechanism is possible in water where the charged products are strongly stabilized due to the polar environment, and the SET becomes highly exergonic in the case of HO^.^ ROS (Table 5). As for the previously described mechanisms, this radical is the most active one, regardless the involved chalcogen and medium. Conversely, the processes involving hydroperoxyl (HOO^.^) and methyl peroxyl radical (CH_3_OO^.^) are much more endergonic and, thus, disfavored.

**Table 5:** Gibbs free energy of reaction (Δ*G*_r_, in kcal mol^−1^) for single electron transfer (SET).^[a]^

	Δ*G*_r_ in water			Δ*G*_r_ in lipid		
ROS	S	Se	Te	S	Se	Te
HO^.^	−10.6	−13.0	−16.7	33.3	31.0	28.9
HOO^.^	11.5	9.0	5.3	52.7	50.4	48.3
CH_3_OO^.^	13.3	10.9	7.2	54.0	51.7	49.5

[a] Computed at (SMD)‐M06‐2X/6‐311++G(d,p), cc‐pVTZ(‐PP). For details, see Materials and Methods.

In water, the reaction with HO^.^ is almost barrierless for **PS** and **PSE** (Table 6) and, for this reason, SET significantly contributes to the overall antioxidant activity. Two details are remarkable: the first one is related to the observed chalcogen trend and the second one deals with the Marcus region for two particular cases. SET is the first mechanism with a clear trend in exergonicity and in terms of transition states energies: tellurium‐based systems appear to be more favored from both thermodynamic and kinetic points of view. The presence of the lighter chalcogen in **PS**, instead, is associated to the less efficient SET, similarly to the **PSE** case. The only exception in the barriers trend is found when comparing the cases of **PSE** and **PTE** with the hydroxyl radical in water. This can be explained because the reorganization energy is in both cases much smaller compared to the absolute value of Δ*G*_r_ and therefore, these two processes occur in the Marcus inverted region, where the greater the exergonicity of reaction, the larger the barrier.

**Table 6:** Gibbs free energy of activation (Δ*G* ^≠^, in kcal mol^−1^) for single electron transfer (SET).^[a]^

	Δ*G* ^≠^ in water			Δ*G* ^≠^ in lipid		
ROS	S	Se	Te	S	Se	Te
HO^.^	0.0	0.1^[b]^	5.7^[b]^	46.6	41.2	50.7
HOO^.^	12.9	11.2	7.4	63.6	59.5	61.5
CH_3_OO^.^	14.3	12.4	8.6	65.6	61.4	63.6

[a] Computed at (SMD)‐M06‐2X/6‐311++G(d,p), cc‐pVTZ(‐PP). For details, see Materials and Methods. [b] Reaction occurs in Marcus inverted region because reorganization energy λ is smaller than the absolute value of Δ*G*_r_.

### Direct oxidation of the chalcogen center

**Scheme 5:**
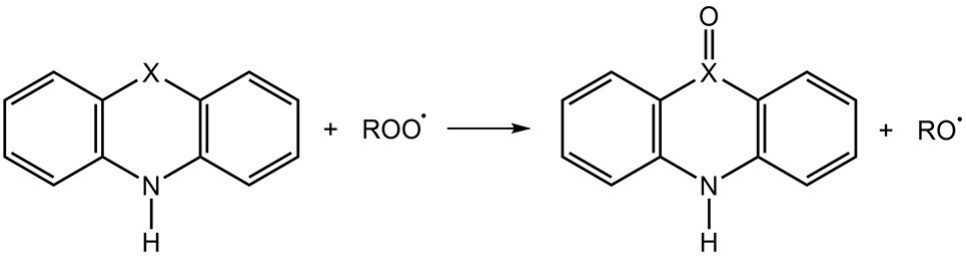
Direct oxidation of phenothiazine (PS, X=S), phenoselenazine (PSE, X=Se) and phenotellurazine (PTE, X=Te) by a generic peroxyl radicals ROO^.^.

In the previous paragraphs, we have seen how different ROSs can be quenched with different mechanisms and by different substrates. From this analysis, it emerges that the shortest‐live and smallest attacking radical, i. e. hydroxyl radical, is the more efficient regardless of the media and the involved chalcogen. The high reactivity‐poor selectivity of HO^.^ is well known in literature. This ROS is an extremely effective one‐electron oxidizing agent and the involved reactions are commonly limited by its diffusion (*k*>10^9^ M^−1^ s^−1^).[^47^, ^48^, ^49^] A very short half‐life (10^−9^ s)^[50]^ and a large and positive one‐electron reduction potential (2.31 V at physiological pH)^[51]^ demonstrates its high reactivity and, consequently, the low selectivity towards the substrate. In general, alkoxyl radicals RO^.^ tends to retain this characteristic but in a much more modest way: they are less active compared to HO^.^ but they are more reactive than peroxyl radicals ROO^.^. A conversion of the latter is possible with direct oxidation of the chalcogen center on **PS**, **PSE**, and **PTE** according to Scheme 5.

From a merely theoretical point of view, this reaction path could in principle enhance the overall antioxidant activity of **PS**, **PSE**, and **PTE** due to the conversion of the ROS from a relatively poorly reactive peroxyl radical to an alkoxyl radical. In the particular case of hydroperoxyl radical, the product is an extremely reactive hydroxyl radical molecule. The generated alkoxyl radical can then react once again via one of the previously investigated mechanism.^[52]^ The nature of the newly formed oxidized molecules and, in particular, of the sulfoxide, selenoxide and telluroxide bond X=O (X=S, Se, Te) is best described by a polarized σ bond rather than a simple double bond. For this reason, due to the formation of a positive partial charge on X and a negative one on O, the molecule does not exhibit a classical hypervalency. The strength of the X=O bond is related to the involved electrostatic interactions between the two atoms and tend to decrease from X=S to X=Te.^[53]^ In our systems, the direct oxidation is an exergonic process for all the analyzed cases (Table 7).

**Table 7:** Gibbs free energy of reaction (Δ*G*_r_, in kcal mol^−1^) for direct oxidation of the chalcogen by HOO^.^ and CH_3_OO^.^ radicals.^[a]^

	Δ*G*_r_ in water			Δ*G*_r_ in lipid		
ROS	S	Se	Te	S	Se	Te
HOO^.^	−7.8	−12.0	−18.4	−0.2	−2.3	−6.2
CH_3_OO^.^	−14.7	−18.9	−25.2	−6.9	−9.1	−12.9

[a] Computed at (SMD)‐M06‐2X/6‐311++G(d,p), cc‐pVTZ(‐PP). For details, see Materials and Methods.

The reaction is thermodynamically favored in physiological environment and in the presence of alkylperoxyl radicals. All the previously explored mechanisms (HAT, RAF, and SET) in which the HOO^.^ and CH_3_OO^.^ radicals are being involved show much more endergonic reactions. However, direct oxidation requires rather prohibitive activation energies under standard conditions (Table 8). In the best scenario (**PTE** with HOO^.^ in water), Δ*G* ^≠^ is about 12 kcal mol^−1^ and considering a barrierless process, such as HAT in our systems, the contribution to the overall antioxidant activity of a mechanism involving the direct oxidation of the chalcogen and subsequent scavenging has to be considered extremely limited.

**Table 8:** Gibbs free energy of activation (Δ*G* ^≠^, in kcal mol^−1^) for direct oxidation of the chalcogen by HOO^.^ and CH_3_OO^.^.^[a]^

	Δ*G* ^≠^ in water			Δ*G* ^≠^ in lipid		
ROS	S	Se	Te	S	Se	Te
HOO^.^	31.5	23.6	12.2	38.2	30.8	20.8
CH_3_OO^.^	34.4	26.4	15.5	42.1	35.7	24.8

[a] Computed at (SMD)‐M06‐2X/6‐311++G(d,p), cc‐pVTZ(‐PP). For details, see Materials and Methods.

### Kinetic constants and antioxidant activity

According to the QM‐ORSA protocol here adopted and described in the material and methods section, the kinetic constants have been calculated for both solvents in order to assess the overall antioxidant capability and to make comparison with data reported in literature for analogous systems. The apparent kinetic constants in water were computed and are summarized in Table 9. For HAT mechanism, only site 1 has been taken into account: the kinetic constant of transfers involving aromatic hydrogens are orders of magnitude smaller when compared to the one of the amino sites. HAT is the primary mechanism for the larger radicals (hydroperoxyl and methyl peroxyl radical) for **PS** and **PSE**. The RAF mechanism, instead, is the favored mechanism for the hydroxyl radical and this could be mainly ascribed to the number of available positions on the aromatic rings (also considering the degenerate pathways due to the symmetry) and to the small reaction barriers. Unfortunately, the barriers increase significantly with the peroxyl radicals and thus, the contribution of RAF to the overall activity is negligible for HOO^.^ and CH_3_OO^.^. However, the most efficient reaction mechanism with the combination of these two radicals and **PTE** is SET.

**Table 9:** Kinetic constants and branching ratios (in M^−1^ s^−1^, %) for all analyzed mechanisms in water at 298.15 K.

		k_app_ in water		
	ROS	**PS**	**PSE**	**PTE**
HAT	HO^.^	2.61 ⋅ 10^9^ (3 %)^[a]^	2.63 ⋅ 10^9^ (3 %)^[a]^	2.58 ⋅ 10^9^ (3 %)^[a]^
	HOO^.^	1.96 ⋅ 10^9^ (100 %)^[a]^	1.98 ⋅ 10^9^ (97 %)^[a]^	1.96 ⋅ 10^9^ (23 %)^[a]^
	CH_3_OO^.^	1.69 ⋅ 10^9^ (100 %)^[a]^	1.69 ⋅ 10^9^ (99 %)^[a]^	1.68 ⋅ 10^9^ (32 %)^[a]^
RAF	HO^.^	6.94 ⋅ 10^10^ (86 %)^[a]^	7.24 ⋅ 10^10^ (87 %)^[a]^	7.10 ⋅ 10^10^ (87 %)^[a]^
	HOO^.^	1.67 ⋅ 10^1^ (0 %)	2.60 ⋅ 10^1^ (0 %)	1.55 ⋅ 10^1^ (0 %)
	CH_3_OO^.^	3.72 ⋅ 10^−1^ (0 %)	3.53 ⋅ 10^−1^ (0 %)	5.09 ⋅ 10^−1^ (0 %)
SET	HO^.^	8.53 ⋅ 10^9^ (11 %)^[a]^	8.47 ⋅ 10^9^ (10 %)^[a]^	8.43 ⋅ 10^9^ (10 %)^[a]^
	HOO^.^	3.62 ⋅ 10^6^ (0 %)	7.02 ⋅ 10^7^ (3 %)	6.76 ⋅ 10^9^ (77 %)^[a]^
	CH_3_OO^.^	3.85 ⋅ 10^5^ (0 %)	9.12 ⋅ 10^6^ (1 %)	3.39 ⋅ 10^9^ (68 %)^[a]^
overall	HO^.^	8.05 ⋅ 10^10 [a]^	8.35 ⋅ 10^10 [a]^	8.21 ⋅ 10^10 [a]^
	HOO^.^	1.96 ⋅ 10^9 [a]^	2.05 ⋅ 10^9 [a]^	8.72 ⋅ 10^9 [a]^
	CH_3_OO^.^	1.69 ⋅ 10^9 [a]^	1.70 ⋅ 10^9 [a]^	5.07 ⋅ 10^9 [a]^

[a] Diffusion‐controlled reaction.

The global antioxidant activity is given by the sum of the k_app_ for all considered mechanisms: in physiological conditions, the sum of all thermal constants exceeds the value estimated for diffusion and therefore, all processes are limited by the latter. Unfortunately, this rules out the fine‐tuning possibility of the antioxidant capacities by a simple modification of the chalcogen and therefore, the scavenging capacity is determined by the radical‘s ability to diffuse in water.

In a lipidic environment, the situation is less diversified than in the water system (Table 10): the HAT mechanism is the favored reaction pathway only for the larger radicals (HOO^.^ and CH_3_OO^.^) and, as seen before, the most active radical reacts via RAF. In this case, the branching ratios are highly independent from the chalcogen in the antioxidant. The lipid medium is not able to stabilize the charged products deriving from SET and, as a consequence, this mechanism is energetically unfavorable both from the thermodynamic and kinetic points of view.

**Table 10:** Kinetic constants and branching ratios (in M^−1^ s^−1^, %) for all analyzed mechanisms in pentyl ethanoate at 298.15 K.

		k_app_ in lipid		
	Radical	**PS**	**PSE**	**PTE**
HAT	HO^.^	2.87 ⋅ 10^9^ (7 %)^[a]^	2.91 ⋅ 10^9^ (7 %)^[a]^	2.81 ⋅ 10^9^ (8 %)^[a]^
	HOO^.^	5.63 ⋅ 10^3^ (100 %)	3.66 ⋅ 10^3^ (100 %)	1.24 ⋅ 10^3^ (100 %)
	CH_3_OO^.^	1.55 ⋅ 10^3^ (100 %)	9.06 ⋅ 10^2^ (100 %)	3.95 ⋅ 10^2^ (100 %)
RAF	HO^.^	3.80 ⋅ 10^10^ (93 %)^[a]^	3.78 ⋅ 10^10^ (93 %)^[a]^	3.33 ⋅ 10^10^ (92 %)^[a]^
	HOO^.^	4.32 ⋅ 10^−2^ (0 %)	5.04 ⋅ 10^−2^ (0 %)	4.47 ⋅ 10^−2^ (0 %)
	CH_3_OO^.^	1.86 ⋅ 10^−4^ (0 %)	2.13 ⋅ 10^−4^ (0 %)	3.50 ⋅ 10^−4^ (0 %)
SET	HO^.^	8.80 ⋅ 10^−19^ (0 %)	6.84 ⋅ 10^−15^ (0 %)	7.45 ⋅ 10^−22^ (0 %)
	HOO^.^	2.60 ⋅ 10^−31^ (0 %)	2.87 ⋅ 10^−28^ (0 %)	9.60 ⋅ 10^−30^ (0 %)
	CH_3_OO^.^	9.24 ⋅ 10^−33^ (0 %)	1.13 ⋅ 10^−29^ (0 %)	2.65 ⋅ 10^−31^ (0 %)
overall	HO^.^	4.09 ⋅ 10^10 [a]^	4.07 ⋅ 10^10 [a]^	3.61 ⋅ 10^10 [a]^
	HOO^.^	5.63 ⋅ 10^3^	3.66 ⋅ 10^3^	1.24 ⋅ 10^3^
	CH_3_OO^.^	1.55 ⋅ 10^3^	9.06 ⋅ 10^2^	3.95 ⋅ 10^2^

[a] Diffusion‐controlled reaction.

The antioxidant capacity in apolar environment results comparable to that computed in water only when considering the hydroxyl radical. The selectivity towards peroxyl radicals is strongly reduced.

In order to better understand the calculated kinetic constants for **PS**, **PSE**, and **PTE**, a comparison to few well‐known antioxidant molecules has been made (Table 11). The majority of the theoretical rate constants in the Table 11 were calculated with the M05‐2X functional: a direct comparison to M06‐2X is possible thanks to the excellent agreement between these two XC functionals.[^13^, ^14^, ^34^, ^36^]

**Table 11:** Calculated and experimental kinetic rate constants (in M^−1^ s^−1^) for the quenching activity of several antioxidant molecules towards different ROSs.

Substrate	ROS	Solvent	*k*_calc_	*k*_exp_
PS	HO^.^	Aqueous	8.05 ⋅ 10^10^	
PSE	HO^.^	Aqueous	8.35 ⋅ 10^10^	
PTE	HO^.^	Aqueous	8.21 ⋅ 10^10^	
Glutathione	HO^.^	Aqueous	7.68 ⋅ 10^9^ ^[54]^	8.72 ⋅ 10^9^[^55^, ^56^, ^57^]
Glutathione	CH_3_O^.^	Aqueous	5.89 ⋅ 10^8^ ^[54]^	9.00 ⋅ 10^8^ ^[58]^
Glutathione	HOO^.^	Aqueous	2.69 ⋅ 10^7^ ^[54]^	
Glutathione	CH_3_OO^.^	Aqueous	2.02 ⋅ 10^4^ ^[54]^	
Sesamol	HO^.^	Aqueous	2.37 ⋅ 10^10^ ^[59]^	1.10 ⋅ 10^10^ ^[60]^
Sesamol	HOO^.^	Aqueous	6.36 ⋅ 10^7^ ^[59]^	
Caffeine	HO^.^	Aqueous	2.15 ⋅ 10^9^ ^[61]^	5.60 ⋅ 10^9^[^62^, ^63^, ^64^]
Melatonin	HO^.^	Aqueous	1.85 ⋅ 10^10^[^65^, ^66^]	3.04 ⋅ 10^10^[^67^, ^68^, ^69^, ^70^, ^71^]
DHMBA^[a]^	HOO^.^	Aqueous	1.34 ⋅ 10^9^ ^[72]^	
Capsaicin	ROO^.^	Mixed	6.50 ⋅ 10^3^ ^[73]^	5.60 ⋅ 10^3^ ^[74]^
Tyrosol	ROO^.^	Aqueous	4.30 ⋅ 10^3^ ^[75]^	9.40 ⋅ 10^3^ ^[76]^
Trolox	HO^.^	Aqueous	2.78 ⋅ 10^10^ ^[74]^	8.10 ⋅ 10^10^ ^[77]^
Edaravone	HO^.^	Aqueous	1.35 ⋅ 10^10^ ^[78]^	1.93 ⋅ 10^9^[^79^, ^80^]

[a] 3,5‐dihydroxy‐4‐methoxybenzyl alcohol.

In aqueous solution, the activity towards the HO^.^ radical shows reaction rates that are approaching the diffusion rate limit: this is a common point also found for glutathione, sesamol, caffeine, melatonin, DHMBA, Trolox, and edaravone. Calculating an accurate kinetic constant strongly depends on the approximation used to estimate the rate of diffusion in a particular media: the most challenging parameter to assess and for which, to the best of our knowledge, there is no accurate technique of evaluation, is the reactants Stokes radius that defines both the diffusion coefficients.

Changing ROS to HOO^.^ or ROO^.^ usually leads to decreased antioxidant capabilities; however, this heavily depends on the substrate structure. For instance, **PS**, **PSE**, and **PTE** show no HAT barrier for the amino hydrogen (site 1) and this is the only reason explaining the outstanding performance towards less active radicals, i. e. HOO^.^ and CH_3_OO^.^. In a similar fashion, we can find an analogy with the 3,5‐dihydroxy‐4‐methoxybenzyl alcohol (DHMBA): one of the hydroxy groups exhibits a barrierless process via HAT and, as a primary consequence, the involved kinetic rate constant for the HOO^.^ quenching easily reaches the diffusion regime. On the other hand, in lipid media, where the NH hydrogen abstraction becomes an activated process (especially for HOO^.^ and CH_3_OO^.^), the overall activity of **PS**, **PSE**, and **PTE** is close to what we find in capsaicin or tyrosol. Finally, another general observation, which is also in agreement with the data reported literature for other scavengers, is the poor selectivity of alkoxyl radicals, especially HO^.^, versus the low reactivity of the peroxyl radicals.

## Conclusions

In this work, we have analyzed *in silico* the scavenging activity of the phenothiazine scaffold and its selenium and tellurium derivatives. The idea of chemically modifying this system by introducing selenium, which yields the parent molecular structure of a well‐known class of psychotropic and antihistaminic drugs, aims at improving its antioxidant action with beneficial therapeutic outcomes.

In contrast to the situation of selenofluoxetine vs fluoxetine,^[23]^ the presence of a different chalcogen than sulfur does not lead to an enhanced antioxidant activity via any of the three scavenging mechanisms considered, i. e., hydrogen atom transfer (HAT), radical adduct formation (RAF) and single electron transfer (SET). In addition, we have explored an alternative pathway via direct oxidation of the chalcogen followed by the ROS quenching mechanisms which, however, appears to be unviable due to unfavorable energetics.

We conclude that the phenothiazine scaffold is a rather good scavenger for HO^.^, comparable to well‐established antioxidants like melatonin and Trolox, but not for peroxyl radicals. Due to the complexity and computational cost of performing fully quantum mechanical analyses of the ROS scavenging activity in large and flexible molecules, we are currently tackling both the thermodynamic and the kinetic aspects using a machine learning approach and the data here obtained have been included in the training dataset.^[81]^

## Conflict of interest

The authors declare no conflict of interest.

## Supporting information

As a service to our authors and readers, this journal provides supporting information supplied by the authors. Such materials are peer reviewed and may be re‐organized for online delivery, but are not copy‐edited or typeset. Technical support issues arising from supporting information (other than missing files) should be addressed to the authors.

Supporting Information
