# Supplementary material for: Radical Scavenging Potential of the Phenothiazine Scaffold: A Computational Analysis
Source: ChemMedChem. 2021 Oct 15;16(24):3763–71. doi: 10.1002/cmdc.202100546 (PMC9292796; doi:10.1002/cmdc.202100546)
Supplement: Supplementary file 1 — Supporting Information [file CMDC-16-3763-s001.pdf]

ChemMedChem

Supporting Information

Radical Scavenging Potential of the Phenothiazine Scaffold: A Computational Analysis

Marco Dalla Tiezza, Trevor A. Hamlin, F. Matthias Bickelhaupt,* and Laura Orian*

Table of Contents

Table 1. Cartesian Coordinates (in Å) and electronic energies (in Ha), computed at SMD-M06-2X/6-311++G(d,p), cc-pVTZ(-PP). S1

General S1

Hydrogen Atom Transfer S4

Radical Adduct Formation S14

Single Electron Transfer S78

Direct Oxidation S80

Table 1. Cartesian Coordinates (in Å), electronic energies (in Ha), Gibbs free energy (in Ha), zero-point correction (ZPC, in Ha), thermal correction to energy (TCE, in Ha), enthalpy (TCH, in Ha) and Gibbs free energy (TCG, in Ha) computed at SMD-M06-2X/6-311++G(d,p), cc-pVTZ(-PP).

GENERAL

PS

Solvent = Water
E = -915.567650
G = -915.424278
ZPC = 0.179392
TCE = 0.189557
TCH = 0.190501
TCG = 0.143373
N_{imag} = 0

S	-0.000000	-1.712462	0.562472
N	0.000000	1.272364	0.744913
C	1.213728	0.734947	0.285050
C	1.348434	-0.646169	0.107207
C	-3.638037	-0.353054	-0.593145
C	-3.511844	1.020672	-0.403483
C	-2.303934	1.564487	0.017380
C	-1.213728	0.734947	0.285050
C	-1.348434	-0.646169	0.107207
C	-2.548278	-1.183632	-0.349087
C	2.303934	1.564487	0.017380
C	2.548278	-1.183632	-0.349087
H	-4.574842	-0.778070	-0.931605
H	-4.352607	1.676691	-0.595259
H	-2.194253	2.635672	0.150858
C	3.511844	1.020672	-0.403483
C	3.638037	-0.353054	-0.593145
H	4.352607	1.676691	-0.595259
H	4.574842	-0.778070	-0.931605
H	0.000000	2.286223	0.764947
H	2.194253	2.635672	0.150858
H	2.631283	-2.254484	-0.496969
H	-2.631283	-2.254484	-0.496969

Solvent = Pentyl Ethanoate
E = -915.573856
G = -915.430581
ZPC = 0.179322
TCE = 0.189512
TCH = 0.190456
TCG = 0.143275
N_{imag} = 0

S	-0.000000	-1.707896	0.555116
N	-0.000000	1.279470	0.708517
C	1.217623	0.738434	0.272827
C	1.350311	-0.644186	0.100770
C	-3.648702	-0.358275	-0.575393
C	-3.525731	1.015035	-0.387493
C	-2.314384	1.562973	0.017144
C	-1.217624	0.738434	0.272827
C	-1.350311	-0.644186	0.100770
C	-2.553054	-1.184159	-0.342479
C	2.314384	1.562974	0.017143
C	2.553055	-1.184159	-0.342478
H	-4.587824	-0.787030	-0.902931
H	-4.371241	1.668127	-0.569064
H	-2.209454	2.635360	0.146474
C	3.525730	1.015035	-0.387494
C	3.648703	-0.358275	-0.575392
H	4.371240	1.668128	-0.569066
H	4.587825	-0.787030	-0.902929
H	0.000000	2.290480	0.761829
H	2.209453	2.635360	0.146472
H	2.633347	-2.255606	-0.488391
H	-2.633346	-2.255606	-0.488393

PSE

Solvent = Water
E = -2919.002843
G = -2918.861275
ZPC = 0.178635
TCE = 0.189175
TCH = 0.190119
TCG = 0.141568
N_{imag} = 0

Se	1.624815	-0.340782	0.000000
N	-1.420799	-0.827597	0.000000
C	-0.964264	-0.299636	1.221945
C	0.384022	0.019306	1.410588
C	-0.068941	0.694975	-3.683241
C	-1.410085	0.363834	-3.504844
C	-1.858414	-0.114657	-2.279942
C	-0.964264	-0.299636	-1.221945
C	0.384022	0.019306	-1.410588
C	0.823180	0.533034	-2.627788
C	-1.858414	-0.114657	2.279942
C	0.823180	0.533034	2.627788
H	0.281727	1.078395	-4.633557
H	-2.115836	0.489319	-4.317470
H	-2.905789	-0.356386	-2.131685
C	-1.410085	0.363834	3.504844
C	-0.068941	0.694975	3.683241
H	-2.115836	0.489319	4.317470
H	0.281727	1.078395	4.633557
H	-2.422912	-0.985019	-0.000000
H	-2.905789	-0.356386	2.131685
H	1.869314	0.789934	2.750616
H	1.869314	0.789934	-2.750616

Solvent = Pentyl Ethanoate
E = -2919.006939
G = -2918.865604
ZPC = 0.178466
TCE = 0.189054
TCH = 0.189999
TCG = 0.141335
N_{imag} = 0

Se	1.616716	-0.336588	0.000000
N	-1.435495	-0.782081	-0.000000
C	-0.969558	-0.282829	1.226962
C	0.382092	0.024505	1.414073
C	-0.056090	0.669905	-3.698620
C	-1.398711	0.347071	-3.523367
C	-1.855868	-0.109441	-2.293620
C	-0.969558	-0.282829	-1.226962
C	0.382092	0.024505	-1.414073
C	0.828649	0.519470	-2.635418
C	-1.855868	-0.109441	2.293620
C	0.828649	0.519470	2.635418
H	0.301703	1.038187	-4.652379
H	-2.099375	0.462864	-4.342041
H	-2.906028	-0.342421	-2.149461
C	-1.398711	0.347071	3.523367
C	-0.056090	0.669905	3.698620
H	-2.099375	0.462864	4.342041
H	0.301703	1.038187	4.652379
H	-2.431754	-0.964958	-0.000000
H	-2.906028	-0.342421	2.149461
H	1.876560	0.771040	2.755957
H	1.876560	0.771040	-2.755957

PTE

Solvent = Water
E = -785.314872
G = -785.175169
ZPC = 0.177715
TCE = 0.188556
TCH = 0.189500
TCG = 0.139703
N_{imag} = 0

Te	1.591483	0.221797	0.000000
N	-1.552670	0.909671	-0.000000
C	-1.180621	0.329459	-1.232476
C	0.132505	-0.080364	-1.489442
C	-0.498760	-0.745325	3.729409
C	-1.801783	-0.320110	3.480985
C	-2.144034	0.198106	2.238988
C	-1.180621	0.329459	1.232476
C	0.132505	-0.080364	1.489442
C	0.459254	-0.634633	2.726471
C	-2.144034	0.198106	-2.238988
C	0.459254	-0.634633	-2.726471
H	-0.229570	-1.161255	4.692762
H	-2.559952	-0.403737	4.250837
H	-3.163156	0.511581	2.036187
C	-1.801783	-0.320110	-3.480985
C	-0.498760	-0.745325	-3.729409
H	-2.559952	-0.403737	-4.250837
H	-0.229570	-1.161255	-4.692762
H	-2.531910	1.173838	-0.000000
H	-3.163156	0.511581	-2.036187
H	1.475053	-0.966871	-2.910967
H	1.475053	-0.966871	2.910967

Solvent = Pentyl Ethanoate
E = -785.318242
G = -785.178701
ZPC = 0.177609
TCE = 0.188482
TCH = 0.189427
TCG = 0.139541
N_{imag} = 0

Te	1.584106	0.223361	0.000000
N	-1.557673	0.878059	-0.000000
C	-1.181662	0.318247	-1.236913
C	0.133187	-0.087292	-1.493669
C	-0.488922	-0.733109	3.741032
C	-1.791887	-0.309157	3.494963
C	-2.139968	0.195771	2.249387
C	-1.181662	0.318247	1.236913
C	0.133187	-0.087292	1.493669
C	0.464295	-0.631934	2.732727
C	-2.139968	0.195771	-2.249387
C	0.464295	-0.631934	-2.732727
H	-0.215574	-1.140861	4.706809
H	-2.546512	-0.385358	4.269273
H	-3.161482	0.504039	2.049340
C	-1.791887	-0.309157	-3.494963
C	-0.488922	-0.733109	-3.741032
H	-2.546512	-0.385358	-4.269273
H	-0.215574	-1.140861	-4.706809
H	-2.523673	1.182590	-0.000000
H	-3.161482	0.504039	-2.049340
H	1.480236	-0.964870	-2.916353
H	1.480236	-0.964870	2.916353

HOH

Solvent = Water
E = -76.434803
G = -76.431136
ZPC = 0.021315
TCE = 0.024151
TCH = 0.025095
TCG = 0.003667
N_{imag} = 0

O	0.000000	0.000000	0.118132
H	-0.000000	0.760469	-0.472529
H	-0.000000	-0.760469	-0.472529

Solvent = Pentyl Ethanoate
E = -76.427362
G = -76.423606
ZPC = 0.021398
TCE = 0.024234
TCH = 0.025178
TCG = 0.003757
N_{imag} = 0

O	0.000000	0.000000	0.117361
H	-0.000000	0.761420	-0.469446
H	-0.000000	-0.761420	-0.469446

HO$^{\bullet}$

Solvent = Water
E = -75.736005
G = -75.744389
ZPC = 0.008542
TCE = 0.010902
TCH = 0.011846
TCG = -0.008384

N_{imag} = 0

```
O    -0.000000   0.000000   0.108299
H     0.000000  -0.000000  -0.866392
```

Solvent = Pentyl Ethanoate
E = -75.731843
G = -75.740244
ZPC = 0.008525
TCE = 0.010885
TCH = 0.011829
TCG = -0.008402
N_{imag} = 0

```
O    -0.000000   0.000000   0.108307
H     0.000000  -0.000000  -0.866453
```

HOOH
Solvent = Water
E = -151.551627
G = -151.546233
ZPC = 0.027019
TCE = 0.030254
TCH = 0.031199
TCG = 0.005394
N_{imag} = 0

```
O    -0.000000   0.710158  -0.069636
O    -0.000000  -0.710158  -0.069636
H    -0.705240  -0.927046   0.557091
H     0.705240   0.927046   0.557091
```

Solvent = Pentyl Ethanoate
E = -151.545562
G = -151.540179
ZPC = 0.026987
TCE = 0.030199
TCH = 0.031144
TCG = 0.005382
N_{imag} = 0

```
O     0.000000   0.711207  -0.059819
O    -0.000000  -0.711207  -0.059819
H    -0.777969  -0.911260   0.478556
H     0.777969   0.911260   0.478556
```

HOO•
Solvent = Water
E = -150.903342
G = -150.910824
ZPC = 0.014651
TCE = 0.017501
TCH = 0.018445
TCG = -0.007481
N_{imag} = 0

```
O     0.054930   0.705918   0.000000
O     0.054930  -0.595337   0.000000
H    -0.878876  -0.884648  -0.000000
```

Solvent = Pentyl Ethanoate
E = -150.901031
G = -150.908675
ZPC = 0.014497
TCE = 0.017348
TCH = 0.018292
TCG = -0.007645
N_{imag} = 0

```
O     0.055100   0.707511   0.000000
O     0.055100  -0.597882  -0.000000
H    -0.881608  -0.877031  -0.000000
```

CH3OOH
Solvent = Water
E = -190.840800
G = -190.810545
ZPC = 0.055552
TCE = 0.059771
TCH = 0.060715
TCG = 0.030255
N_{imag} = 0

```
C     1.122649   0.228573  -0.001096
H     1.967550  -0.454559   0.092101
H     1.180051   0.770483  -0.946912
H     1.109202   0.924308   0.841126
O    -0.027636  -0.605342   0.025107
O    -1.169578   0.225830  -0.121935
H    -1.414988   0.424426   0.794881
```

Solvent = Pentyl Ethanoate
E = -190.838019
G = -190.808235
ZPC = 0.055327
TCE = 0.059667
TCH = 0.060611
TCG = 0.029784
N_{imag} = 0

```
C     1.122876   0.222981   0.016964
H     1.966218  -0.469047   0.046045
H     1.179368   0.837927  -0.884290
H     1.131684   0.856922   0.908284
O    -0.027492  -0.601563  -0.009577
O    -1.156007   0.258482  -0.108556
H    -1.546528   0.180964   0.773246
```

CH3OO•
Solvent = Water
E = -190.195356
G = -190.177310
ZPC = 0.043689
TCE = 0.047499
TCH = 0.048443
TCG = 0.018046
N_{imag} = 0

```
C    -1.094976  -0.178863   0.000001
H    -1.860234   0.593352   0.000278
H    -1.144954  -0.789141  -0.900378
H    -1.144722  -0.789568   0.900101
O     0.163313   0.530531   0.000000
O     1.176658  -0.273214  -0.000001
```

Solvent = Pentyl Ethanoate

E = -190.197481
G = -190.179419
ZPC = 0.043707
TCE = 0.047516
TCH = 0.048460
TCG = 0.018063
N_{imag} = 0

C	-1.091016	-0.178297	0.000002
H	-1.863409	0.588152	0.000362
H	-1.148552	-0.792528	-0.898305
H	-1.148237	-0.793082	0.897945
O	0.162405	0.533179	-0.000000
O	1.175882	-0.274774	-0.000001

Hydrogen Atom Transfer

PS(-H)• - Site 1
Solvent = Water
E = -914.930095
G = -914.801040
ZPC = 0.165834
TCE = 0.175921
TCH = 0.176865
TCG = 0.129055
N_{imag} = 0

H	-2.233965	2.663296	-0.000012
H	-4.495118	1.655556	0.000020
H	-4.744119	-0.823933	0.000060
H	-2.744055	-2.262694	0.000045
N	0.000000	1.457134	-0.000030
C	-3.616133	1.022365	0.000017
C	-2.362613	1.587177	-0.000004
C	-1.185382	0.793303	-0.000017
C	-1.358372	-0.615490	-0.000002
C	-2.636949	-1.183229	0.000027
C	2.362613	1.587177	-0.000005
C	2.636949	-1.183229	0.000027
C	1.185382	0.793303	-0.000017
C	1.358372	-0.615490	-0.000002
C	3.616133	1.022365	0.000017
C	3.758648	-0.374757	0.000035
C	-3.758648	-0.374757	0.000035
H	4.495118	1.655556	0.000019
H	4.744119	-0.823933	0.000060
H	2.233965	2.663296	-0.000012
H	2.744055	-2.262694	0.000045
S	0.000000	-1.713551	-0.000043

Solvent = Pentyl Ethanoate
E = -914.936085
G = -914.807154
ZPC = 0.165702
TCE = 0.175796
TCH = 0.176740
TCG = 0.128931
N_{imag} = 0

H	-2.219098	2.662886	-0.000050
H	-4.492372	1.661532	-0.000030
H	-4.746334	-0.816761	0.000063
H	-2.749013	-2.261229	0.000086
N	0.000000	1.451561	0.000015
C	-3.614298	1.026618	-0.000011
C	-2.359613	1.588159	-0.000018
C	-1.184257	0.790376	0.000005
C	-1.359695	-0.618823	0.000021
C	-2.639874	-1.181783	0.000049
C	2.359613	1.588159	-0.000019
C	2.639874	-1.181783	0.000049
C	1.184257	0.790376	0.000005
C	1.359695	-0.618823	0.000021
C	3.614298	1.026618	-0.000011
C	3.759514	-0.370013	0.000040
C	-3.759514	-0.370013	0.000040
H	4.492372	1.661532	-0.000030
H	4.746334	-0.816761	0.000063
H	2.219098	2.662886	-0.000050
H	2.749013	-2.261229	0.000086
S	0.000000	-1.716762	-0.000079

PS(-H)• - Site 2
Solvent = Water
E = -914.879075
G = -914.749311
ZPC = 0.166439
TCE = 0.176598
TCH = 0.177542
TCG = 0.129764
N_{imag} = 0

H	0.017239	2.320463	0.768805
H	-4.357854	1.790771	-0.591314
H	-4.617057	-0.677595	-0.927872
H	-2.694958	-2.179932	-0.495418
N	0.006900	1.306501	0.756806
C	-3.533856	1.112479	-0.401384
C	-2.309578	1.562255	0.017830
C	-1.208907	0.780459	0.296216
C	-1.379719	-0.599614	0.110695
C	-2.593186	-1.111403	-0.345348
C	2.313814	1.558220	0.023094
C	2.506938	-1.192490	-0.355616
C	1.210283	0.747065	0.290429
C	1.318374	-0.635295	0.106425
C	3.510162	0.994268	-0.403961
C	3.610576	-0.380649	-0.599501
C	-3.673291	-0.267725	-0.589130
H	4.362053	1.635612	-0.596042
H	4.538305	-0.821326	-0.942812
H	2.223166	2.630553	0.161040
H	2.570087	-2.263970	-0.508577
S	-0.044934	-1.686594	0.558504

Solvent = Pentyl Ethanoate
E = -914.886003
G = -914.756248
ZPC = 0.166450
TCE = 0.176624
TCH = 0.177568
TCG = 0.129755
N_{imag} = 0

H	0.014861	2.323940	0.769106
H	-4.373897	1.784298	-0.568045

H	-4.629674	-0.685445	-0.900723
H	-2.698220	-2.181504	-0.486410
N	0.006642	1.312469	0.722353
C	-3.546260	1.107872	-0.387030
C	-2.318611	1.561854	0.017079
C	-1.212882	0.783228	0.284506
C	-1.381941	-0.598415	0.104971
C	-2.598507	-1.112327	-0.338665
C	2.323288	1.556942	0.023055
C	2.511330	-1.192835	-0.349677
C	1.213646	0.750532	0.279081
C	1.319959	-0.633399	0.100102
C	3.522917	0.988945	-0.388674
C	3.620468	-0.385471	-0.582811
C	-3.683479	-0.272218	-0.572396
H	4.379235	1.627519	-0.571021
H	4.550467	-0.829670	-0.915700
H	2.237379	2.630388	0.156905
H	2.571971	-2.264829	-0.500953
S	-0.044887	-1.682514	0.551446

PS(-H)• - Site 3
Solvent = Water
E = -914.880622
G = -914.751008
ZPC = 0.166249
TCE = 0.176399
TCH = 0.177344
TCG = 0.129615
N_{imag} = 0

H	0.004393	2.313876	0.747544
H	-2.194137	2.719042	0.103284
H	-4.644795	-0.679486	-0.962575
H	-2.710014	-2.188826	-0.504127
N	-0.019053	1.300224	0.727143
C	-3.482526	1.064871	-0.427363
C	-2.312535	1.646311	-0.012552
C	-1.239910	0.788096	0.269611
C	-1.407142	-0.593306	0.097736
C	-2.610709	-1.117885	-0.364557
C	2.294050	1.533826	0.006018
C	2.477732	-1.223532	-0.331663
C	1.185703	0.730825	0.277814
C	1.288937	-0.654248	0.114463
C	3.490806	0.958360	-0.404914
C	3.586446	-0.419698	-0.579939
C	-3.697750	-0.276943	-0.624837
H	4.346857	1.593082	-0.600506
H	4.514666	-0.869095	-0.910470
H	2.207481	2.608442	0.128021
H	2.537064	-2.297493	-0.468173
S	-0.085422	-1.682573	0.578631

Solvent = Pentyl Ethanoate
E = -914.887001
G = -914.757439
ZPC = 0.166226
TCE = 0.176397
TCH = 0.177341
TCG = 0.129562
N_{imag} = 0

H	0.004137	2.315504	0.752811
H	-2.209867	2.717946	0.100812
H	-4.656316	-0.686381	-0.938414
H	-2.710871	-2.190121	-0.497869
N	-0.019062	1.304843	0.696701
C	-3.495762	1.060396	-0.413522
C	-2.322616	1.644159	-0.011151
C	-1.243872	0.790325	0.260591
C	-1.408708	-0.592327	0.092737
C	-2.614756	-1.118620	-0.359182
C	2.302846	1.532849	0.006444
C	2.481667	-1.223165	-0.327661
C	1.189270	0.733769	0.268038
C	1.290717	-0.652463	0.108531
C	3.502276	0.954260	-0.391560
C	3.595071	-0.423100	-0.566307
C	-3.707104	-0.281526	-0.609470
H	4.362244	1.586592	-0.578379
H	4.525035	-0.875316	-0.888396
H	2.221185	2.608423	0.125341
H	2.538618	-2.297416	-0.463577
S	-0.084682	-1.678779	0.572865

PS(-H)• - Site 4
Solvent = Water
E = -914.879697
G = -914.749889
ZPC = 0.166431
TCE = 0.176561
TCH = 0.177505
TCG = 0.129807
N_{imag} = 0

H	-0.082111	2.262871	0.790462
H	-2.260754	2.606038	0.139608
H	-4.421031	1.634979	-0.645522
H	-2.657208	-2.302683	-0.563026
N	-0.069861	1.249415	0.757490
C	-3.570397	0.993511	-0.447180
C	-2.362292	1.535170	-0.004569
C	-1.273130	0.705575	0.272151
C	-1.385777	-0.678084	0.084341
C	-2.574770	-1.233985	-0.397652
C	2.233113	1.581581	0.039797
C	2.515363	-1.160329	-0.351715
C	1.153472	0.735128	0.295885
C	1.307827	-0.641862	0.105889
C	3.449373	1.057304	-0.381886
C	3.594515	-0.312793	-0.584175
C	-3.614629	-0.367641	-0.627189
H	4.282058	1.726069	-0.564650
H	4.538083	-0.721851	-0.923450
H	2.108834	2.649928	0.182629
H	2.612763	-2.228562	-0.509145
S	-0.029226	-1.728384	0.548031

Solvent = Pentyl Ethanoate
E = -914.886023
G = -914.756374
ZPC = 0.166312
TCE = 0.176476
TCH = 0.177420
TCG = 0.129648

N_{imag} = 0

H	-0.081420	2.267479	0.787202
H	-2.275842	2.605746	0.134577
H	-4.440887	1.624328	-0.618783
H	-2.660119	-2.303877	-0.552570
N	-0.069124	1.257218	0.722007
C	-3.584843	0.986913	-0.430435
C	-2.372943	1.533572	-0.004774
C	-1.276940	0.709422	0.260708
C	-1.387818	-0.675856	0.079160
C	-2.580012	-1.234602	-0.389533
C	2.243927	1.579941	0.038899
C	2.519760	-1.161157	-0.345716
C	1.157623	0.738775	0.283679
C	1.309667	-0.639857	0.099563
C	3.463289	1.051230	-0.367200
C	3.604834	-0.318559	-0.567765
C	-3.626593	-0.373742	-0.609057
H	4.300740	1.716881	-0.540277
H	4.550453	-0.731601	-0.896716
H	2.124455	2.649578	0.177788
H	2.614229	-2.230067	-0.501136
S	-0.029215	-1.723468	0.541918

PS(-H)$^{\bullet}$ - Site 5
Solvent = Water
E = -914.880267
G = -914.750485
ZPC = 0.166473
TCE = 0.176631
TCH = 0.177575
TCG = 0.129782
N_{imag} = 0

H	-0.061012	2.264838	0.765440
H	-2.282869	2.569849	0.167941
H	-4.418210	1.577705	-0.588279
H	-4.600292	-0.891688	-0.956797
N	-0.047546	1.250969	0.740096
C	-3.563811	0.936501	-0.404381
C	-2.366094	1.497972	0.024739
C	-1.250807	0.695922	0.285351
C	-1.350779	-0.692091	0.098044
C	-2.546463	-1.179999	-0.358431
C	2.254536	1.577068	0.030635
C	2.545530	-1.162696	-0.356733
C	1.176270	0.728623	0.286180
C	1.334844	-0.648805	0.098388
C	3.473195	1.056799	-0.388197
C	3.622770	-0.312952	-0.588370
C	-3.675632	-0.443414	-0.614548
H	4.303758	1.728304	-0.570308
H	4.567862	-0.720027	-0.925702
H	2.126605	2.645282	0.171297
H	2.646261	-2.230743	-0.513510
S	0.008085	-1.751117	0.537074

Solvent = Pentyl Ethanoate
E = -914.886632
G = -914.756952
ZPC = 0.166403
TCE = 0.176586
TCH = 0.177530
TCG = 0.129680
N_{imag} = 0

H	-0.060439	2.269573	0.759340
H	-2.296173	2.570620	0.160750
H	-4.435952	1.570834	-0.561948
H	-4.614421	-0.900959	-0.922269
N	-0.047248	1.258623	0.702826
C	-3.577505	0.931889	-0.388064
C	-2.375385	1.497495	0.023008
C	-1.254421	0.699669	0.271958
C	-1.353370	-0.689986	0.091438
C	-2.553114	-1.181003	-0.348857
C	2.265377	1.575371	0.030531
C	2.550552	-1.163483	-0.349088
C	1.180396	0.732018	0.273561
C	1.336460	-0.647081	0.091891
C	3.487788	1.050879	-0.371086
C	3.634029	-0.318620	-0.568890
C	-3.687328	-0.447787	-0.593160
H	4.323231	1.719364	-0.542688
H	4.581592	-0.729727	-0.894601
H	2.141799	2.644885	0.166948
H	2.648616	-2.232185	-0.503961
S	0.007601	-1.747433	0.527450

PSE(-H)$^{\bullet}$ - Site 1
Solvent = Water
E = -2918.363956
G = -2918.236970
ZPC = 0.164791
TCE = 0.175243
TCH = 0.176187
TCG = 0.126986
N_{imag} = 0

H	2.956683	-0.000004	2.184583
H	2.031830	0.000012	4.477965
H	-0.438993	0.000018	4.815706
H	-1.944843	0.000010	2.861944
N	1.694960	-0.000016	0.000000
C	1.366745	0.000008	3.622657
C	1.885406	-0.000001	2.349471
C	1.051537	-0.000007	1.196704
C	-0.350631	-0.000001	1.420928
C	-0.869501	0.000007	2.720088
C	1.885406	-0.000001	-2.349471
C	-0.869501	0.000007	-2.720088
C	1.051537	-0.000007	-1.196704
C	-0.350631	-0.000001	-1.420928
C	1.366745	0.000008	-3.622657
C	-0.024462	0.000011	-3.814968
C	-0.024462	0.000011	3.814968
H	2.031830	0.000012	-4.477965
H	-0.438993	0.000018	-4.815706
H	2.956683	-0.000004	-2.184583
H	-1.944843	0.000010	-2.861944
Se	-1.581859	-0.000005	-0.000000

Solvent = Pentyl Ethanoate
E = -2918.367528
G = -2918.240692

ZPC = 0.164682
TCE = 0.175142
TCH = 0.176086
TCG = 0.126835
N_{imag} = 0

H	2.956041	-0.000003	2.168963
H	2.038549	0.000012	4.473568
H	-0.430540	0.000014	4.817552
H	-1.943075	0.000007	2.871389
N	1.688557	-0.000015	0.000000
C	1.371363	0.000007	3.619642
C	1.886499	-0.000001	2.345219
C	1.048279	-0.000006	1.195680
C	-0.353623	-0.000001	1.423686
C	-0.868355	0.000005	2.723292
C	1.886499	-0.000001	-2.345219
C	-0.868355	0.000005	-2.723292
C	1.048279	-0.000006	-1.195680
C	-0.353623	-0.000001	-1.423686
C	1.371363	0.000007	-3.619642
C	-0.018918	0.000009	-3.815366
C	-0.018918	0.000009	3.815366
H	2.038549	0.000012	-4.473568
H	-0.430540	0.000014	-4.817552
H	2.956041	-0.000003	-2.168963
H	-1.943075	0.000007	-2.871389
Se	-1.583670	-0.000004	-0.000000

PSE(-H)$^{\bullet}$ - Site 2
Solvent = Water
E = -2918.314758
G = -2918.186868
ZPC = 0.165631
TCE = 0.176169
TCH = 0.177113
TCG = 0.127890
N_{imag} = 0

H	-0.028523	2.453972	-0.993906
H	4.306209	2.242085	0.468565
H	4.669646	-0.165899	1.061903
H	2.813983	-1.784896	0.782447
N	-0.017210	1.450922	-0.843054
C	3.513744	1.511956	0.349279
C	2.274432	1.860539	-0.117681
C	1.207271	1.008472	-0.315866
C	1.436925	-0.334852	0.012847
C	2.668782	-0.742729	0.522766
C	-2.303617	1.844228	-0.126170
C	-2.593405	-0.840014	0.539499
C	-1.228560	0.970791	-0.307796
C	-1.386591	-0.379088	0.020108
C	-3.517545	1.373407	0.358523
C	-3.666708	0.030962	0.698277
C	3.711227	0.165810	0.680882
H	-4.344385	2.062766	0.481857
H	-4.608478	-0.337092	1.086390
H	-2.177453	2.892827	-0.374935
H	-2.693891	-1.886743	0.803478
Se	0.043754	-1.601481	-0.332425

Solvent = Pentyl Ethanoate
E = -2918.319551
G = -2918.191801
ZPC = 0.165530
TCE = 0.176103
TCH = 0.177047
TCG = 0.127751
N_{imag} = 0

H	-0.026477	2.462405	-0.975658
H	4.328039	2.227564	0.445779
H	4.687444	-0.184851	1.025294
H	2.819688	-1.792528	0.764800
N	-0.016860	1.465001	-0.799180
C	3.530666	1.501360	0.334786
C	2.286841	1.858745	-0.112469
C	1.212216	1.013214	-0.299617
C	1.440678	-0.333586	0.017564
C	2.676471	-0.748620	0.510207
C	-2.316079	1.841721	-0.121816
C	-2.600074	-0.844911	0.527665
C	-1.232848	0.975957	-0.292300
C	-1.389719	-0.377071	0.025147
C	-3.534319	1.362531	0.342625
C	-3.680494	0.018931	0.675573
C	3.725792	0.153542	0.658098
H	-4.366777	2.046978	0.456659
H	-4.625283	-0.355713	1.050095
H	-2.193650	2.892919	-0.362349
H	-2.698270	-1.893138	0.787445
Se	0.043486	-1.593809	-0.329135

PSE(-H)$^{\bullet}$ - Site 3
Solvent = Water
E = -2918.316037
G = -2918.188265
ZPC = 0.165452
TCE = 0.175976
TCH = 0.176920
TCG = 0.127773
N_{imag} = 0

H	-0.036052	2.448192	-0.974264
H	2.090974	3.008546	-0.319774
H	4.688382	-0.137087	1.107909
H	2.830602	-1.775731	0.801089
N	-0.004340	1.446862	-0.814746
C	3.449723	1.484238	0.386462
C	2.257738	1.960178	-0.092875
C	1.226613	1.026824	-0.288230
C	1.458632	-0.318016	0.029091
C	2.682130	-0.731132	0.549821
C	-2.299230	1.809343	-0.107454
C	-2.564922	-0.886070	0.524705
C	-1.214696	0.948663	-0.294179
C	-1.361082	-0.406359	0.016056
C	-3.510438	1.320320	0.366349
C	-3.647458	-0.027513	0.689543
C	3.726481	0.182279	0.725851
H	-4.344827	1.999674	0.494293
H	-4.587105	-0.409317	1.069489
H	-2.182841	2.862083	-0.343373
H	-2.655955	-1.936956	0.775184
Se	0.088419	-1.598940	-0.351123

Solvent = Pentyl Ethanoate
E = -2918.320282
G = -2918.192692
ZPC = 0.165315
TCE = 0.175882
TCH = 0.176827
TCG = 0.127589
N_{imag} = 0

H	-0.035235	2.454268	-0.961180
H	2.113693	3.007225	-0.309249
H	4.706630	-0.157270	1.070903
H	2.833572	-1.783674	0.786174
N	-0.004138	1.459447	-0.773129
C	3.469608	1.472351	0.369674
C	2.273212	1.955979	-0.090348
C	1.232283	1.030770	-0.274021
C	1.461572	-0.317205	0.033942
C	2.688798	-0.737493	0.538254
C	-2.310556	1.807714	-0.102940
C	-2.572062	-0.889412	0.513524
C	-1.218974	0.953727	-0.278964
C	-1.364577	-0.404010	0.021351
C	-3.526181	1.311598	0.351022
C	-3.660949	-0.037144	0.667480
C	3.741551	0.168365	0.702271
H	-4.365557	1.986716	0.469794
H	-4.603690	-0.424734	1.033958
H	-2.197676	2.862895	-0.330758
H	-2.661399	-1.941597	0.760056
Se	0.086949	-1.591511	-0.347508

PSE(-H)˙ - Site 4
Solvent = Water
E = -2918.315144
G = -2918.187226
ZPC = 0.165595
TCE = 0.176106
TCH = 0.177050
TCG = 0.127917
N_{imag} = 0

H	0.069114	2.405154	-1.002158
H	2.181163	2.894428	-0.333933
H	4.372748	2.103340	0.548993
H	2.781006	-1.897194	0.853208
N	0.060466	1.404583	-0.835412
C	3.552939	1.407701	0.412967
C	2.326140	1.847930	-0.085286
C	1.273681	0.948278	-0.282103
C	1.446446	-0.402517	0.043084
C	2.655214	-0.854575	0.582778
C	-2.219886	1.869610	-0.136201
C	-2.594356	-0.805235	0.527347
C	-1.169108	0.965068	-0.309876
C	-1.371477	-0.379100	0.016491
C	-3.450966	1.434619	0.338903
C	-3.642596	0.097307	0.678086
C	3.655182	0.074145	0.728537
H	-4.258207	2.147987	0.455792
H	-4.597789	-0.242529	1.059109
H	-2.061264	2.914028	-0.383849
H	-2.727398	-1.848566	0.790322
Se	0.030885	-1.633180	-0.330234

Solvent = Pentyl Ethanoate
E = -2918.319325
G = -2918.191657
ZPC = 0.165407
TCE = 0.175973
TCH = 0.176917
TCG = 0.127667
N_{imag} = 0

H	0.068535	2.413108	-0.985110
H	2.199076	2.894107	-0.321053
H	4.398822	2.084474	0.521394
H	2.787068	-1.904714	0.834183
N	0.059806	1.418779	-0.792096
C	3.572205	1.394933	0.395076
C	2.340242	1.844760	-0.081232
C	1.278713	0.953243	-0.266830
C	1.450142	-0.400832	0.047151
C	2.663157	-0.860496	0.568601
C	-2.233622	1.867230	-0.130517
C	-2.601552	-0.810581	0.515572
C	-1.174294	0.970388	-0.293363
C	-1.375032	-0.377100	0.022040
C	-3.469018	1.423475	0.324035
C	-3.657272	0.084736	0.655635
C	3.671775	0.060419	0.704052
H	-4.282157	2.131816	0.431807
H	-4.615565	-0.262050	1.022630
H	-2.079544	2.914403	-0.370095
H	-2.732216	-1.855657	0.773600
Se	0.030726	-1.624941	-0.327177

PSE(-H)˙ - Site 5
Solvent = Water
E = -2918.316351
G = -2918.188511
ZPC = 0.165623
TCE = 0.176173
TCH = 0.177117
TCG = 0.127840
N_{imag} = 0

H	0.067044	2.414330	-0.977380
H	2.230102	2.845822	-0.366948
H	4.393105	2.016540	0.487718
H	4.664070	-0.395282	1.107481
N	0.051440	1.412434	-0.818330
C	3.564495	1.327709	0.368922
C	2.349301	1.797046	-0.117019
C	1.262641	0.933368	-0.296996
C	1.415048	-0.423420	0.029478
C	2.624044	-0.816407	0.534554
C	-2.225977	1.881060	-0.128387
C	-2.622592	-0.789817	0.529911
C	-1.180833	0.969591	-0.300636
C	-1.393995	-0.374053	0.023600
C	-3.461872	1.455771	0.342101
C	-3.664782	0.119633	0.678548
C	3.725841	-0.018708	0.718906
H	-4.263529	2.175599	0.457460

H	-4.623530	-0.214042	1.055987
H	-2.058358	2.924636	-0.373581
H	-2.764375	-1.832358	0.791515
Se	-0.010368	-1.652733	-0.316230

Solvent = Pentyl Ethanoate
E = -2918.320623
G = -2918.193008
ZPC = 0.165454
TCE = 0.176052
TCH = 0.176996
TCG = 0.127615
N_{imag} = 0

H	0.065919	2.426191	-0.950575
H	2.245537	2.847926	-0.348349
H	4.417250	2.001244	0.459497
H	4.685116	-0.417103	1.057754
N	0.050874	1.429748	-0.768646
C	3.583156	1.317052	0.350724
C	2.361850	1.795946	-0.109681
C	1.267145	0.939867	-0.277819
C	1.419396	-0.420775	0.034275
C	2.634114	-0.822749	0.516203
C	-2.241629	1.878038	-0.122451
C	-2.630603	-0.796781	0.513540
C	-1.186673	0.975459	-0.282357
C	-1.397299	-0.372297	0.028170
C	-3.482653	1.442454	0.323777
C	-3.681583	0.104200	0.650282
C	3.742865	-0.031738	0.687893
H	-4.291120	2.156443	0.428991
H	-4.643863	-0.237856	1.011162
H	-2.078974	2.924843	-0.357907
H	-2.769570	-1.841454	0.769054
Se	-0.009263	-1.644721	-0.310719

PTE(-H)• - Site 1
Solvent = Water
E = -784.672949
G = -784.547983
ZPC = 0.163727
TCE = 0.174487
TCH = 0.175431
TCG = 0.124965
N_{imag} = 0

H	3.236884	0.000034	2.115787
H	2.424498	0.000243	4.448517
H	-0.029270	0.000208	4.904343
H	-1.622237	0.000059	3.027735
N	1.902319	-0.000096	-0.000000
C	1.718344	0.000130	3.626578
C	2.174268	0.000008	2.329812
C	1.287851	-0.000201	1.213050
C	-0.104711	-0.000115	1.503198
C	-0.555173	0.000027	2.828583
C	2.174268	0.000008	-2.329812
C	-0.555173	0.000027	-2.828583
C	1.287851	-0.000201	-1.213050
C	-0.104711	-0.000115	-1.503198
C	1.718344	0.000130	-3.626578
C	0.338314	0.000111	-3.885271
C	0.338314	0.000111	3.885271
H	2.424498	0.000243	-4.448517
H	-0.029270	0.000208	-4.904343
H	3.236884	0.000034	-2.115787
H	-1.622237	0.000059	-3.027735
Te	-1.531590	0.000001	0.000000

Solvent = Pentyl Ethanoate
E = -784.675611
G = -784.550593
ZPC = 0.163732
TCE = 0.174488
TCH = 0.175432
TCG = 0.125017
N_{imag} = 0

H	3.234942	0.000049	2.096856
H	2.434070	0.000157	4.442278
H	-0.017018	0.000106	4.907550
H	-1.619061	0.000017	3.040704
N	1.893211	-0.000034	-0.000000
C	1.724689	0.000082	3.622912
C	2.175041	0.000014	2.324313
C	1.282844	-0.000139	1.212478
C	-0.108618	-0.000087	1.508287
C	-0.553077	-0.000001	2.834224
C	2.175041	0.000014	-2.324313
C	-0.553077	-0.000001	-2.834224
C	1.282844	-0.000139	-1.212478
C	-0.108618	-0.000087	-1.508287
C	1.724689	0.000082	-3.622912
C	0.346336	0.000053	-3.886726
C	0.346336	0.000053	3.886726
H	2.434070	0.000157	-4.442278
H	-0.017018	0.000106	-4.907550
H	3.234942	0.000049	-2.096856
H	-1.619061	0.000017	-3.040704
Te	-1.533172	0.000010	0.000000

PTE(-H)• - Site 2
Solvent = Water
E = -784.627464
G = -784.501396
ZPC = 0.164757
TCE = 0.175593
TCH = 0.176537
TCG = 0.126068
N_{imag} = 0

H	-0.042974	2.562215	-1.184891
H	4.217185	2.700025	0.371285
H	4.717302	0.365513	1.139351
H	2.970195	-1.375906	0.957130
N	-0.029396	1.580911	-0.927644
C	3.472323	1.915608	0.297341
C	2.220650	2.152039	-0.203866
C	1.206047	1.226241	-0.349565
C	1.510140	-0.076825	0.071770
C	2.760525	-0.366652	0.621271
C	-2.279398	2.120022	-0.211181
C	-2.699042	-0.489824	0.638587
C	-1.250875	1.180826	-0.339937
C	-1.472024	-0.135960	0.078547

C	-3.510770	1.750031	0.313248
C	-3.725005	0.443738	0.746617
C	3.745966	0.610701	0.726260
H	-4.298903	2.489304	0.395225
H	-4.679926	0.153046	1.167447
H	-2.102330	3.141694	-0.531423
H	-2.857354	-1.507583	0.978229
Te	0.046372	-1.568737	-0.214104

Solvent = Pentyl Ethanoate
E = -784.631490
G = -784.505515
ZPC = 0.164695
TCE = 0.175561
TCH = 0.176505
TCG = 0.125975
N_{imag} = 0

H	-0.041472	2.561215	-1.180201
H	4.238928	2.683502	0.350809
H	4.733561	0.346393	1.115654
H	2.975263	-1.383969	0.952421
N	-0.029069	1.590366	-0.890339
C	3.489001	1.903064	0.285453
C	2.233458	2.148036	-0.201028
C	1.211246	1.228443	-0.335471
C	1.514613	-0.077222	0.079154
C	2.767531	-0.373952	0.617033
C	-2.291588	2.115459	-0.208146
C	-2.706002	-0.495986	0.634353
C	-1.255344	1.183084	-0.326313
C	-1.476293	-0.136033	0.086068
C	-3.526807	1.738030	0.300607
C	-3.738255	0.431372	0.731962
C	3.759578	0.597401	0.712051
H	-4.320224	2.472789	0.373736
H	-4.695926	0.135354	1.142926
H	-2.118055	3.139670	-0.522646
H	-2.863040	-1.514294	0.973675
Te	0.046300	-1.560450	-0.215930

PTE(-H)$^{\bullet}$ - Site 3
Solvent = Water
E = -784.628401
G = -784.502151
ZPC = 0.164778
TCE = 0.175559
TCH = 0.176504
TCG = 0.126250
N_{imag} = 0

H	-0.079999	2.562861	-1.152114
H	1.948296	3.289247	-0.484654
H	4.729714	0.430284	1.175359
H	2.991192	-1.340841	0.974754
N	-0.035318	1.584222	-0.889123
C	3.395673	1.912249	0.332869
C	2.179722	2.271891	-0.183703
C	1.209824	1.261166	-0.316438
C	1.524214	-0.042357	0.092832
C	2.770675	-0.330111	0.647759
C	-2.301219	2.067696	-0.185266
C	-2.678413	-0.562694	0.620524
C	-1.253456	1.150439	-0.318701
C	-1.453488	-0.176113	0.078006
C	-3.530078	1.666033	0.321786
C	-3.723212	0.349309	0.733016
C	3.753030	0.658526	0.766039
H	-4.333026	2.388780	0.407303
H	-4.676440	0.033853	1.139631
H	-2.141767	3.097473	-0.488423
H	-2.819711	-1.588646	0.942533
Te	0.101333	-1.563822	-0.227363

Solvent = Pentyl Ethanoate
E = -784.631937
G = -784.505759
ZPC = 0.164709
TCE = 0.175511
TCH = 0.176455
TCG = 0.126178
N_{imag} = 0

H	-0.076899	2.558270	-1.153607
H	1.963725	3.287583	-0.470987
H	4.746718	0.412687	1.148737
H	2.997403	-1.349802	0.966370
N	-0.034429	1.590810	-0.856394
C	3.412005	1.901294	0.322588
C	2.191625	2.266834	-0.178884
C	1.215258	1.261523	-0.304577
C	1.528930	-0.044476	0.097761
C	2.778434	-0.338063	0.640657
C	-2.311569	2.063966	-0.185307
C	-2.684235	-0.565922	0.618744
C	-1.257414	1.152424	-0.307523
C	-1.457304	-0.175507	0.085568
C	-3.543574	1.657146	0.308837
C	-3.734107	0.341108	0.720876
C	3.766768	0.645374	0.749781
H	-4.350918	2.376212	0.385421
H	-4.689383	0.022100	1.120066
H	-2.155289	3.095316	-0.485410
H	-2.824467	-1.591705	0.942533
Te	0.100023	-1.556549	-0.228259

PTE(-H)$^{\bullet}$ - Site 4
Solvent = Water
E = -784.627511
G = -784.501274
ZPC = 0.164801
TCE = 0.175593
TCH = 0.176537
TCG = 0.126236
N_{imag} = 0

H	0.049554	2.517814	-1.188885
H	2.061048	3.173091	-0.483999
H	4.283781	2.584718	0.470781
H	2.946150	-1.476692	1.032820
N	0.047426	1.541171	-0.915329
C	3.511250	1.830658	0.373947
C	2.266709	2.156117	-0.164590
C	1.273137	1.179771	-0.309258
C	1.523184	-0.134806	0.105814
C	2.753318	-0.466333	0.688550

C	-2.194932	2.143778	-0.216980
C	-2.692141	-0.455891	0.623976
C	-1.190109	1.177690	-0.339145
C	-1.452108	-0.132957	0.074461
C	-3.440250	1.805659	0.296219
C	-3.693387	0.504904	0.725573
C	3.691769	0.530591	0.781555
H	-4.209126	2.565527	0.373057
H	-4.659403	0.239489	1.137721
H	-1.988528	3.161244	-0.533481
H	-2.880256	-1.469969	0.959308
Te	0.037042	-1.594587	-0.215399

Solvent = Pentyl Ethanoate
E = -784.630954
G = -784.504853
ZPC = 0.164696
TCE = 0.175518
TCH = 0.176463
TCG = 0.126101
N_{imag} = 0

H	0.049611	2.504041	-1.206900
H	2.070525	3.171086	-0.478509
H	4.299420	2.573001	0.455900
H	2.952660	-1.479975	1.033510
N	0.046933	1.541905	-0.890393
C	3.522752	1.822344	0.364811
C	2.274965	2.152260	-0.163251
C	1.277122	1.179320	-0.301312
C	1.527816	-0.136291	0.111228
C	2.759534	-0.470325	0.686978
C	-2.203636	2.139756	-0.216608
C	-2.696987	-0.459162	0.625346
C	-1.194231	1.177522	-0.330737
C	-1.456063	-0.133855	0.081695
C	-3.451489	1.797758	0.287160
C	-3.702392	0.497990	0.718106
C	3.702575	0.522936	0.772514
H	-4.223940	2.554867	0.357308
H	-4.670031	0.230124	1.125064
H	-1.999763	3.158898	-0.530157
H	-2.884406	-1.472559	0.963720
Te	0.036877	-1.587968	-0.217361

PTE(-H)$^{\bullet}$ - Site 5
Solvent = Water
E = -784.630124
G = -784.504057
ZPC = 0.164766
TCE = 0.175599
TCH = 0.176544
TCG = 0.126067
N_{imag} = 0

H	0.076353	2.537336	-1.158767
H	2.150275	3.108161	-0.508455
H	4.341556	2.452486	0.415513
H	4.729752	0.102709	1.195173
N	0.058003	1.557288	-0.897533
C	3.552065	1.713785	0.334055
C	2.319744	2.084593	-0.191413
C	1.278964	1.155668	-0.324126
C	1.497958	-0.169687	0.088828
C	2.719528	-0.458796	0.628010
C	-2.177882	2.177429	-0.210508
C	-2.714596	-0.413991	0.625117
C	-1.185177	1.198234	-0.330186
C	-1.467168	-0.109686	0.080751
C	-3.429851	1.857070	0.296988
C	-3.703052	0.559797	0.724006
C	3.778624	0.403339	0.773045
H	-4.187823	2.628029	0.371156
H	-4.674118	0.307102	1.132171
H	-1.955939	3.192397	-0.524517
H	-2.918076	-1.425666	0.958770
Te	-0.015057	-1.611348	-0.203187

Solvent = Pentyl Ethanoate
E = -784.633718
G = -784.507784
ZPC = 0.164670
TCE = 0.175534
TCH = 0.176478
TCG = 0.125934
N_{imag} = 0

H	0.073414	2.536015	-1.156388
H	2.163872	3.108579	-0.496964
H	4.361506	2.438469	0.394321
H	4.746680	0.084552	1.164235
N	0.056404	1.567539	-0.859677
C	3.567433	1.703515	0.321055
C	2.330350	2.082183	-0.187089
C	1.282605	1.159487	-0.309648
C	1.502361	-0.168540	0.095509
C	2.727846	-0.465210	0.619493
C	-2.191073	2.173207	-0.203874
C	-2.722652	-0.422758	0.615344
C	-1.191171	1.200550	-0.315974
C	-1.472008	-0.110940	0.085333
C	-3.447703	1.843791	0.285445
C	-3.718204	0.544491	0.705136
C	3.792411	0.391896	0.754182
H	-4.211231	2.610069	0.352938
H	-4.692496	0.284778	1.101268
H	-1.971977	3.192125	-0.507776
H	-2.924928	-1.435851	0.946145
Te	-0.013478	-1.603491	-0.203260

TS[PS-OH$^{\bullet}$] - Site 1
Solvent = Pentyl Ethanoate
E = -991.308202
G = -991.160769
ZPC = 0.188402
TCE = 0.201512
TCH = 0.202456
TCG = 0.147433
N_{imag} = 1, -942.05 cm^{-1}

S	-0.006150	-1.861927	0.562734
N	-0.001080	1.138034	-0.202780
C	1.231603	0.503752	-0.208657
C	1.366626	-0.862712	0.071504
C	-3.760535	-0.686411	-0.262891
C	-3.627210	0.678673	-0.518877

C	-2.376446	1.269695	-0.496980
C	-1.236962	0.510163	-0.202544
C	-1.375483	-0.856504	0.072444
C	-2.637052	-1.448013	0.025956
C	2.372982	1.258575	-0.510876
C	2.626652	-1.457262	0.025129
H	-4.735988	-1.156400	-0.286720
H	-4.500068	1.280555	-0.740707
H	-2.257261	2.329075	-0.695654
C	3.622502	0.664025	-0.531090
C	3.752165	-0.699841	-0.268031
H	4.496342	1.261781	-0.760111
H	4.726106	-1.172947	-0.291628
H	0.006945	2.161354	-0.211823
H	2.255754	2.316175	-0.720432
H	2.722064	-2.517869	0.230646
H	-2.734937	-2.508990	0.228346
O	-0.011818	3.005937	1.310510
H	0.468488	2.539519	2.009202

TS[PS-OOH$^{\bullet}$] - Site 1
Solvent = Pentyl Ethanoate
E = -1066.459617
G = -1066.310738
ZPC = 0.190893
TCE = 0.204505
TCH = 0.205449
TCG = 0.148880
N_{imag} = 1, -2409.16 cm^{-1}

S	-0.244061	-2.150014	0.233226
N	-0.025783	0.826540	-0.767249
C	-1.283559	0.345758	-0.421547
C	-1.523452	-0.955838	0.044686
C	3.612174	-1.082507	0.046407
C	3.573884	0.229063	-0.434325
C	2.359261	0.833716	-0.692902
C	1.146289	0.150366	-0.475721
C	1.197164	-1.170105	0.005796
C	2.430666	-1.773248	0.260684
C	-2.374113	1.218070	-0.574930
C	-2.826766	-1.366135	0.335462
H	4.560214	-1.563829	0.252907
H	4.493931	0.773562	-0.609160
H	2.312575	1.846212	-1.080966
C	-3.658535	0.808141	-0.277142
C	-3.889713	-0.492921	0.177848
H	-4.484035	1.499088	-0.397388
H	-4.894730	-0.821627	0.412394
H	0.090419	1.891200	-0.531357
H	-2.180697	2.224587	-0.928823
H	-2.998577	-2.375098	0.694449
H	2.456782	-2.791454	0.633394
O	0.368170	3.033722	0.286097
O	0.917489	2.588025	1.458837
H	1.864492	2.491666	1.268316

TS[PS-OOCH$_3^{\bullet}$] - Site 1
Solvent = Pentyl Ethanoate
E = -1105.755117
G = -1105.580127
ZPC = 0.218964
TCE = 0.233843
TCH = 0.234787
TCG = 0.174991
N_{imag} = 1, -2627.74 cm^{-1}

S	-0.419559	-2.027898	0.724872
N	-0.085151	0.656332	-0.887739
C	-1.370742	0.257464	-0.542839
C	-1.667909	-0.930599	0.141010
C	3.425928	-1.537839	-0.288380
C	3.444946	-0.309692	-0.952754
C	2.269864	0.393112	-1.135916
C	1.044771	-0.105358	-0.654042
C	1.041160	-1.336545	0.027932
C	2.229609	-2.047133	0.194867
C	-2.429711	1.099266	-0.920545
C	-2.994863	-1.269454	0.414529
H	4.342015	-2.098632	-0.147494
H	4.377207	0.093535	-1.328774
H	2.262537	1.345059	-1.653597
C	-3.739215	0.769491	-0.627968
C	-4.026416	-0.425011	0.037413
H	-4.540629	1.435517	-0.923806
H	-5.051120	-0.695775	0.260771
H	0.106763	1.740854	-0.721350
H	-2.191609	2.014688	-1.451061
H	-3.211528	-2.196140	0.935208
H	2.213127	-2.999755	0.713173
O	0.424059	2.892186	0.005033
O	1.533978	2.556376	0.724646
C	1.137041	2.072184	1.997852
H	0.500147	1.188499	1.883532
H	2.056278	1.807859	2.521736
H	0.594735	2.848530	2.543515

TS[PSE-OH$^{\bullet}$] - Site 1
Solvent = Pentyl Ethanoate
E = -2994.741366
G = -2994.595958
ZPC = 0.187381
TCE = 0.200852
TCH = 0.201796
TCG = 0.145408
N_{imag} = 1, -923.69 cm^{-1}

Se	-0.009579	-1.762407	0.366910
N	0.001125	1.393636	-0.170076
C	1.244970	0.781084	-0.230102
C	1.431104	-0.596417	-0.056916
C	-3.820215	-0.297413	-0.373746
C	-3.632370	1.077443	-0.523484
C	-2.359866	1.614573	-0.455555
C	-1.247976	0.791425	-0.222326
C	-1.442132	-0.585175	-0.055181
C	-2.726392	-1.120353	-0.145929
C	2.361516	1.596233	-0.473249
C	2.712103	-1.138952	-0.147462
H	-4.813522	-0.725312	-0.432925
H	-4.479895	1.729325	-0.697398
H	-2.200479	2.681330	-0.570278
C	3.631087	1.051020	-0.539698
C	3.810822	-0.323665	-0.380950
H	4.481796	1.696288	-0.722567
H	4.801310	-0.758096	-0.439647

H	0.011015	2.414578	-0.105578
H	2.207413	2.662447	-0.600546
H	2.845380	-2.208218	-0.023491
H	-2.865717	-2.189345	-0.026411
O	0.005566	3.141844	1.479349
H	0.510073	2.629828	2.127207

TS[PSE-OOH•] - Site 1
Solvent = Pentyl Ethanoate
E = -3069.892053
G = -3069.745208
ZPC = 0.189871
TCE = 0.203858
TCH = 0.204802
TCG = 0.146846
N_{imag} = 1, -2635.71 cm^{-1}

Se	-0.311762	-1.967962	0.229817
N	0.048271	1.129874	-0.702191
C	-1.243215	0.703019	-0.417002
C	-1.591854	-0.594551	-0.014564
C	3.666885	-0.900518	-0.114274
C	3.635070	0.430743	-0.539275
C	2.423622	1.066713	-0.723940
C	1.203039	0.399855	-0.482502
C	1.248806	-0.940734	-0.060688
C	2.479624	-1.576252	0.116365
C	-2.269012	1.652926	-0.578954
C	-2.931785	-0.924524	0.202075
H	4.612887	-1.407103	0.033187
H	4.557646	0.965655	-0.730376
H	2.383226	2.093759	-1.072916
C	-3.589440	1.322401	-0.350485
C	-3.928098	0.022999	0.038194
H	-4.360219	2.073198	-0.475954
H	-4.962425	-0.245473	0.215488
H	0.211990	2.176040	-0.429158
H	-1.994823	2.656872	-0.883887
H	-3.185686	-1.933488	0.509372
H	2.498030	-2.610407	0.443296
O	0.552809	3.285274	0.425674
O	1.170021	2.788755	1.544299
H	2.096901	2.667858	1.283024

TS[PSE-OOCH$_3$•] - Site 1
Solvent = Pentyl Ethanoate
E = -3109.187825
G = -3109.014758
ZPC = 0.217856
TCE = 0.233086
TCH = 0.234030
TCG = 0.173066
N_{imag} = 1, -2443.18 cm^{-1}

Se	-0.376169	-1.841075	0.571854
N	-0.009385	1.038508	-0.865798
C	-1.313810	0.620769	-0.621248
C	-1.673586	-0.609802	-0.053622
C	3.522162	-1.202033	-0.656009
C	3.508194	0.093665	-1.180069
C	2.325089	0.802649	-1.233590
C	1.120178	0.246374	-0.757755
C	1.154041	-1.048617	-0.209391
C	2.349892	-1.766533	-0.176271
C	-2.336863	1.515524	-0.983286
C	-3.019723	-0.940255	0.117139
H	4.445023	-1.768461	-0.619728
H	4.422200	0.543063	-1.548841
H	2.293701	1.806266	-1.642254
C	-3.666390	1.194256	-0.789017
C	-4.013735	-0.044557	-0.243218
H	-4.437170	1.901099	-1.071736
H	-5.054660	-0.308315	-0.100112
H	0.194635	2.077805	-0.503029
H	-2.052433	2.464606	-1.424601
H	-3.280900	-1.901663	0.546347
H	2.357647	-2.769158	0.237703
O	0.518737	3.046227	0.434718
O	1.574211	2.515604	1.119808
C	1.090738	1.796781	2.242205
H	0.446971	0.970641	1.916960
H	1.971171	1.405056	2.753075
H	0.528564	2.462092	2.902352

TS[PTE-OH•] - Site 1
Solvent = Pentyl Ethanoate
E = -861.052297
G = -860.909164
ZPC = 0.186465
TCE = 0.200250
TCH = 0.201194
TCG = 0.143133
N_{imag} = 1, -1001.43 cm^{-1}

Te	-0.012666	-1.706393	0.298693
N	0.004945	1.612989	-0.073496
C	1.262101	1.035374	-0.219474
C	1.511663	-0.342872	-0.156073
C	-3.875174	0.103129	-0.535595
C	-3.618749	1.474841	-0.567577
C	-2.326727	1.941536	-0.415074
C	-1.259627	1.049989	-0.210673
C	-1.522210	-0.325488	-0.153295
C	-2.828068	-0.784972	-0.334156
C	2.337363	1.915756	-0.434847
C	2.812644	-0.815004	-0.337942
H	-4.884059	-0.270276	-0.663450
H	-4.427947	2.179418	-0.717769
H	-2.116750	3.005348	-0.443938
C	3.624964	1.435560	-0.587006
C	3.868224	0.061978	-0.545916
H	4.439936	2.131252	-0.747145
H	4.873055	-0.322009	-0.674317
H	0.017232	2.620144	0.121206
H	2.136734	2.981043	-0.476239
H	3.000870	-1.883076	-0.303432
H	-3.026520	-1.851311	-0.303670
O	0.014301	3.209747	1.733521
H	0.578606	2.674031	2.308788

TS[PTE-OOH•] - Site 1
Solvent = Pentyl Ethanoate
E = -936.201799
G = -936.057074
ZPC = 0.188770
TCE = 0.203142

TCH = 0.204087
TCG = 0.144726
N_{imag} = 1, -2988.47 cm^{-1}

Te	-0.423835	-1.878739	0.191515
N	0.182880	1.370968	-0.646782
C	-1.150086	1.051696	-0.410984
C	-1.655036	-0.213701	-0.070685
C	3.747926	-0.794015	-0.213520
C	3.739818	0.552548	-0.588277
C	2.541234	1.222482	-0.721452
C	1.305010	0.578097	-0.477647
C	1.324148	-0.781490	-0.111564
C	2.547203	-1.447018	0.013979
C	-2.060260	2.118433	-0.564488
C	-3.032916	-0.388353	0.095276
H	4.683738	-1.328720	-0.104577
H	4.670571	1.072736	-0.781063
H	2.521143	2.262198	-1.032554
C	-3.415177	1.935778	-0.383995
C	-3.911819	0.671022	-0.055416
H	-4.090322	2.775078	-0.500338
H	-4.974812	0.516622	0.085517
H	0.411944	2.399796	-0.352860
H	-1.665913	3.095223	-0.822229
H	-3.418232	-1.368803	0.355669
H	2.556117	-2.494029	0.299551
O	0.797000	3.479755	0.519683
O	1.432726	2.937574	1.609898
H	2.346931	2.796017	1.317558

TS[PTE-OOCH$_3^{\bullet}$] - Site 1
Solvent = Pentyl Ethanoate
E = -975.497891
G = -975.326932
ZPC = 0.217011
TCE = 0.232554
TCH = 0.233498
TCG = 0.170960
N_{imag} = 1, -2660.93 cm^{-1}

Te	-0.453482	-1.811475	0.411030
N	0.118964	1.302161	-0.789020
C	-1.221668	0.970224	-0.621796
C	-1.724342	-0.269008	-0.193836
C	3.617500	-1.000288	-0.832485
C	3.613628	0.346685	-1.205952
C	2.437686	1.066494	-1.173215
C	1.224675	0.471053	-0.759857
C	1.246396	-0.878597	-0.357201
C	2.440201	-1.601890	-0.413659
C	-2.138230	1.999109	-0.919890
C	-3.105730	-0.467657	-0.105341
H	4.535047	-1.575751	-0.864495
H	4.530155	0.828145	-1.525121
H	2.416377	2.110314	-1.465417
C	-3.499304	1.800526	-0.804018
C	-3.991734	0.555444	-0.402918
H	-4.182083	2.609519	-1.034802
H	-5.058890	0.387153	-0.321007
H	0.369783	2.307740	-0.364175
H	-1.744262	2.954712	-1.249085
H	-3.488259	-1.431394	0.214760
H	2.447490	-2.645812	-0.117095
O	0.720805	3.202632	0.636146
O	1.722250	2.567096	1.321444
C	1.153202	1.781718	2.352793
H	0.493284	1.014215	1.928255
H	1.988137	1.308896	2.871714
H	0.583402	2.413138	3.039569

Radical Adduct Formation

PS-OH$^{\bullet}$ - Site 2a
Solvent = Water
E = -991.338794
G = -991.184350
ZPC = 0.192928
TCE = 0.204762
TCH = 0.205707
TCG = 0.154444
N_{imag} = 0

S	0.103554	1.811177	0.036093
N	-0.010070	-1.233312	-0.512086
C	-1.105409	-0.690536	0.259108
C	-1.292772	0.771902	-0.065866
C	3.846860	0.358817	0.035496
C	3.671716	-0.995278	-0.250007
C	2.396605	-1.514666	-0.412431
C	1.266525	-0.695858	-0.307167
C	1.450582	0.666239	-0.036460
C	2.735887	1.182237	0.143437
C	-2.349528	-1.488492	-0.007292
C	-2.529575	1.311967	-0.336456
H	4.838963	0.770075	0.173945
H	4.530006	-1.651023	-0.335188
H	2.252761	-2.570025	-0.620187
C	-3.542693	-0.910773	-0.275831
C	-3.665373	0.501644	-0.420904
H	-4.422820	-1.531732	-0.398283
H	-4.629042	0.944398	-0.635076
H	-0.008912	-2.248977	-0.495109
H	-2.241858	-2.564010	0.094330
H	-2.619690	2.379532	-0.508308
H	2.859436	2.237740	0.361967
O	-0.746766	-0.828545	1.653637
H	-1.468036	-0.466482	2.186158

Solvent = Pentyl Ethanoate
E = -991.338814
G = -991.185128
ZPC = 0.192491
TCE = 0.204532
TCH = 0.205476
TCG = 0.153686
N_{imag} = 0

S	0.109159	1.803813	0.086358
N	-0.009811	-1.246268	-0.442806
C	-1.116539	-0.687699	0.292712
C	-1.285631	0.771889	-0.063633
C	3.852525	0.356667	-0.014070
C	3.671951	-0.997292	-0.295157
C	2.394666	-1.518718	-0.421265
C	1.264508	-0.704035	-0.281129

C	1.454087	0.659492	-0.018651
C	2.743035	1.176674	0.123527
C	-2.360395	-1.480924	0.004266
C	-2.509381	1.312107	-0.390456
H	4.847384	0.770086	0.096075
H	4.528924	-1.651279	-0.405729
H	2.248723	-2.574614	-0.624921
C	-3.539203	-0.901268	-0.317763
C	-3.646384	0.507756	-0.500794
H	-4.419934	-1.519063	-0.453334
H	-4.598195	0.950901	-0.762664
H	-0.018982	-2.259733	-0.459189
H	-2.269771	-2.554238	0.140799
H	-2.584617	2.376420	-0.588315
H	2.870305	2.233269	0.335302
O	-0.789664	-0.809102	1.691787
H	-1.503831	-0.403957	2.200069

PS-OH$^{\bullet}$ - Site 2

Solvent = Water
E = -991.338839
G = -991.184718
ZPC = 0.193384
TCE = 0.205442
TCH = 0.206386
TCG = 0.154121
N_{imag} = 0

S	0.413893	1.808473	0.630668
N	0.049433	-1.175926	0.340131
C	-1.062513	-0.421596	0.043056
C	-1.040225	0.960347	0.040004
C	3.960574	0.149741	-0.396474
C	3.666774	-1.211116	-0.402049
C	2.369292	-1.648468	-0.165803
C	1.356688	-0.728476	0.107247
C	1.656112	0.638116	0.132574
C	2.949932	1.071929	-0.140027
C	-2.309817	-1.202641	-0.283528
C	-2.163929	1.705383	-0.321622
H	4.968363	0.495617	-0.590199
H	4.445617	-1.937227	-0.601516
H	2.125800	-2.705335	-0.187535
C	-3.466159	-0.321269	-0.631056
C	-3.377412	1.030638	-0.642623
H	-4.394053	-0.826547	-0.874459
H	-4.246467	1.625484	-0.900874
H	-0.079338	-2.180216	0.278468
H	-2.085429	-1.886291	-1.112375
H	-2.119842	2.786965	-0.332535
H	3.167909	2.134030	-0.132902
O	-2.635079	-2.098711	0.799035
H	-2.906158	-1.556407	1.551856

Solvent = Pentyl Ethanoate
E = -991.341431
G = -991.187425
ZPC = 0.193221
TCE = 0.205285
TCH = 0.206229
TCG = 0.154005
N_{imag} = 0

S	0.413280	1.828462	0.588491
N	0.044121	-1.161336	0.322077
C	-1.067645	-0.406450	0.035571
C	-1.047883	0.974566	0.027042
C	3.964741	0.148935	-0.379314
C	3.672736	-1.211260	-0.361988
C	2.372594	-1.645160	-0.134781
C	1.354659	-0.722014	0.107349
C	1.653440	0.645945	0.113586
C	2.950041	1.073935	-0.151536
C	-2.307907	-1.195404	-0.306557
C	-2.187305	1.713163	-0.297193
H	4.974535	0.492409	-0.567491
H	4.454849	-1.940360	-0.537159
H	2.134960	-2.703683	-0.140543
C	-3.495881	-0.319568	-0.543890
C	-3.412912	1.032847	-0.552267
H	-4.433745	-0.828693	-0.736224
H	-4.299826	1.624294	-0.751702
H	-0.097446	-2.164743	0.329995
H	-2.096057	-1.796698	-1.202788
H	-2.147753	2.794938	-0.311803
H	3.166748	2.136389	-0.160490
O	-2.554202	-2.193009	0.696723
H	-2.836045	-1.733033	1.497884

PS-OH$^{\bullet}$ - Site 3

Solvent = Water
E = -991.336142
G = -991.181902
ZPC = 0.193413
TCE = 0.205447
TCH = 0.206391
TCG = 0.154241
N_{imag} = 0

S	-0.495081	-1.756814	0.477733
N	-0.246678	1.256211	0.516532
C	0.920975	0.595147	0.094526
C	0.911916	-0.835928	0.011358
C	-4.104132	-0.155313	-0.366218
C	-3.852269	1.213112	-0.291658
C	-2.571447	1.679625	-0.027799
C	-1.520766	0.784059	0.190666
C	-1.782491	-0.588676	0.122295
C	-3.062478	-1.054209	-0.166992
C	2.052205	1.289495	-0.191165
C	2.097807	-1.525571	-0.353643
H	-5.099857	-0.522051	-0.581882
H	-4.654668	1.923973	-0.449357
H	-2.368276	2.744246	0.020523
C	3.355565	0.630842	-0.509001
C	3.248534	-0.858174	-0.617775
H	3.760550	1.038478	-1.441821
H	4.145790	-1.398421	-0.897625
H	-0.168068	2.266257	0.476516
H	2.040469	2.373981	-0.143764
H	2.067932	-2.607970	-0.423963
H	-3.241222	-2.122095	-0.227759
O	4.367441	0.988956	0.465010
H	4.065357	0.661053	1.322025

Solvent = Pentyl Ethanoate

E = -991.337915
G = -991.183842
ZPC = 0.193388
TCE = 0.205491
TCH = 0.206435
TCG = 0.154074
N_{imag} = 0

S	-0.495192	-1.754841	0.451630
N	-0.248100	1.263774	0.453817
C	0.925513	0.598673	0.067312
C	0.913846	-0.834412	-0.005414
C	-4.120278	-0.160454	-0.325574
C	-3.870576	1.208160	-0.260815
C	-2.585429	1.678035	-0.028668
C	-1.526082	0.786785	0.167767
C	-1.786392	-0.587305	0.112000
C	-3.071754	-1.054905	-0.146395
C	2.065216	1.287190	-0.199203
C	2.106835	-1.527338	-0.338975
H	-5.119703	-0.530608	-0.517380
H	-4.678194	1.916832	-0.401918
H	-2.386640	2.744017	0.010570
C	3.375325	0.625767	-0.487315
C	3.264416	-0.864848	-0.584253
H	3.792060	1.021363	-1.421030
H	4.167340	-1.409472	-0.836475
H	-0.169277	2.272956	0.444383
H	2.058912	2.372214	-0.159581
H	2.076117	-2.610542	-0.398715
H	-3.248783	-2.123660	-0.198570
O	4.371588	0.994479	0.488379
H	4.051400	0.690001	1.346071

PS-OH$^{\bullet}$ - Site 4
Solvent = Water
E = -991.339005
G = -991.184452
ZPC = 0.193603
TCE = 0.205620
TCH = 0.206564
TCG = 0.154553
N_{imag} = 0

S	0.288535	-1.676400	-0.354481
N	0.429376	1.366183	-0.470422
C	-0.808106	0.858628	-0.137538
C	-1.020370	-0.540211	0.046508
C	4.089341	-0.506941	0.343620
C	4.023288	0.875652	0.180416
C	2.807560	1.491428	-0.080160
C	1.641251	0.730450	-0.202586
C	1.711174	-0.660203	-0.054486
C	2.931203	-1.268235	0.232883
C	-1.907308	1.742343	0.045986
C	-2.227813	-1.030572	0.423829
H	5.033737	-0.991643	0.557836
H	4.918770	1.479197	0.266780
H	2.742352	2.568374	-0.194119
C	-3.136461	1.288895	0.391890
C	-3.444960	-0.166757	0.553547
H	-3.954151	1.985079	0.540043
H	-3.935908	-0.347631	1.515243
H	0.481303	2.377085	-0.531113
H	-1.728853	2.806149	-0.080891
H	-2.361458	-2.095852	0.579167
H	2.972033	-2.344591	0.358503
O	-4.456712	-0.578242	-0.403094
H	-4.089122	-0.437974	-1.285510

Solvent = Pentyl Ethanoate
E = -991.340452
G = -991.186294
ZPC = 0.193504
TCE = 0.205602
TCH = 0.206546
TCG = 0.154158
N_{imag} = 0

S	0.288818	-1.672756	-0.346225
N	0.433702	1.373046	-0.436663
C	-0.808743	0.863523	-0.127769
C	-1.021223	-0.537079	0.052495
C	4.096133	-0.513474	0.325697
C	4.033273	0.869243	0.169154
C	2.816659	1.489261	-0.075127
C	1.646089	0.733473	-0.189194
C	1.713064	-0.658755	-0.050176
C	2.934419	-1.269782	0.222586
C	-1.913425	1.743941	0.034808
C	-2.231599	-1.027135	0.417783
H	5.041131	-1.002167	0.528446
H	4.931635	1.469725	0.248550
H	2.757699	2.567588	-0.182325
C	-3.146111	1.289540	0.367402
C	-3.453268	-0.166188	0.542761
H	-3.969738	1.983327	0.492691
H	-3.935153	-0.336546	1.511980
H	0.485260	2.381195	-0.515543
H	-1.740047	2.807893	-0.100658
H	-2.367389	-2.093394	0.565113
H	2.972273	-2.346886	0.343425
O	-4.465613	-0.588884	-0.394858
H	-4.099380	-0.466311	-1.279098

PS-OH$^{\bullet}$ - Site 5
Solvent = Water
E = -991.335996
G = -991.181519
ZPC = 0.193253
TCE = 0.205176
TCH = 0.206120
TCG = 0.154477
N_{imag} = 0

S	-0.042595	-1.572220	0.414140
N	-0.405405	1.383100	0.770034
C	0.852812	1.000523	0.259525
C	1.140510	-0.321583	0.039614
C	-3.856904	-0.614368	-0.560987
C	-3.893549	0.753962	-0.307093
C	-2.748795	1.422995	0.110566
C	-1.557722	0.723817	0.311463
C	-1.529170	-0.653140	0.069201
C	-2.666574	-1.313952	-0.384119
C	1.824914	1.991653	0.005417

C	2.463170	-0.793966	-0.490810
H	-4.744621	-1.136375	-0.896041
H	-4.813812	1.309138	-0.444801
H	-2.767753	2.492113	0.293733
C	3.121659	1.617896	-0.430618
C	3.446864	0.321274	-0.663426
H	3.862941	2.395345	-0.576428
H	4.441429	0.037423	-0.988480
H	-0.521221	2.389443	0.814239
H	1.577755	3.032543	0.177794
H	2.301876	-1.293045	-1.455872
H	-2.622509	-2.378997	-0.583189
O	3.002523	-1.841045	0.336265
H	3.201799	-1.456072	1.200057

Solvent = Pentyl Ethanoate
E = -991.337410
G = -991.183273
ZPC = 0.193088
TCE = 0.205089
TCH = 0.206033
TCG = 0.154137
N_{imag} = 0

S	-0.042779	-1.569469	0.386965
N	-0.404348	1.391602	0.730306
C	0.856869	1.006375	0.238299
C	1.141607	-0.320060	0.023086
C	-3.870013	-0.616876	-0.535232
C	-3.908384	0.750270	-0.280485
C	-2.759367	1.423655	0.117542
C	-1.561822	0.729950	0.298870
C	-1.531798	-0.647983	0.058699
C	-2.674089	-1.311103	-0.376477
C	1.836959	1.989627	-0.003466
C	2.474376	-0.800728	-0.477729
H	-4.760814	-1.142610	-0.856337
H	-4.833225	1.301747	-0.402702
H	-2.782728	2.493206	0.299460
C	3.140614	1.608607	-0.416241
C	3.464252	0.311207	-0.640522
H	3.887867	2.382720	-0.550770
H	4.463736	0.021876	-0.945451
H	-0.516913	2.393769	0.816943
H	1.595674	3.033580	0.160140
H	2.328841	-1.308266	-1.441190
H	-2.628586	-2.376173	-0.575793
O	2.990385	-1.840406	0.366104
H	3.182738	-1.443963	1.225224

PS-OH• - Site 5a
Solvent = Water
E = -991.344115
G = -991.189944
ZPC = 0.192632
TCE = 0.204655
TCH = 0.205600
TCG = 0.154171
N_{imag} = 0

S	0.034543	-1.525607	-0.651981
N	-0.103325	1.464171	-0.086755
C	1.139832	0.891457	-0.069385
C	1.219233	-0.539730	0.374422
C	-3.822295	-0.461786	-0.056397
C	-3.729435	0.912291	0.168134
C	-2.492463	1.536251	0.178796
C	-1.323714	0.800049	-0.053078
C	-1.417244	-0.581435	-0.284169
C	-2.666607	-1.201925	-0.271094
C	2.275501	1.622849	-0.381524
C	2.585795	-1.119334	0.231349
H	-4.786403	-0.954990	-0.056329
H	-4.623756	1.498274	0.343054
H	-2.409167	2.602727	0.359608
C	3.531069	1.024864	-0.401494
C	3.659691	-0.362583	-0.107377
H	4.405808	1.607596	-0.658299
H	4.640246	-0.823222	-0.152406
H	-0.143655	2.471696	-0.192713
H	2.157845	2.671313	-0.636454
H	2.683486	-2.172564	0.471354
H	-2.726935	-2.271836	-0.436873
O	0.733747	-0.675625	1.717665
H	1.346966	-0.189292	2.287632

Solvent = Pentyl Ethanoate
E = -991.345410
G = -991.190927
ZPC = 0.192833
TCE = 0.204795
TCH = 0.205739
TCG = 0.154482
N_{imag} = 0

S	0.036084	-1.519829	-0.659465
N	-0.102932	1.464275	-0.085862
C	1.140799	0.893324	-0.068266
C	1.217261	-0.539883	0.375659
C	-3.820593	-0.462891	-0.056057
C	-3.730280	0.909839	0.171265
C	-2.493662	1.534745	0.183097
C	-1.324774	0.801028	-0.052122
C	-1.415626	-0.579841	-0.286088
C	-2.663895	-1.200630	-0.272557
C	2.277110	1.622356	-0.381707
C	2.585456	-1.117908	0.237896
H	-4.783956	-0.957885	-0.054893
H	-4.625362	1.493982	0.349382
H	-2.415278	2.600630	0.371320
C	3.532897	1.023354	-0.399405
C	3.660385	-0.362303	-0.100268
H	4.407796	1.604733	-0.659150
H	4.641074	-0.823340	-0.141275
H	-0.143184	2.469606	-0.199049
H	2.164467	2.670141	-0.642755
H	2.681664	-2.170472	0.481284
H	-2.721779	-2.270900	-0.437017
O	0.723128	-0.679087	1.710660
H	1.362235	-0.243595	2.290665

TS[PS-OH•] - Site 2a
Solvent = Water
E = -991.312213
G = -991.160995
ZPC = 0.190378

TCE = 0.202706
TCH = 0.203650
TCG = 0.151219
N_{imag} = 1, -302.58 cm^{-1}

S	0.106835	1.794367	-0.190149
N	0.060932	-1.264255	-0.553914
C	-1.123411	-0.690228	-0.184758
C	-1.269852	0.737993	-0.160064
C	3.842779	0.384086	0.168047
C	3.694698	-0.985493	-0.056986
C	2.440309	-1.524576	-0.287816
C	1.308774	-0.702612	-0.303812
C	1.461954	0.670969	-0.089756
C	2.727725	1.207633	0.151740
C	-2.310354	-1.483184	-0.315314
C	-2.542723	1.305524	-0.087205
H	4.821445	0.807548	0.355454
H	4.560665	-1.636047	-0.045975
H	2.313578	-2.588644	-0.456161
C	-3.542933	-0.900933	-0.256031
C	-3.666830	0.501735	-0.123735
H	-4.433179	-1.513930	-0.321915
H	-4.649358	0.953472	-0.070704
H	0.048313	-2.275904	-0.632886
H	-2.190191	-2.554926	-0.427156
H	-2.643905	2.382657	-0.018499
H	2.831761	2.273456	0.322757
O	-0.895064	-0.795220	1.910613
H	-1.755326	-0.471504	2.204092

Solvent = Pentyl Ethanoate
E = -991.306711
G = -991.156095
ZPC = 0.190010
TCE = 0.202463
TCH = 0.203407
TCG = 0.150615
N_{imag} = 1, -351.17 cm^{-1}

S	0.097967	1.814262	-0.314180
N	0.060205	-1.207731	-0.742379
C	-1.128253	-0.665920	-0.278572
C	-1.280688	0.744018	-0.153218
C	3.805310	0.368763	0.310972
C	3.662553	-0.988836	0.040889
C	2.418452	-1.509490	-0.290028
C	1.302047	-0.677702	-0.376825
C	1.452530	0.687272	-0.117361
C	2.695141	1.202582	0.237786
C	-2.305048	-1.465284	-0.320156
C	-2.541732	1.293394	0.058608
H	4.770500	0.778718	0.581449
H	4.518882	-1.650275	0.098451
H	2.298327	-2.570164	-0.484822
C	-3.544609	-0.902691	-0.133333
C	-3.667963	0.481394	0.066820
H	-4.427290	-1.530321	-0.141859
H	-4.644881	0.923344	0.220534
H	0.049482	-2.218988	-0.797538
H	-2.189681	-2.532856	-0.472123
H	-2.639518	2.364451	0.195630
H	2.791797	2.261257	0.452747
O	-0.767228	-0.903767	1.766430
H	-1.585140	-0.574087	2.166127

TS[PS-OH$^{\bullet}$] - Site 2
Solvent = Water
E = -991.308891
G = -991.159160
ZPC = 0.189834
TCE = 0.202441
TCH = 0.203385
TCG = 0.149732
N_{imag} = 1, -409.12 cm^{-1}

S	-0.334528	-1.708849	0.695028
N	-0.084300	1.258473	0.213838
C	1.040630	0.533142	-0.124893
C	1.048498	-0.870260	-0.040844
C	-3.970833	-0.242093	-0.306571
C	-3.721459	1.122616	-0.422429
C	-2.432317	1.616158	-0.263913
C	-1.381570	0.751047	0.042415
C	-1.636719	-0.617301	0.174944
C	-2.922909	-1.111540	-0.019525
C	2.213526	1.199713	-0.532560
C	2.167566	-1.582938	-0.437010
H	-4.972504	-0.631411	-0.439946
H	-4.529465	1.807856	-0.648703
H	-2.225801	2.675845	-0.370856
C	3.338267	0.460013	-0.934993
C	3.320602	-0.918999	-0.882245
H	4.221392	0.990260	-1.269767
H	4.186685	-1.495308	-1.181997
H	-0.001821	2.257761	0.059957
H	2.178095	2.273029	-0.673985
H	2.154355	-2.666013	-0.385763
H	-3.103974	-2.176795	0.070299
O	2.824113	1.599099	1.619726
H	3.062990	0.666910	1.751369

Solvent = Pentyl Ethanoate
E = -991.308433
G = -991.157955
ZPC = 0.190337
TCE = 0.202815
TCH = 0.203759
TCG = 0.150478
N_{imag} = 1, -345.69 cm^{-1}

S	-0.358260	-1.726024	0.695435
N	-0.071361	1.234532	0.189906
C	1.051417	0.498905	-0.116436
C	1.046775	-0.902602	-0.017050
C	-3.975457	-0.217194	-0.309794
C	-3.712653	1.144438	-0.421246
C	-2.417456	1.623011	-0.267095
C	-1.374154	0.746659	0.032587
C	-1.643874	-0.619766	0.165250
C	-2.935556	-1.097872	-0.027832
C	2.242194	1.159858	-0.497713
C	2.169335	-1.627862	-0.379600
H	-4.981641	-0.595845	-0.440946
H	-4.514372	1.839165	-0.641610
H	-2.202564	2.681215	-0.372796

C	3.368230	0.399408	-0.876194
C	3.338708	-0.976288	-0.804080
H	4.261128	0.919356	-1.200972
H	4.207028	-1.562434	-1.078143
H	0.031389	2.238068	0.102680
H	2.195949	2.215410	-0.731081
H	2.144888	-2.710271	-0.318223
H	-3.127546	-2.161411	0.060800
O	2.786607	1.801000	1.435992
H	2.979515	0.919238	1.791278

TS[PS-OH•] - Site 3
Solvent = Water
E = -991.305701
G = -991.155684
ZPC = 0.189990
TCE = 0.202490
TCH = 0.203434
TCG = 0.150018
N_{imag} = 1, -286.79 cm^{-1}

S	-0.406413	1.721435	-0.497768
N	-0.252582	-1.288333	-0.470195
C	0.898395	-0.653314	0.003706
C	0.950139	0.758466	0.079401
C	-4.086481	0.250974	0.293072
C	-3.880372	-1.124947	0.230525
C	-2.609455	-1.636458	0.000229
C	-1.528764	-0.775779	-0.199520
C	-1.743462	0.604405	-0.148674
C	-3.011297	1.114853	0.113806
C	2.011579	-1.387018	0.372183
C	2.098626	1.404574	0.537433
H	-5.074147	0.652066	0.483248
H	-4.709841	-1.807221	0.373030
H	-2.439799	-2.707432	-0.033510
C	3.200447	-0.738858	0.751504
C	3.217819	0.667537	0.882213
H	4.013928	-1.325748	1.155132
H	4.113591	1.165147	1.230884
H	-0.203627	-2.300222	-0.422751
H	1.971687	-2.469678	0.327614
H	2.107716	2.485501	0.615431
H	-3.155659	2.188237	0.165524
O	4.357487	-0.646829	-1.073096
H	3.683897	-0.137252	-1.549436

Solvent = Pentyl Ethanoate
E = -991.304805
G = -991.154235
ZPC = 0.190300
TCE = 0.202714
TCH = 0.203658
TCG = 0.150570
N_{imag} = 1, -441.85 cm^{-1}

S	-0.426961	1.724554	-0.527128
N	-0.243475	-1.275457	-0.471052
C	0.907046	-0.627793	-0.006382
C	0.944190	0.781777	0.059122
C	-4.086771	0.223285	0.314619
C	-3.869199	-1.149807	0.250639
C	-2.594767	-1.649254	0.012971
C	-1.523231	-0.779498	-0.196124
C	-1.750234	0.599685	-0.147167
C	-3.020226	1.096448	0.126743
C	2.026703	-1.348720	0.367689
C	2.089572	1.440033	0.512549
H	-5.076834	0.615511	0.511499
H	-4.691669	-1.839708	0.398418
H	-2.417691	-2.719249	-0.019970
C	3.221310	-0.690139	0.728146
C	3.217005	0.718175	0.858503
H	4.019585	-1.265175	1.175800
H	4.110051	1.227505	1.197461
H	-0.180008	-2.285941	-0.451657
H	1.999933	-2.432322	0.331466
H	2.086379	2.521651	0.585629
H	-3.173912	2.168646	0.178330
O	4.342976	-0.763544	-0.990244
H	3.747669	-0.232373	-1.541449

TS[PS-OH•] - Site 4
Solvent = Water
E = -991.310220
G = -991.160948
ZPC = 0.190334
TCE = 0.202924
TCH = 0.203868
TCG = 0.149272
N_{imag} = 1, -363.72 cm^{-1}

S	0.312535	-1.704392	0.178792
N	0.372704	1.394162	0.032202
C	-0.846145	0.833942	0.213287
C	-1.029045	-0.575068	0.305463
C	4.106971	-0.461638	-0.189811
C	3.998115	0.928726	-0.258948
C	2.755233	1.530346	-0.184667
C	1.602064	0.750047	-0.042594
C	1.710046	-0.642731	0.024943
C	2.968894	-1.240732	-0.048589
C	-1.973446	1.683753	0.332115
C	-2.293120	-1.092662	0.494724
H	5.077477	-0.938396	-0.246045
H	4.884146	1.541288	-0.369009
H	2.650580	2.608669	-0.234867
C	-3.222046	1.157093	0.503530
C	-3.415802	-0.249376	0.523025
H	-4.078032	1.813382	0.597245
H	-4.375282	-0.654279	0.806502
H	0.400012	2.408902	-0.025397
H	-1.814234	2.755633	0.291161
H	-2.427920	-2.164773	0.581323
H	3.051554	-2.320596	0.005074
O	-4.222227	-0.398148	-1.578365
H	-3.370282	-0.083711	-1.900019

Solvent = Pentyl Ethanoate
E = -991.307614
G = -991.157469
ZPC = 0.190197
TCE = 0.202731
TCH = 0.203675
TCG = 0.150145
N_{imag} = 1, -351.12 cm^{-1}

S	0.264295	-1.684988	-0.334222
N	0.382143	1.321513	-0.563261
C	-0.825960	0.839985	-0.093626
C	-1.006363	-0.538903	0.142586
C	4.065969	-0.475449	0.337734
C	3.987090	0.897295	0.124916
C	2.764515	1.492904	-0.159365
C	1.610666	0.715861	-0.264157
C	1.694485	-0.666198	-0.066538
C	2.915017	-1.252393	0.252433
C	-1.890224	1.722968	0.152145
C	-2.204454	-1.007048	0.641933
H	5.014986	-0.942402	0.570787
H	4.876679	1.512282	0.191151
H	2.694171	2.565444	-0.308349
C	-3.099988	1.247677	0.611770
C	-3.297021	-0.133419	0.824000
H	-3.915673	1.936218	0.794910
H	-4.174354	-0.481331	1.349414
H	0.424730	2.327697	-0.671068
H	-1.740314	2.783612	-0.019475
H	-2.326646	-2.065176	0.842909
H	2.963115	-2.322641	0.419820
O	-4.412747	-0.544457	-0.920946
H	-3.700835	-0.308514	-1.535129

TS[PS-OH$^{\bullet}$] - Site 5

Solvent = Water
E = -991.305026
G = -991.154138
ZPC = 0.190347
TCE = 0.202640
TCH = 0.203585
TCG = 0.150887
N_{imag} = 1, -354.03 cm^{-1}

S	0.070812	-1.629482	-0.205073
N	0.356397	1.299802	-0.793179
C	-0.867102	0.944556	-0.203712
C	-1.121296	-0.378791	0.149596
C	3.905974	-0.541798	0.501266
C	3.904117	0.803907	0.144529
C	2.727804	1.422712	-0.262559
C	1.542250	0.692143	-0.348237
C	1.551613	-0.662282	-0.001783
C	2.722047	-1.270850	0.439609
C	-1.855459	1.908135	0.016119
C	-2.377312	-0.750633	0.678252
H	4.818802	-1.023539	0.828885
H	4.819313	1.381775	0.193480
H	2.716235	2.475213	-0.525139
C	-3.090628	1.557198	0.566900
C	-3.349006	0.248185	0.922249
H	-3.836888	2.325546	0.726592
H	-4.297925	-0.036885	1.358260
H	0.453062	2.299676	-0.933286
H	-1.649134	2.938228	-0.255313
H	-2.492580	-1.732765	1.117313
H	2.707631	-2.318093	0.720066
O	-3.235085	-1.553721	-1.054223
H	-3.143616	-0.741186	-1.577028

Solvent = Pentyl Ethanoate
E = -991.304623
G = -991.153758
ZPC = 0.190325
TCE = 0.202659
TCH = 0.203604
TCG = 0.150864
N_{imag} = 1, -456.41 cm^{-1}

S	0.061072	-1.615736	-0.207447
N	0.361274	1.317968	-0.760579
C	-0.871860	0.962170	-0.196576
C	-1.127843	-0.364185	0.150243
C	3.905363	-0.554102	0.494509
C	3.912816	0.791321	0.140364
C	2.738352	1.420524	-0.255523
C	1.545553	0.700669	-0.333738
C	1.545395	-0.656331	0.006525
C	2.714805	-1.272645	0.438223
C	-1.865868	1.922343	0.007796
C	-2.391886	-0.744290	0.656007
H	4.816474	-1.044321	0.814718
H	4.833475	1.361305	0.182899
H	2.737688	2.474213	-0.515118
C	-3.110833	1.563074	0.533231
C	-3.370169	0.253727	0.884144
H	-3.864249	2.327589	0.679698
H	-4.326510	-0.035149	1.301066
H	0.458689	2.310780	-0.933884
H	-1.661195	2.955304	-0.254606
H	-2.496282	-1.714871	1.121827
H	2.693706	-2.320817	0.715316
O	-3.160974	-1.622706	-1.005160
H	-3.153030	-0.840032	-1.578671

TS[PS-OH$^{\bullet}$] - Site 5a

Solvent = Water
E = -991.313677
G = -991.161931
ZPC = 0.190738
TCE = 0.203000
TCH = 0.203944
TCG = 0.151746
N_{imag} = 1, -171.44 cm^{-1}

S	-0.065317	-1.657206	-0.546603
N	-0.085663	1.405301	-0.378104
C	1.132188	0.820735	-0.259537
C	1.259191	-0.592443	-0.105357
C	-3.825130	-0.396730	0.131773
C	-3.696694	0.992607	0.148735
C	-2.452645	1.579046	-0.011003
C	-1.317653	0.783404	-0.199128
C	-1.447303	-0.610230	-0.219483
C	-2.702631	-1.192682	-0.048661
C	2.293281	1.621259	-0.243959
C	2.554453	-1.169201	-0.089954
H	-4.795358	-0.859268	0.263069
H	-4.568108	1.619388	0.292659
H	-2.337096	2.657363	0.006338
C	3.532045	1.036753	-0.152098
C	3.667105	-0.370049	-0.082575
H	4.417461	1.660595	-0.144464

H	4.653431	-0.814462	-0.038612
H	-0.099390	2.420217	-0.407053
H	2.181014	2.697345	-0.316325
H	2.643849	-2.248655	-0.050915
H	-2.795142	-2.272864	-0.058815
O	0.842298	-0.412306	2.045547
H	1.628423	0.102165	2.269584

Solvent = Pentyl Ethanoate
E = -991.308479
G = -991.157282
ZPC = 0.190168
TCE = 0.202532
TCH = 0.203476
TCG = 0.151197
N_{imag} = 1, -261.05 cm^{-1}

S	-0.060736	-1.644329	-0.659262
N	-0.076686	1.390262	-0.527336
C	1.145632	0.813516	-0.304845
C	1.259972	-0.591286	-0.128027
C	-3.790464	-0.391310	0.253227
C	-3.663455	0.994011	0.226672
C	-2.427553	1.577697	-0.018989
C	-1.306914	0.782236	-0.261904
C	-1.438969	-0.610442	-0.246917
C	-2.673724	-1.189071	0.025177
C	2.299632	1.608023	-0.227864
C	2.542227	-1.167617	0.014060
H	-4.749353	-0.852266	0.456006
H	-4.524767	1.625553	0.408941
H	-2.318531	2.657353	-0.024212
C	3.536599	1.023190	-0.041701
C	3.663893	-0.371787	0.081106
H	4.418851	1.650615	0.006857
H	4.640584	-0.819653	0.215134
H	-0.087249	2.402317	-0.540162
H	2.202587	2.683209	-0.332035
H	2.619748	-2.245893	0.095237
H	-2.758963	-2.269666	0.053323
O	0.701927	-0.377512	1.927936
H	1.569012	-0.097002	2.257010

PS-OOH• - Site 2a
Solvent = Water
E = -1066.466405
G = -1066.310259
ZPC = 0.196356
TCE = 0.209461
TCH = 0.210406
TCG = 0.156146
N_{imag} = 0

S	0.231987	1.812611	-0.032412
N	0.139194	-1.232882	-0.639265
C	-1.012120	-0.674173	0.003718
C	-1.155760	0.791960	-0.292664
C	3.970660	0.355660	0.062730
C	3.805490	-0.993015	-0.253683
C	2.537766	-1.508888	-0.471480
C	1.405184	-0.691310	-0.386380
C	1.578760	0.665413	-0.087361
C	2.857156	1.177841	0.144983
C	-2.238453	-1.458133	-0.338139
C	-2.364650	1.349342	-0.646191
H	4.957297	0.763786	0.242743
H	4.666286	-1.647379	-0.321617
H	2.400755	-2.559791	-0.704669
C	-3.402048	-0.863243	-0.683722
C	-3.497254	0.552342	-0.822519
H	-4.277322	-1.471908	-0.879881
H	-4.437969	1.007692	-1.102199
H	0.127548	-2.247882	-0.656992
H	-2.145841	-2.535675	-0.246505
H	-2.430770	2.419598	-0.811187
H	2.972327	2.229856	0.383924
O	-0.721506	-0.847204	1.445156
O	-1.775056	-0.296281	2.216060
H	-1.454354	0.595205	2.424337

Solvent = Pentyl Ethanoate
E = -1066.467112
G = -1066.311130
ZPC = 0.196165
TCE = 0.209336
TCH = 0.210280
TCG = 0.155982
N_{imag} = 0

S	0.231662	1.796115	0.023527
N	0.134003	-1.268519	-0.516572
C	-1.027115	-0.675218	0.073709
C	-1.143495	0.777034	-0.306707
C	3.979632	0.352083	-0.015891
C	3.806806	-1.001904	-0.301821
C	2.533952	-1.524898	-0.457460
C	1.399653	-0.712542	-0.335999
C	1.581128	0.651394	-0.070656
C	2.866341	1.170263	0.096619
C	-2.251032	-1.462074	-0.277096
C	-2.315711	1.319364	-0.778231
H	4.971246	0.767225	0.114551
H	4.666718	-1.654487	-0.396065
H	2.393793	-2.580329	-0.667278
C	-3.380834	-0.879925	-0.737142
C	-3.445052	0.521466	-0.982132
H	-4.254025	-1.490522	-0.937741
H	-4.357875	0.965330	-1.357176
H	0.115049	-2.280750	-0.545030
H	-2.190689	-2.530261	-0.093541
H	-2.355260	2.377623	-1.014255
H	2.987421	2.227838	0.307254
O	-0.772957	-0.786620	1.516915
O	-1.823255	-0.163525	2.234588
H	-1.476942	0.731027	2.373681

PS-OOH• - Site 2
Solvent = Water
E = -1066.466902
G = -1066.310561
ZPC = 0.197110
TCE = 0.210263
TCH = 0.211207
TCG = 0.156341
N_{imag} = 0

S	0.649307	1.778486	0.704839
N	0.184675	-1.141396	0.023434
C	-0.875325	-0.305247	-0.222974
C	-0.796758	1.070267	-0.060606
C	4.195138	0.060733	-0.208455
C	3.847691	-1.273126	-0.404013
C	2.517346	-1.666931	-0.333441
C	1.523237	-0.733345	-0.039698
C	1.874031	0.603854	0.176012
C	3.204638	0.997614	0.071029
C	-2.155924	-0.971423	-0.632184
C	-1.861340	1.898313	-0.399826
H	5.229626	0.374617	-0.272157
H	4.610798	-2.010181	-0.622743
H	2.233439	-2.700291	-0.501563
C	-3.220633	-0.011016	-1.036881
C	-3.068923	1.326834	-0.902177
H	-4.137972	-0.440422	-1.422469
H	-3.874104	1.993347	-1.190346
H	0.022800	-2.124953	-0.166018
H	-1.959958	-1.712406	-1.418057
H	-1.774513	2.970970	-0.281323
H	3.465303	2.038620	0.226223
O	-2.616110	-1.864587	0.428363
O	-2.942001	-1.097100	1.578781
H	-2.131231	-1.140958	2.109129

Solvent = Pentyl Ethanoate
E = -1066.468533
G = -1066.312966
ZPC = 0.196598
TCE = 0.209899
TCH = 0.210843
TCG = 0.155568
N_{imag} = 0

S	0.666670	1.802545	0.709765
N	0.155286	-1.108853	0.058317
C	-0.889329	-0.252607	-0.214651
C	-0.795412	1.114873	-0.043307
C	4.179442	0.035370	-0.240127
C	3.814054	-1.295433	-0.416478
C	2.479938	-1.672370	-0.324362
C	1.501144	-0.724696	-0.025775
C	1.870630	0.610428	0.171658
C	3.203793	0.986415	0.042807
C	-2.162612	-0.910348	-0.666834
C	-1.866014	1.954483	-0.348712
H	5.216691	0.336166	-0.322017
H	4.565413	-2.043825	-0.638158
H	2.185641	-2.705166	-0.480340
C	-3.262791	0.057597	-0.938724
C	-3.099569	1.392456	-0.789896
H	-4.206396	-0.364176	-1.264997
H	-3.923667	2.062810	-1.007161
H	-0.023964	-2.089312	-0.125892
H	-1.962436	-1.537307	-1.549364
H	-1.766828	3.025405	-0.224472
H	3.477732	2.026430	0.181492
O	-2.545778	-1.941333	0.279418
O	-2.849587	-1.336553	1.527037
H	-1.992619	-1.343698	1.981217

PS-OOH• - Site 3
Solvent = Water
E = -1066.464966
G = -1066.309066
ZPC = 0.196894
TCE = 0.210121
TCH = 0.211065
TCG = 0.155900
N_{imag} = 0

S	0.744439	1.749841	0.456826
N	0.449795	-1.265250	0.284780
C	-0.681382	-0.555532	-0.138595
C	-0.645483	0.879719	-0.131380
C	4.387654	0.127243	-0.149699
C	4.110941	-1.238138	-0.187653
C	2.805497	-1.694845	-0.070995
C	1.751397	-0.794021	0.107565
C	2.036934	0.574487	0.151599
C	3.345059	1.031775	0.012257
C	-1.808264	-1.207201	-0.527116
C	-1.788408	1.614847	-0.541596
H	5.403886	0.487241	-0.249846
H	4.914419	-1.953345	-0.317557
H	2.582734	-2.755998	-0.108624
C	-3.079746	-0.493612	-0.824457
C	-2.937986	0.989938	-0.895710
H	-3.547933	-0.884126	-1.735489
H	-3.802644	1.560978	-1.212995
H	0.358526	-2.269715	0.182722
H	-1.815598	-2.292058	-0.561698
H	-1.723551	2.697249	-0.578487
H	3.543424	2.097629	0.038421
O	-4.117831	-0.856243	0.145938
O	-3.734284	-0.410007	1.437712
H	-3.133215	-1.106520	1.746341

Solvent = Pentyl Ethanoate
E = -1066.466477
G = -1066.310518
ZPC = 0.196905
TCE = 0.210154
TCH = 0.211098
TCG = 0.155959
N_{imag} = 0

S	0.756362	1.750883	0.440763
N	0.459580	-1.265622	0.251742
C	-0.678526	-0.554649	-0.142776
C	-0.637033	0.881317	-0.140270
C	4.402358	0.123124	-0.129958
C	4.125856	-1.241503	-0.168041
C	2.818960	-1.696640	-0.066510
C	1.762330	-0.795662	0.097629
C	2.047958	0.573242	0.144495
C	3.358071	1.027427	0.018954
C	-1.815901	-1.202891	-0.506624
C	-1.785895	1.616870	-0.530813
H	5.419827	0.482754	-0.219515
H	4.930447	-1.957543	-0.287264
H	2.599447	-2.758571	-0.105845
C	-3.089675	-0.488631	-0.807297
C	-2.942987	0.996067	-0.864776

H	-3.533037	-0.865117	-1.738132
H	-3.814041	1.571306	-1.156380
H	0.365345	-2.270844	0.178408
H	-1.825569	-2.288114	-0.547215
H	-1.720932	2.699591	-0.561004
H	3.556302	2.093415	0.045331
O	-4.139915	-0.868789	0.127629
O	-3.800780	-0.408351	1.425298
H	-3.164176	-1.072970	1.729712

PS-OOH• - Site 4
Solvent = Water
E = -1066.467936
G = -1066.311543
ZPC = 0.197163
TCE = 0.210366
TCH = 0.211310
TCG = 0.156393
N_{imag} = 0

S	-0.513448	-1.679706	0.176511
N	-0.656024	1.363412	0.391978
C	0.557855	0.870962	-0.026945
C	0.764726	-0.523485	-0.254485
C	-4.360141	-0.512792	-0.173030
C	-4.287493	0.865177	0.022955
C	-3.057555	1.481205	0.202796
C	-1.882249	0.724448	0.208501
C	-1.957140	-0.661425	0.026823
C	-3.193911	-1.269354	-0.177436
C	1.625936	1.771980	-0.292701
C	1.952338	-0.996107	-0.708615
H	-5.316581	-0.997685	-0.323947
H	-5.189486	1.465108	0.025312
H	-2.987979	2.555004	0.341262
C	2.836011	1.334804	-0.718142
C	3.148865	-0.117876	-0.841626
H	3.627498	2.039670	-0.944836
H	3.704284	-0.334649	-1.760457
H	-0.707651	2.372102	0.485147
H	1.431685	2.834488	-0.179842
H	2.083706	-2.054340	-0.906040
H	-3.240336	-2.342039	-0.329813
O	4.175458	-0.498826	0.142401
O	3.661792	-0.328883	1.454920
H	3.820746	0.610209	1.638043

Solvent = Pentyl Ethanoate
E = -1066.468634
G = -1066.312494
ZPC = 0.196999
TCE = 0.210253
TCH = 0.211197
TCG = 0.156140
N_{imag} = 0

S	-0.513795	-1.672705	0.185500
N	-0.673221	1.368569	0.388815
C	0.549422	0.881704	-0.013556
C	0.760857	-0.512778	-0.246580
C	-4.365463	-0.527098	-0.180653
C	-4.302309	0.850073	0.018376
C	-3.076245	1.473111	0.199705
C	-1.895746	0.724574	0.206323
C	-1.961252	-0.662406	0.024570
C	-3.194235	-1.275507	-0.183792
C	1.620059	1.783529	-0.257377
C	1.950038	-0.979453	-0.696180
H	-5.318661	-1.017736	-0.334367
H	-5.208315	1.444224	0.021111
H	-3.017720	2.547849	0.338998
C	2.833592	1.351777	-0.681440
C	3.147221	-0.099985	-0.839130
H	3.625980	2.061126	-0.893044
H	3.665750	-0.291896	-1.786309
H	-0.727463	2.373102	0.505502
H	1.430276	2.845593	-0.130060
H	2.086248	-2.038545	-0.886030
H	-3.232695	-2.348234	-0.338661
O	4.200474	-0.498162	0.091383
O	3.722675	-0.377129	1.421329
H	3.849058	0.564898	1.609857

PS-OOH• - Site 5
Solvent = Water
E = -1066.464052
G = -1066.307780
ZPC = 0.196864
TCE = 0.209947
TCH = 0.210891
TCG = 0.156272
N_{imag} = 0

S	-0.166063	-1.529067	0.171733
N	-0.661620	1.363372	0.805631
C	0.581253	1.096006	0.197801
C	0.917651	-0.183087	-0.163006
C	-4.080950	-0.716002	-0.483166
C	-4.172642	0.622176	-0.110478
C	-3.040060	1.319254	0.294088
C	-1.803577	0.675982	0.362010
C	-1.718975	-0.671915	0.000057
C	-2.846654	-1.358017	-0.439296
C	1.489118	2.155555	-0.014465
C	2.236991	-0.524524	-0.778083
H	-4.959928	-1.258700	-0.808148
H	-5.127478	1.133157	-0.144635
H	-3.103131	2.366627	0.569708
C	2.764855	1.901307	-0.578491
C	3.140132	0.652243	-0.951327
H	3.444418	2.734111	-0.718447
H	4.115337	0.456593	-1.382585
H	-0.828847	2.354720	0.938868
H	1.204517	3.160443	0.274429
H	2.087603	-1.041196	-1.736593
H	-2.759507	-2.398561	-0.731780
O	2.892029	-1.581625	-0.027468
O	3.153953	-1.124936	1.291895
H	4.044653	-0.747108	1.222759

Solvent = Pentyl Ethanoate
E = -1066.464835
G = -1066.309323
ZPC = 0.196333
TCE = 0.209609
TCH = 0.210553

TCG = 0.155512
N_{imag} = 0

S	-0.162816	-1.516924	0.146143
N	-0.663112	1.384547	0.754591
C	0.587996	1.110437	0.175193
C	0.923665	-0.176385	-0.175746
C	-4.087842	-0.724610	-0.456952
C	-4.183874	0.613237	-0.087619
C	-3.049700	1.320659	0.293295
C	-1.806241	0.688619	0.341518
C	-1.717449	-0.661569	-0.013697
C	-2.847701	-1.356063	-0.430355
C	1.506683	2.158180	-0.017239
C	2.253049	-0.527996	-0.767083
H	-4.967930	-1.275835	-0.764494
H	-5.143541	1.116257	-0.105745
H	-3.119061	2.368617	0.566367
C	2.796356	1.893106	-0.549384
C	3.170312	0.643260	-0.914902
H	3.485139	2.721415	-0.673086
H	4.151935	0.443281	-1.330791
H	-0.823979	2.369282	0.925737
H	1.226378	3.167276	0.261692
H	2.110018	-1.028060	-1.736179
H	-2.756851	-2.397686	-0.718477
O	2.883415	-1.596820	-0.022158
O	3.099233	-1.163662	1.312080
H	3.952023	-0.706985	1.253005

PS-OOH• - Site 5a
Solvent = Water
E = -1066.471860
G = -1066.315475
ZPC = 0.196392
TCE = 0.209560
TCH = 0.210504
TCG = 0.156385
N_{imag} = 0

S	-0.125722	-1.578956	-0.741421
N	-0.208422	1.444308	-0.293323
C	1.032133	0.868768	-0.332378
C	1.139926	-0.551600	0.117990
C	-3.935558	-0.444866	-0.011873
C	-3.821205	0.935872	0.153960
C	-2.580517	1.547499	0.081348
C	-1.430054	0.791511	-0.176961
C	-1.546457	-0.595996	-0.347017
C	-2.797725	-1.204396	-0.251466
C	2.153870	1.596562	-0.703974
C	2.490046	-1.139573	-0.077195
H	-4.902140	-0.928640	0.053519
H	-4.701374	1.536927	0.348022
H	-2.479737	2.619387	0.214889
C	3.405866	0.999700	-0.765229
C	3.547786	-0.386085	-0.466223
H	4.269002	1.579526	-1.064336
H	4.524140	-0.848679	-0.557025
H	-0.249808	2.449408	-0.423331
H	2.024147	2.640522	-0.970665
H	2.595418	-2.192557	0.159863
H	-2.874693	-2.279335	-0.371258
O	0.705211	-0.684998	1.507916
O	1.574209	0.080777	2.333880
H	1.081537	0.905981	2.466044

Solvent = Pentyl Ethanoate
E = -1066.472810
G = -1066.316547
ZPC = 0.196296
TCE = 0.209478
TCH = 0.210422
TCG = 0.156263
N_{imag} = 0

S	-0.102509	-1.593238	-0.698083
N	-0.198126	1.441684	-0.252218
C	1.041110	0.858425	-0.334334
C	1.150154	-0.545759	0.166168
C	-3.927986	-0.458626	-0.063268
C	-3.821930	0.921194	0.106848
C	-2.580627	1.534499	0.064077
C	-1.422747	0.783038	-0.170385
C	-1.529842	-0.605080	-0.343013
C	-2.782524	-1.213967	-0.275732
C	2.147566	1.558581	-0.784311
C	2.504603	-1.133337	0.000740
H	-4.894407	-0.945514	-0.020031
H	-4.707862	1.520000	0.281531
H	-2.488915	2.606745	0.204846
C	3.398595	0.954989	-0.848140
C	3.551022	-0.406142	-0.459975
H	4.251875	1.513667	-1.209715
H	4.528474	-0.869693	-0.533324
H	-0.242836	2.437209	-0.433605
H	2.014516	2.585730	-1.109406
H	2.618631	-2.165479	0.312991
H	-2.852971	-2.289641	-0.393873
O	0.688976	-0.642610	1.539351
O	1.495233	0.205825	2.347546
H	0.962497	1.014396	2.388215

TS[PS-OOH•] - Site 2a
Solvent = Water
E = -1066.453691
G = -1066.299008
ZPC = 0.195045
TCE = 0.208180
TCH = 0.209124
TCG = 0.154683
N_{imag} = 1, -468.72 cm^{-1}

S	0.271582	1.858464	-0.238834
N	0.208058	-1.149753	-0.873266
C	-0.994543	-0.609129	-0.441877
C	-1.132542	0.819684	-0.323356
C	3.951156	0.335647	0.316178
C	3.800003	-1.003530	-0.036862
C	2.556818	-1.492237	-0.413454
C	1.445953	-0.647566	-0.457697
C	1.604481	0.698229	-0.115355
C	2.850002	1.181946	0.280141
C	-2.186259	-1.365736	-0.717094
C	-2.393044	1.389415	-0.232221
H	4.916463	0.720255	0.620450

H	4.650376	-1.674244	-0.011572
H	2.428929	-2.536678	-0.677841
C	-3.416903	-0.779361	-0.635709
C	-3.532305	0.603227	-0.373743
H	-4.309443	-1.372865	-0.791243
H	-4.510314	1.062643	-0.306258
H	0.186920	-2.155392	-1.004639
H	-2.070739	-2.422677	-0.931118
H	-2.488851	2.458253	-0.076290
H	2.954464	2.226369	0.553433
O	-0.784510	-1.016956	1.422243
O	-1.863635	-0.619345	2.210153
H	-1.691254	0.314034	2.406408

Solvent = Pentyl Ethanoate
E = -1066.451866
G = -1066.297907
ZPC = 0.194731
TCE = 0.207993
TCH = 0.208937
TCG = 0.153959
N_{imag} = 1, -597.63 cm^{-1}

S	0.279797	1.863963	-0.233683
N	0.209843	-1.140233	-0.874136
C	-0.997807	-0.602665	-0.427318
C	-1.131864	0.827510	-0.309822
C	3.957305	0.330063	0.322929
C	3.803522	-1.006124	-0.035137
C	2.558202	-1.490291	-0.411670
C	1.447981	-0.645081	-0.454591
C	1.610469	0.699340	-0.107150
C	2.856132	1.177324	0.290521
C	-2.191580	-1.346483	-0.728724
C	-2.388508	1.397016	-0.201952
H	4.923218	0.712139	0.629060
H	4.652358	-1.679250	-0.012332
H	2.429750	-2.534627	-0.677333
C	-3.421927	-0.759742	-0.632960
C	-3.533781	0.614714	-0.344949
H	-4.315697	-1.350380	-0.793901
H	-4.510135	1.075673	-0.262206
H	0.186572	-2.144450	-1.003118
H	-2.081969	-2.401128	-0.957263
H	-2.480061	2.465324	-0.036919
H	2.961783	2.220408	0.569146
O	-0.799586	-1.041081	1.378155
O	-1.877762	-0.664412	2.156822
H	-1.701563	0.264976	2.367871

TS[PS-OOH•] - Site 2
Solvent = Water
E = -1066.452890
G = -1066.298053
ZPC = 0.195689
TCE = 0.208852
TCH = 0.209796
TCG = 0.154837
N_{imag} = 1, -517.41 cm^{-1}

S	0.557020	1.727456	0.648566
N	0.246206	-1.215044	-0.031198
C	-0.840650	-0.436554	-0.334695
C	-0.816823	0.958082	-0.178037
C	4.205810	0.164569	-0.120855
C	3.923340	-1.182143	-0.331737
C	2.609760	-1.633157	-0.309259
C	1.566442	-0.744273	-0.049909
C	1.851831	0.605561	0.178496
C	3.166766	1.057401	0.123466
C	-2.058153	-1.076415	-0.731765
C	-1.902259	1.718785	-0.571796
H	5.227404	0.522754	-0.147152
H	4.724639	-1.884802	-0.525560
H	2.376002	-2.676938	-0.490231
C	-3.116298	-0.260556	-1.233784
C	-3.052895	1.101166	-1.115963
H	-3.988858	-0.746806	-1.651852
H	-3.873921	1.722778	-1.451808
H	0.139294	-2.205104	-0.225714
H	-1.985256	-2.107195	-1.063659
H	-1.868950	2.796905	-0.464796
H	3.375776	2.108535	0.288047
O	-2.695147	-1.671556	0.947041
O	-3.028458	-0.576747	1.737182
H	-2.194289	-0.312515	2.155299

Solvent = Pentyl Ethanoate
E = -1066.452704
G = -1066.298457
ZPC = 0.195369
TCE = 0.208614
TCH = 0.209558
TCG = 0.154247
N_{imag} = 1, -601.96 cm^{-1}

S	0.558587	1.722845	0.681226
N	0.229652	-1.199329	-0.015852
C	-0.851810	-0.408591	-0.340403
C	-0.823523	0.976261	-0.156071
C	4.195736	0.159315	-0.151035
C	3.906688	-1.185335	-0.360441
C	2.591620	-1.632105	-0.326437
C	1.553121	-0.741922	-0.052458
C	1.846759	0.606454	0.178356
C	3.161632	1.054156	0.106790
C	-2.068946	-1.040821	-0.752788
C	-1.911673	1.752491	-0.526784
H	5.218303	0.514191	-0.187898
H	4.703895	-1.890442	-0.563299
H	2.357251	-2.675722	-0.508397
C	-3.131938	-0.208650	-1.219515
C	-3.066905	1.150686	-1.068118
H	-4.011108	-0.683143	-1.637319
H	-3.894473	1.778587	-1.375474
H	0.111232	-2.189985	-0.190600
H	-1.991004	-2.050345	-1.145036
H	-1.870667	2.827747	-0.395941
H	3.374775	2.104583	0.271088
O	-2.676144	-1.724572	0.862460
O	-2.933617	-0.688676	1.740449
H	-2.069640	-0.491328	2.134370

TS[PS-OOH•] - Site 3
Solvent = Water
E = -1066.450539

G = -1066.295708
ZPC = 0.195487
TCE = 0.208562
TCH = 0.209507
TCG = 0.154831
N_{imag} = 1, -576.82 cm^{-1}

S	0.619032	1.723087	0.435081
N	0.425508	-1.293844	0.233812
C	-0.699608	-0.611508	-0.233554
C	-0.721487	0.810324	-0.227894
C	4.327533	0.222230	-0.101870
C	4.097623	-1.151060	-0.141799
C	2.804948	-1.650770	-0.054865
C	1.720591	-0.784026	0.098937
C	1.958455	0.592542	0.148961
C	3.252262	1.093149	0.033994
C	-1.806017	-1.298063	-0.677998
C	-1.843506	1.504561	-0.709442
H	5.333507	0.615181	-0.179248
H	4.927006	-1.839600	-0.250924
H	2.617800	-2.718578	-0.097658
C	-3.011158	-0.604272	-0.982985
C	-2.959670	0.818595	-1.119760
H	-3.783473	-1.142979	-1.519251
H	-3.827438	1.350170	-1.489172
H	0.365682	-2.300190	0.124623
H	-1.787119	-2.382135	-0.701045
H	-1.813825	2.587224	-0.758221
H	3.414811	2.164960	0.061860
O	-4.021701	-0.735862	0.610825
O	-3.276764	-0.211173	1.654383
H	-2.662097	-0.920472	1.899043

Solvent = Pentyl Ethanoate
E = -1066.450457
G = -1066.295640
ZPC = 0.195492
TCE = 0.208586
TCH = 0.209530
TCG = 0.154817
N_{imag} = 1, -633.43 cm^{-1}

S	0.615426	1.718752	0.445846
N	0.434585	-1.290967	0.270759
C	-0.697330	-0.621503	-0.206146
C	-0.719915	0.797985	-0.228642
C	4.327824	0.229273	-0.126975
C	4.103892	-1.144476	-0.144034
C	2.813757	-1.647251	-0.036908
C	1.727932	-0.782809	0.118069
C	1.959712	0.596147	0.146580
C	3.250041	1.097622	0.007668
C	-1.801981	-1.318703	-0.639367
C	-1.833531	1.478693	-0.742999
H	5.331436	0.625100	-0.221017
H	4.935079	-1.831263	-0.252173
H	2.632904	-2.716878	-0.064088
C	-3.009614	-0.631563	-0.964153
C	-2.947289	0.784929	-1.148141
H	-3.770387	-1.183409	-1.504346
H	-3.811310	1.309249	-1.536273
H	0.374695	-2.299191	0.199911
H	-1.784621	-2.403593	-0.642776
H	-1.801972	2.560115	-0.815462
H	3.408415	2.170440	0.017462
O	-4.019506	-0.733944	0.592035
O	-3.324874	-0.130975	1.617515
H	-2.689096	-0.804553	1.903803

TS[PS-OOH•] - Site 4
Solvent = Water
E = -1066.454802
G = -1066.300125
ZPC = 0.195670
TCE = 0.208863
TCH = 0.209807
TCG = 0.154677
N_{imag} = 1, -462.16 cm^{-1}

S	-0.479228	-1.693972	0.101447
N	-0.567213	1.349249	0.328081
C	0.615407	0.843109	-0.141155
C	0.786856	-0.546292	-0.372938
C	-4.320335	-0.469403	-0.093416
C	-4.218581	0.906655	0.095000
C	-2.972070	1.503310	0.226331
C	-1.812800	0.726181	0.191895
C	-1.915902	-0.656936	0.015483
C	-3.167606	-1.245988	-0.140966
C	1.683122	1.724481	-0.430742
C	1.973297	-1.025465	-0.867355
H	-5.289528	-0.939254	-0.205410
H	-5.109972	1.521070	0.129993
H	-2.879831	2.576170	0.358555
C	2.876906	1.244070	-0.895845
C	3.098551	-0.162512	-1.004920
H	3.683899	1.927265	-1.130542
H	3.945324	-0.513059	-1.582154
H	-0.600808	2.358441	0.427123
H	1.518933	2.789548	-0.304464
H	2.090008	-2.084496	-1.066904
H	-3.237400	-2.317900	-0.288901
O	4.073486	-0.489060	0.617660
O	3.234177	-0.195082	1.690080
H	3.295130	0.766903	1.792832

Solvent = Pentyl Ethanoate
E = -1066.453330
G = -1066.298573
ZPC = 0.195572
TCE = 0.208718
TCH = 0.209662
TCG = 0.154756
N_{imag} = 1, -581.88 cm^{-1}

S	-0.472689	-1.687854	0.162182
N	-0.574848	1.335957	0.413283
C	0.611074	0.845606	-0.088428
C	0.783731	-0.537523	-0.336752
C	-4.313366	-0.474007	-0.166365
C	-4.220848	0.897583	0.049868
C	-2.980071	1.493954	0.232100
C	-1.818078	0.720833	0.226285
C	-1.913295	-0.660521	0.026451
C	-3.157130	-1.246956	-0.186831

C	1.663787	1.733794	-0.397673
C	1.956985	-1.007254	-0.869998
H	-5.277421	-0.942916	-0.320410
H	-5.114369	1.510193	0.065537
H	-2.899112	2.565548	0.383704
C	2.849684	1.264354	-0.900797
C	3.079332	-0.140323	-1.022838
H	3.645721	1.954844	-1.152333
H	3.902805	-0.477541	-1.641003
H	-0.611881	2.341350	0.528995
H	1.500134	2.798469	-0.263234
H	2.071469	-2.064281	-1.080451
H	-3.217600	-2.316265	-0.356713
O	4.084631	-0.485439	0.524269
O	3.287189	-0.236187	1.625519
H	3.361815	0.720324	1.759578

TS[PS-OOH$^•$] - Site 5
Solvent = Water
E = -1066.449116
G = -1066.294123
ZPC = 0.195490
TCE = 0.208539
TCH = 0.209483
TCG = 0.154994
N_{imag} = 1, -593.74 cm^{-1}

S	-0.247710	-1.602521	0.033446
N	-0.554854	1.301868	0.743963
C	0.642026	0.984629	0.081296
C	0.892300	-0.312394	-0.340564
C	-4.143129	-0.576061	-0.379973
C	-4.143378	0.758430	0.015891
C	-2.954619	1.388998	0.364911
C	-1.752325	0.681376	0.352674
C	-1.758305	-0.662004	-0.035118
C	-2.943769	-1.281446	-0.417070
C	1.608181	1.975033	-0.139226
C	2.159401	-0.663739	-0.898326
H	-5.066060	-1.067537	-0.661629
H	-5.070357	1.318652	0.043837
H	-2.945726	2.432982	0.659552
C	2.812159	1.671037	-0.797595
C	3.070030	0.393564	-1.225972
H	3.522648	2.466773	-0.986153
H	3.986969	0.146782	-1.746122
H	-0.662542	2.295770	0.914779
H	1.406237	2.987256	0.194405
H	2.223859	-1.590153	-1.459139
H	-2.928543	-2.319570	-0.729440
O	3.020302	-1.458245	0.537500
O	3.056095	-0.574766	1.600502
H	3.838247	-0.024143	1.437444

Solvent = Pentyl Ethanoate
E = -1066.449282
G = -1066.294420
ZPC = 0.195381
TCE = 0.208464
TCH = 0.209408
TCG = 0.154862
N_{imag} = 1, -629.47 cm^{-1}

S	-0.244567	-1.592392	0.016457
N	-0.557803	1.320110	0.700646
C	0.650090	0.996053	0.067690
C	0.899293	-0.305751	-0.348233
C	-4.146990	-0.584177	-0.363206
C	-4.151729	0.749527	0.032046
C	-2.962511	1.388589	0.362802
C	-1.754257	0.691038	0.334485
C	-1.755875	-0.654448	-0.049493
C	-2.942700	-1.280054	-0.414629
C	1.625076	1.977897	-0.135906
C	2.174464	-0.667367	-0.881038
H	-5.070074	-1.082769	-0.632044
H	-5.082493	1.302941	0.073727
H	-2.960910	2.433283	0.656349
C	2.838796	1.663923	-0.774511
C	3.094541	0.386083	-1.200445
H	3.556332	2.455757	-0.955762
H	4.016618	0.134428	-1.709691
H	-0.660450	2.305622	0.908042
H	1.426914	2.993313	0.190647
H	2.233845	-1.589421	-1.450094
H	-2.923698	-2.318947	-0.724794
O	2.992335	-1.470202	0.546548
O	3.017345	-0.595281	1.610437
H	3.794983	-0.040704	1.442519

TS[PS-OOH$^•$] - Site 5a
Solvent = Water
E = -1066.455967
G = -1066.300969
ZPC = 0.195186
TCE = 0.208373
TCH = 0.209317
TCG = 0.154998
N_{imag} = 1, -411.55 cm^{-1}

S	-0.222192	-1.659592	-0.694172
N	-0.228283	1.401199	-0.539215
C	1.003275	0.816798	-0.487776
C	1.130630	-0.594027	-0.256672
C	-3.944441	-0.384785	0.195956
C	-3.809597	1.002056	0.198185
C	-2.571768	1.583011	-0.033258
C	-1.451791	0.785046	-0.284387
C	-1.589666	-0.607622	-0.290614
C	-2.832488	-1.184467	-0.041728
C	2.158548	1.605385	-0.583012
C	2.430084	-1.177810	-0.346463
H	-4.906929	-0.844346	0.383068
H	-4.668371	1.634497	0.387442
H	-2.453327	2.661317	-0.026183
C	3.406438	1.019084	-0.549017
C	3.541576	-0.384865	-0.437804
H	4.290857	1.639448	-0.625907
H	4.527263	-0.833369	-0.450777
H	-0.243768	2.413869	-0.594082
H	2.047182	2.677892	-0.700096
H	2.515911	-2.256547	-0.282120
H	-2.927298	-2.264653	-0.039034
O	0.803541	-0.615561	1.664254
O	1.782383	0.117929	2.337063
H	1.459332	1.031192	2.317952

Solvent = Pentyl Ethanoate
E = -1066.453423
G = -1066.298839
ZPC = 0.194979
TCE = 0.208220
TCH = 0.209164
TCG = 0.154584
N_{imag} = 1, -561.14 cm^{-1}

S	-0.221746	-1.634733	-0.744721
N	-0.237223	1.412151	-0.539349
C	1.003963	0.832206	-0.476621
C	1.131198	-0.580834	-0.256941
C	-3.941188	-0.393790	0.218032
C	-3.813923	0.992092	0.228954
C	-2.579825	1.581234	-0.008543
C	-1.458598	0.793047	-0.279938
C	-1.590346	-0.600508	-0.299299
C	-2.825664	-1.184298	-0.037323
C	2.154104	1.621503	-0.541864
C	2.431605	-1.159689	-0.352620
H	-4.898864	-0.860505	0.412911
H	-4.673953	1.618875	0.432380
H	-2.471189	2.660806	0.011042
C	3.408861	1.036345	-0.503775
C	3.545047	-0.364122	-0.419618
H	4.291352	1.662255	-0.558889
H	4.531199	-0.812056	-0.427415
H	-0.256204	2.422456	-0.595450
H	2.046848	2.696259	-0.647391
H	2.516783	-2.238991	-0.299705
H	-2.911921	-2.265286	-0.038972
O	0.802013	-0.649235	1.620810
O	1.791932	0.024542	2.311546
H	1.491485	0.945288	2.320963

PS-OOCH$_3^•$ - Site 2a
Solvent = Water
E = -1105.754975
G = -1105.572202
ZPC = 0.224826
TCE = 0.239233
TCH = 0.240177
TCG = 0.182773
N_{imag} = 0

S	-0.322316	-1.610759	-0.873660
N	-0.299997	1.445007	-0.483546
C	0.894494	0.772222	-0.057678
C	1.059958	-0.557839	-0.737833
C	-4.034949	-0.407731	0.069631
C	-3.908681	0.977142	0.176049
C	-2.671306	1.580631	0.009439
C	-1.536586	0.816923	-0.280065
C	-1.672059	-0.570492	-0.402573
C	-2.915894	-1.176568	-0.215790
C	2.086329	1.659899	-0.234318
C	2.286686	-0.988599	-1.196745
H	-4.996014	-0.886537	0.209680
H	-4.774615	1.588456	0.400208
H	-2.561659	2.655782	0.105422
C	3.263511	1.197579	-0.710371
C	3.399641	-0.147576	-1.164337
H	4.117404	1.862889	-0.767831
H	4.353757	-0.502206	-1.531057
H	-0.304097	2.423515	-0.210912
H	1.962276	2.684029	0.102998
H	2.377718	-1.982661	-1.622470
H	-3.003091	-2.254207	-0.304386
O	0.678011	0.559371	1.389651
O	1.829566	-0.068422	1.943877
C	1.416537	-1.318021	2.482984
H	2.311511	-1.724312	2.955813
H	0.635905	-1.170809	3.232068
H	1.071226	-1.989860	1.695269

Solvent = Pentyl Ethanoate
E = -1105.757786
G = -1105.575529
ZPC = 0.224546
TCE = 0.239048
TCH = 0.239992
TCG = 0.182257
N_{imag} = 0

S	-0.327112	-1.544679	-0.934036
N	-0.301023	1.470018	-0.244915
C	0.904398	0.755319	0.065115
C	1.042262	-0.475721	-0.791990
C	-4.061967	-0.391645	-0.018913
C	-3.930751	0.975310	0.222432
C	-2.684861	1.578577	0.158505
C	-1.541236	0.836320	-0.157748
C	-1.682772	-0.532051	-0.420473
C	-2.937569	-1.137948	-0.337942
C	2.087688	1.667513	-0.053109
C	2.232158	-0.810390	-1.399061
H	-5.030542	-0.872389	0.039723
H	-4.800443	1.571829	0.471541
H	-2.576178	2.639267	0.359766
C	3.229596	1.297459	-0.672514
C	3.338122	0.038671	-1.331007
H	4.076718	1.974181	-0.684137
H	4.262887	-0.240833	-1.818725
H	-0.300446	2.425033	0.094066
H	1.992628	2.624705	0.449881
H	2.297680	-1.731485	-1.969224
H	-3.027785	-2.200867	-0.536411
O	0.733101	0.374010	1.469313
O	1.909981	-0.300882	1.902391
C	1.506588	-1.576432	2.368560
H	2.419400	-2.034793	2.753423
H	0.775469	-1.480648	3.175505
H	1.096965	-2.184174	1.558803

PS-OOCH$_3^•$ - Site 2
Solvent = Water
E = -1105.755628
G = -1105.573137
ZPC = 0.225235
TCE = 0.239794
TCH = 0.240738
TCG = 0.182491
N_{imag} = 0

S	0.783362	1.651685	0.921859
N	0.317038	-1.064104	-0.358249
C	-0.711631	-0.168608	-0.488776
C	-0.613235	1.152356	-0.068468
C	4.354490	0.025684	-0.061570
C	3.997601	-1.229870	-0.545714
C	2.658552	-1.584713	-0.648102
C	1.662571	-0.695232	-0.242932
C	2.021394	0.559988	0.261179
C	3.363650	0.920841	0.330897
C	-1.991016	-0.705493	-1.056993
C	-1.617749	2.070259	-0.349339
H	5.396606	0.311025	0.011572
H	4.760617	-1.933449	-0.855917
H	2.368040	-2.553223	-1.041070
C	-2.952064	0.364810	-1.450543
C	-2.773046	1.653296	-1.078582
H	-3.829109	0.052310	-2.005130
H	-3.511275	2.401214	-1.345677
H	0.152232	-1.990993	-0.735668
H	-1.776920	-1.385237	-1.890326
H	-1.516406	3.100835	-0.033432
H	3.632069	1.900607	0.709821
O	-2.605322	-1.647449	-0.121489
O	-3.040025	-0.929609	1.031518
C	-2.244339	-1.352574	2.132102
H	-2.678492	-0.847893	2.996222
H	-2.315152	-2.434332	2.261426
H	-1.203569	-1.047085	2.006969

Solvent = Pentyl Ethanoate
E = -1105.760398
G = -1105.578234
ZPC = 0.225030
TCE = 0.239619
TCH = 0.240563
TCG = 0.182164
N_{imag} = 0

S	0.847375	1.663494	0.987735
N	0.264941	-0.998276	-0.327076
C	-0.730546	-0.056999	-0.445845
C	-0.583059	1.241122	0.009596
C	4.342951	-0.047755	-0.120017
C	3.935218	-1.285087	-0.608477
C	2.583244	-1.596228	-0.683201
C	1.625402	-0.682330	-0.242894
C	2.036622	0.553757	0.269774
C	3.390085	0.872284	0.307478
C	-2.008964	-0.534049	-1.076260
C	-1.571851	2.197495	-0.209209
H	5.395169	0.204092	-0.070059
H	4.668149	-2.008646	-0.944924
H	2.256514	-2.551127	-1.081759
C	-2.999443	0.560258	-1.294680
C	-2.776478	1.827332	-0.877078
H	-3.922817	0.282699	-1.789911
H	-3.527156	2.590878	-1.048429
H	0.050304	-1.919013	-0.690546
H	-1.779758	-1.047958	-2.021589
H	-1.428753	3.213740	0.135449
H	3.696793	1.839040	0.691062
O	-2.569247	-1.639388	-0.324839
O	-3.022209	-1.145584	0.933400
C	-2.231831	-1.757841	1.938259
H	-2.670686	-1.422572	2.879777
H	-2.297122	-2.846995	1.871731
H	-1.189683	-1.434083	1.881811

PS-OOCH$_3^•$ - Site 3
Solvent = Water
E = -1105.753547
G = -1105.571312
ZPC = 0.225245
TCE = 0.239767
TCH = 0.240711
TCG = 0.182235
N_{imag} = 0

S	1.025912	1.676870	0.738286
N	0.587478	-1.215857	-0.058165
C	-0.469177	-0.360735	-0.405424
C	-0.359150	1.037595	-0.105299
C	4.614063	-0.023280	0.018368
C	4.269923	-1.332608	-0.312171
C	2.937364	-1.717981	-0.364602
C	1.921925	-0.805083	-0.064901
C	2.274769	0.505874	0.272561
C	3.611186	0.896245	0.301960
C	-1.595397	-0.845433	-0.989535
C	-1.423168	1.916176	-0.438785
H	5.652115	0.282867	0.051164
H	5.042250	-2.057670	-0.539606
H	2.663998	-2.733641	-0.630319
C	-2.807208	-0.007581	-1.198936
C	-2.575255	1.451427	-0.981403
H	-3.243297	-0.186834	-2.188566
H	-3.380604	2.130383	-1.236048
H	0.452467	-2.171048	-0.369406
H	-1.658842	-1.898876	-1.242469
H	-1.294641	2.978342	-0.258718
H	3.862398	1.920313	0.555284
O	-3.920283	-0.464640	-0.363302
O	-3.567936	-0.274528	1.004722
C	-3.466605	-1.559991	1.601935
H	-2.655099	-2.135780	1.152306
H	-3.248740	-1.363226	2.652679
H	-4.412810	-2.098153	1.514310

Solvent = Pentyl Ethanoate
E = -1105.757542
G = -1105.575620
ZPC = 0.225017
TCE = 0.239591
TCH = 0.240535
TCG = 0.181922
N_{imag} = 0

S	1.066763	1.683051	0.723415
N	0.610247	-1.210719	-0.052221
C	-0.451323	-0.358398	-0.386714
C	-0.327526	1.043280	-0.106678
C	4.644038	-0.048840	0.023648
C	4.292142	-1.359907	-0.287640
C	2.957159	-1.736260	-0.338372
C	1.946137	-0.812964	-0.054489

C	2.307266	0.500624	0.266470
C	3.646163	0.880519	0.291698
C	-1.590424	-0.844675	-0.943411
C	-1.395871	1.920384	-0.425933
H	5.684163	0.250913	0.053801
H	5.060008	-2.093715	-0.502532
H	2.681625	-2.754597	-0.592165
C	-2.795290	0.000446	-1.182454
C	-2.557280	1.457101	-0.947969
H	-3.177920	-0.158764	-2.199621
H	-3.368407	2.137052	-1.181543
H	0.463450	-2.176098	-0.318695
H	-1.663458	-1.900587	-1.185647
H	-1.265441	2.982159	-0.244061
H	3.902907	1.906854	0.530523
O	-3.944212	-0.458564	-0.421086
O	-3.673401	-0.258533	0.963008
C	-3.643942	-1.536331	1.568411
H	-2.818794	-2.139660	1.180857
H	-3.490786	-1.342630	2.631876
H	-4.593886	-2.057797	1.423349

PS-OOCH$_3^{\bullet}$ - Site 4
Solvent = Water
E = -1105.756236
G = -1105.573795
ZPC = 0.225326
TCE = 0.239852
TCH = 0.240796
TCG = 0.182442
N_{imag} = 0

S	-0.842483	-1.687687	0.254960
N	-0.874659	1.366538	0.192232
C	0.288773	0.791258	-0.266218
C	0.435937	-0.624469	-0.372076
C	-4.667260	-0.413349	0.084319
C	-4.537364	0.973090	0.142931
C	-3.278926	1.556226	0.170425
C	-2.130030	0.759644	0.160035
C	-2.261315	-0.633366	0.116584
C	-3.528367	-1.210406	0.064345
C	1.358858	1.622741	-0.698749
C	1.572576	-1.181579	-0.860637
H	-5.646986	-0.873513	0.052520
H	-5.417705	1.604283	0.156287
H	-3.166412	2.634935	0.201039
C	2.521531	1.103253	-1.161026
C	2.785574	-0.364502	-1.155669
H	3.313338	1.754056	-1.513092
H	3.265586	-0.689036	-2.085410
H	-0.889738	2.380416	0.181758
H	1.203814	2.697472	-0.680501
H	1.657488	-2.257793	-0.963802
H	-3.619212	-2.290121	0.019179
O	3.861105	-0.696028	-0.211767
O	3.428757	-0.363161	1.105808
C	4.290858	0.658608	1.586901
H	3.954435	0.851927	2.606551
H	5.326355	0.311837	1.598186
H	4.197420	1.563376	0.982975

Solvent = Pentyl Ethanoate
E = -1105.759756
G = -1105.577794
ZPC = 0.225162
TCE = 0.239729
TCH = 0.240674
TCG = 0.181962
N_{imag} = 0

S	-0.842575	-1.676607	0.239278
N	-0.917847	1.373449	0.231198
C	0.262578	0.825457	-0.220931
C	0.424088	-0.586130	-0.366779
C	-4.684440	-0.452928	0.061210
C	-4.575089	0.932797	0.151268
C	-3.325046	1.532244	0.198709
C	-2.164143	0.753272	0.179119
C	-2.275014	-0.640975	0.105499
C	-3.533773	-1.232294	0.031295
C	1.334154	1.677167	-0.603013
C	1.565952	-1.117204	-0.865763
H	-5.657312	-0.926249	0.012602
H	-5.464575	1.551137	0.172884
H	-3.231605	2.612230	0.252871
C	2.503886	1.183035	-1.076155
C	2.770843	-0.284185	-1.161932
H	3.296881	1.853380	-1.387685
H	3.192822	-0.547984	-2.140202
H	-0.941605	2.384455	0.275672
H	1.180898	2.750576	-0.535210
H	1.664636	-2.190650	-0.985203
H	-3.607991	-2.312045	-0.038687
O	3.886079	-0.672676	-0.311698
O	3.517338	-0.441195	1.045111
C	4.418977	0.520906	1.556001
H	4.144058	0.638511	2.606077
H	5.450300	0.164904	1.484761
H	4.314437	1.477298	1.036809

PS-OOCH$_3^{\bullet}$ - Site 5
Solvent = Water
E = -1105.752749
G = -1105.570306
ZPC = 0.225064
TCE = 0.239530
TCH = 0.240474
TCG = 0.182443
N_{imag} = 0

S	-0.369197	-1.486025	0.009557
N	-0.989074	1.325931	0.857904
C	0.227718	1.172554	0.163155
C	0.613300	-0.057798	-0.300584
C	-4.357162	-0.873600	-0.364246
C	-4.501884	0.431161	0.099233
C	-3.387995	1.169382	0.481996
C	-2.114954	0.599487	0.436332
C	-1.976007	-0.715913	-0.017141
C	-3.087874	-1.440761	-0.434012
C	1.058444	2.297545	-0.031286
C	1.910743	-0.280720	-1.012126
H	-5.222180	-1.447761	-0.672195
H	-5.484722	0.883978	0.154071
H	-3.492149	2.192244	0.828272

C	2.305586	2.158523	-0.690525
C	2.728619	0.960233	-1.165729
H	2.923244	3.039774	-0.820710
H	3.679424	0.855264	-1.675106
H	-1.203335	2.295357	1.064517
H	0.734736	3.262844	0.339454
H	1.728865	-0.743955	-1.992444
H	-2.959808	-2.454239	-0.797725
O	2.663283	-1.341022	-0.371111
O	2.971859	-0.928714	0.960126
C	4.388440	-0.867768	1.061875
H	4.580475	-0.594202	2.100190
H	4.829165	-1.843439	0.847554
H	4.793981	-0.107022	0.392105

Solvent = Pentyl Ethanoate
E = -1105.756477
G = -1105.574470
ZPC = 0.224756
TCE = 0.239292
TCH = 0.240236
TCG = 0.182007
N_{imag} = 0

S	-0.359752	-1.470170	-0.025574
N	-0.997940	1.341227	0.824322
C	0.228199	1.192154	0.150151
C	0.620623	-0.039633	-0.312749
C	-4.356429	-0.893808	-0.344965
C	-4.510323	0.405851	0.127044
C	-3.399354	1.156324	0.493597
C	-2.119517	0.603933	0.424513
C	-1.970735	-0.709142	-0.035110
C	-3.080968	-1.443693	-0.436777
C	1.061494	2.313843	-0.026343
C	1.929813	-0.260680	-1.007462
H	-5.218986	-1.478013	-0.641166
H	-5.498060	0.845439	0.201597
H	-3.513692	2.175958	0.846960
C	2.322344	2.176424	-0.662970
C	2.752563	0.981441	-1.135183
H	2.943364	3.058038	-0.776486
H	3.712339	0.879518	-1.628777
H	-1.209301	2.299429	1.073228
H	0.735339	3.279426	0.342370
H	1.749657	-0.699316	-2.000437
H	-2.945589	-2.454445	-0.805873
O	2.664090	-1.335165	-0.385416
O	2.945706	-0.953730	0.960259
C	4.354311	-0.932017	1.093758
H	4.533422	-0.690886	2.143202
H	4.781715	-1.911384	0.864032
H	4.800923	-0.164451	0.456516

PS-OOCH$_3^•$ - Site 5a
Solvent = Water
E = -1105.760801
G = -1105.578387
ZPC = 0.224695
TCE = 0.239233
TCH = 0.240178
TCG = 0.182414
N_{imag} = 0

S	-0.215401	-1.893483	0.197500
N	-0.238904	0.780540	-1.253296
C	0.995041	0.297071	-0.914537
C	1.083345	-0.586431	0.288740
C	-3.971928	-0.395986	0.233625
C	-3.829893	0.804152	-0.463627
C	-2.586340	1.198489	-0.930673
C	-1.462536	0.388773	-0.724418
C	-1.607016	-0.819361	-0.025535
C	-2.858665	-1.196014	0.458982
C	2.128434	0.656631	-1.631052
C	2.421735	-1.207340	0.473028
H	-4.940638	-0.703335	0.607553
H	-4.690477	1.438654	-0.638174
H	-2.463476	2.132065	-1.469837
C	3.370986	0.120527	-1.322345
C	3.490566	-0.837317	-0.274052
H	4.242829	0.406417	-1.895818
H	4.458283	-1.283445	-0.073898
H	-0.263792	1.530771	-1.935521
H	2.013099	1.347147	-2.460515
H	2.510077	-1.928047	1.278537
H	-2.955877	-2.124544	1.010293
O	0.673602	0.125664	1.492958
O	1.559385	1.234074	1.674946
C	0.758692	2.409802	1.648158
H	0.293234	2.544847	0.670028
H	0.000369	2.371516	2.432721
H	1.456704	3.224019	1.846711

Solvent = Pentyl Ethanoate
E = -1105.764991
G = -1105.582717
ZPC = 0.224593
TCE = 0.239150
TCH = 0.240094
TCG = 0.182274
N_{imag} = 0

S	-0.195449	-1.865430	0.310761
N	-0.237453	0.754331	-1.253122
C	0.996901	0.257577	-0.925013
C	1.094307	-0.539310	0.338181
C	-3.973133	-0.425680	0.226162
C	-3.841695	0.743677	-0.521824
C	-2.597201	1.137371	-0.986688
C	-1.463055	0.357311	-0.730739
C	-1.596239	-0.821118	0.019566
C	-2.849448	-1.194129	0.502443
C	2.112262	0.525316	-1.701818
C	2.435596	-1.145864	0.552929
H	-4.941700	-0.732788	0.601139
H	-4.710063	1.355092	-0.736724
H	-2.483430	2.050838	-1.561371
C	3.354329	-0.009159	-1.381302
C	3.488820	-0.865307	-0.251663
H	4.213292	0.205425	-2.003610
H	4.457079	-1.300826	-0.031099
H	-0.270219	1.433457	-2.003825
H	1.989795	1.139540	-2.588487
H	2.535916	-1.781940	1.425407
H	-2.938181	-2.097253	1.096035

O	0.675381	0.231524	1.486875
O	1.556649	1.352405	1.603318
C	0.740160	2.509295	1.561025
H	0.248298	2.613722	0.590680
H	-0.004294	2.487637	2.360794
H	1.427000	3.342342	1.721636

TS[PS-OOCH$_3^\bullet$] - Site 2a

Solvent = Water
E = -1105.743013
G = -1105.561788
ZPC = 0.223321
TCE = 0.237768
TCH = 0.238712
TCG = 0.181225
N_{imag} = 1, -480.92 cm^{-1}

S	-0.366582	1.675836	0.913792
N	-0.365499	-1.378930	0.784593
C	0.862652	-0.784707	0.515524
C	1.025045	0.630024	0.742470
C	-3.991540	0.433570	-0.316823
C	-3.876227	-0.954351	-0.287273
C	-2.671555	-1.550016	0.061025
C	-1.567559	-0.767389	0.402238
C	-1.691136	0.624496	0.384414
C	-2.894995	1.220211	0.014196
C	2.034085	-1.610742	0.671307
C	2.296371	1.179822	0.820982
H	-4.925633	0.903447	-0.598125
H	-4.723661	-1.577892	-0.545521
H	-2.568955	-2.629929	0.072398
C	3.272354	-1.047825	0.768876
C	3.417549	0.358171	0.817053
H	4.150167	-1.679759	0.826074
H	4.404344	0.798482	0.884136
H	-0.361625	-2.386792	0.665092
H	1.895019	-2.685896	0.642510
H	2.410888	2.254064	0.915627
H	-2.972116	2.301727	-0.006990
O	0.718506	-0.806323	-1.375183
O	1.835262	-0.287217	-2.023712
C	1.509381	0.993779	-2.547087
H	2.420560	1.359833	-3.021997
H	0.711307	0.906707	-3.288414
H	1.206814	1.673215	-1.747855

Solvent = Pentyl Ethanoate
E = -1105.743626
G = -1105.562501
ZPC = 0.223168
TCE = 0.237614
TCH = 0.238558
TCG = 0.181125
N_{imag} = 1, -617.20 cm^{-1}

S	-0.379725	1.745506	0.788271
N	-0.368184	-1.305410	0.878808
C	0.865587	-0.731102	0.555476
C	1.021612	0.696664	0.683071
C	-4.000442	0.404617	-0.356895
C	-3.881538	-0.975508	-0.221641
C	-2.674037	-1.539296	0.168579
C	-1.570930	-0.732051	0.450001
C	-1.699704	0.654750	0.327963
C	-2.904141	1.215683	-0.087194
C	2.037984	-1.534419	0.799932
C	2.289199	1.254827	0.713715
H	-4.935738	0.849555	-0.673214
H	-4.727318	-1.619578	-0.431426
H	-2.571541	-2.615873	0.258697
C	3.275330	-0.962322	0.846706
C	3.416176	0.440365	0.772009
H	4.154841	-1.585362	0.956617
H	4.401375	0.889180	0.797571
H	-0.360691	-2.317502	0.836585
H	1.905014	-2.609197	0.859998
H	2.398221	2.334089	0.724133
H	-2.982802	2.292496	-0.190813
O	0.732550	-0.922602	-1.281389
O	1.852293	-0.476638	-1.954524
C	1.536375	0.745702	-2.601275
H	2.458308	1.071887	-3.085948
H	0.756896	0.589286	-3.351978
H	1.208757	1.497255	-1.879578

TS[PS-OOCH$_3^\bullet$] - Site 2

Solvent = Water
E = -1105.742164
G = -1105.561571
ZPC = 0.223610
TCE = 0.238209
TCH = 0.239153
TCG = 0.180593
N_{imag} = 1, -529.69 cm^{-1}

S	0.702849	1.682138	0.709156
N	0.388287	-1.167437	-0.309697
C	-0.666531	-0.336479	-0.583723
C	-0.628497	1.034633	-0.276358
C	4.367200	0.113215	0.035308
C	4.076979	-1.194129	-0.344275
C	2.757189	-1.610238	-0.465989
C	1.713470	-0.728849	-0.183755
C	2.005539	0.580210	0.212301
C	3.328803	1.000550	0.302000
C	-1.863765	-0.898835	-1.134837
C	-1.667113	1.860137	-0.661571
H	5.394086	0.445621	0.122987
H	4.877603	-1.891975	-0.557268
H	2.518418	-2.621552	-0.777593
C	-2.859180	-0.002157	-1.632469
C	-2.780161	1.335491	-1.362312
H	-3.702256	-0.416832	-2.170932
H	-3.558021	2.012251	-1.694351
H	0.279619	-2.127693	-0.618144
H	-1.775119	-1.882100	-1.586089
H	-1.622581	2.919376	-0.435887
H	3.544232	2.021590	0.596279
O	-2.682884	-1.684607	0.364873
O	-3.156787	-0.697813	1.218457
C	-2.279602	-0.577264	2.332339
H	-2.718440	0.194390	2.965964
H	-2.228040	-1.522988	2.876286
H	-1.281707	-0.270586	2.013545

Solvent = Pentyl Ethanoate
E = -1105.744915
G = -1105.564217
ZPC = 0.223584
TCE = 0.238141
TCH = 0.239085
TCG = 0.180698
N_{imag} = 1, -630.73 cm^{-1}

S	0.710546	1.633447	0.840526
N	0.357792	-1.140099	-0.298676
C	-0.681020	-0.274266	-0.572437
C	-0.627068	1.069865	-0.189748
C	4.355597	0.102217	-0.024819
C	4.048677	-1.186812	-0.448528
C	2.723998	-1.593985	-0.549784
C	1.691473	-0.724064	-0.198437
C	2.002361	0.565539	0.247013
C	3.328456	0.979430	0.310632
C	-1.874472	-0.790284	-1.175549
C	-1.649278	1.937953	-0.540217
H	5.386209	0.427823	0.044525
H	4.840137	-1.877373	-0.714302
H	2.477047	-2.591164	-0.899164
C	-2.852682	0.152205	-1.625862
C	-2.760673	1.471148	-1.275177
H	-3.698211	-0.218090	-2.192294
H	-3.530712	2.173655	-1.570503
H	0.226915	-2.090594	-0.622378
H	-1.779090	-1.728161	-1.715519
H	-1.586469	2.981573	-0.254454
H	3.554797	1.987254	0.640501
O	-2.709091	-1.686091	0.200683
O	-3.122025	-0.795140	1.167614
C	-2.220538	-0.847018	2.263854
H	-2.608038	-0.135329	2.994685
H	-2.203789	-1.851292	2.695521
H	-1.212159	-0.554466	1.963668

TS[PS-OOCH$_3^•$] - Site 3
Solvent = Water
E = -1105.739851
G = -1105.558615
ZPC = 0.223755
TCE = 0.238142
TCH = 0.239086
TCG = 0.181235
N_{imag} = 1, -583.35 cm^{-1}

S	0.843603	1.629126	0.780989
N	0.535636	-1.218799	-0.198967
C	-0.522665	-0.372272	-0.542020
C	-0.485077	0.996296	-0.170565
C	4.512877	0.104758	0.062625
C	4.227676	-1.196817	-0.343174
C	2.911761	-1.627038	-0.453220
C	1.858103	-0.767802	-0.133895
C	2.150612	0.534305	0.282447
C	3.469213	0.972331	0.365462
C	-1.624595	-0.857813	-1.208952
C	-1.540881	1.857516	-0.522251
H	5.537470	0.446270	0.140034
H	5.032559	-1.880366	-0.585574
H	2.682541	-2.635821	-0.780156
C	-2.783884	-0.046273	-1.374077
C	-2.656918	1.364582	-1.146138
H	-3.536087	-0.382195	-2.078519
H	-3.476076	2.021222	-1.410497
H	0.439592	-2.158582	-0.567024
H	-1.648730	-1.897974	-1.514770
H	-1.458097	2.914144	-0.293789
H	3.675644	1.990182	0.677189
O	-3.902014	-0.505767	0.055821
O	-3.197619	-0.316720	1.231945
C	-2.791073	-1.581399	1.742824
H	-2.187001	-2.117347	1.008263
H	-2.196544	-1.360480	2.630149
H	-3.666199	-2.175815	2.015140

Solvent = Pentyl Ethanoate
E = -1105.742354
G = -1105.561804
ZPC = 0.223530
TCE = 0.238000
TCH = 0.238945
TCG = 0.180550
N_{imag} = 1, -652.35 cm^{-1}

S	0.825755	1.626047	0.769939
N	0.541538	-1.228229	-0.168562
C	-0.523273	-0.395258	-0.534532
C	-0.494356	0.977037	-0.191290
C	4.509431	0.128821	0.046287
C	4.234787	-1.183502	-0.327389
C	2.921864	-1.626899	-0.421828
C	1.861765	-0.770825	-0.115838
C	2.143755	0.543326	0.272030
C	3.458757	0.993202	0.334828
C	-1.620602	-0.902747	-1.193674
C	-1.548015	1.825307	-0.575003
H	5.531391	0.481396	0.110184
H	5.044734	-1.865438	-0.558013
H	2.703821	-2.645405	-0.726249
C	-2.790347	-0.103955	-1.367500
C	-2.661251	1.313832	-1.188273
H	-3.536009	-0.459256	-2.070137
H	-3.483224	1.960819	-1.467607
H	0.447924	-2.181100	-0.497712
H	-1.640875	-1.949285	-1.479114
H	-1.468218	2.887698	-0.372902
H	3.657136	2.019945	0.622109
O	-3.878778	-0.541020	0.048620
O	-3.207573	-0.256078	1.215436
C	-2.764373	-1.467042	1.807516
H	-2.104154	-2.015240	1.131245
H	-2.214992	-1.172100	2.703122
H	-3.618427	-2.092188	2.081540

TS[PS-OOCH$_3^•$] - Site 4
Solvent = Water
E = -1105.743331
G = -1105.561765
ZPC = 0.224119
TCE = 0.238519
TCH = 0.239463
TCG = 0.181565

N_{imag} = 1, -476.26 cm^{-1}

S	-0.795358	-1.697415	0.322615
N	-0.749252	1.339660	0.023659
C	0.372927	0.700569	-0.437463
C	0.472845	-0.716117	-0.432975
C	-4.590924	-0.334850	0.168375
C	-4.423037	1.046479	0.109855
C	-3.148583	1.593726	0.054175
C	-2.024622	0.765876	0.078547
C	-2.193618	-0.620253	0.151207
C	-3.474900	-1.164494	0.180405
C	1.443638	1.459461	-0.958201
C	1.594978	-1.334044	-0.919686
H	-5.583083	-0.767392	0.202147
H	-5.285414	1.701877	0.097473
H	-3.006864	2.667578	-0.006962
C	2.580496	0.845957	-1.411852
C	2.743240	-0.567558	-1.285237
H	3.389341	1.437031	-1.823725
H	3.514558	-1.054078	-1.870110
H	-0.738441	2.350608	-0.061302
H	1.331395	2.537312	-1.012458
H	1.653369	-2.416322	-0.939476
H	-3.595087	-2.241300	0.224211
O	3.837305	-0.727510	0.263448
O	3.145492	-0.157313	1.327786
C	3.765454	1.074773	1.672052
H	3.159603	1.495658	2.475759
H	4.784452	0.899153	2.025667
H	3.776926	1.752330	0.816246

Solvent = Pentyl Ethanoate
E = -1105.744343
G = -1105.563131
ZPC = 0.223888
TCE = 0.238299
TCH = 0.239243
TCG = 0.181212
N_{imag} = 1, -601.92 cm^{-1}

S	-0.783789	-1.689157	0.341935
N	-0.751617	1.338915	0.095277
C	0.374758	0.714287	-0.398630
C	0.477902	-0.699055	-0.417729
C	-4.589602	-0.351043	0.116960
C	-4.428195	1.031142	0.092959
C	-3.155275	1.585430	0.074886
C	-2.026647	0.764432	0.107162
C	-2.189835	-0.624649	0.148230
C	-3.468497	-1.174188	0.134915
C	1.429230	1.482537	-0.928934
C	1.592973	-1.307564	-0.930494
H	-5.579997	-0.789293	0.120086
H	-5.293878	1.682554	0.076992
H	-3.021133	2.661671	0.039328
C	2.564545	0.879260	-1.408159
C	2.739853	-0.533534	-1.291694
H	3.363085	1.478449	-1.828957
H	3.497637	-1.008257	-1.904157
H	-0.741764	2.349976	0.041411
H	1.313025	2.560941	-0.973790
H	1.653776	-2.389284	-0.962915
H	-3.582594	-2.252494	0.152326
O	3.831941	-0.719965	0.205596
O	3.156826	-0.200266	1.291973
C	3.769916	1.021560	1.666260
H	3.175545	1.410832	2.494995
H	4.798062	0.848234	1.996410
H	3.763286	1.730937	0.835428

TS[PS-OOCH$_3^\bullet$] - Site 5
Solvent = Water
E = -1105.737853
G = -1105.556533
ZPC = 0.223797
TCE = 0.238180
TCH = 0.239124
TCG = 0.181319
N_{imag} = 1, -611.67 cm^{-1}

S	0.519428	-1.590974	0.063447
N	0.813636	1.292813	-0.729869
C	-0.337622	1.005498	0.021916
C	-0.574490	-0.275131	0.487402
C	4.446625	-0.610464	0.191899
C	4.436938	0.714295	-0.235187
C	3.235299	1.353849	-0.517762
C	2.027620	0.663973	-0.407194
C	2.042822	-0.670229	0.011358
C	3.243490	-1.297359	0.327494
C	-1.270487	2.019547	0.293549
C	-1.804818	-0.598135	1.144346
H	5.380313	-1.108357	0.422237
H	5.367183	1.259854	-0.339905
H	3.220109	2.390846	-0.836067
C	-2.421873	1.749964	1.046413
C	-2.669822	0.482858	1.516766
H	-3.103938	2.560700	1.272748
H	-3.547046	0.264316	2.112560
H	0.921792	2.281774	-0.926780
H	-1.076856	3.021231	-0.074697
H	-1.828668	-1.501018	1.745918
H	3.235744	-2.327684	0.665176
O	-2.740989	-1.456933	-0.185071
O	-2.913084	-0.593884	-1.249985
C	-4.275161	-0.177520	-1.296270
H	-4.549548	0.334458	-0.372415
H	-4.339107	0.508722	-2.141178
H	-4.924821	-1.039297	-1.461595

Solvent = Pentyl Ethanoate
E = -1105.740535
G = -1105.559672
ZPC = 0.223528
TCE = 0.237982
TCH = 0.238926
TCG = 0.180863
N_{imag} = 1, -663.44 cm^{-1}

S	0.506887	-1.577875	0.074049
N	0.830399	1.299663	-0.724598
C	-0.334661	1.024246	0.007785
C	-0.580454	-0.252173	0.482545
C	4.443662	-0.634325	0.198690
C	4.448595	0.685246	-0.241465

C	3.253456	1.335617	-0.526375
C	2.037212	0.661737	-0.406867
C	2.037539	-0.670095	0.022991
C	3.232485	-1.305092	0.342536
C	-1.264494	2.043783	0.260894
C	-1.815609	-0.565094	1.137379
H	5.372105	-1.140872	0.432045
H	5.384984	1.218998	-0.354011
H	3.253415	2.370321	-0.853586
C	-2.420502	1.786018	1.010923
C	-2.673774	0.526608	1.497580
H	-3.100044	2.602253	1.227308
H	-3.551179	0.320587	2.098055
H	0.937843	2.277360	-0.964297
H	-1.066311	3.041647	-0.115347
H	-1.830375	-1.450062	1.765998
H	3.212987	-2.332264	0.689685
O	-2.738371	-1.457854	-0.144435
O	-2.915869	-0.641443	-1.236423
C	-4.275490	-0.235062	-1.290706
H	-4.549747	0.320635	-0.391438
H	-4.352947	0.409793	-2.167387
H	-4.927586	-1.104133	-1.408223

TS[PS-OOCH$_3$•] - Site 5a
Solvent = Water
E = -1105.745348
G = -1105.564082
ZPC = 0.223432
TCE = 0.237956
TCH = 0.238900
TCG = 0.181266
N_{imag} = 1, -431.63 cm^{-1}

S	0.364314	-1.946319	-0.092393
N	0.289749	0.747651	1.332996
C	-0.930827	0.248059	0.979123
C	-1.026103	-0.846902	0.053454
C	3.990364	-0.196028	-0.387163
C	3.820007	0.991396	0.321831
C	2.586902	1.304043	0.874897
C	1.509125	0.424970	0.738572
C	1.683064	-0.769003	0.029456
C	2.918819	-1.069014	-0.537172
C	-2.103259	0.843580	1.465989
C	-2.310075	-1.445745	-0.145910
H	4.948987	-0.440812	-0.827338
H	4.647157	1.680857	0.439470
H	2.440098	2.228422	1.423739
C	-3.335987	0.315363	1.146705
C	-3.436898	-0.848067	0.345405
H	-4.234188	0.782088	1.531788
H	-4.410089	-1.277954	0.142451
H	0.278367	1.603575	1.877960
H	-2.016225	1.711295	2.110895
H	-2.368728	-2.346488	-0.746236
H	3.039412	-1.991458	-1.094076
O	-0.744727	0.056676	-1.621051
O	-1.741567	1.007089	-1.835543
C	-1.203460	2.303833	-1.612777
H	-0.861293	2.414432	-0.581544
H	-0.379704	2.494864	-2.304627
H	-2.020737	2.999703	-1.806886

Solvent = Pentyl Ethanoate
E = -1105.745563
G = -1105.564853
ZPC = 0.223021
TCE = 0.237630
TCH = 0.238574
TCG = 0.180710
N_{imag} = 1, -587.00 cm^{-1}

S	0.351264	-1.945319	-0.049666
N	0.288994	0.763224	1.333009
C	-0.941105	0.266525	0.980156
C	-1.037271	-0.827775	0.055498
C	3.983067	-0.208571	-0.390743
C	3.822331	0.981921	0.312161
C	2.591275	1.304674	0.866624
C	1.508160	0.431100	0.743740
C	1.674383	-0.769942	0.043342
C	2.904206	-1.075428	-0.530006
C	-2.108695	0.868023	1.456989
C	-2.323041	-1.418771	-0.146492
H	4.937922	-0.460685	-0.835372
H	4.653157	1.668653	0.422139
H	2.456279	2.236882	1.405472
C	-3.348212	0.346244	1.132939
C	-3.451868	-0.813797	0.336049
H	-4.244073	0.821617	1.513714
H	-4.426122	-1.236948	0.123408
H	0.284151	1.606056	1.893782
H	-2.025031	1.736138	2.102533
H	-2.382327	-2.316678	-0.750833
H	3.014449	-2.000336	-1.085181
O	-0.730617	0.021791	-1.606422
O	-1.701861	0.965487	-1.867255
C	-1.160861	2.257764	-1.648899
H	-0.867005	2.391986	-0.604882
H	-0.300192	2.426505	-2.301437
H	-1.958773	2.959306	-1.898485

PSE-OH• - Site 2a
Solvent = Water
E = -2994.773044
G = -2994.620581
ZPC = 0.191959
TCE = 0.204156
TCH = 0.205100
TCG = 0.152464
N_{imag} = 0

Se	-0.101661	-1.688129	-0.002454
N	0.032022	1.453522	-0.540032
C	1.130402	0.932017	0.246593
C	1.375061	-0.524232	-0.067928
C	-3.882676	0.015310	0.056832
C	-3.651624	1.361523	-0.224899
C	-2.356532	1.825354	-0.399135
C	-1.260498	0.957442	-0.313126
C	-1.501501	-0.395923	-0.045502
C	-2.805465	-0.855063	0.148309
C	2.352526	1.771856	-0.001128
C	2.633698	-1.020198	-0.312125
H	-4.890300	-0.352916	0.205125

```
H    -4.480956   2.055174  -0.297420
H    -2.171068   2.875065  -0.604105
C     3.570567   1.235544  -0.244441
C     3.744704  -0.171605  -0.380286
H     4.430423   1.886949  -0.352331
H     4.727370  -0.581708  -0.572601
H     0.046910   2.468821  -0.557480
H     2.207304   2.843611   0.095270
H     2.762570  -2.084873  -0.477080
H    -2.969938  -1.905395   0.364337
O     0.750681   1.063698   1.637813
H     1.472588   0.715270   2.178456
```

Solvent = Pentyl Ethanoate
E = -2994.770788
G = -2994.619175
ZPC = 0.191484
TCE = 0.203913
TCH = 0.204857
TCG = 0.151613
N_{imag} = 0

```
Se   -0.107193  -1.680048   0.028417
N     0.034373   1.471254  -0.462302
C     1.142395   0.924118   0.285552
C     1.370185  -0.525031  -0.080108
C    -3.888375   0.016607  -0.003473
C    -3.652010   1.364452  -0.270995
C    -2.354427   1.832206  -0.403002
C    -1.257027   0.968350  -0.286140
C    -1.504268  -0.387994  -0.038082
C    -2.811917  -0.850771   0.112861
C     2.366335   1.761497   0.029593
C     2.615125  -1.018210  -0.392385
H    -4.899094  -0.354826   0.112895
H    -4.480600   2.056700  -0.364949
H    -2.167337   2.884086  -0.595526
C     3.570257   1.226574  -0.278288
C     3.728253  -0.174747  -0.476185
H     4.431498   1.876025  -0.389195
H     4.698992  -0.582828  -0.725721
H     0.060214   2.483807  -0.500345
H     2.238862   2.829464   0.179422
H     2.730143  -2.077115  -0.600396
H    -2.981661  -1.903220   0.315219
O     0.790580   1.021465   1.680994
H     1.501116   0.616732   2.194460
```

PSE-OH• - Site 2
Solvent = Water
E = -2994.774287
G = -2994.622047
ZPC = 0.192344
TCE = 0.204776
TCH = 0.205721
TCG = 0.152240
N_{imag} = 0

```
Se   -0.420919  -1.716043   0.395675
N     0.044911   1.381083   0.347380
C     1.152810   0.640207   0.004442
C     1.146325  -0.735887  -0.117012
C    -3.942784   0.353325  -0.474619
C    -3.560033   1.688247  -0.368653
C    -2.236936   2.017576  -0.105742
C    -1.281581   1.014285   0.080329
C    -1.671564  -0.326003  -0.005714
C    -2.992756  -0.648821  -0.302232
C     2.407725   1.451876  -0.212950
C     2.284820  -1.435881  -0.521747
H    -4.971511   0.091120  -0.689053
H    -4.289369   2.478395  -0.501176
H    -1.926978   3.055379  -0.039344
C     3.572081   0.618469  -0.640379
C     3.495836  -0.727975  -0.772095
H     4.496994   1.151678  -0.829581
H     4.371741  -1.291083  -1.074958
H     0.202740   2.381719   0.395377
H     2.195287   2.227050  -0.959832
H     2.251560  -2.513158  -0.623257
H    -3.279203  -1.691746  -0.379571
O     2.716379   2.217640   0.971622
H     2.970923   1.590898   1.662049
```

Solvent = Pentyl Ethanoate
E = -2994.774967
G = -2994.623057
ZPC = 0.192236
TCE = 0.204695
TCH = 0.205639
TCG = 0.151910
N_{imag} = 0

```
Se   -0.422706  -1.727850   0.372508
N     0.052608   1.365537   0.339191
C     1.158692   0.621076   0.004003
C     1.153501  -0.753415  -0.116453
C    -3.942464   0.357299  -0.458969
C    -3.558926   1.689418  -0.333487
C    -2.233616   2.014158  -0.076439
C    -1.276731   1.008516   0.086565
C    -1.668909  -0.330791  -0.015504
C    -2.991468  -0.647490  -0.309019
C     2.402414   1.441606  -0.255866
C     2.305924  -1.454996  -0.479474
H    -4.972759   0.098938  -0.671147
H    -4.289254   2.481763  -0.447503
H    -1.925367   3.051842   0.001331
C     3.602550   0.597393  -0.538817
C     3.531193  -0.750788  -0.657115
H     4.539018   1.127681  -0.671831
H     4.426302  -1.318849  -0.886272
H     0.226020   2.356887   0.455384
H     2.199046   2.107222  -1.107506
H     2.275452  -2.532338  -0.582303
H    -3.279471  -1.688936  -0.402123
O     2.625594   2.363111   0.823264
H     2.907052   1.847136   1.589680
```

PSE-OH• - Site 3
Solvent = Water
E = -2994.770780
G = -2994.618667
ZPC = 0.192292
TCE = 0.204724
TCH = 0.205669

TCG = 0.152113
N_{imag} = 0

Se	0.501405	-1.664329	-0.306141
N	0.132497	1.417640	-0.608971
C	-1.023705	0.782457	-0.112434
C	-1.021814	-0.634589	0.089039
C	4.057966	0.366739	0.476876
C	3.707981	1.700837	0.277949
C	2.403552	2.043459	-0.051149
C	1.425553	1.055178	-0.210783
C	1.786589	-0.282443	-0.019526
C	3.089695	-0.621201	0.335789
C	-2.152277	1.503642	0.123990
C	-2.211695	-1.284499	0.511546
H	5.072239	0.096474	0.743439
H	4.450963	2.481811	0.389032
H	2.125293	3.082440	-0.196046
C	-3.456343	0.883138	0.507650
C	-3.359520	-0.594151	0.724533
H	-3.842703	1.362398	1.414469
H	-4.259790	-1.106189	1.045310
H	0.015521	2.421838	-0.684507
H	-2.137585	2.580669	-0.012766
H	-2.186336	-2.358323	0.664444
H	3.344273	-1.663741	0.491257
O	-4.480342	1.181798	-0.473897
H	-4.190304	0.800554	-1.312742

Solvent = Pentyl Ethanoate
E = -2994.770321
G = -2994.618776
ZPC = 0.192039
TCE = 0.204586
TCH = 0.205530
TCG = 0.151544
N_{imag} = 0

Se	0.496332	-1.655248	-0.296634
N	0.135153	1.434680	-0.538400
C	-1.028050	0.791002	-0.080422
C	-1.026407	-0.629809	0.103492
C	4.077634	0.345756	0.433782
C	3.733649	1.682733	0.249147
C	2.424602	2.037357	-0.044728
C	1.433104	1.058952	-0.184808
C	1.790242	-0.282922	-0.013418
C	3.098200	-0.632790	0.309494
C	-2.164580	1.504553	0.141274
C	-2.221816	-1.286842	0.497008
H	5.095730	0.066184	0.675225
H	4.485518	2.457373	0.345171
H	2.155138	3.080546	-0.176580
C	-3.477260	0.876900	0.486689
C	-3.375886	-0.602429	0.695022
H	-3.883731	1.345357	1.391018
H	-4.281354	-1.120833	0.990229
H	0.018947	2.435298	-0.638176
H	-2.154220	2.583517	0.018502
H	-2.196519	-2.362466	0.639157
H	3.348960	-1.678187	0.453204
O	-4.478023	1.180460	-0.507098
H	-4.166251	0.810446	-1.341814

PSE-OH• - Site 4
Solvent = Water
E = -2994.773995
G = -2994.621952
ZPC = 0.192412
TCE = 0.204929
TCH = 0.205873
TCG = 0.152043
N_{imag} = 0

Se	0.283359	-1.587302	-0.202688
N	0.359192	1.562244	-0.465430
C	-0.878545	1.061377	-0.120903
C	-1.116779	-0.322843	0.122831
C	4.127758	-0.087250	0.366037
C	3.979335	1.280643	0.142197
C	2.726315	1.812906	-0.121230
C	1.598536	0.984636	-0.185036
C	1.752714	-0.390831	0.023037
C	3.011584	-0.914054	0.311834
C	-1.973644	1.965970	-0.002715
C	-2.340203	-0.776765	0.495539
H	5.102038	-0.508038	0.581957
H	4.839508	1.938161	0.182355
H	2.602226	2.878823	-0.282282
C	-3.215135	1.548298	0.341139
C	-3.547837	0.107326	0.567269
H	-4.024600	2.262941	0.438709
H	-4.056003	-0.021945	1.528505
H	0.386944	2.567878	-0.592155
H	-1.780411	3.020399	-0.178904
H	-2.493670	-1.832174	0.694760
H	3.114598	-1.980066	0.482584
O	-4.551640	-0.334518	-0.384894
H	-4.170660	-0.233749	-1.266973

Solvent = Pentyl Ethanoate
E = -2994.773456
G = -2994.621578
ZPC = 0.192366
TCE = 0.204929
TCH = 0.205873
TCG = 0.151878
N_{imag} = 0

Se	0.282646	-1.582405	-0.200264
N	0.363870	1.567646	-0.436444
C	-0.878809	1.065367	-0.113461
C	-1.117909	-0.320131	0.127566
C	4.133748	-0.095610	0.350250
C	3.987864	1.273317	0.135726
C	2.734119	1.810181	-0.113061
C	1.603284	0.986107	-0.173171
C	1.755041	-0.391366	0.025069
C	3.014688	-0.918352	0.300670
C	-1.978184	1.966600	-0.014063
C	-2.343170	-0.774251	0.491197
H	5.108589	-0.520531	0.555789
H	4.850433	1.928132	0.172621
H	2.614117	2.878158	-0.265172
C	-3.222542	1.547887	0.318125
C	-3.554898	0.107557	0.558092

H	-4.037131	2.259372	0.394642
H	-4.053709	-0.010266	1.526659
H	0.392115	2.569444	-0.581398
H	-1.788839	3.020708	-0.198641
H	-2.499355	-1.830371	0.685268
H	3.116443	-1.985473	0.466552
O	-4.560012	-0.344546	-0.374076
H	-4.179020	-0.268418	-1.257265

PSE-OH• - Site 5
Solvent = Water
E = -2994.771167
G = -2994.618729
ZPC = 0.192270
TCE = 0.204601
TCH = 0.205545
TCG = 0.152438
N_{imag} = 0

Se	0.033874	-1.503594	-0.293130
N	0.399691	1.521082	-0.820931
C	-0.863089	1.196835	-0.277776
C	-1.196802	-0.094446	0.027419
C	3.914956	-0.261256	0.654947
C	3.896228	1.089763	0.317105
C	2.727793	1.681943	-0.146234
C	1.565397	0.923053	-0.308818
C	1.594319	-0.436276	0.015892
C	2.755720	-1.018506	0.514825
C	-1.806659	2.236317	-0.091360
C	-2.527996	-0.482007	0.601719
H	4.821358	-0.723648	1.025990
H	4.791223	1.691132	0.424446
H	2.703974	2.738318	-0.393203
C	-3.113290	1.937229	0.366195
C	-3.479668	0.670420	0.689576
H	-3.829164	2.746439	0.455591
H	-4.482039	0.442300	1.034120
H	0.511868	2.519159	-0.959505
H	-1.527122	3.254805	-0.334626
H	-2.375529	-0.905283	1.603929
H	2.754151	-2.070778	0.776198
O	-3.103486	-1.577124	-0.134205
H	-3.291847	-1.259251	-1.027305

Solvent = Pentyl Ethanoate
E = -2994.770917
G = -2994.618774
ZPC = 0.192053
TCE = 0.204435
TCH = 0.205380
TCG = 0.152142
N_{imag} = 0

Se	0.031518	-1.494052	-0.278398
N	0.399737	1.542105	-0.767808
C	-0.867224	1.208874	-0.250372
C	-1.198619	-0.088631	0.040085
C	3.930677	-0.275147	0.616441
C	3.914367	1.078338	0.292759
C	2.740893	1.682385	-0.141166
C	1.570459	0.933019	-0.290774
C	1.597332	-0.430830	0.017607
C	2.763959	-1.023082	0.489772
C	-1.822331	2.236965	-0.074813
C	-2.538255	-0.491094	0.585524
H	4.840749	-0.747137	0.966102
H	4.815166	1.673136	0.389287
H	2.722291	2.742470	-0.373885
C	-3.139514	1.922673	0.344781
C	-3.504150	0.651632	0.649683
H	-3.865099	2.725135	0.418441
H	-4.514184	0.411781	0.962936
H	0.508872	2.533664	-0.939020
H	-1.547799	3.261254	-0.299789
H	-2.401134	-0.909142	1.592809
H	2.761178	-2.077767	0.742309
O	-3.076246	-1.595463	-0.156041
H	-3.265410	-1.277271	-1.047830

PSE-OH• - Site 5a
Solvent = Water
E = -2994.778815
G = -2994.626714
ZPC = 0.191580
TCE = 0.203995
TCH = 0.204939
TCG = 0.152101
N_{imag} = 0

Se	0.063679	-1.483561	-0.446655
N	-0.123142	1.656569	-0.182882
C	1.130984	1.109053	-0.098863
C	1.264107	-0.222626	0.563866
C	-3.875952	-0.197369	0.069900
C	-3.752702	1.190524	0.148275
C	-2.502786	1.783880	0.087621
C	-1.347982	1.004167	-0.071904
C	-1.473359	-0.391083	-0.160628
C	-2.736294	-0.978210	-0.074398
C	2.237525	1.794924	-0.581538
C	2.651471	-0.749954	0.563199
H	-4.849773	-0.667863	0.127372
H	-4.632857	1.811542	0.264830
H	-2.396956	2.861609	0.155176
C	3.510799	1.241425	-0.520476
C	3.695409	-0.048352	0.052468
H	4.358993	1.787976	-0.911394
H	4.692654	-0.471874	0.093470
H	-0.168839	2.637206	-0.435711
H	2.080244	2.772202	-1.026811
H	2.788381	-1.724450	1.018788
H	-2.819852	-2.057867	-0.128976
O	0.698382	-0.212037	1.878572
H	1.250552	0.374648	2.415982

Solvent = Pentyl Ethanoate
E = -2994.778296
G = -2994.626164
ZPC = 0.191628
TCE = 0.204067
TCH = 0.205011
TCG = 0.152131
N_{imag} = 0

Se	0.065786	-1.478889	-0.448451

N	-0.123751	1.655109	-0.182232
C	1.131050	1.109384	-0.099105
C	1.262079	-0.222581	0.567496
C	-3.875136	-0.199922	0.067586
C	-3.754075	1.186901	0.150599
C	-2.504300	1.781028	0.092342
C	-1.349800	1.003327	-0.071155
C	-1.472619	-0.391464	-0.162351
C	-2.734533	-0.978918	-0.077740
C	2.237987	1.792013	-0.584316
C	2.650816	-0.748347	0.569018
H	-4.848445	-0.671847	0.124733
H	-4.634979	1.806340	0.271262
H	-2.401927	2.858795	0.169552
C	3.511626	1.238409	-0.520439
C	3.695548	-0.048184	0.057621
H	4.359735	1.782801	-0.914803
H	4.692925	-0.471500	0.101418
H	-0.169138	2.631273	-0.446793
H	2.085604	2.766674	-1.037533
H	2.787238	-1.721306	1.028371
H	-2.816645	-2.058924	-0.130583
O	0.690665	-0.213002	1.874848
H	1.257971	0.348285	2.421232

TS[PSE-OH•] - Site 2a

Solvent = Water
E = -2994.747617
G = -2994.598416
ZPC = 0.189387
TCE = 0.202078
TCH = 0.203022
TCG = 0.149201
N_{imag} = 1, -361.18 cm^{-1}

Se	-0.105757	-1.677467	-0.107386
N	-0.034874	1.474211	-0.571506
C	1.154858	0.924442	-0.174786
C	1.344945	-0.500014	-0.121672
C	-3.882935	-0.006135	0.190228
C	-3.673861	1.347874	-0.078536
C	-2.396288	1.822103	-0.320733
C	-1.298373	0.951860	-0.304991
C	-1.512777	-0.406150	-0.048520
C	-2.803364	-0.875617	0.204546
C	2.323520	1.746074	-0.335644
C	2.635289	-1.024753	-0.039287
H	-4.880067	-0.380043	0.386415
H	-4.509853	2.036683	-0.092124
H	-2.225193	2.874654	-0.521779
C	3.572157	1.202174	-0.267520
C	3.737910	-0.192816	-0.098768
H	4.443426	1.839564	-0.354632
H	4.733403	-0.614690	-0.039436
H	-0.014202	2.481267	-0.691949
H	2.175888	2.811443	-0.474807
H	2.764356	-2.097155	0.052866
H	-2.952317	-1.930382	0.407688
O	0.916053	1.119852	1.892997
H	1.769493	0.800013	2.209547

Solvent = Pentyl Ethanoate
E = -2994.739416
G = -2994.590769
ZPC = 0.189027
TCE = 0.201818
TCH = 0.202762
TCG = 0.148647
N_{imag} = 1, -354.34 cm^{-1}

Se	-0.100554	-1.700013	-0.181020
N	-0.036905	1.393632	-0.778876
C	1.158868	0.893200	-0.282508
C	1.356463	-0.503205	-0.085323
C	-3.845305	0.025766	0.356721
C	-3.640561	1.359492	0.014868
C	-2.374278	1.804150	-0.339242
C	-1.292881	0.920400	-0.378730
C	-1.506967	-0.419812	-0.047798
C	-2.772542	-0.858081	0.330574
C	2.315129	1.722725	-0.367571
C	2.632874	-1.001169	0.153086
H	-4.828392	-0.325078	0.645785
H	-4.465725	2.061649	0.034957
H	-2.207715	2.847218	-0.589091
C	3.571445	1.208491	-0.154634
C	3.736756	-0.158283	0.117530
H	4.434128	1.862114	-0.197394
H	4.726606	-0.562841	0.290855
H	-0.017673	2.396770	-0.916401
H	2.170710	2.778100	-0.572860
H	2.762633	-2.060644	0.344129
H	-2.916311	-1.898811	0.600175
O	0.796089	1.253985	1.738612
H	1.616182	0.952622	2.155936

TS[PSE-OH•] - Site 2

Solvent = Water
E = -2994.744099
G = -2994.596784
ZPC = 0.188588
TCE = 0.201605
TCH = 0.202550
TCG = 0.147315
N_{imag} = 1, -421.78 cm^{-1}

Se	-0.336815	-1.657808	0.412884
N	-0.010056	1.448105	0.294286
C	1.115940	0.765859	-0.127777
C	1.146491	-0.635348	-0.224816
C	-3.969361	0.230092	-0.416814
C	-3.638746	1.581821	-0.364334
C	-2.324484	1.972939	-0.144832
C	-1.324867	1.016654	0.052254
C	-1.662549	-0.339050	0.017143
C	-2.975346	-0.726488	-0.236532
C	2.286643	1.493432	-0.430834
C	2.283600	-1.273956	-0.692298
H	-4.991149	-0.080271	-0.597363
H	-4.402508	2.337248	-0.505135
H	-2.056739	3.024026	-0.118964
C	3.427623	0.829931	-0.909189
C	3.431688	-0.545102	-1.034765
H	4.305753	1.412339	-1.160208
H	4.310547	-1.065956	-1.393382
H	0.095151	2.456787	0.284837

H	2.240313	2.575785	-0.427190
H	2.286007	-2.354990	-0.777548
H	-3.218680	-1.782301	-0.275934
O	2.881796	1.602590	1.771669
H	3.119250	0.660649	1.776250

Solvent = Pentyl Ethanoate
E = -2994.741650
G = -2994.593440
ZPC = 0.189055
TCE = 0.201967
TCH = 0.202911
TCG = 0.148210
N_{imag} = 1, -337.35 cm^{-1}

Se	-0.360407	-1.667067	0.423047
N	0.011220	1.430307	0.230211
C	1.134900	0.724965	-0.140224
C	1.153600	-0.678068	-0.195167
C	-3.969648	0.255970	-0.411408
C	-3.624542	1.603460	-0.368447
C	-2.303552	1.981629	-0.168185
C	-1.310430	1.016720	0.021054
C	-1.664861	-0.335946	0.002793
C	-2.983612	-0.709619	-0.235325
C	2.325302	1.438263	-0.423679
C	2.298116	-1.342154	-0.605066
H	-4.996649	-0.044530	-0.579256
H	-4.382229	2.366423	-0.502421
H	-2.028541	3.031195	-0.150894
C	3.473316	0.741946	-0.851488
C	3.466710	-0.634255	-0.928034
H	4.363449	1.307969	-1.097592
H	4.351244	-1.175033	-1.241078
H	0.141191	2.432962	0.282755
H	2.268836	2.512330	-0.542827
H	2.290831	-2.425297	-0.661003
H	-3.239569	-1.763026	-0.264388
O	2.802617	1.884206	1.591163
H	3.014011	0.974024	1.851365

TS[PSE-OH•] - Site 3
Solvent = Water
E = -2994.740899
G = -2994.593728
ZPC = 0.188707
TCE = 0.201747
TCH = 0.202691
TCG = 0.147171
N_{imag} = 1, -281.92 cm^{-1}

Se	-0.406693	-1.639129	0.302789
N	-0.157819	1.459258	0.528292
C	0.988613	0.857300	-0.001722
C	1.054409	-0.540952	-0.199370
C	-4.076164	0.227959	-0.372096
C	-3.778579	1.577424	-0.196197
C	-2.477905	1.979619	0.076712
C	-1.455500	1.034823	0.204572
C	-1.762085	-0.318441	0.040789
C	-3.061151	-0.716591	-0.263062
C	2.100417	1.628606	-0.299022
C	2.214165	-1.133239	-0.701943
H	-5.087534	-0.088661	-0.595066
H	-4.559178	2.323937	-0.282537
H	-2.237686	3.030444	0.200794
C	3.297730	1.027791	-0.723511
C	3.330396	-0.362363	-0.972597
H	4.108950	1.654967	-1.066890
H	4.234079	-0.820912	-1.353320
H	-0.076733	2.467910	0.592770
H	2.052086	2.703350	-0.161906
H	2.232641	-2.203599	-0.870589
H	-3.275948	-1.770357	-0.401008
O	4.440138	0.802237	1.108806
H	3.774423	0.228985	1.519113

Solvent = Pentyl Ethanoate
E = -2994.737757
G = -2994.589508
ZPC = 0.189118
TCE = 0.201951
TCH = 0.202895
TCG = 0.148249
N_{imag} = 1, -441.09 cm^{-1}

Se	-0.425835	-1.640731	0.329255
N	-0.145142	1.441451	0.539995
C	1.000579	0.830132	0.011978
C	1.050874	-0.565079	-0.187132
C	-4.067162	0.261967	-0.411540
C	-3.759567	1.605735	-0.218096
C	-2.458268	1.993584	0.072704
C	-1.445533	1.039057	0.205301
C	-1.765132	-0.310677	0.030647
C	-3.062242	-0.692891	-0.296828
C	2.119771	1.589901	-0.287246
C	2.203263	-1.168295	-0.697507
H	-5.078508	-0.042705	-0.651237
H	-4.532461	2.360306	-0.306022
H	-2.214577	3.042369	0.208299
C	3.321059	0.978127	-0.702321
C	3.327988	-0.411590	-0.967185
H	4.116620	1.599612	-1.088782
H	4.226619	-0.880238	-1.347792
H	-0.049515	2.444623	0.639999
H	2.087424	2.665191	-0.148581
H	2.207473	-2.238028	-0.873480
H	-3.285819	-1.743290	-0.447481
O	4.424984	0.906948	1.025172
H	3.823468	0.331462	1.522421

TS[PSE-OH•] - Site 4
Solvent = Water
E = -2994.746464
G = -2994.598093
ZPC = 0.189654
TCE = 0.202452
TCH = 0.203396
TCG = 0.148370
N_{imag} = 1, -339.57 cm^{-1}

Se	-0.314016	-1.603138	-0.144589
N	-0.296814	1.608085	-0.134368
C	0.922824	1.029564	-0.258430
C	1.127145	-0.379284	-0.263106

C	-4.120166	-0.032981	0.295660
C	-3.928769	1.350070	0.281556
C	-2.655641	1.870750	0.140526
C	-1.550149	1.018445	0.010132
C	-1.741430	-0.366604	0.025203
C	-3.030979	-0.881505	0.168481
C	2.045759	1.885434	-0.408538
C	2.403362	-0.885923	-0.382985
H	-5.114557	-0.447495	0.405797
H	-4.773109	2.020885	0.380633
H	-2.493338	2.943341	0.128240
C	3.304948	1.369418	-0.519563
C	3.520355	-0.032670	-0.437645
H	4.152607	2.032441	-0.639425
H	4.487757	-0.441177	-0.687631
H	-0.300854	2.624335	-0.146075
H	1.874796	2.956099	-0.440565
H	2.551683	-1.959617	-0.400794
H	-3.172619	-1.956546	0.179328
O	4.290534	-0.056090	1.651045
H	3.434078	0.258261	1.960971

Solvent = Pentyl Ethanoate
E = -2994.740834
G = -2994.592399
ZPC = 0.189361
TCE = 0.202198
TCH = 0.203142
TCG = 0.148435
N_{imag} = 1, -344.97 cm^{-1}

Se	-0.346403	-1.641958	-0.401366
N	-0.306524	1.445837	-0.838542
C	0.947417	0.924503	-0.575127
C	1.133004	-0.438789	-0.267415
C	-3.908431	0.125858	0.873435
C	-3.732457	1.463885	0.532626
C	-2.532498	1.893006	-0.018227
C	-1.500311	0.983757	-0.264272
C	-1.687351	-0.362992	0.059947
C	-2.878444	-0.780884	0.646103
C	2.070976	1.769367	-0.638635
C	2.394186	-0.920553	0.018244
H	-4.838180	-0.212054	1.314514
H	-4.525702	2.181323	0.706257
H	-2.383888	2.939009	-0.266788
C	3.332910	1.278521	-0.383806
C	3.514924	-0.063831	0.011124
H	4.190266	1.937974	-0.440533
H	4.508601	-0.484689	0.062170
H	-0.308231	2.437753	-1.042822
H	1.924755	2.814114	-0.893299
H	2.526585	-1.967968	0.264270
H	-3.002343	-1.825490	0.909630
O	3.756781	0.258743	2.089550
H	2.853710	0.584710	2.222476

TS[PSE-OH•] - Site 5
Solvent = Water
E = -2994.740435
G = -2994.591458
ZPC = 0.189535
TCE = 0.202229
TCH = 0.203173
TCG = 0.148976
N_{imag} = 1, -338.68 cm^{-1}

Se	-0.063018	-1.538011	0.118812
N	-0.340156	1.474762	0.810149
C	0.888090	1.157984	0.204994
C	1.183525	-0.142108	-0.196551
C	-3.967699	-0.192477	-0.519751
C	-3.904229	1.142186	-0.127661
C	-2.699222	1.696170	0.286289
C	-1.542711	0.913827	0.343291
C	-1.614934	-0.428697	-0.038700
C	-2.815411	-0.971891	-0.486336
C	1.855024	2.155327	0.031625
C	2.453985	-0.455640	-0.729067
H	-4.903145	-0.624994	-0.852614
H	-4.793095	1.761339	-0.153621
H	-2.641605	2.740111	0.576467
C	3.103889	1.861875	-0.520954
C	3.401761	0.575614	-0.925645
H	3.828938	2.657019	-0.643461
H	4.361252	0.334204	-1.365129
H	-0.428354	2.465191	1.009439
H	1.620096	3.168534	0.341577
H	2.600624	-1.417888	-1.201302
H	-2.848276	-2.011217	-0.793186
O	3.306505	-1.308615	0.988988
H	3.222823	-0.507351	1.530067

Solvent = Pentyl Ethanoate
E = -2994.738078
G = -2994.589247
ZPC = 0.189373
TCE = 0.202112
TCH = 0.203056
TCG = 0.148831
N_{imag} = 1, -448.69 cm^{-1}

Se	-0.049152	-1.521946	0.131779
N	-0.351085	1.491047	0.780778
C	0.888470	1.177057	0.202729
C	1.188010	-0.123260	-0.198147
C	-3.966416	-0.215350	-0.522549
C	-3.915917	1.119621	-0.132449
C	-2.714829	1.687048	0.274155
C	-1.549773	0.917427	0.328328
C	-1.609337	-0.428141	-0.048231
C	-2.805837	-0.982329	-0.491094
C	1.860448	2.172249	0.050261
C	2.464507	-0.443137	-0.715393
H	-4.898594	-0.658101	-0.851512
H	-4.811747	1.729090	-0.155296
H	-2.669941	2.732977	0.560884
C	3.118923	1.872884	-0.478801
C	3.417908	0.589041	-0.888997
H	3.850963	2.664690	-0.583449
H	4.383924	0.347550	-1.313931
H	-0.443110	2.471475	1.015823
H	1.625666	3.186662	0.356779
H	2.598322	-1.386601	-1.227159
H	-2.829621	-2.022520	-0.796696
O	3.232627	-1.396297	0.906318

H	3.204957	-0.644656	1.519227

TS[PSE-OH$^{\bullet}$] - Site 5a
Solvent = Water
E = -2994.748514
G = -2994.598459
ZPC = 0.189873
TCE = 0.202480
TCH = 0.203425
TCG = 0.150055
N_{imag} = 1, -182.96 cm^{-1}

Se	-0.065760	-1.591535	-0.330468
N	-0.075060	1.587604	-0.433926
C	1.152589	1.029988	-0.275817
C	1.324253	-0.353967	0.032011
C	-3.881302	-0.040260	0.208455
C	-3.695621	1.339558	0.114983
C	-2.427925	1.858580	-0.083944
C	-1.321415	1.006935	-0.201154
C	-1.508746	-0.376629	-0.107269
C	-2.789194	-0.889562	0.101151
C	2.293788	1.857643	-0.365705
C	2.639633	-0.887484	0.085188
H	-4.870321	-0.451539	0.368219
H	-4.540072	2.012227	0.202198
H	-2.272900	2.930149	-0.152533
C	3.550787	1.322860	-0.231311
C	3.729444	-0.064286	-0.013855
H	4.416401	1.969960	-0.304585
H	4.729283	-0.473898	0.057787
H	-0.085847	2.593700	-0.570301
H	2.152770	2.917352	-0.549146
H	2.757887	-1.954758	0.233570
H	-2.922482	-1.962887	0.177436
O	0.913192	0.006617	2.116539
H	1.693258	0.545458	2.302040

Solvent = Pentyl Ethanoate
E = -2994.740871
G = -2994.592127
ZPC = 0.189156
TCE = 0.202029
TCH = 0.202974
TCG = 0.148744
N_{imag} = 1, -264.34 cm^{-1}

Se	-0.064566	-1.597296	-0.392509
N	-0.068096	1.542474	-0.600914
C	1.164929	1.008989	-0.328037
C	1.327195	-0.358496	0.016559
C	-3.849124	-0.014246	0.346662
C	-3.663724	1.353637	0.169346
C	-2.403380	1.853891	-0.125757
C	-1.311424	0.993205	-0.269693
C	-1.503239	-0.381989	-0.102308
C	-2.763864	-0.874584	0.218951
C	2.299339	1.838513	-0.369696
C	2.629343	-0.873920	0.206771
H	-4.827741	-0.410927	0.587880
H	-4.498074	2.037402	0.271412
H	-2.251379	2.921579	-0.248151
C	3.555825	1.318618	-0.134016
C	3.728472	-0.046548	0.156618
H	4.418009	1.973951	-0.175905
H	4.720104	-0.446520	0.327849
H	-0.075740	2.543751	-0.750797
H	2.169286	2.890790	-0.600459
H	2.740469	-1.931712	0.417566
H	-2.894360	-1.941241	0.364779
O	0.783952	0.067238	2.040984
H	1.637658	0.433354	2.317268

PSE-OOH$^{\bullet}$ - Site 2a
Solvent = Water
E = -3069.901391
G = -3069.746922
ZPC = 0.195462
TCE = 0.208867
TCH = 0.209811
TCG = 0.154469
N_{imag} = 0

Se	-0.220713	-1.694631	-0.036258
N	-0.085501	1.462507	-0.624784
C	1.063399	0.896714	0.022413
C	1.247978	-0.557175	-0.310925
C	-3.989448	0.030653	0.047164
C	-3.758210	1.373028	-0.253031
C	-2.465564	1.829453	-0.456520
C	-1.370995	0.958813	-0.375144
C	-1.611032	-0.390718	-0.089639
C	-2.914152	-0.842160	0.128213
C	2.278989	1.718367	-0.273521
C	2.467267	-1.070549	-0.686002
H	-4.995692	-0.332636	0.215205
H	-4.585881	2.069130	-0.319916
H	-2.279966	2.875768	-0.677812
C	3.457348	1.163321	-0.637244
C	3.583455	-0.241707	-0.835654
H	4.320634	1.798641	-0.799439
H	4.534371	-0.664273	-1.132215
H	-0.055942	2.476943	-0.644554
H	2.166252	2.789086	-0.134424
H	2.557312	-2.133229	-0.884895
H	-3.078566	-1.890350	0.354536
O	0.745757	1.027251	1.462428
O	1.769978	0.430961	2.238164
H	1.460146	-0.483116	2.344386

Solvent = Pentyl Ethanoate
E = -3069.899436
G = -3069.745381
ZPC = 0.195213
TCE = 0.208726
TCH = 0.209671
TCG = 0.154054
N_{imag} = 0

Se	-0.221884	-1.683054	-0.015284
N	-0.076625	1.490572	-0.516811
C	1.077260	0.891614	0.089560
C	1.240780	-0.545299	-0.330561
C	-3.995064	0.035706	-0.022397
C	-3.756166	1.382816	-0.291326
C	-2.459134	1.844724	-0.441652

C	-1.363317	0.976971	-0.334957
C	-1.612531	-0.378954	-0.086075
C	-2.920783	-0.835815	0.077405
C	2.294170	1.716555	-0.200595
C	2.426520	-1.038381	-0.815360
H	-5.005658	-0.332176	0.105096
H	-4.582634	2.078791	-0.375131
H	-2.270152	2.895573	-0.637604
C	3.442812	1.179874	-0.670781
C	3.540264	-0.205968	-0.979133
H	4.305859	1.817128	-0.827787
H	4.466489	-0.612632	-1.363643
H	-0.037423	2.502298	-0.547596
H	2.210702	2.773428	0.033316
H	2.494661	-2.085528	-1.091298
H	-3.092555	-1.888559	0.276279
O	0.780012	0.958670	1.526827
O	1.800496	0.303834	2.258041
H	1.458216	-0.601606	2.321989

PSE-OOH• - Site 2
Solvent = Water
E = -3069.902391
G = -3069.748357
ZPC = 0.195987
TCE = 0.209605
TCH = 0.210549
TCG = 0.154034
N_{imag} = 0

Se	-0.659170	-1.709577	0.446385
N	-0.044242	1.347541	0.093336
C	0.997626	0.519062	-0.246300
C	0.911885	-0.863855	-0.254357
C	-4.129523	0.477941	-0.331305
C	-3.674348	1.793418	-0.372152
C	-2.319936	2.068289	-0.238819
C	-1.404466	1.031794	-0.037823
C	-1.864680	-0.287104	0.023106
C	-3.219909	-0.558187	-0.143318
C	2.290911	1.219114	-0.558136
C	1.977426	-1.651079	-0.681773
H	-5.183687	0.256543	-0.444255
H	-4.371760	2.609365	-0.518894
H	-1.954045	3.088480	-0.287739
C	3.354898	0.309608	-1.067338
C	3.193797	-1.033802	-1.100115
H	4.278958	0.777516	-1.386588
H	3.998020	-1.666150	-1.459628
H	0.162896	2.339638	0.046405
H	2.108403	2.052580	-1.249016
H	1.882801	-2.729472	-0.694171
H	-3.563188	-1.586034	-0.107740
O	2.746814	1.973078	0.607515
O	3.057173	1.068135	1.658319
H	2.249077	1.069466	2.194491

Solvent = Pentyl Ethanoate
E = -3069.902131
G = -3069.748118
ZPC = 0.195818
TCE = 0.209432
TCH = 0.210376
TCG = 0.154014
N_{imag} = 0

Se	-0.675292	-1.721818	0.459134
N	-0.013712	1.316293	0.108620
C	1.013266	0.468772	-0.250017
C	0.910413	-0.907537	-0.242580
C	-4.108181	0.512217	-0.357761
C	-3.633825	1.820099	-0.395680
C	-2.276611	2.074653	-0.250559
C	-1.378922	1.026086	-0.036938
C	-1.858883	-0.286011	0.019908
C	-3.215548	-0.536760	-0.161097
C	2.302969	1.161186	-0.601891
C	1.978573	-1.714101	-0.636409
H	-5.164412	0.306249	-0.481241
H	-4.317981	2.645874	-0.550741
H	-1.896942	3.089971	-0.300703
C	3.398002	0.223999	-0.979361
C	3.221803	-1.117599	-0.996471
H	4.348659	0.674172	-1.240947
H	4.042232	-1.765053	-1.285674
H	0.212626	2.303305	0.064913
H	2.121265	1.895645	-1.401327
H	1.869421	-2.791226	-0.642678
H	-3.574490	-1.559645	-0.131623
O	2.684352	2.061778	0.470426
O	2.965262	1.303555	1.636941
H	2.100310	1.255769	2.073324

PSE-OOH• - Site 3
Solvent = Water
E = -3069.899600
G = -3069.746449
ZPC = 0.195623
TCE = 0.209358
TCH = 0.210302
TCG = 0.153151
N_{imag} = 0

Se	-0.739507	-1.660650	0.295080
N	-0.298988	1.436094	0.396881
C	0.813985	0.745260	-0.108860
C	0.771690	-0.681755	-0.237414
C	-4.318874	0.398114	-0.266472
C	-3.932795	1.734304	-0.176192
C	-2.599299	2.068689	0.013654
C	-1.624824	1.071650	0.138951
C	-2.020652	-0.266972	0.056392
C	-3.356266	-0.598862	-0.157773
C	1.945236	1.424668	-0.440333
C	1.910167	-1.378614	-0.722115
H	-5.357071	0.133230	-0.423735
H	-4.671403	2.522374	-0.263461
H	-2.293093	3.107886	0.075334
C	3.210478	0.739231	-0.818865
C	3.062355	-0.730757	-1.023265
H	3.665039	1.210832	-1.698399
H	3.923447	-1.274094	-1.394860
H	-0.160555	2.440280	0.410361
H	1.960551	2.507960	-0.371287
H	1.838316	-2.452768	-0.856594
H	-3.639123	-1.643274	-0.229796

O	4.266720	1.011878	0.161279
O	3.913709	0.436784	1.410385
H	3.359421	1.117986	1.821995

Solvent = Pentyl Ethanoate
E = -3069.898905
G = -3069.745480
ZPC = 0.195666
TCE = 0.209328
TCH = 0.210272
TCG = 0.153424
N_{imag} = 0

Se	-0.746178	-1.656055	0.286775
N	-0.305292	1.442879	0.347213
C	0.815435	0.744514	-0.122574
C	0.768740	-0.683886	-0.245200
C	-4.333301	0.395459	-0.237716
C	-3.948197	1.731764	-0.157002
C	-2.612573	2.068374	0.009007
C	-1.633001	1.074475	0.120764
C	-2.028859	-0.265178	0.050530
C	-3.366992	-0.598595	-0.141439
C	1.958291	1.415131	-0.432757
C	1.913397	-1.387927	-0.701646
H	-5.373436	0.128357	-0.378076
H	-4.689497	2.518573	-0.233722
H	-2.310314	3.109430	0.061488
C	3.227224	0.724924	-0.801755
C	3.074399	-0.748548	-0.983861
H	3.664720	1.178021	-1.701016
H	3.942582	-1.300986	-1.324427
H	-0.164129	2.444752	0.383908
H	1.975680	2.499845	-0.379435
H	1.842137	-2.463778	-0.824135
H	-3.649701	-1.643772	-0.206950
O	4.286161	1.022780	0.153378
O	3.955755	0.454465	1.410027
H	3.326346	1.094254	1.776189

PSE-OOH• - Site 4
Solvent = Water
E = -3069.902963
G = -3069.749263
ZPC = 0.196042
TCE = 0.209713
TCH = 0.210657
TCG = 0.153700
N_{imag} = 0

Se	-0.502174	-1.591159	0.068250
N	-0.546270	1.573840	0.326527
C	0.665520	1.059815	-0.071930
C	0.885460	-0.330610	-0.306604
C	-4.375579	-0.080757	-0.120703
C	-4.205404	1.288783	0.076641
C	-2.932558	1.819907	0.215942
C	-1.804623	0.990152	0.175973
C	-1.978460	-0.386110	-0.007273
C	-3.260480	-0.908941	-0.167248
C	1.743802	1.964254	-0.298829
C	2.090540	-0.793551	-0.723988
H	-5.366393	-0.501559	-0.239349
H	-5.064697	1.947600	0.112478
H	-2.791903	2.886776	0.356594
C	2.967317	1.536401	-0.691682
C	3.289353	0.086774	-0.816711
H	3.761584	2.247365	-0.888082
H	3.872586	-0.122757	-1.720030
H	-0.562124	2.581139	0.443138
H	1.546129	3.026069	-0.182824
H	2.231855	-1.849813	-0.926245
H	-3.380753	-1.976020	-0.319278
O	4.289625	-0.298042	0.193786
O	3.741802	-0.132067	1.493127
H	3.890776	0.807897	1.680581

Solvent = Pentyl Ethanoate
E = -3069.901698
G = -3069.748142
ZPC = 0.195920
TCE = 0.209631
TCH = 0.210575
TCG = 0.153557
N_{imag} = 0

Se	-0.498688	-1.583109	0.077493
N	-0.564230	1.574978	0.334320
C	0.656318	1.070633	-0.053408
C	0.881856	-0.317605	-0.302901
C	-4.380897	-0.099034	-0.137982
C	-4.221213	1.269537	0.068955
C	-2.952416	1.807904	0.215996
C	-1.818597	0.986734	0.177100
C	-1.982468	-0.390185	-0.013205
C	-3.259894	-0.919048	-0.184028
C	1.735296	1.977942	-0.254410
C	2.087042	-0.771988	-0.722274
H	-5.368373	-0.525718	-0.264311
H	-5.085278	1.922324	0.105336
H	-2.821747	2.875516	0.363218
C	2.961633	1.558711	-0.650681
C	3.286299	0.111295	-0.819392
H	3.755498	2.275599	-0.829307
H	3.833219	-0.066436	-1.753438
H	-0.583770	2.576948	0.480361
H	1.540330	3.038061	-0.116759
H	2.234208	-1.827410	-0.925489
H	-3.373070	-1.985844	-0.344533
O	4.314196	-0.296636	0.135550
O	3.799111	-0.195531	1.453465
H	3.909770	0.745773	1.655196

PSE-OOH• - Site 5
Solvent = Water
E = -3069.899158
G = -3069.745427
ZPC = 0.195538
TCE = 0.209147
TCH = 0.210091
TCG = 0.153730
N_{imag} = 0

Se	-0.146848	-1.460975	0.117719
N	-0.632065	1.519401	0.817443
C	0.614349	1.281342	0.202253

C	0.986567	0.026156	-0.196295
C	-4.140194	-0.387659	-0.511968
C	-4.168119	0.945408	-0.110248
C	-3.002968	1.578803	0.304109
C	-1.794437	0.878596	0.352597
C	-1.775434	-0.463826	-0.035927
C	-2.936503	-1.085687	-0.485257
C	1.504345	2.370163	0.037303
C	2.315451	-0.258982	-0.818778
H	-5.044641	-0.881998	-0.844569
H	-5.097872	1.501601	-0.128862
H	-3.017659	2.621871	0.602452
C	2.792679	2.165796	-0.513868
C	3.200724	0.939146	-0.927593
H	3.456141	3.017602	-0.610360
H	4.185807	0.780401	-1.351651
H	-0.787053	2.503981	1.004030
H	1.194362	3.358121	0.357207
H	2.181997	-0.728576	-1.803787
H	-2.898019	-2.123276	-0.797437
O	2.985822	-1.341661	-0.120236
O	3.211583	-0.959187	1.229172
H	4.086200	-0.541160	1.198958

Solvent = Pentyl Ethanoate
E = -3069.898271
G = -3069.744735
ZPC = 0.195294
TCE = 0.208968
TCH = 0.209912
TCG = 0.153535
N_{imag} = 0

Se	-0.138898	-1.444464	0.108977
N	-0.640386	1.546931	0.763665
C	0.615880	1.301421	0.180666
C	0.991595	0.038968	-0.202224
C	-4.146135	-0.411945	-0.485093
C	-4.183825	0.922739	-0.093065
C	-3.019967	1.573333	0.297027
C	-1.801334	0.889375	0.331931
C	-1.773398	-0.457689	-0.042759
C	-2.933714	-1.094953	-0.469573
C	1.516043	2.379361	0.032552
C	2.327701	-0.256370	-0.806164
H	-5.049456	-0.919736	-0.800382
H	-5.120593	1.467653	-0.100932
H	-3.046296	2.618937	0.586730
C	2.820748	2.161864	-0.478742
C	3.230577	0.932889	-0.879307
H	3.494008	3.008405	-0.555968
H	4.224612	0.768558	-1.281320
H	-0.792947	2.524136	0.979718
H	1.207761	3.372649	0.338125
H	2.197612	-0.696136	-1.806581
H	-2.888125	-2.134881	-0.773839
O	2.965351	-1.363947	-0.128011
O	3.170489	-1.015743	1.233238
H	4.026906	-0.562744	1.210259

PSE-OOH$^{\bullet}$ - Site 5a
Solvent = Water
E = -3069.907307
G = -3069.753064
ZPC = 0.195262
TCE = 0.208863
TCH = 0.209807
TCG = 0.154244
N_{imag} = 0

Se	-0.100070	-1.600225	-0.387677
N	-0.179574	1.564708	-0.514329
C	1.065982	0.997895	-0.439134
C	1.213831	-0.272394	0.322355
C	-3.966101	-0.119575	0.128399
C	-3.799620	1.262682	0.032209
C	-2.538144	1.801577	-0.155780
C	-1.414459	0.969684	-0.269965
C	-1.584339	-0.419779	-0.179680
C	-2.857539	-0.950197	0.031073
C	2.165268	1.627509	-1.009616
C	2.582977	-0.836130	0.301564
H	-4.949166	-0.546318	0.284563
H	-4.655021	1.922982	0.110358
H	-2.399670	2.875414	-0.226107
C	3.431452	1.062769	-0.943649
C	3.617494	-0.189041	-0.290577
H	4.273663	1.565823	-1.400251
H	4.607367	-0.630498	-0.264321
H	-0.213997	2.520311	-0.851938
H	2.005351	2.568789	-1.525852
H	2.718378	-1.783956	0.810509
H	-2.973533	-2.025118	0.113189
O	0.715124	-0.130959	1.681503
O	1.506285	0.846481	2.355525
H	0.973955	1.653064	2.271740

Solvent = Pentyl Ethanoate
E = -3069.906722
G = -3069.752839
ZPC = 0.195067
TCE = 0.208742
TCH = 0.209686
TCG = 0.153883
N_{imag} = 0

Se	-0.063999	-1.602764	-0.348449
N	-0.174432	1.574673	-0.470034
C	1.069888	0.991055	-0.442300
C	1.224982	-0.241977	0.375158
C	-3.956833	-0.163425	0.061172
C	-3.805944	1.220275	-0.022668
C	-2.545970	1.773953	-0.174803
C	-1.410431	0.957571	-0.268149
C	-1.563459	-0.434325	-0.187264
C	-2.835549	-0.978979	-0.010972
C	2.147987	1.564218	-1.097961
C	2.599695	-0.788121	0.393596
H	-4.937885	-0.603619	0.191573
H	-4.671037	1.869933	0.038488
H	-2.420362	2.850557	-0.230820
C	3.414184	0.994155	-1.030615
C	3.618644	-0.194603	-0.276803
H	4.242667	1.453369	-1.553986
H	4.611949	-0.626513	-0.228421
H	-0.216491	2.504467	-0.869473

H	1.980785	2.466032	-1.678677
H	2.750466	-1.682288	0.988089
H	-2.940860	-2.055345	0.068764
O	0.680608	-0.074404	1.700831
O	1.377142	0.993093	2.341068
H	0.792597	1.746387	2.166432

TS[PSE-OOH$^{\bullet}$] - Site 2a

Solvent = Water
E = -3069.888869
G = -3069.736652
ZPC = 0.194014
TCE = 0.207546
TCH = 0.208490
TCG = 0.152217
N_{imag} = 1, -462.45 cm^{-1}

Se	0.268021	1.744026	-0.133588
N	0.148352	-1.337549	-0.903652
C	-1.055065	-0.817503	-0.443381
C	-1.224454	0.604083	-0.278895
C	3.963149	-0.089856	0.345266
C	3.739463	-1.401760	-0.067674
C	2.471270	-1.803834	-0.461227
C	1.404795	-0.899505	-0.464047
C	1.636706	0.418968	-0.063507
C	2.908008	0.814029	0.349436
C	-2.235542	-1.589049	-0.740310
C	-2.496493	1.141691	-0.169191
H	4.948387	0.228025	0.663024
H	4.551949	-2.118489	-0.075627
H	2.287674	-2.827341	-0.771763
C	-3.478050	-1.031897	-0.638422
C	-3.622020	0.338819	-0.332746
H	-4.357779	-1.639641	-0.811590
H	-4.609312	0.775647	-0.250050
H	0.110331	-2.333253	-1.092396
H	-2.102092	-2.636612	-0.988139
H	-2.611320	2.203148	0.020621
H	3.067667	1.838468	0.667567
O	-0.829475	-1.290624	1.397921
O	-1.894189	-0.903930	2.211867
H	-1.717958	0.027325	2.415795

Solvent = Pentyl Ethanoate
E = -3069.884667
G = -3069.732222
ZPC = 0.193895
TCE = 0.207444
TCH = 0.208388
TCG = 0.152445
N_{imag} = 1, -597.12 cm^{-1}

Se	0.283240	1.758532	-0.139698
N	0.144893	-1.287980	-0.957075
C	-1.060699	-0.793145	-0.447849
C	-1.224766	0.624474	-0.237445
C	3.952799	-0.120149	0.392349
C	3.722853	-1.418440	-0.054300
C	2.456792	-1.798549	-0.477478
C	1.400149	-0.883943	-0.483140
C	1.641302	0.422479	-0.049773
C	2.905798	0.794165	0.398403
C	-2.248091	-1.538978	-0.783223
C	-2.488814	1.155649	-0.064651
H	4.935174	0.179765	0.735911
H	4.527700	-2.144055	-0.062931
H	2.270145	-2.814545	-0.810031
C	-3.488080	-0.987008	-0.624605
C	-3.623105	0.360215	-0.236947
H	-4.372111	-1.584764	-0.811509
H	-4.607034	0.792592	-0.103515
H	0.102058	-2.275321	-1.179600
H	-2.124349	-2.573266	-1.085917
H	-2.598435	2.209014	0.170838
H	3.069665	1.808776	0.745453
O	-0.831787	-1.358652	1.305315
O	-1.893869	-1.035719	2.129035
H	-1.718785	-0.118063	2.387706

TS[PSE-OOH$^{\bullet}$] - Site 2

Solvent = Water
E = -3069.888468
G = -3069.735604
ZPC = 0.194665
TCE = 0.208225
TCH = 0.209169
TCG = 0.152865
N_{imag} = 1, -507.82 cm^{-1}

Se	-0.552998	-1.676433	0.368004
N	-0.129913	1.425308	0.028655
C	0.952915	0.672379	-0.346653
C	0.937148	-0.730554	-0.368547
C	-4.171774	0.318173	-0.211056
C	-3.794689	1.657588	-0.265689
C	-2.453369	2.008347	-0.195062
C	-1.471918	1.024761	-0.045925
C	-1.852795	-0.318104	0.026248
C	-3.197080	-0.665198	-0.074942
C	2.180015	1.359253	-0.627597
C	2.039424	-1.428669	-0.827083
H	-5.215850	0.037393	-0.274604
H	-4.543414	2.433025	-0.373821
H	-2.147892	3.047749	-0.253608
C	3.256537	0.620435	-1.201786
C	3.199390	-0.745476	-1.261350
H	4.135720	1.160707	-1.530234
H	4.032678	-1.316760	-1.652187
H	0.011022	2.428495	-0.024048
H	2.113453	2.424717	-0.823741
H	2.011013	-2.511889	-0.855762
H	-3.479074	-1.711206	-0.031031
O	2.773171	1.730604	1.134590
O	3.079272	0.540045	1.786101
H	2.231291	0.226547	2.137463

Solvent = Pentyl Ethanoate
E = -3069.886221
G = -3069.733646
ZPC = 0.194396
TCE = 0.207955
TCH = 0.208900
TCG = 0.152575
N_{imag} = 1, -600.27 cm^{-1}

Se	-0.551276	-1.671510	0.395258
N	-0.118345	1.408822	0.073535
C	0.959229	0.654899	-0.342222
C	0.940706	-0.741311	-0.359605
C	-4.160702	0.321719	-0.260746
C	-3.780334	1.660078	-0.292657
C	-2.439835	2.008321	-0.194335
C	-1.461767	1.023335	-0.034259
C	-1.848657	-0.319265	0.020035
C	-3.189794	-0.664070	-0.115238
C	2.184214	1.342058	-0.633915
C	2.039984	-1.449672	-0.823891
H	-5.203912	0.043732	-0.348233
H	-4.526017	2.437980	-0.406251
H	-2.135977	3.049257	-0.237552
C	3.256736	0.594587	-1.207641
C	3.197472	-0.772622	-1.260576
H	4.138340	1.130143	-1.537379
H	4.031820	-1.345476	-1.647178
H	0.033855	2.409824	0.050502
H	2.111573	2.400635	-0.864729
H	2.004836	-2.532891	-0.849714
H	-3.473564	-1.710392	-0.088898
O	2.766307	1.757889	1.079820
O	3.016512	0.594019	1.782159
H	2.144790	0.323173	2.110366

TS[PSE-OOH•] - Site 3

Solvent = Water
E = -3069.885453
G = -3069.733353
ZPC = 0.194065
TCE = 0.207671
TCH = 0.208615
TCG = 0.152101
N_{imag} = 1, -571.10 cm^{-1}

Se	-0.610160	-1.642268	0.261911
N	-0.295282	1.472841	0.315252
C	0.819005	0.815777	-0.215256
C	0.841152	-0.599686	-0.341217
C	-4.289453	0.262311	-0.187606
C	-3.958328	1.613705	-0.113675
C	-2.633850	2.005187	0.025680
C	-1.615849	1.050486	0.118623
C	-1.955521	-0.303650	0.055316
C	-3.281908	-0.692946	-0.112272
C	1.930169	1.538740	-0.590905
C	1.966058	-1.244724	-0.882714
H	-5.320479	-0.047289	-0.305425
H	-4.732593	2.369269	-0.174776
H	-2.368836	3.056376	0.071550
C	3.137416	0.877232	-0.952452
C	3.085841	-0.526870	-1.221915
H	3.911198	1.463908	-1.433612
H	3.955586	-1.022556	-1.634453
H	-0.194521	2.481564	0.321185
H	1.912606	2.620570	-0.514797
H	1.934381	-2.318203	-1.031103
H	-3.522247	-1.748653	-0.171946
O	4.139516	0.866251	0.651181
O	3.386671	0.254148	1.641574
H	2.759446	0.935680	1.929943

Solvent = Pentyl Ethanoate
E = -3069.883318
G = -3069.730823
ZPC = 0.194298
TCE = 0.207831
TCH = 0.208775
TCG = 0.152495
N_{imag} = 1, -632.26 cm^{-1}

Se	-0.604222	-1.637437	0.273610
N	-0.309084	1.459020	0.382725
C	0.812097	0.826930	-0.171460
C	0.837199	-0.580883	-0.344088
C	-4.288818	0.253101	-0.234707
C	-3.966305	1.602536	-0.118208
C	-2.646878	1.995831	0.060216
C	-1.626866	1.043800	0.153092
C	-1.959259	-0.310292	0.050281
C	-3.278558	-0.699601	-0.160494
C	1.918424	1.565342	-0.532203
C	1.948535	-1.201676	-0.934723
H	-5.315526	-0.057219	-0.385187
H	-4.742728	2.356220	-0.178086
H	-2.391692	3.047966	0.135815
C	3.125916	0.917933	-0.931198
C	3.062248	-0.470766	-1.267376
H	3.882951	1.525743	-1.413741
H	3.924968	-0.950568	-1.712192
H	-0.210386	2.464949	0.444225
H	1.902444	2.644789	-0.423691
H	1.914938	-2.268895	-1.123778
H	-3.513206	-1.754302	-0.254112
O	4.141154	0.860476	0.619040
O	3.460203	0.142506	1.578028
H	2.818121	0.773637	1.937602

TS[PSE-OOH•] - Site 4

Solvent = Water
E = -3069.890059
G = -3069.737415
ZPC = 0.194710
TCE = 0.208278
TCH = 0.209223
TCG = 0.152644
N_{imag} = 1, -443.00 cm^{-1}

Se	-0.471356	-1.600800	0.023275
N	-0.466726	1.567263	0.247287
C	0.717636	1.036595	-0.187553
C	0.903111	-0.351844	-0.413055
C	-4.331700	-0.044596	-0.027167
C	-4.136974	1.324563	0.140075
C	-2.851910	1.840471	0.220710
C	-1.740500	0.993811	0.152998
C	-1.937611	-0.381133	-0.003093
C	-3.230439	-0.889506	-0.105029
C	1.796984	1.919537	-0.443822
C	2.109248	-0.824511	-0.865077
H	-5.331854	-0.453848	-0.098296
H	-4.985359	1.995537	0.199347
H	-2.691807	2.907365	0.337882
C	3.006288	1.444939	-0.870447

C	3.236539	0.039421	-0.971592
H	3.816925	2.132275	-1.079456
H	4.102210	-0.309565	-1.521189
H	-0.467904	2.575981	0.355650
H	1.628431	2.984903	-0.324154
H	2.235452	-1.883856	-1.058392
H	-3.370346	-1.956931	-0.235363
O	4.170346	-0.282778	0.682768
O	3.299579	0.013068	1.730962
H	3.353995	0.975688	1.830064

Solvent = Pentyl Ethanoate
E = -3069.886511
G = -3069.734070
ZPC = 0.194576
TCE = 0.208156
TCH = 0.209100
TCG = 0.152441
N_{imag} = 1, -576.91 cm^{-1}

Se	-0.457216	-1.593657	0.073674
N	-0.475386	1.537319	0.385838
C	0.709969	1.041213	-0.112249
C	0.897730	-0.332954	-0.392236
C	-4.324618	-0.052474	-0.146943
C	-4.141230	1.308110	0.083233
C	-2.862325	1.821704	0.246252
C	-1.747272	0.979147	0.205437
C	-1.935082	-0.389763	-0.008796
C	-3.218379	-0.893437	-0.201161
C	1.769212	1.940844	-0.375789
C	2.087108	-0.782142	-0.908007
H	-5.319234	-0.458404	-0.285337
H	-4.993443	1.975958	0.125350
H	-2.712528	2.884363	0.410051
C	2.969150	1.491404	-0.861046
C	3.209925	0.091242	-1.011697
H	3.766371	2.192866	-1.076136
H	4.051184	-0.226854	-1.616041
H	-0.480432	2.538304	0.539126
H	1.598301	3.001796	-0.220369
H	2.213017	-1.833089	-1.142414
H	-3.349159	-1.954838	-0.381770
O	4.176680	-0.289410	0.552935
O	3.348938	-0.074782	1.639858
H	3.408912	0.879184	1.797415

TS[PSE-OOH$^{\bullet}$] - Site 5
Solvent = Water
E = -3069.884285
G = -3069.731194
ZPC = 0.194578
TCE = 0.208006
TCH = 0.208951
TCG = 0.153091
N_{imag} = 1, -587.88 cm^{-1}

Se	-0.238016	-1.526052	0.014087
N	-0.503780	1.481090	0.747955
C	0.693858	1.172807	0.079444
C	0.970886	-0.112539	-0.356962
C	-4.188069	-0.215946	-0.371654
C	-4.113755	1.116604	0.025002
C	-2.890864	1.680125	0.367810
C	-1.725153	0.909655	0.349061
C	-1.807542	-0.430889	-0.038130
C	-3.027904	-0.983447	-0.414037
C	1.647802	2.183132	-0.122582
C	2.248780	-0.431056	-0.911430
H	-5.137887	-0.656533	-0.648561
H	-5.008482	1.726905	0.058066
H	-2.826252	2.722369	0.662544
C	2.859404	1.911930	-0.779170
C	3.142656	0.645018	-1.223785
H	3.555288	2.723714	-0.953631
H	4.065973	0.421741	-1.743461
H	-0.589263	2.470757	0.952044
H	1.427986	3.187453	0.223813
H	2.333929	-1.350987	-1.479960
H	-3.069392	-2.021157	-0.725358
O	3.119713	-1.224553	0.519660
O	3.141637	-0.349482	1.591013
H	3.915715	0.213799	1.433080

Solvent = Pentyl Ethanoate
E = -3069.882464
G = -3069.729841
ZPC = 0.194321
TCE = 0.207839
TCH = 0.208784
TCG = 0.152622
N_{imag} = 1, -626.03 cm^{-1}

Se	-0.230137	-1.512837	0.006499
N	-0.510015	1.500549	0.701001
C	0.700159	1.185876	0.065281
C	0.977910	-0.102669	-0.366821
C	-4.192155	-0.232440	-0.355334
C	-4.124890	1.099912	0.040363
C	-2.902436	1.674755	0.364725
C	-1.729137	0.916303	0.329903
C	-1.805050	-0.427161	-0.051738
C	-3.025393	-0.988868	-0.411386
C	1.663855	2.187950	-0.115615
C	2.263517	-0.431780	-0.896704
H	-5.141515	-0.681810	-0.619831
H	-5.024999	1.701710	0.086409
H	-2.846415	2.718425	0.657444
C	2.886644	1.907301	-0.749385
C	3.168158	0.640869	-1.194936
H	3.590894	2.714986	-0.912277
H	4.097608	0.413909	-1.702534
H	-0.592861	2.480802	0.939696
H	1.446611	3.194798	0.225602
H	2.344057	-1.344617	-1.478089
H	-3.062076	-2.027473	-0.721279
O	3.088455	-1.241825	0.523208
O	3.091061	-0.383776	1.601640
H	3.860235	0.186407	1.447999

TS[PSE-OOH$^{\bullet}$] - Site 5a
Solvent = Water
E = -3069.890608
G = -3069.738403
ZPC = 0.193999
TCE = 0.207746

TCH = 0.208690
TCG = 0.152205
N_{imag} = 1, -402.32 cm^{-1}

Se	-0.224304	-1.648453	-0.351155
N	-0.180805	1.510895	-0.689742
C	1.052776	0.939468	-0.559244
C	1.211543	-0.408449	-0.095402
C	-3.977201	0.030504	0.300994
C	-3.768950	1.392833	0.091925
C	-2.503299	1.864278	-0.218909
C	-1.423084	0.981232	-0.339303
C	-1.634803	-0.386007	-0.130564
C	-2.907814	-0.850107	0.194797
C	2.195669	1.716857	-0.809151
C	2.522937	-0.973569	-0.097404
H	-4.962296	-0.344295	0.549886
H	-4.592079	2.091906	0.177093
H	-2.331823	2.924066	-0.376516
C	3.454801	1.168227	-0.694220
C	3.619244	-0.192480	-0.342548
H	4.325010	1.781832	-0.892801
H	4.613238	-0.620000	-0.294086
H	-0.182216	2.499740	-0.916236
H	2.064446	2.752066	-1.105839
H	2.627886	-2.024316	0.147974
H	-3.056126	-1.911197	0.361954
O	0.884022	-0.118179	1.802436
O	1.845696	0.742383	2.339658
H	1.497274	1.630987	2.173471

Solvent = Pentyl Ethanoate
E = -3069.885986
G = -3069.733903
ZPC = 0.193895
TCE = 0.207632
TCH = 0.208576
TCG = 0.152083
N_{imag} = 1, -556.84 cm^{-1}

Se	-0.219514	-1.639731	-0.385457
N	-0.186618	1.501613	-0.723259
C	1.057212	0.942527	-0.561866
C	1.214405	-0.399495	-0.081439
C	-3.967553	0.028948	0.343579
C	-3.767318	1.387190	0.112373
C	-2.506493	1.858374	-0.222553
C	-1.426031	0.978524	-0.352032
C	-1.632707	-0.387247	-0.128018
C	-2.895742	-0.849302	0.230209
C	2.195712	1.722615	-0.790294
C	2.526433	-0.959228	-0.066366
H	-4.946648	-0.345464	0.616209
H	-4.590947	2.085510	0.202191
H	-2.342927	2.918019	-0.391677
C	3.461265	1.179615	-0.652101
C	3.625786	-0.174549	-0.296586
H	4.330381	1.799341	-0.837411
H	4.620346	-0.597852	-0.225461
H	-0.192415	2.482208	-0.975103
H	2.068691	2.755639	-1.098241
H	2.632125	-2.006580	0.192999
H	-3.037298	-1.908046	0.417807
O	0.860733	-0.131888	1.775164
O	1.818466	0.694393	2.335521
H	1.485086	1.588914	2.172117

PSE-OOCH$_3^•$ - Site 2a
Solvent = Water
E = -3109.189268
G = -3109.008731
ZPC = 0.223626
TCE = 0.238452
TCH = 0.239397
TCG = 0.180537
N_{imag} = 0

Se	-0.328211	-1.712649	-0.290642
N	-0.233954	1.398187	-0.917300
C	0.959232	0.905535	-0.282673
C	1.153986	-0.572241	-0.479743
C	-4.042589	0.048290	0.268460
C	-3.837466	1.380952	-0.086916
C	-2.574000	1.818890	-0.455308
C	-1.490735	0.933896	-0.493594
C	-1.705861	-0.405589	-0.152124
C	-2.974361	-0.837637	0.237901
C	2.144426	1.704700	-0.731719
C	2.394639	-1.106390	-0.746812
H	-5.023890	-0.297927	0.568531
H	-4.661087	2.084734	-0.066558
H	-2.405668	2.858340	-0.718510
C	3.338061	1.132321	-1.005498
C	3.499887	-0.283446	-0.978627
H	4.184548	1.756221	-1.268997
H	4.466118	-0.723250	-1.188239
H	-0.211411	2.409071	-1.009944
H	2.003254	2.780716	-0.758818
H	2.505452	-2.183574	-0.817131
H	-3.119787	-1.878118	0.507826
O	0.728286	1.186170	1.152349
O	1.878555	0.798825	1.896515
C	1.471208	-0.203035	2.820607
H	2.365094	-0.416568	3.408146
H	0.682133	0.178186	3.472158
H	1.138788	-1.102514	2.299824

Solvent = Pentyl Ethanoate
E = -3109.189718
G = -3109.010104
ZPC = 0.223424
TCE = 0.238381
TCH = 0.239325
TCG = 0.179614
N_{imag} = 0

Se	-0.329197	-1.686275	-0.295075
N	-0.229885	1.462601	-0.768615
C	0.971260	0.917725	-0.194488
C	1.143993	-0.541935	-0.525252
C	-4.069058	0.033283	0.194651
C	-3.859499	1.381185	-0.092849
C	-2.586662	1.841381	-0.391072
C	-1.492356	0.967630	-0.424821
C	-1.713911	-0.387943	-0.155270
C	-2.993592	-0.842821	0.163935

```
C     2.150554   1.739941  -0.624443
C     2.350441  -1.056760  -0.937591
H    -5.058792  -0.331997   0.439694
H    -4.688263   2.079251  -0.074039
H    -2.418072   2.893078  -0.600854
C     3.312315   1.185894  -1.035669
C     3.449610  -0.224870  -1.170374
H     4.153041   1.824805  -1.282123
H     4.389813  -0.650409  -1.496407
H    -0.202905   2.473534  -0.832489
H     2.036317   2.814410  -0.518968
H     2.438804  -2.122783  -1.122047
H    -3.142862  -1.895974   0.378544
O     0.775341   1.091353   1.247680
O     1.945426   0.641906   1.923860
C     1.531907  -0.353967   2.842617
H     2.438485  -0.626719   3.386079
H     0.792410   0.046731   3.540914
H     1.127756  -1.227314   2.325976
```

PSE-OOCH$_3^•$ - Site 2

Solvent = Water
E = -3109.191181
G = -3109.011030
ZPC = 0.224231
TCE = 0.239235
TCH = 0.240179
TCG = 0.180151
N_{imag} = 0

```
Se   -0.779043  -1.680699   0.500865
N    -0.164586   1.320494  -0.240645
C     0.851598   0.444881  -0.521865
C     0.749068  -0.933126  -0.383465
C    -4.277413   0.487242  -0.193661
C    -3.814802   1.780888  -0.421546
C    -2.451334   2.040557  -0.442498
C    -1.530898   1.013989  -0.212438
C    -1.996751  -0.281381   0.035306
C    -3.364524  -0.540119   0.024262
C     2.152246   1.079840  -0.924407
C     1.766428  -1.774479  -0.818638
H    -5.339609   0.275949  -0.184614
H    -4.514220   2.590137  -0.593501
H    -2.081174   3.041950  -0.635794
C     3.124856   0.114306  -1.510796
C     2.938558  -1.222851  -1.419810
H     4.014115   0.533701  -1.966626
H     3.683860  -1.902413  -1.818231
H     0.046716   2.299621  -0.400609
H     1.965782   1.925257  -1.597159
H     1.661026  -2.847489  -0.719708
H    -3.713866  -1.550690   0.204462
O     2.738139   1.794510   0.210769
O     3.135692   0.844129   1.196512
C     2.290736   1.010596   2.329071
H     2.701711   0.335480   3.080900
H     2.336624   2.039468   2.691343
H     1.261327   0.728187   2.099293
```

Solvent = Pentyl Ethanoate
E = -3109.193841
G = -3109.013957
ZPC = 0.224008
TCE = 0.238999
TCH = 0.239943
TCG = 0.179883
N_{imag} = 0

```
Se   -0.853556  -1.671913   0.610897
N    -0.099524   1.245953  -0.216983
C     0.871703   0.312991  -0.501236
C     0.709226  -1.045306  -0.305716
C    -4.245246   0.594564  -0.288503
C    -3.722281   1.860137  -0.537628
C    -2.348900   2.063803  -0.521923
C    -1.479900   1.009704  -0.227980
C    -2.008139  -0.256581   0.044720
C    -3.383225  -0.461490  -0.009237
C     2.169358   0.884484  -1.008487
C     1.694611  -1.953610  -0.690752
H    -5.315120   0.426876  -0.310691
H    -4.381980   2.691121  -0.757586
H    -1.934076   3.043092  -0.737994
C     3.152084  -0.157638  -1.424829
C     2.911664  -1.479036  -1.261962
H     4.084922   0.199907  -1.845563
H     3.656962  -2.204393  -1.569290
H     0.170543   2.210464  -0.368237
H     1.961650   1.577756  -1.836948
H     1.537689  -3.015459  -0.548358
H    -3.779726  -1.451765   0.186353
O     2.729885   1.812101  -0.045863
O     3.167514   1.073680   1.091864
C     2.351940   1.453641   2.187605
H     2.790501   0.946384   3.048935
H     2.390866   2.535085   2.341986
H     1.318777   1.124087   2.053322
```

PSE-OOCH$_3^•$ - Site 3

Solvent = Water
E = -3109.188187
G = -3109.007889
ZPC = 0.224312
TCE = 0.239194
TCH = 0.240138
TCG = 0.180298
N_{imag} = 0

```
Se   -1.015685  -1.638014   0.469386
N    -0.393630   1.404659   0.119823
C     0.633330   0.581074  -0.374410
C     0.501129  -0.843194  -0.304037
C    -4.503697   0.557777  -0.153594
C    -4.036363   1.865438  -0.271065
C    -2.675751   2.133024  -0.210293
C    -1.754084   1.099683  -0.007380
C    -2.230697  -0.208952   0.117947
C    -3.594569  -0.477576   0.031728
C     1.768068   1.134599  -0.881051
C     1.550693  -1.672739  -0.780473
H    -5.563806   0.343651  -0.209520
H    -4.732807   2.682077  -0.420250
H    -2.307518   3.148617  -0.311407
C     2.964098   0.322979  -1.235010
C     2.710456  -1.148056  -1.246822
```

H	3.391165	0.650066	-2.190470
H	3.504838	-1.790631	-1.608608
H	-0.198190	2.390620	-0.013259
H	1.850264	2.213930	-0.962331
H	1.403123	-2.747434	-0.770757
H	-3.941153	-1.501264	0.120503
O	4.094528	0.629308	-0.354911
O	3.757403	0.231985	0.971791
C	3.681688	1.409806	1.763217
H	2.871920	2.057717	1.421374
H	3.475888	1.054548	2.773991
H	4.633737	1.944452	1.745277

Solvent = Pentyl Ethanoate
E = -3109.189992
G = -3109.010444
ZPC = 0.223913
TCE = 0.238901
TCH = 0.239845
TCG = 0.179548
N_{imag} = 0

Se	-1.036901	-1.633507	0.463389
N	-0.412824	1.403622	0.095928
C	0.623156	0.579344	-0.373491
C	0.482490	-0.844814	-0.311534
C	-4.525770	0.569253	-0.130619
C	-4.056644	1.875510	-0.247663
C	-2.694999	2.138517	-0.201602
C	-1.772710	1.103168	-0.011608
C	-2.252005	-0.204874	0.115535
C	-3.616907	-0.468400	0.041200
C	1.771349	1.128170	-0.853159
C	1.536263	-1.676907	-0.770240
H	-5.587001	0.357897	-0.176664
H	-4.752628	2.694549	-0.386572
H	-2.326487	3.153907	-0.305721
C	2.965670	0.313845	-1.217572
C	2.705899	-1.157418	-1.215790
H	3.356637	0.626265	-2.195277
H	3.506910	-1.804684	-1.554199
H	-0.210041	2.390453	-0.003315
H	1.860115	2.207618	-0.932608
H	1.388061	-2.751730	-0.757562
H	-3.966141	-1.491612	0.128463
O	4.116089	0.631453	-0.388162
O	3.831266	0.222944	0.946384
C	3.813548	1.391730	1.742425
H	2.999586	2.059485	1.447976
H	3.649177	1.037891	2.762077
H	4.771340	1.915918	1.685608

PSE-OOCH$_3$• - Site 4
Solvent = Water
E = -3109.191253
G = -3109.011054
ZPC = 0.224349
TCE = 0.239289
TCH = 0.240233
TCG = 0.180199
N_{imag} = 0

Se	-0.813743	-1.604930	0.130302
N	-0.721521	1.566626	0.157900
C	0.436003	0.975310	-0.295697
C	0.583747	-0.435176	-0.447786
C	-4.633263	0.048870	0.097558
C	-4.398205	1.420565	0.179196
C	-3.099640	1.905941	0.189139
C	-2.008935	1.028686	0.134700
C	-2.247219	-0.348469	0.066338
C	-3.555876	-0.826887	0.035251
C	1.525831	1.814968	-0.669455
C	1.732693	-0.977321	-0.924859
H	-5.645057	-0.336896	0.080831
H	-5.227417	2.116285	0.225706
H	-2.909436	2.973309	0.239269
C	2.698196	1.309172	-1.120234
C	2.956544	-0.158339	-1.164439
H	3.500960	1.970241	-1.425524
H	3.456064	-0.451218	-2.094694
H	-0.691997	2.579402	0.196672
H	1.377250	2.889633	-0.614857
H	1.816984	-2.050039	-1.061783
H	-3.725825	-1.896179	-0.027332
O	4.010777	-0.531576	-0.210945
O	3.553531	-0.246153	1.109408
C	4.412329	0.750486	1.645934
H	4.056081	0.908627	2.664912
H	5.444943	0.395561	1.665638
H	4.337663	1.677500	1.074023

Solvent = Pentyl Ethanoate
E = -3109.192826
G = -3109.012880
ZPC = 0.224269
TCE = 0.239226
TCH = 0.240170
TCG = 0.179946
N_{imag} = 0

Se	-0.804243	-1.590743	0.129155
N	-0.767397	1.568991	0.223166
C	0.407089	1.011543	-0.235546
C	0.571831	-0.391236	-0.437603
C	-4.652986	-0.003759	0.053641
C	-4.442251	1.368017	0.176975
C	-3.152117	1.873429	0.221924
C	-2.046121	1.016079	0.164443
C	-2.260721	-0.363047	0.056791
C	-3.560202	-0.859908	-0.012965
C	1.494304	1.873287	-0.557173
C	1.722648	-0.903481	-0.936304
H	-5.658061	-0.404786	0.007574
H	-5.283740	2.048851	0.227248
H	-2.982462	2.942595	0.302575
C	2.672120	1.397349	-1.026810
C	2.936077	-0.064826	-1.177237
H	3.473376	2.078955	-1.289229
H	3.372095	-0.284554	-2.160443
H	-0.750615	2.577228	0.314359
H	1.346160	2.943913	-0.443774
H	1.821941	-1.971492	-1.098808
H	-3.712589	-1.929497	-0.109582
O	4.037144	-0.498862	-0.330197
O	3.646964	-0.338495	1.031011

C	4.537409	0.598258	1.604778
H	4.246594	0.660230	2.655352
H	5.570991	0.249435	1.530653
H	4.437010	1.580073	1.134637

PSE-OOCH$_3^•$ - Site 5
Solvent = Water
E = -3109.187839
G = -3109.007667
ZPC = 0.223963
TCE = 0.238859
TCH = 0.239803
TCG = 0.180171
N_{imag} = 0

Se	-0.334072	-1.432840	0.017697
N	-0.929097	1.484980	0.865444
C	0.289224	1.339970	0.169142
C	0.701790	0.124096	-0.301782
C	-4.403844	-0.541194	-0.373253
C	-4.476206	0.771403	0.086196
C	-3.323705	1.445398	0.470838
C	-2.081837	0.805670	0.431397
C	-2.017132	-0.517326	-0.014849
C	-3.167937	-1.178157	-0.434312
C	1.106316	2.483765	-0.006511
C	2.000293	-0.063808	-1.020427
H	-5.298845	-1.066651	-0.682970
H	-5.431612	1.280183	0.136196
H	-3.373187	2.473715	0.813313
C	2.360888	2.374096	-0.653047
C	2.806571	1.188512	-1.142156
H	2.966844	3.266199	-0.762992
H	3.762955	1.105629	-1.645250
H	-1.121862	2.451880	1.102540
H	0.764025	3.439435	0.372639
H	1.821820	-0.499387	-2.014244
H	-3.095077	-2.198944	-0.792366
O	2.765168	-1.135573	-0.415201
O	3.070594	-0.765599	0.929421
C	4.486244	-0.687120	1.030842
H	4.676839	-0.449990	2.078349
H	4.941185	-1.647209	0.779127
H	4.878950	0.104374	0.389640

Solvent = Pentyl Ethanoate
E = -3109.189946
G = -3109.010115
ZPC = 0.223754
TCE = 0.238690
TCH = 0.239634
TCG = 0.179831
N_{imag} = 0

Se	-0.314594	-1.411326	-0.001080
N	-0.948072	1.505079	0.829045
C	0.281348	1.368252	0.156742
C	0.707955	0.152498	-0.307790
C	-4.400286	-0.585296	-0.353425
C	-4.491601	0.723777	0.109975
C	-3.346710	1.419400	0.477798
C	-2.092527	0.805099	0.419748
C	-2.008994	-0.517825	-0.026462
C	-3.152876	-1.197455	-0.432002
C	1.098108	2.511206	-0.002556
C	2.016722	-0.030977	-1.011613
H	-5.289187	-1.127490	-0.652080
H	-5.456102	1.213651	0.175575
H	-3.413801	2.446612	0.821613
C	2.371553	2.403081	-0.614720
C	2.830855	1.220645	-1.095024
H	2.980709	3.295799	-0.703209
H	3.800418	1.141729	-1.573545
H	-1.143758	2.457995	1.109178
H	0.748417	3.467910	0.367784
H	1.838745	-0.429802	-2.022319
H	-3.066075	-2.216345	-0.793233
O	2.756632	-1.126975	-0.436749
O	3.039157	-0.803726	0.924336
C	4.448057	-0.779686	1.055343
H	4.628549	-0.582727	2.113720
H	4.880379	-1.745703	0.782489
H	4.888465	0.017202	0.450665

PSE-OOCH$_3^•$ - Site 5a
Solvent = Water
E = -3109.196191
G = -3109.015951
ZPC = 0.223526
TCE = 0.238517
TCH = 0.239462
TCG = 0.180241
N_{imag} = 0

Se	-0.160842	-1.797275	0.111129
N	-0.183126	1.050615	-1.231651
C	1.056046	0.566946	-0.904094
C	1.177647	-0.318775	0.287685
C	-3.973225	-0.094939	0.145234
C	-3.779556	1.136109	-0.482611
C	-2.516081	1.511140	-0.909225
C	-1.420372	0.654035	-0.732766
C	-1.618026	-0.585852	-0.106599
C	-2.890441	-0.943803	0.337932
C	2.172399	0.924692	-1.650803
C	2.537647	-0.868543	0.496567
H	-4.957465	-0.388657	0.488745
H	-4.614657	1.809750	-0.633194
H	-2.354663	2.468920	-1.393524
C	3.429297	0.416573	-1.355086
C	3.588835	-0.501407	-0.277821
H	4.284450	0.704741	-1.952262
H	4.571028	-0.912168	-0.072835
H	-0.201740	1.815042	-1.897699
H	2.032098	1.599303	-2.489447
H	2.652436	-1.557226	1.326211
H	-3.026930	-1.897993	0.834239
O	0.676673	0.321622	1.487645
O	1.472203	1.491455	1.730705
C	0.576718	2.596182	1.750333
H	0.111014	2.740597	0.773625
H	-0.183468	2.457113	2.521723
H	1.202063	3.454875	1.998300

Solvent = Pentyl Ethanoate
E = -3109.198703

G = -3109.018582
ZPC = 0.223382
TCE = 0.238416
TCH = 0.239360
TCG = 0.180121
N_{imag} = 0

Se	-0.124228	-1.778080	0.181232
N	-0.185161	1.054028	-1.219707
C	1.055961	0.552915	-0.917471
C	1.194526	-0.263312	0.320792
C	-3.973321	-0.166289	0.100920
C	-3.798905	1.055339	-0.548657
C	-2.536107	1.452932	-0.955966
C	-1.422645	0.628197	-0.742736
C	-1.600279	-0.602948	-0.093419
C	-2.872705	-0.980980	0.333707
C	2.148644	0.824104	-1.728534
C	2.560232	-0.788857	0.546919
H	-4.956276	-0.478125	0.432411
H	-4.647536	1.704291	-0.730150
H	-2.390340	2.407383	-1.452201
C	3.406946	0.314022	-1.436086
C	3.590671	-0.506029	-0.288010
H	4.244513	0.533901	-2.085286
H	4.576348	-0.903052	-0.072396
H	-0.214865	1.758079	-1.946878
H	1.994534	1.430739	-2.615409
H	2.695842	-1.392359	1.437496
H	-2.995249	-1.926040	0.851155
O	0.672154	0.403034	1.480848
O	1.437179	1.603115	1.671827
C	0.496498	2.659209	1.745378
H	-0.040668	2.778090	0.801107
H	-0.210041	2.493754	2.562713
H	1.091880	3.550850	1.951069

TS[PSE-OOCH$_3^{\bullet}$] - Site 2a
Solvent = Water
E = -3109.177951
G = -3108.998845
ZPC = 0.222273
TCE = 0.237118
TCH = 0.238062
TCG = 0.179105
N_{imag} = 1, -470.43 cm^{-1}

Se	0.378038	1.762303	-0.275892
N	0.298061	-1.286531	-1.120801
C	-0.930250	-0.792729	-0.690967
C	-1.108836	0.623903	-0.482063
C	4.006615	-0.116931	0.478309
C	3.807047	-1.416113	0.016106
C	2.572821	-1.795305	-0.492386
C	1.519732	-0.879039	-0.563448
C	1.728085	0.426582	-0.111771
C	2.962701	0.797978	0.417047
C	-2.093361	-1.542478	-1.105310
C	-2.386759	1.157745	-0.411487
H	4.964494	0.182941	0.884890
H	4.611122	-2.141011	0.059141
H	2.407375	-2.808957	-0.842431
C	-3.337341	-0.987899	-1.047286
C	-3.499865	0.366233	-0.672677
H	-4.205911	-1.581338	-1.305853
H	-4.491167	0.798726	-0.620880
H	0.270223	-2.275819	-1.344129
H	-1.943977	-2.575821	-1.399553
H	-2.511589	2.210124	-0.181350
H	3.104100	1.812752	0.772511
O	-0.804668	-1.387974	1.101741
O	-1.939260	-1.108195	1.860185
C	-1.644622	-0.044612	2.756502
H	-2.566843	0.138642	3.309680
H	-0.849925	-0.341165	3.445603
H	-1.352026	0.853683	2.209496

Solvent = Pentyl Ethanoate
E = -3109.176249
G = -3108.997418
ZPC = 0.222065
TCE = 0.236945
TCH = 0.237889
TCG = 0.178830
N_{imag} = 1, -615.29 cm^{-1}

Se	0.388173	1.778182	-0.246762
N	0.290312	-1.227808	-1.173407
C	-0.941764	-0.758277	-0.698589
C	-1.115566	0.650569	-0.440157
C	3.997011	-0.148391	0.496340
C	3.792433	-1.427740	-0.012858
C	2.558889	-1.781033	-0.542447
C	1.511932	-0.857225	-0.593224
C	1.728603	0.430761	-0.095607
C	2.958371	0.774814	0.458299
C	-2.110404	-1.477345	-1.151502
C	-2.387821	1.179939	-0.315643
H	4.953119	0.129682	0.922514
H	4.591029	-2.159896	0.011917
H	2.392259	-2.782947	-0.924410
C	-3.353935	-0.927657	-1.040934
C	-3.509369	0.399978	-0.588180
H	-4.227006	-1.507967	-1.314760
H	-4.499184	0.828408	-0.489832
H	0.257553	-2.204895	-1.439540
H	-1.969077	-2.495068	-1.499293
H	-2.507644	2.221565	-0.036631
H	3.103049	1.775028	0.852418
O	-0.806456	-1.440090	1.008947
O	-1.923727	-1.192173	1.781561
C	-1.604490	-0.189603	2.733465
H	-2.524679	-0.007759	3.291670
H	-0.822650	-0.543442	3.411196
H	-1.278699	0.729119	2.240668

TS[PSE-OOCH$_3^{\bullet}$] - Site 2
Solvent = Water
E = -3109.177772
G = -3108.998932
ZPC = 0.222805
TCE = 0.237754
TCH = 0.238698
TCG = 0.178840
N_{imag} = 1, -518.73 cm^{-1}

Se	-0.698360	-1.677953	0.330410
N	-0.245067	1.404729	-0.178434
C	0.801907	0.613508	-0.573518
C	0.761749	-0.790359	-0.527926
C	-4.311717	0.369397	-0.063479
C	-3.916842	1.696735	-0.208046
C	-2.568044	2.022240	-0.254388
C	-1.594518	1.026709	-0.133254
C	-1.992193	-0.303610	0.028783
C	-3.346039	-0.626503	0.044199
C	2.015883	1.264224	-0.978026
C	1.817968	-1.529331	-1.025798
H	-5.362161	0.107319	-0.035206
H	-4.657861	2.482127	-0.295516
H	-2.250874	3.051623	-0.384331
C	3.029886	0.476200	-1.602711
C	2.949563	-0.888442	-1.585670
H	3.885648	0.983177	-2.031037
H	3.739947	-1.493562	-2.013116
H	-0.091137	2.401579	-0.284929
H	1.939810	2.314884	-1.239980
H	1.771957	-2.611964	-0.998934
H	-3.642219	-1.663480	0.156699
O	2.788740	1.758166	0.669948
O	3.225298	0.626222	1.345346
C	2.307667	0.318167	2.388742
H	2.717238	-0.562491	2.885049
H	2.246510	1.149836	3.094254
H	1.318930	0.089540	1.986380

Solvent = Pentyl Ethanoate
E = -3109.178334
G = -3108.999620
ZPC = 0.222619
TCE = 0.237563
TCH = 0.238507
TCG = 0.178714
N_{imag} = 1, -629.46 cm^{-1}

Se	-0.708094	-1.665726	0.409333
N	-0.216523	1.375916	-0.126935
C	0.812687	0.567069	-0.564470
C	0.755354	-0.827823	-0.493729
C	-4.299355	0.392395	-0.146204
C	-3.886447	1.713783	-0.287368
C	-2.533506	2.026549	-0.293632
C	-1.573178	1.024940	-0.129445
C	-1.991576	-0.299522	0.033305
C	-3.346980	-0.611286	0.001959
C	2.022381	1.198779	-1.009411
C	1.791131	-1.597465	-1.000974
H	-5.352922	0.141105	-0.151833
H	-4.616841	2.505456	-0.405474
H	-2.205775	3.052897	-0.424415
C	3.015878	0.380069	-1.631800
C	2.919924	-0.983926	-1.585767
H	3.873890	0.865908	-2.079805
H	3.699838	-1.605258	-2.009607
H	-0.040613	2.369016	-0.216350
H	1.941010	2.234268	-1.327303
H	1.725561	-2.678531	-0.955005
H	-3.656182	-1.644993	0.111421
O	2.817273	1.768479	0.555605
O	3.211325	0.685868	1.311803
C	2.281528	0.491149	2.367845
H	2.660554	-0.359600	2.936657
H	2.241846	1.377109	3.007115
H	1.284653	0.262847	1.984690

TS[PSE-OOCH$_3^•$] - Site 3
Solvent = Water
E = -3109.174802
G = -3108.995762
ZPC = 0.222700
TCE = 0.237492
TCH = 0.238436
TCG = 0.179039
N_{imag} = 1, -576.74 cm^{-1}

Se	-0.840872	-1.631103	0.449234
N	-0.369779	1.425055	-0.039713
C	0.668867	0.621528	-0.523092
C	0.618241	-0.789774	-0.400210
C	-4.444803	0.393928	-0.084255
C	-4.043083	1.716749	-0.256964
C	-2.694248	2.045067	-0.267740
C	-1.720664	1.058346	-0.080896
C	-2.130191	-0.264990	0.104422
C	-3.482684	-0.595463	0.086186
C	1.781945	1.206414	-1.088274
C	1.676458	-1.582685	-0.883978
H	-5.495604	0.132118	-0.084518
H	-4.781576	2.497803	-0.393598
H	-2.376033	3.072015	-0.414002
C	2.938461	0.429505	-1.383679
C	2.802994	-0.998924	-1.401058
H	3.696211	0.877083	-2.016677
H	3.622650	-1.605817	-1.764852
H	-0.218420	2.412501	-0.212718
H	1.815427	2.283785	-1.209609
H	1.583676	-2.662089	-0.842441
H	-3.779167	-1.630095	0.218353
O	4.053489	0.634575	0.106972
O	3.343218	0.253081	1.232634
C	2.937019	1.415379	1.946051
H	2.334504	2.066578	1.309885
H	2.340797	1.051054	2.783530
H	3.812099	1.955465	2.314754

Solvent = Pentyl Ethanoate
E = -3109.175172
G = -3108.996321
ZPC = 0.222503
TCE = 0.237308
TCH = 0.238252
TCG = 0.178851
N_{imag} = 1, -650.43 cm^{-1}

Se	-0.809140	-1.625285	0.428145
N	-0.387307	1.432757	0.045662
C	0.660344	0.667011	-0.486815
C	0.624555	-0.744962	-0.435787
C	-4.446047	0.348063	-0.114999
C	-4.064134	1.682409	-0.224797
C	-2.720799	2.032530	-0.196983
C	-1.734166	1.055808	-0.029871

C	-2.125155	-0.280882	0.095676
C	-3.470296	-0.631682	0.034049
C	1.764181	1.291822	-1.027358
C	1.672917	-1.501435	-0.990327
H	-5.492193	0.069578	-0.148178
H	-4.813399	2.456111	-0.345061
H	-2.420655	3.070526	-0.299222
C	2.929692	0.540435	-1.364702
C	2.790670	-0.883120	-1.485056
H	3.674983	1.029925	-1.982042
H	3.608771	-1.463402	-1.893100
H	-0.243807	2.429634	-0.060206
H	1.790016	2.374443	-1.091954
H	1.585453	-2.582093	-1.013831
H	-3.751764	-1.675895	0.115826
O	4.023323	0.671312	0.102427
O	3.361717	0.142902	1.187370
C	2.918783	1.199865	2.024035
H	2.256179	1.877168	1.480183
H	2.372050	0.720990	2.838059
H	3.772681	1.754527	2.422192

TS[PSE-OOCH$_3$•] - Site 4
Solvent = Water
E = -3109.178439
G = -3108.999675
ZPC = 0.222819
TCE = 0.237745
TCH = 0.238689
TCG = 0.178764
N_{imag} = 1, -457.33 cm^{-1}

Se	-0.784777	-1.625540	0.168140
N	-0.597837	1.539647	-0.023419
C	0.518822	0.885638	-0.474308
C	0.614736	-0.530076	-0.524620
C	-4.551076	0.144472	0.177796
C	-4.273302	1.509428	0.153289
C	-2.960864	1.951940	0.078260
C	-1.902934	1.037507	0.044046
C	-2.182775	-0.331438	0.081811
C	-3.504115	-0.769190	0.134639
C	1.613533	1.657671	-0.930919
C	1.754119	-1.129438	-0.994941
H	-5.573621	-0.208550	0.228768
H	-5.078961	2.232949	0.184604
H	-2.735624	3.013045	0.046127
C	2.762234	1.060121	-1.372471
C	2.916935	-0.357407	-1.295333
H	3.584570	1.664913	-1.734928
H	3.705842	-0.825625	-1.871714
H	-0.540028	2.551709	-0.059449
H	1.508066	2.737587	-0.945750
H	1.810494	-2.210438	-1.054830
H	-3.707619	-1.834167	0.153445
O	3.965518	-0.582501	0.283164
O	3.250990	-0.033326	1.345130
C	3.877844	1.179117	1.741301
H	3.257196	1.585596	2.541196
H	4.884958	0.979214	2.115746
H	3.920995	1.881140	0.906316

Solvent = Pentyl Ethanoate
E = -3109.177449
G = -3108.998721
ZPC = 0.222730
TCE = 0.237624
TCH = 0.238568
TCG = 0.178728
N_{imag} = 1, -597.03 cm^{-1}

Se	-0.758407	-1.611707	0.189630
N	-0.608837	1.530343	0.137141
C	0.510582	0.918341	-0.389173
C	0.614079	-0.488534	-0.515272
C	-4.556982	0.101101	0.069468
C	-4.294373	1.467126	0.123921
C	-2.984696	1.925816	0.132698
C	-1.914658	1.025749	0.111690
C	-2.181415	-0.346582	0.073015
C	-3.497429	-0.798848	0.034780
C	1.579394	1.720069	-0.842825
C	1.737215	-1.057715	-1.055652
H	-5.576701	-0.263585	0.051850
H	-5.109436	2.180704	0.148667
H	-2.774443	2.990307	0.158181
C	2.720909	1.153733	-1.349166
C	2.891881	-0.264270	-1.341399
H	3.526214	1.782413	-1.710038
H	3.656757	-0.692789	-1.978814
H	-0.555412	2.540913	0.176595
H	1.467449	2.799282	-0.808700
H	1.797083	-2.133928	-1.171295
H	-3.690121	-1.865209	-0.009532
O	3.965820	-0.576137	0.150163
O	3.284113	-0.136701	1.267893
C	3.903129	1.046760	1.741682
H	3.307182	1.372678	2.596298
H	4.928397	0.840347	2.061650
H	3.905437	1.820714	0.970697

TS[PSE-OOCH$_3$•] - Site 5
Solvent = Water
E = -3109.172981
G = -3108.993713
ZPC = 0.222790
TCE = 0.237579
TCH = 0.238523
TCG = 0.179268
N_{imag} = 1, -605.35 cm^{-1}

Se	0.497794	-1.526038	0.033580
N	0.716929	1.476296	-0.721763
C	-0.431104	1.171862	0.031003
C	-0.679215	-0.107828	0.489187
C	4.465303	-0.215624	0.172786
C	4.366488	1.114190	-0.227471
C	3.124584	1.676196	-0.497093
C	1.962326	0.906376	-0.400604
C	2.068699	-0.432148	-0.012015
C	3.310000	-0.982519	0.291634
C	-1.362947	2.191228	0.303359
C	-1.914992	-0.422484	1.141535
H	5.430482	-0.654696	0.393316
H	5.257542	1.723710	-0.320188
H	3.042492	2.716913	-0.793044

C	-2.517195	1.928681	1.052010
C	-2.774358	0.661420	1.518525
H	-3.193850	2.743823	1.279019
H	-3.653766	0.446713	2.112647
H	0.789010	2.465478	-0.933695
H	-1.163256	3.193506	-0.060356
H	-1.948540	-1.329169	1.736810
H	3.370785	-2.018267	0.606275
O	-2.859343	-1.265688	-0.192951
O	-3.028437	-0.394899	-1.252880
C	-4.387872	0.029869	-1.293200
H	-4.656606	0.539549	-0.366368
H	-4.450458	0.720196	-2.134903
H	-5.043403	-0.827141	-1.460190

Solvent = Pentyl Ethanoate
E = -3109.173767
G = -3108.994838
ZPC = 0.222569
TCE = 0.237420
TCH = 0.238364
TCG = 0.178929
N_{imag} = 1, -660.31 cm^{-1}

Se	0.470035	-1.503196	0.056764
N	0.741601	1.491878	-0.700497
C	-0.424645	1.204514	0.026541
C	-0.687830	-0.067779	0.498094
C	4.463080	-0.266772	0.161810
C	4.386783	1.059706	-0.251459
C	3.153316	1.643825	-0.511726
C	1.975989	0.899780	-0.394642
C	2.059882	-0.438369	0.004337
C	3.293486	-1.008251	0.300534
C	-1.355607	2.230719	0.266720
C	-1.931793	-0.370679	1.141387
H	5.421710	-0.723066	0.376493
H	5.289141	1.649787	-0.361306
H	3.091373	2.683569	-0.817066
C	-2.523871	1.981941	0.997997
C	-2.791933	0.724598	1.484486
H	-3.201850	2.802779	1.201773
H	-3.678539	0.525672	2.073880
H	0.821208	2.468635	-0.954747
H	-1.146923	3.227484	-0.107385
H	-1.958480	-1.251441	1.775849
H	3.337391	-2.041919	0.625558
O	-2.838528	-1.278124	-0.143491
O	-2.996779	-0.477073	-1.250183
C	-4.351801	-0.059226	-1.324932
H	-4.629257	0.515170	-0.438427
H	-4.414994	0.570504	-2.213687
H	-5.011068	-0.924122	-1.432920

TS[PSE-OOCH$_3$•] - Site 5a
Solvent = Water
E = -3109.179883
G = -3109.000812
ZPC = 0.222364
TCE = 0.237330
TCH = 0.238274
TCG = 0.179071
N_{imag} = 1, -428.29 cm^{-1}

Se	-0.376445	-1.825001	0.041847
N	-0.212587	1.030545	-1.292290
C	1.003890	0.500419	-0.964006
C	1.115179	-0.626916	-0.082007
C	-3.992169	0.227231	0.336139
C	-3.733168	1.433302	-0.312949
C	-2.472495	1.690691	-0.829153
C	-1.451325	0.739347	-0.717669
C	-1.714835	-0.472173	-0.069427
C	-2.979328	-0.716451	0.461303
C	2.171548	1.113011	-1.447943
C	2.405576	-1.221941	0.086143
H	-4.973107	0.023524	0.747456
H	-4.512406	2.179337	-0.412044
H	-2.258706	2.629256	-1.330184
C	3.409858	0.579361	-1.164431
C	3.525424	-0.608144	-0.401318
H	4.300791	1.062143	-1.546873
H	4.502436	-1.041629	-0.225627
H	-0.176345	1.903671	-1.807991
H	2.074545	2.003500	-2.059967
H	2.472897	-2.142409	0.654775
H	-3.167296	-1.655066	0.970583
O	0.830288	0.207759	1.627031
O	1.821254	1.158168	1.875317
C	1.271190	2.457840	1.705624
H	0.923773	2.605903	0.680847
H	0.448850	2.614603	2.407737
H	2.083410	3.152506	1.923922

Solvent = Pentyl Ethanoate
E = -3109.178041
G = -3108.999773
ZPC = 0.221812
TCE = 0.236893
TCH = 0.237837
TCG = 0.178268
N_{imag} = 1, -584.30 cm^{-1}

Se	-0.354720	-1.824864	0.019485
N	-0.212661	1.016408	-1.310967
C	1.015707	0.505004	-0.966559
C	1.129896	-0.604487	-0.064733
C	-3.981459	0.211061	0.346834
C	-3.733102	1.412132	-0.312125
C	-2.475608	1.672509	-0.837336
C	-1.449582	0.726956	-0.731728
C	-1.704439	-0.482062	-0.074621
C	-2.961187	-0.725904	0.470623
C	2.177401	1.120363	-1.446634
C	2.423596	-1.181867	0.123863
H	-4.957794	0.005884	0.768627
H	-4.516031	2.154918	-0.409527
H	-2.273210	2.613597	-1.339085
C	3.423721	0.605179	-1.142151
C	3.544640	-0.563351	-0.360217
H	4.311673	1.093682	-1.525073
H	4.524202	-0.981100	-0.161261
H	-0.186709	1.865543	-1.861818
H	2.081744	1.998483	-2.077198
H	2.495193	-2.088048	0.714590
H	-3.139354	-1.659748	0.992453

O	0.798651	0.196934	1.616380
O	1.751106	1.152509	1.905517
C	1.191597	2.439647	1.703663
H	0.926003	2.595995	0.655156
H	0.309609	2.575744	2.334896
H	1.968635	3.148938	1.994088

PTE-OH$^{\bullet}$ - Site 2a

Solvent = Water
E = -861.084064
G = -860.933324
ZPC = 0.191097
TCE = 0.203605
TCH = 0.204549
TCG = 0.150739
N_{imag} = 0

Te	-0.107264	-1.644608	-0.009664
N	0.061493	1.642378	-0.557241
C	1.163545	1.145920	0.247787
C	1.479167	-0.298958	-0.068916
C	-3.928745	0.426460	0.062200
C	-3.616413	1.755496	-0.219590
C	-2.295152	2.137389	-0.395448
C	-1.252709	1.203227	-0.312923
C	-1.571255	-0.135169	-0.044321
C	-2.903862	-0.506514	0.151720
C	2.351535	2.044101	0.030363
C	2.760955	-0.724809	-0.315875
H	-4.956734	0.119439	0.211366
H	-4.401342	2.499268	-0.291580
H	-2.047304	3.174075	-0.600848
C	3.595736	1.570036	-0.212830
C	3.834692	0.176518	-0.371329
H	4.424965	2.262997	-0.300428
H	4.836004	-0.185093	-0.565542
H	0.099348	2.654705	-0.620656
H	2.157995	3.106369	0.147042
H	2.948041	-1.779152	-0.493548
H	-3.140398	-1.543597	0.367643
O	0.756257	1.255796	1.633003
H	1.471682	0.905411	2.180740

Solvent = Pentyl Ethanoate
E = -861.080851
G = -860.930816
ZPC = 0.190758
TCE = 0.203451
TCH = 0.204395
TCG = 0.150035
N_{imag} = 0

Te	-0.113290	-1.635050	0.006631
N	0.065988	1.661388	-0.481223
C	1.175920	1.133984	0.286715
C	1.478021	-0.301987	-0.088950
C	-3.933694	0.426725	0.007724
C	-3.616855	1.757044	-0.260598
C	-2.293377	2.143477	-0.397719
C	-1.247967	1.214555	-0.287688
C	-1.573230	-0.126484	-0.040250
C	-2.908548	-0.502811	0.117421
C	2.368156	2.030395	0.073985
C	2.747313	-0.723318	-0.400702
H	-4.964474	0.116442	0.128674
H	-4.401596	2.499228	-0.351257
H	-2.045992	3.182530	-0.591107
C	3.599487	1.560009	-0.232968
C	3.823096	0.174236	-0.462511
H	4.431062	2.251256	-0.314375
H	4.813773	-0.183405	-0.711723
H	0.115744	2.670298	-0.562154
H	2.192892	3.087156	0.253166
H	2.923401	-1.770777	-0.625101
H	-3.148828	-1.542247	0.319362
O	0.790354	1.199946	1.674731
H	1.480399	0.767916	2.193685

PTE-OH$^{\bullet}$ - Site 2

Solvent = Water
E = -861.086583
G = -860.936223
ZPC = 0.191662
TCE = 0.204371
TCH = 0.205316
TCG = 0.150359
N_{imag} = 0

Te	-0.466577	-1.669326	0.273775
N	0.167805	1.549826	0.400997
C	1.263692	0.809223	0.007802
C	1.260777	-0.555562	-0.204078
C	-3.887833	0.935143	-0.526805
C	-3.378253	2.218073	-0.340585
C	-2.034040	2.398374	-0.047612
C	-1.179660	1.297595	0.089940
C	-1.694671	0.006956	-0.075290
C	-3.039797	-0.160579	-0.402882
C	2.519547	1.638214	-0.158163
C	2.411043	-1.218821	-0.647827
H	-4.934000	0.786708	-0.765274
H	-4.023983	3.083157	-0.434086
H	-1.627855	3.396799	0.077876
C	3.695881	0.837533	-0.611431
C	3.624273	-0.497088	-0.835696
H	4.621840	1.384656	-0.749444
H	4.505841	-1.038198	-1.161881
H	0.372029	2.533190	0.542933
H	2.307725	2.443299	-0.873541
H	2.387955	-2.287079	-0.824321
H	-3.430571	-1.162408	-0.545106
O	2.809650	2.356977	1.059827
H	3.065437	1.705879	1.726722

Solvent = Pentyl Ethanoate
E = -861.086697
G = -860.936238
ZPC = 0.191589
TCE = 0.204285
TCH = 0.205229
TCG = 0.150459
N_{imag} = 0

Te	-0.468055	-1.674857	0.262983
N	0.174181	1.534812	0.393071
C	1.268382	0.791080	0.006457

C	1.269036	-0.572885	-0.199372
C	-3.886264	0.938349	-0.515064
C	-3.375839	2.217780	-0.313065
C	-2.030230	2.393744	-0.025079
C	-1.175387	1.291490	0.095110
C	-1.693116	0.003212	-0.082577
C	-3.038401	-0.159151	-0.408630
C	2.513451	1.630869	-0.203051
C	2.431822	-1.240166	-0.605457
H	-4.933323	0.793765	-0.752444
H	-4.021933	3.084214	-0.392270
H	-1.623631	3.391238	0.107866
C	3.720921	0.813334	-0.525443
C	3.655884	-0.524709	-0.731994
H	4.655377	1.356012	-0.616970
H	4.554576	-1.074223	-0.991070
H	0.392065	2.503886	0.595087
H	2.308174	2.337965	-1.020254
H	2.413397	-2.308740	-0.781296
H	-3.431302	-1.158297	-0.564776
O	2.728422	2.497473	0.922269
H	3.017276	1.945582	1.660368

PTE-OH• - Site 3
Solvent = Water
E = -861.082127
G = -860.931627
ZPC = 0.191564
TCE = 0.204249
TCH = 0.205193
TCG = 0.150500
N_{imag} = 0

Te	0.540606	-1.623653	-0.222372
N	0.001818	1.535606	-0.728777
C	-1.141680	0.930096	-0.154329
C	-1.142256	-0.466135	0.158343
C	3.969679	0.970428	0.569490
C	3.488904	2.243324	0.269707
C	2.169797	2.418600	-0.125152
C	1.308947	1.321269	-0.253172
C	1.799128	0.042080	0.035115
C	3.116967	-0.122743	0.460352
C	-2.265249	1.675846	0.039020
C	-2.328060	-1.063370	0.668001
H	4.995189	0.829072	0.888346
H	4.138886	3.106444	0.353512
H	1.789576	3.410996	-0.345196
C	-3.566506	1.098025	0.490965
C	-3.470055	-0.352129	0.842636
H	-3.940574	1.661695	1.353405
H	-4.365287	-0.828194	1.227123
H	-0.165354	2.517560	-0.919620
H	-2.249121	2.738286	-0.185252
H	-2.309002	-2.119346	0.917546
H	3.480595	-1.117204	0.695816
O	-4.601020	1.308942	-0.503161
H	-4.328665	0.838140	-1.301494

Solvent = Pentyl Ethanoate
E = -861.080817
G = -860.930387
ZPC = 0.191536
TCE = 0.204235
TCH = 0.205179
TCG = 0.150431
N_{imag} = 0

Te	0.536130	-1.616804	-0.222914
N	0.005838	1.541030	-0.692851
C	-1.143411	0.934150	-0.138736
C	-1.145347	-0.463952	0.169944
C	3.985121	0.953544	0.552825
C	3.508559	2.228349	0.257775
C	2.186270	2.411419	-0.120884
C	1.316668	1.319925	-0.240288
C	1.804951	0.037968	0.040109
C	3.125042	-0.134299	0.452506
C	-2.271881	1.675421	0.043508
C	-2.334580	-1.065116	0.664782
H	5.013047	0.806602	0.861427
H	4.164485	3.087805	0.334530
H	1.810469	3.407501	-0.332526
C	-3.578751	1.094968	0.479835
C	-3.480321	-0.357114	0.827993
H	-3.955852	1.652528	1.345853
H	-4.379826	-0.836888	1.197829
H	-0.158745	2.514777	-0.918367
H	-2.259557	2.738673	-0.178076
H	-2.317091	-2.121894	0.912307
H	3.486277	-1.130260	0.686298
O	-4.604354	1.312552	-0.511392
H	-4.321925	0.855768	-1.312888

PTE-OH• - Site 4
Solvent = Water
E = -861.085733
G = -860.935793
ZPC = 0.191547
TCE = 0.204387
TCH = 0.205332
TCG = 0.149940
N_{imag} = 0

Te	0.292141	-1.562936	-0.138309
N	0.283115	1.733188	-0.496213
C	-0.955425	1.241859	-0.127973
C	-1.222994	-0.128306	0.160736
C	4.161390	0.379696	0.390638
C	3.906746	1.724203	0.124051
C	2.616429	2.146701	-0.153570
C	1.553391	1.231882	-0.189054
C	1.811512	-0.122236	0.059241
C	3.111197	-0.530449	0.362323
C	-2.036194	2.172998	-0.050518
C	-2.462350	-0.536033	0.542238
H	5.165387	0.042832	0.618072
H	4.712448	2.448531	0.142616
H	2.411843	3.195211	-0.346539
C	-3.287750	1.797519	0.301635
C	-3.652087	0.374762	0.581883
H	-4.081248	2.533614	0.367278
H	-4.163609	0.292021	1.546678
H	0.282916	2.730856	-0.675002
H	-1.821864	3.216408	-0.265175
H	-2.648227	-1.579268	0.777678

```
H     3.303126  -1.578602   0.566295
O    -4.664873  -0.081187  -0.354716
H    -4.278118  -0.027308  -1.238400
```

Solvent = Pentyl Ethanoate
E = -861.084490
G = -860.934222
ZPC = 0.191704
TCE = 0.204515
TCH = 0.205459
TCG = 0.150269
N_{imag} = 0

```
Te    0.290935  -1.557285  -0.140935
N     0.289010   1.735347  -0.473882
C    -0.954608   1.243944  -0.124181
C    -1.224542  -0.126228   0.164644
C     4.167875   0.369251   0.385017
C     3.916525   1.714580   0.123229
C     2.626264   2.141597  -0.145602
C     1.559420   1.231130  -0.178821
C     1.815295  -0.124675   0.063428
C     3.114621  -0.536856   0.359504
C    -2.039378   2.171779  -0.067531
C    -2.465219  -0.533453   0.539745
H     5.171860   0.028493   0.607049
H     4.724813   2.436249   0.139637
H     2.425921   3.192188  -0.333064
C    -3.293297   1.796657   0.275872
C    -3.657635   0.376802   0.578059
H    -4.091600   2.529300   0.318853
H    -4.152946   0.310782   1.553573
H     0.289500   2.727405  -0.675538
H    -1.828890   3.213779  -0.294518
H    -2.655278  -1.576585   0.772920
H     3.305551  -1.585638   0.562230
O    -4.678857  -0.091391  -0.328666
H    -4.301697  -0.060621  -1.216184
```

PTE-OH• - Site 5
Solvent = Water
E = -861.083761
G = -860.933192
ZPC = 0.191448
TCE = 0.204130
TCH = 0.205074
TCG = 0.150569
N_{imag} = 0

```
Te    0.018267  -1.494453  -0.211157
N     0.401556   1.652831  -0.855762
C    -0.869217   1.391563  -0.290669
C    -1.262558   0.133678   0.075443
C     4.000603   0.101345   0.692394
C     3.908365   1.434851   0.300957
C     2.708736   1.941442  -0.181327
C     1.585762   1.115292  -0.309633
C     1.685254  -0.229101   0.066148
C     2.882809  -0.720302   0.584645
C    -1.770946   2.482029  -0.169023
C    -2.610373  -0.152280   0.671712
H     4.931190  -0.296609   1.078496
H     4.768765   2.088861   0.381144
H     2.629927   2.984931  -0.469215
C    -3.094988   2.266601   0.281643
C    -3.520459   1.036245   0.669759
H    -3.776182   3.109533   0.312162
H    -4.536038   0.870423   1.011551
H     0.511984   2.635329  -1.079900
H    -1.446052   3.473549  -0.462704
H    -2.479784  -0.502546   1.705296
H     2.945651  -1.759193   0.889928
O    -3.223508  -1.280039   0.019116
H    -3.380123  -1.030405  -0.901463
```

Solvent = Pentyl Ethanoate
E = -861.083242
G = -860.932502
ZPC = 0.191472
TCE = 0.204102
TCH = 0.205046
TCG = 0.150740
N_{imag} = 0

```
Te    0.014948  -1.482819  -0.203990
N     0.403816   1.682252  -0.795184
C    -0.872119   1.406199  -0.260023
C    -1.263937   0.141101   0.086894
C     4.018156   0.077678   0.652508
C     3.930829   1.415435   0.278108
C     2.726329   1.938456  -0.172530
C     1.592673   1.125264  -0.289008
C     1.688726  -0.225770   0.065102
C     2.890723  -0.731755   0.555845
C    -1.788658   2.483505  -0.147539
C    -2.619860  -0.164536   0.654613
H     4.952004  -0.333177   1.016872
H     4.798844   2.060708   0.348652
H     2.653407   2.987269  -0.443300
C    -3.123450   2.248207   0.262104
C    -3.546789   1.010812   0.627987
H    -3.815474   3.082994   0.278096
H    -4.570228   0.830248   0.937656
H     0.512772   2.658148  -1.041071
H    -1.469162   3.483204  -0.419427
H    -2.504293  -0.507320   1.693235
H     2.950482  -1.774404   0.849872
O    -3.190881  -1.307096  -0.002288
H    -3.381067  -1.047662  -0.912852
```

PTE-OH• - Site 5a
Solvent = Water
E = -861.130863
G = -860.981010
ZPC = 0.192131
TCE = 0.205128
TCH = 0.206072
TCG = 0.149852
N_{imag} = 0

```
Te   -0.019646   1.760232   0.002612
N     0.074086  -1.381052  -1.246826
C    -1.184731  -1.139867  -0.614345
C    -1.406802  -1.421701   0.743854
C     3.761601  -0.335063   0.505743
C     3.602603  -1.552357  -0.151228
```

C	2.374531	-1.887654	-0.703348
C	1.286428	-1.005212	-0.640015
C	1.453570	0.237849	-0.004180
C	2.685630	0.542436	0.582720
C	-2.241223	-0.629434	-1.372942
C	-2.639938	-1.129638	1.325730
H	4.709616	-0.069846	0.957753
H	4.427647	-2.251692	-0.219339
H	2.236908	-2.846851	-1.192933
C	-3.479034	-0.369107	-0.803597
C	-3.669055	-0.605847	0.557732
H	-4.282261	0.030301	-1.410166
H	-4.624001	-0.389668	1.021955
H	0.125918	-2.289868	-1.694826
H	-2.057509	-0.420435	-2.420698
H	-2.774554	-1.338536	2.380719
H	2.809298	1.492465	1.092256
O	-0.456818	-1.971465	1.558135
H	0.324980	-2.235246	1.055421

Solvent = Pentyl Ethanoate
E = -861.132270
G = -860.982375
ZPC = 0.192245
TCE = 0.205304
TCH = 0.206248
TCG = 0.149894
N_{imag} = 0

Te	0.004283	1.764898	0.005498
N	0.055545	-1.360746	-1.239990
C	-1.201675	-1.118581	-0.611901
C	-1.420340	-1.413183	0.745287
C	3.758733	-0.380877	0.507955
C	3.585987	-1.588481	-0.161688
C	2.353287	-1.904396	-0.715237
C	1.277444	-1.006606	-0.647850
C	1.459300	0.227943	0.002551
C	2.693487	0.508324	0.595513
C	-2.254216	-0.597122	-1.368628
C	-2.660660	-1.137368	1.322211
H	4.709674	-0.132677	0.963661
H	4.402667	-2.297073	-0.236393
H	2.204895	-2.858812	-1.211202
C	-3.496115	-0.354145	-0.801763
C	-3.690055	-0.614592	0.554917
H	-4.299117	0.051314	-1.404841
H	-4.649029	-0.411884	1.017450
H	0.092963	-2.218049	-1.778176
H	-2.060555	-0.369074	-2.410921
H	-2.797124	-1.356464	2.374801
H	2.827381	1.449250	1.119334
O	-0.479586	-1.971993	1.544023
H	0.362332	-2.055578	1.079936

TS[PTE-OH•] - Site 2a
Solvent = Water
E = -861.058894
G = -860.911403
ZPC = 0.188566
TCE = 0.201534
TCH = 0.202478
TCG = 0.147490
N_{imag} = 1, -377.35 cm^{-1}

Te	-0.112689	-1.632078	-0.070251
N	-0.005981	1.667389	-0.580696
C	1.194379	1.140739	-0.171352
C	1.441558	-0.277021	-0.112037
C	-3.933554	0.397997	0.191079
C	-3.642941	1.733789	-0.091917
C	-2.338440	2.126533	-0.333088
C	-1.290513	1.193268	-0.302886
C	-1.583821	-0.148330	-0.033058
C	-2.904855	-0.531035	0.219840
C	2.333129	2.007303	-0.337107
C	2.755057	-0.743460	-0.031611
H	-4.951891	0.085476	0.386602
H	-4.435654	2.471887	-0.117587
H	-2.106586	3.165640	-0.544078
C	3.603221	1.515649	-0.268906
C	3.826334	0.129825	-0.095575
H	4.446767	2.189144	-0.359365
H	4.838203	-0.251759	-0.038150
H	0.025104	2.669723	-0.730653
H	2.145895	3.066409	-0.479223
H	2.932749	-1.809407	0.061883
H	-3.123996	-1.571621	0.435355
O	0.947029	1.359133	1.886841
H	1.797533	1.036237	2.208139

Solvent = Pentyl Ethanoate
E = -861.050026
G = -860.902955
ZPC = 0.188270
TCE = 0.201312
TCH = 0.202256
TCG = 0.147071
N_{imag} = 1, -376.79 cm^{-1}

Te	-0.109928	-1.658002	-0.122654
N	-0.007364	1.554952	-0.821029
C	1.196731	1.101681	-0.287992
C	1.454167	-0.281110	-0.049114
C	-3.884913	0.453980	0.388664
C	-3.596545	1.758086	-0.003616
C	-2.306533	2.104541	-0.378290
C	-1.282042	1.151800	-0.389255
C	-1.578689	-0.161947	-0.014565
C	-2.870517	-0.496645	0.387318
C	2.319844	1.977619	-0.394953
C	2.751261	-0.708391	0.211592
H	-4.886658	0.178307	0.695253
H	-4.373652	2.513544	-0.005674
H	-2.075671	3.125471	-0.665983
C	3.596979	1.527251	-0.161474
C	3.820532	0.179608	0.155680
H	4.429635	2.217494	-0.222487
H	4.826169	-0.176359	0.344866
H	0.021985	2.545163	-1.030569
H	2.131893	3.019340	-0.632942
H	2.934048	-1.754064	0.435175
H	-3.087767	-1.513979	0.696105
O	0.812412	1.536778	1.699268
H	1.626848	1.243467	2.133330

TS[PTE-OH•] - Site 2
Solvent = Water
E = -861.056398
G = -860.910034
ZPC = 0.188281
TCE = 0.201472
TCH = 0.202416
TCG = 0.146364
N_{imag} = 1, -413.40 cm^{-1}

Te	-0.365734	-1.630415	0.275715
N	0.083164	1.603953	0.398567
C	1.205138	0.957877	-0.099472
C	1.254417	-0.428270	-0.325696
C	-3.945667	0.765553	-0.488125
C	-3.503708	2.075475	-0.319128
C	-2.167388	2.331869	-0.045764
C	-1.253263	1.280286	0.089631
C	-1.700985	-0.036684	-0.058040
C	-3.038667	-0.282000	-0.366961
C	2.366093	1.728970	-0.335290
C	2.404465	-0.995929	-0.856752
H	-4.985214	0.559167	-0.711923
H	-4.196754	2.903286	-0.411549
H	-1.816029	3.352326	0.067974
C	3.515393	1.134551	-0.877350
C	3.539802	-0.222133	-1.134935
H	4.382709	1.753060	-1.074221
H	4.424418	-0.692445	-1.545864
H	0.224706	2.601885	0.510897
H	2.305275	2.805467	-0.227894
H	2.429413	-2.063670	-1.046357
H	-3.375435	-1.304731	-0.497118
O	2.975599	1.636359	1.866552
H	3.184334	0.691290	1.783805

Solvent = Pentyl Ethanoate
E = -861.053213
G = -860.906237
ZPC = 0.188687
TCE = 0.201816
TCH = 0.202760
TCG = 0.146976
N_{imag} = 1, -336.33 cm^{-1}

Te	-0.393641	-1.634503	0.289714
N	0.110305	1.592243	0.331893
C	1.228721	0.914622	-0.115062
C	1.262840	-0.477611	-0.298143
C	-3.939752	0.803814	-0.483370
C	-3.476889	2.108151	-0.334785
C	-2.133438	2.347394	-0.083657
C	-1.232308	1.285126	0.053768
C	-1.702672	-0.026830	-0.071222
C	-3.046493	-0.255304	-0.361584
C	2.411917	1.665451	-0.336504
C	2.417466	-1.080857	-0.776166
H	-4.985051	0.610204	-0.691718
H	-4.159192	2.945004	-0.427274
H	-1.765750	3.364330	0.010124
C	3.565497	1.029280	-0.834114
C	3.575366	-0.333818	-1.040968
H	4.446353	1.628375	-1.030640
H	4.463758	-0.831517	-1.409952
H	0.274667	2.577308	0.499002
H	2.343998	2.745438	-0.352462
H	2.429299	-2.153767	-0.937218
H	-3.401522	-1.273867	-0.477434
O	2.919439	1.921639	1.705152
H	3.113572	0.987298	1.878823

TS[PTE-OH•] - Site 3
Solvent = Water
E = -861.054010
G = -860.906299
ZPC = 0.188870
TCE = 0.201778
TCH = 0.202722
TCG = 0.147711
N_{imag} = 1, -606.07 cm^{-1}

Te	0.459447	-1.585990	-0.054940
N	0.038969	1.724808	0.230683
C	-1.137998	1.005295	0.298516
C	-1.189460	-0.416878	0.216114
C	4.096174	0.738189	-0.181921
C	3.668041	2.064406	-0.053972
C	2.326485	2.357556	0.082231
C	1.359334	1.333279	0.094081
C	1.791811	0.005374	-0.039510
C	3.157639	-0.277735	-0.174344
C	-2.317602	1.718370	0.480220
C	-2.440838	-1.065707	0.356219
H	5.148619	0.506678	-0.287269
H	4.389563	2.872750	-0.061285
H	1.993336	3.385520	0.180225
C	-3.571899	1.079287	0.449372
C	-3.596691	-0.353980	0.506515
H	-4.433308	1.624031	0.808895
H	-4.544023	-0.866073	0.621105
H	-0.088619	2.727844	0.305694
H	-2.264814	2.797452	0.572755
H	-2.469293	-2.150409	0.353878
H	3.475981	-1.310050	-0.274834
O	-4.326598	1.351461	-1.412950
H	-3.628662	0.873665	-1.874603

Solvent = Pentyl Ethanoate
E = -861.048967
G = -860.902237
ZPC = 0.188453
TCE = 0.201531
TCH = 0.202476
TCG = 0.146730
N_{imag} = 1, -446.47 cm^{-1}

Te	0.456682	-1.609483	-0.230779
N	0.032181	1.562472	-0.653607
C	-1.106271	0.991797	-0.055174
C	-1.167964	-0.381893	0.259177
C	4.016902	0.828565	0.491906
C	3.583790	2.119559	0.202053
C	2.260551	2.352254	-0.146377
C	1.351175	1.292942	-0.243920
C	1.794605	-0.006138	0.026740
C	3.114638	-0.227097	0.415114

C	-2.216299	1.787360	0.189536
C	-2.322306	-0.919821	0.840045
H	5.045454	0.643339	0.777012
H	4.273961	2.953210	0.260225
H	1.916528	3.361349	-0.350543
C	-3.418750	1.229444	0.671396
C	-3.435369	-0.131185	1.059971
H	-4.200539	1.892905	1.014087
H	-4.333073	-0.553891	1.493385
H	-0.104727	2.541113	-0.875996
H	-2.177122	2.847010	-0.039702
H	-2.341296	-1.969522	1.111856
H	3.443214	-1.235049	0.645062
O	-4.534910	1.031331	-1.029957
H	-3.944039	0.409974	-1.482796

TS[PTE-OH•] - Site 4
Solvent = Water
E = -861.058568
G = -860.911006
ZPC = 0.188976
TCE = 0.201953
TCH = 0.202897
TCG = 0.147562
N_{imag} = 1, -346.29 cm^{-1}

Te	-0.317939	-1.570479	-0.064858
N	-0.213270	1.799185	-0.086114
C	1.004799	1.217627	-0.236885
C	1.237248	-0.187162	-0.259663
C	-4.157440	0.425816	0.237041
C	-3.858700	1.789444	0.230186
C	-2.545676	2.207788	0.124107
C	-1.502493	1.272635	0.024162
C	-1.797874	-0.095243	0.036072
C	-3.130908	-0.501825	0.140905
C	2.109978	2.099870	-0.398080
C	2.524509	-0.657638	-0.415539
H	-5.183499	0.088595	0.318021
H	-4.650172	2.524798	0.306872
H	-2.304012	3.265913	0.116625
C	3.377727	1.615528	-0.540118
C	3.624615	0.217886	-0.482916
H	4.207211	2.299904	-0.668041
H	4.595575	-0.167055	-0.756271
H	-0.190347	2.814457	-0.086830
H	1.916839	3.167393	-0.416456
H	2.702473	-1.727018	-0.450381
H	-3.363100	-1.561683	0.148529
O	4.426153	0.181034	1.598661
H	3.570815	0.488673	1.918418

Solvent = Pentyl Ethanoate
E = -861.052174
G = -860.905470
ZPC = 0.188572
TCE = 0.201701
TCH = 0.202646
TCG = 0.146704
N_{imag} = 1, -342.40 cm^{-1}

Te	-0.262500	-1.566281	0.134075
N	-0.252112	1.663549	0.624723
C	0.959636	1.232423	0.105266
C	1.199439	-0.106877	-0.265221
C	-4.130314	0.400372	-0.408390
C	-3.869926	1.728069	-0.081235
C	-2.580265	2.127089	0.238958
C	-1.531657	1.200382	0.264925
C	-1.797609	-0.138292	-0.041904
C	-3.089678	-0.522022	-0.397043
C	1.997468	2.176797	-0.031088
C	2.427953	-0.465524	-0.788084
H	-5.132474	0.084189	-0.671554
H	-4.668788	2.460322	-0.087163
H	-2.371070	3.165793	0.474766
C	3.231319	1.804295	-0.514550
C	3.488576	0.461167	-0.864164
H	4.018534	2.541993	-0.612082
H	4.387315	0.203210	-1.405505
H	-0.247730	2.644808	0.876285
H	1.803099	3.208810	0.242127
H	2.605852	-1.490159	-1.095743
H	-3.286321	-1.557489	-0.653879
O	4.578189	-0.113000	0.851649
H	3.851208	0.057001	1.469793

TS[PTE-OH•] - Site 5
Solvent = Water
E = -861.052145
G = -860.905561
ZPC = 0.188508
TCE = 0.201675
TCH = 0.202619
TCG = 0.146584
N_{imag} = 1, -496.45 cm^{-1}

Te	0.078269	-1.445706	0.239251
N	0.319694	1.882263	0.093363
C	-0.984585	1.406927	0.133885
C	-1.316809	0.034436	0.200933
C	4.087425	0.063000	-0.254276
C	3.930718	1.454233	-0.286338
C	2.680259	2.025150	-0.174960
C	1.527413	1.226792	-0.023442
C	1.691257	-0.168032	0.013070
C	2.968904	-0.734615	-0.106623
C	-2.010236	2.352056	0.153773
C	-2.686107	-0.389190	0.125683
H	5.069322	-0.384178	-0.342841
H	4.796587	2.095417	-0.402041
H	2.563367	3.103176	-0.204969
C	-3.340208	1.932870	0.299842
C	-3.685804	0.600457	0.343637
H	-4.111521	2.689444	0.400144
H	-4.715754	0.294227	0.475873
H	0.387608	2.892589	0.056688
H	-1.759950	3.406104	0.118223
H	-2.905957	-1.410383	0.412705
H	3.072556	-1.814092	-0.079184
O	-2.892062	-0.950015	-1.739953
H	-2.740982	-0.095817	-2.160686

Solvent = Pentyl Ethanoate
E = -861.049238
G = -860.903455

ZPC = 0.187871
TCE = 0.201101
TCH = 0.202046
TCG = 0.145783
N_{imag} = 1, -443.98 cm^{-1}

Te	0.124520	-1.538399	0.414768
N	0.266575	1.648705	0.904495
C	-1.021097	1.264293	0.486043
C	-1.341518	-0.062907	0.202551
C	3.857838	0.351280	-0.869500
C	3.681414	1.684570	-0.510087
C	2.482455	2.105929	0.048679
C	1.448895	1.194065	0.289928
C	1.637108	-0.149784	-0.050493
C	2.827396	-0.557015	-0.649809
C	-2.028375	2.236685	0.413197
C	-2.644790	-0.400609	-0.236426
H	4.786358	0.019832	-1.318419
H	4.473768	2.404975	-0.677120
H	2.336428	3.149644	0.309367
C	-3.341736	1.890834	0.090732
C	-3.663285	0.582028	-0.212025
H	-4.105000	2.659765	0.076012
H	-4.675980	0.300233	-0.471508
H	0.314576	2.631714	1.141657
H	-1.778047	3.269950	0.633296
H	-2.922838	-1.445507	-0.289981
H	2.955151	-1.596597	-0.932432
O	-2.380734	-0.519437	-2.250488
H	-2.045453	0.381087	-2.383079

TS[PTE-OH•] - Site 5a
Solvent = Water
E = -861.059723
G = -860.911812
ZPC = 0.188889
TCE = 0.201887
TCH = 0.202831
TCG = 0.147911
N_{imag} = 1, -141.73 cm^{-1}

Te	-0.069575	-1.576364	-0.230860
N	-0.059256	1.748850	-0.514756
C	1.179994	1.224815	-0.310101
C	1.407263	-0.127962	0.084156
C	-3.935500	0.353786	0.274765
C	-3.673961	1.711724	0.089503
C	-2.382100	2.141633	-0.156524
C	-1.323813	1.222952	-0.230747
C	-1.584756	-0.141352	-0.051671
C	-2.892457	-0.559137	0.206857
C	2.290462	2.090606	-0.458504
C	2.745407	-0.589398	0.199329
H	-4.943269	0.010045	0.473262
H	-4.477119	2.436685	0.143095
H	-2.170005	3.197042	-0.293715
C	3.567674	1.623444	-0.279513
C	3.803740	0.267189	0.046541
H	4.404424	2.301461	-0.397375
H	4.818925	-0.092741	0.159058
H	-0.064834	2.741411	-0.726284
H	2.109696	3.128710	-0.717154
H	2.913997	-1.634884	0.432903
H	-3.091155	-1.615286	0.354179
O	0.950457	0.374929	2.173073
H	1.716650	0.947335	2.310940

Solvent = Pentyl Ethanoate
E = -861.051750
G = -860.904468
ZPC = 0.188432
TCE = 0.201568
TCH = 0.202512
TCG = 0.147282
N_{imag} = 1, -266.63 cm^{-1}

Te	-0.067572	-1.578336	-0.266137
N	-0.053882	1.694447	-0.659764
C	1.192917	1.205239	-0.350819
C	1.412350	-0.125998	0.092380
C	-3.908768	0.386644	0.388417
C	-3.644796	1.728942	0.131626
C	-2.358869	2.135230	-0.191298
C	-1.314702	1.206875	-0.284571
C	-1.583479	-0.144600	-0.039650
C	-2.873628	-0.538405	0.309595
C	2.295969	2.073096	-0.467006
C	2.738761	-0.563489	0.320362
H	-4.907861	0.061607	0.652505
H	-4.437316	2.465434	0.193691
H	-2.147286	3.183675	-0.375883
C	3.574110	1.627221	-0.201822
C	3.804938	0.298635	0.196742
H	4.406947	2.313567	-0.302280
H	4.812714	-0.045310	0.394224
H	-0.058295	2.677288	-0.902675
H	2.124501	3.099755	-0.774255
H	2.900475	-1.593592	0.619414
H	-3.072204	-1.584290	0.518547
O	0.826671	0.382421	2.085353
H	1.647033	0.838510	2.327640

PTE-OOH• - Site 2a
Solvent = Water
E = -936.212815
G = -936.059796
ZPC = 0.194733
TCE = 0.208379
TCH = 0.209323
TCG = 0.153018
N_{imag} = 0

Te	-0.233243	-1.662274	-0.039646
N	-0.027363	1.636498	-0.657684
C	1.120549	1.089964	0.017833
C	1.363595	-0.360109	-0.307097
C	-4.015423	0.473174	0.063911
C	-3.690557	1.792908	-0.246709
C	-2.369240	2.151897	-0.460493
C	-1.338402	1.204433	-0.381236
C	-1.668188	-0.124605	-0.084287
C	-3.002318	-0.472585	0.144247
C	2.314844	1.955642	-0.248510
C	2.607953	-0.819638	-0.666017
H	-5.043916	0.183583	0.241292

H	-4.465846	2.547090	-0.313846
H	-2.110912	3.180795	-0.690263
C	3.519311	1.447104	-0.595715
C	3.698403	0.050042	-0.798745
H	4.361343	2.115022	-0.737799
H	4.668288	-0.337374	-1.082153
H	0.029840	2.647264	-0.725687
H	2.163313	3.020923	-0.102761
H	2.747579	-1.876819	-0.867872
H	-3.248942	-1.502978	0.379969
O	0.767314	1.216216	1.449888
O	1.790046	0.652752	2.251128
H	1.517371	-0.275856	2.333277

Solvent = Pentyl Ethanoate
E = -936.209735
G = -936.056947
ZPC = 0.194623
TCE = 0.208323
TCH = 0.209267
TCG = 0.152788
N_{imag} = 0

Te	-0.234351	-1.648286	-0.029924
N	-0.016913	1.671107	-0.542860
C	1.134233	1.082712	0.089990
C	1.357655	-0.347276	-0.335963
C	-4.021770	0.472743	-0.006297
C	-3.690837	1.798012	-0.282310
C	-2.365199	2.165707	-0.441558
C	-1.330022	1.224146	-0.338594
C	-1.668649	-0.111917	-0.082929
C	-3.007504	-0.469570	0.089533
C	2.332581	1.951630	-0.157443
C	2.568793	-0.783707	-0.809346
H	-5.054662	0.176207	0.129428
H	-4.466320	2.550698	-0.364955
H	-2.106545	3.200039	-0.644602
C	3.507939	1.464621	-0.616455
C	3.657497	0.090109	-0.946549
H	4.351047	2.134383	-0.744034
H	4.603221	-0.279232	-1.321549
H	0.049471	2.679853	-0.607516
H	2.210919	3.000484	0.095842
H	2.687277	-1.823118	-1.098902
H	-3.259349	-1.505908	0.293450
O	0.798431	1.131492	1.518902
O	1.818710	0.502390	2.272302
H	1.504192	-0.414587	2.316820

PTE-OOH$^{\bullet}$ - Site 2
Solvent = Water
E = -936.214801
G = -936.062893
ZPC = 0.195054
TCE = 0.209075
TCH = 0.210019
TCG = 0.151909
N_{imag} = 0

Te	-0.711439	-1.662878	0.299747
N	0.122378	1.521291	0.137931
C	1.141185	0.682016	-0.253408
C	1.041812	-0.695432	-0.365853
C	-4.028572	1.100149	-0.376977
C	-3.432112	2.358032	-0.331835
C	-2.058640	2.471928	-0.172364
C	-1.260098	1.330381	-0.031989
C	-1.859601	0.066440	-0.056610
C	-3.236977	-0.036486	-0.248416
C	2.451131	1.388920	-0.489105
C	2.116335	-1.453385	-0.833208
H	-5.099031	1.002294	-0.510734
H	-4.033212	3.253961	-0.431391
H	-1.585580	3.448475	-0.154909
C	3.517499	0.513501	-1.048120
C	3.345167	-0.821891	-1.186343
H	4.450402	0.996776	-1.314731
H	4.149690	-1.434214	-1.578778
H	0.389928	2.498425	0.187784
H	2.289197	2.274714	-1.117266
H	2.020395	-2.528039	-0.928673
H	-3.696665	-1.018405	-0.281739
O	2.891819	2.047428	0.738765
O	3.168904	1.062313	1.724881
H	2.344491	1.023652	2.234298

Solvent = Pentyl Ethanoate
E = -936.213979
G = -936.062122
ZPC = 0.194922
TCE = 0.208890
TCH = 0.209834
TCG = 0.151857
N_{imag} = 0

Te	-0.733181	-1.668562	0.315441
N	0.159745	1.484372	0.144463
C	1.160280	0.623213	-0.263941
C	1.040898	-0.748446	-0.356612
C	-3.994293	1.149265	-0.412228
C	-3.375353	2.394875	-0.357805
C	-2.001524	2.482843	-0.185359
C	-1.225884	1.327199	-0.037309
C	-1.849618	0.075159	-0.068168
C	-3.225406	-0.001813	-0.277191
C	2.469185	1.321152	-0.543481
C	2.115608	-1.533431	-0.787345
H	-5.064864	1.071685	-0.558529
H	-3.958855	3.302057	-0.461759
H	-1.513526	3.452259	-0.163590
C	3.564603	0.402292	-0.959233
C	3.373662	-0.932197	-1.078284
H	4.525449	0.859407	-1.166155
H	4.192812	-1.568475	-1.395109
H	0.451748	2.453694	0.195455
H	2.311341	2.107636	-1.297330
H	2.001573	-2.606892	-0.876326
H	-3.703810	-0.974527	-0.320207
O	2.835578	2.146702	0.592499
O	3.072043	1.312928	1.716736
H	2.191402	1.240085	2.117248

PTE-OOH$^{\bullet}$ - Site 3
Solvent = Water
E = -936.211016

G = -936.058399
ZPC = 0.195222
TCE = 0.209024
TCH = 0.209968
TCG = 0.152617
N_{imag} = 0

Te	-0.775173	-1.620789	0.218064
N	-0.130811	1.549467	0.559673
C	0.958901	0.887594	-0.045341
C	0.904560	-0.520416	-0.304110
C	-4.201170	1.022157	-0.384939
C	-3.671994	2.294399	-0.177396
C	-2.321613	2.454255	0.099960
C	-1.475044	1.342975	0.200535
C	-2.012134	0.065586	0.003935
C	-3.364181	-0.085292	-0.303073
C	2.089039	1.592036	-0.334599
C	2.031201	-1.167025	-0.882431
H	-5.252478	0.892279	-0.611158
H	-4.309483	3.168425	-0.241456
H	-1.904519	3.445126	0.248085
C	3.342044	0.942937	-0.806942
C	3.182009	-0.499898	-1.145820
H	3.773713	1.495578	-1.650439
H	4.032357	-1.008439	-1.585625
H	0.070684	2.533039	0.702533
H	2.114300	2.664103	-0.164122
H	1.955527	-2.223346	-1.119212
H	-3.765999	-1.079357	-0.467287
O	4.424253	1.122000	0.165662
O	4.098328	0.438947	1.366977
H	3.560201	1.083905	1.851833

Solvent = Pentyl Ethanoate
E = -936.209340
G = -936.057142
ZPC = 0.195011
TCE = 0.208878
TCH = 0.209822
TCG = 0.152198
N_{imag} = 0

Te	-0.767020	-1.613426	0.216842
N	-0.142447	1.557498	0.519033
C	0.957769	0.895295	-0.059304
C	0.905002	-0.512602	-0.324524
C	-4.221482	0.997378	-0.361265
C	-3.699282	2.273265	-0.163903
C	-2.346588	2.444082	0.092378
C	-1.488856	1.340403	0.184896
C	-2.020518	0.058704	-0.000038
C	-3.374741	-0.102749	-0.288761
C	2.097212	1.594843	-0.323607
C	2.035692	-1.159058	-0.892056
H	-5.274756	0.859040	-0.573196
H	-4.344241	3.142434	-0.221363
H	-1.937330	3.440219	0.227653
C	3.357025	0.946176	-0.786474
C	3.193553	-0.495865	-1.131704
H	3.781489	1.497530	-1.635790
H	4.050747	-1.008143	-1.553612
H	0.056815	2.535020	0.694851
H	2.121292	2.668291	-0.158892
H	1.961758	-2.214732	-1.133355
H	-3.772208	-1.099683	-0.447495
O	4.431088	1.132105	0.180360
O	4.115811	0.427021	1.370047
H	3.494686	1.023433	1.815100

PTE-OOH• - Site 4
Solvent = Water
E = -936.214751
G = -936.062214
ZPC = 0.195428
TCE = 0.209294
TCH = 0.210238
TCG = 0.152537
N_{imag} = 0

Te	-0.507029	-1.570031	0.061321
N	-0.433901	1.738397	0.382271
C	0.771199	1.224979	-0.050414
C	1.005415	-0.155575	-0.325202
C	-4.382892	0.412744	-0.168920
C	-4.094830	1.757335	0.060092
C	-2.782367	2.168442	0.228902
C	-1.729152	1.242489	0.189616
C	-2.018734	-0.111193	-0.021306
C	-3.342989	-0.508158	-0.213897
C	1.841929	2.149244	-0.252012
C	2.218585	-0.584763	-0.761716
H	-5.404978	0.083785	-0.311459
H	-4.892078	2.490160	0.097538
H	-2.552289	3.216680	0.392317
C	3.068447	1.751619	-0.663902
C	3.406979	0.312081	-0.841832
H	3.852194	2.479686	-0.839691
H	3.980027	0.139868	-1.759781
H	-0.411157	2.738620	0.547170
H	1.634799	3.205060	-0.101165
H	2.380311	-1.632117	-0.995567
H	-3.561029	-1.556308	-0.390133
O	4.425665	-0.095444	0.140664
O	3.894994	0.020249	1.452763
H	4.042219	0.953500	1.672298

Solvent = Pentyl Ethanoate
E = -936.212823
G = -936.060747
ZPC = 0.195233
TCE = 0.209205
TCH = 0.210149
TCG = 0.152076
N_{imag} = 0

Te	-0.500200	-1.561288	0.071349
N	-0.456836	1.734109	0.399823
C	0.758415	1.235274	-0.024769
C	1.000941	-0.139858	-0.322776
C	-4.391376	0.387453	-0.197356
C	-4.116124	1.731204	0.047050
C	-2.808400	2.150852	0.230030
C	-1.747300	1.234343	0.192862
C	-2.025431	-0.119843	-0.031719
C	-3.343796	-0.524483	-0.241367

C	1.829520	2.163681	-0.191625
C	2.213468	-0.556607	-0.766458
H	-5.409632	0.052045	-0.352533
H	-4.919652	2.457363	0.084277
H	-2.590436	3.200180	0.403848
C	3.058757	1.779170	-0.610147
C	3.400235	0.346242	-0.848698
H	3.841993	2.514201	-0.760847
H	3.930848	0.217182	-1.800308
H	-0.439954	2.726302	0.602131
H	1.624575	3.215182	-0.008392
H	2.383073	-1.601029	-1.008478
H	-3.552983	-1.572268	-0.431269
O	4.452044	-0.089330	0.066674
O	3.961906	-0.056123	1.397690
H	4.055376	0.878094	1.637586

PTE-OOH$^{\bullet}$ - Site 5

Solvent = Water
E = -936.211729
G = -936.059693
ZPC = 0.194843
TCE = 0.208752
TCH = 0.209696
TCG = 0.152035
N_{imag} = 0

Te	-0.129654	-1.466295	0.094610
N	-0.613758	1.642501	0.857343
C	0.635989	1.452571	0.225104
C	1.060016	0.226716	-0.208145
C	-4.219140	-0.026406	-0.545615
C	-4.164358	1.297986	-0.118276
C	-2.963725	1.844354	0.315421
C	-1.800527	1.066580	0.358258
C	-1.861233	-0.269128	-0.053982
C	-3.062109	-0.799043	-0.523680
C	1.482930	2.585952	0.097483
C	2.394765	0.030374	-0.853784
H	-5.150870	-0.455179	-0.894102
H	-5.055658	1.914271	-0.132510
H	-2.914507	2.881458	0.631250
C	2.780071	2.455894	-0.451068
C	3.239321	1.261337	-0.905684
H	3.408697	3.336969	-0.512176
H	4.231446	1.157164	-1.330647
H	-0.753789	2.612621	1.115632
H	1.130852	3.549625	0.446835
H	2.273226	-0.387383	-1.864017
H	-3.096053	-1.830326	-0.857780
O	3.110920	-1.065545	-0.223868
O	3.344503	-0.749296	1.141563
H	4.189589	-0.273793	1.118661

Solvent = Pentyl Ethanoate
E = -936.210426
G = -936.058638
ZPC = 0.194590
TCE = 0.208580
TCH = 0.209525
TCG = 0.151788
N_{imag} = 0

Te	-0.115216	-1.447889	0.098436
N	-0.630163	1.668206	0.808923
C	0.630102	1.475975	0.207612
C	1.060622	0.246622	-0.214678
C	-4.225545	-0.065993	-0.529895
C	-4.185901	1.261393	-0.112855
C	-2.988530	1.829223	0.301102
C	-1.812945	1.069966	0.337659
C	-1.859550	-0.271407	-0.059963
C	-3.056941	-0.820803	-0.513548
C	1.488042	2.600103	0.100739
C	2.399992	0.043312	-0.848677
H	-5.154446	-0.511112	-0.865535
H	-5.086716	1.864088	-0.121568
H	-2.953604	2.870753	0.605586
C	2.802024	2.458271	-0.406581
C	3.264023	1.263139	-0.854530
H	3.441203	3.333600	-0.442297
H	4.265656	1.155810	-1.257285
H	-0.774783	2.626518	1.100572
H	1.137630	3.568042	0.440359
H	2.276069	-0.331091	-1.876938
H	-3.080087	-1.854794	-0.841159
O	3.080540	-1.087963	-0.255895
O	3.305109	-0.823517	1.121677
H	4.153891	-0.356021	1.112573

PTE-OOH$^{\bullet}$ - Site 5a

Solvent = Water
E = -936.252145
G = -936.100451
ZPC = 0.195380
TCE = 0.209667
TCH = 0.210611
TCG = 0.151694
N_{imag} = 0

Te	-0.176351	-1.921502	0.043714
N	0.076460	1.134747	-1.340582
C	1.313455	0.914862	-0.719990
C	1.513790	0.971149	0.670481
C	-3.749009	0.552840	0.303609
C	-3.449481	1.706097	-0.414326
C	-2.172240	1.890773	-0.930330
C	-1.181301	0.913423	-0.767481
C	-1.494166	-0.273144	-0.077094
C	-2.768364	-0.419737	0.477104
C	2.431655	0.652084	-1.521998
C	2.754920	0.696269	1.234319
H	-4.734953	0.407364	0.728118
H	-4.200161	2.474138	-0.558482
H	-1.921122	2.798917	-1.469069
C	3.674937	0.402329	-0.962288
C	3.839579	0.396366	0.423104
H	4.517165	0.192024	-1.610726
H	4.805032	0.181318	0.863539
H	0.095272	1.805229	-2.098987
H	2.294246	0.619944	-2.597058
H	2.847147	0.738062	2.313360
H	-3.003245	-1.320121	1.034257
O	0.477811	1.262301	1.546372
O	0.080594	2.628462	1.357307
H	-0.814212	2.532050	0.985922

Solvent = Pentyl Ethanoate
E = -936.253592
G = -936.101347
ZPC = 0.195599
TCE = 0.209795
TCH = 0.210739
TCG = 0.152245
N_{imag} = 0

Te	-0.126531	-1.910582	0.052321
N	0.046169	1.147495	-1.330969
C	1.290053	0.934163	-0.718383
C	1.494491	1.020559	0.670988
C	-3.764494	0.467762	0.307184
C	-3.495939	1.626246	-0.413294
C	-2.222815	1.843739	-0.927172
C	-1.206243	0.891160	-0.765476
C	-1.487536	-0.299401	-0.068503
C	-2.757514	-0.476424	0.485236
C	2.397290	0.642321	-1.523551
C	2.739963	0.748249	1.227825
H	-4.746228	0.297681	0.732522
H	-4.266832	2.373799	-0.559807
H	-1.996686	2.758799	-1.466488
C	3.643676	0.397198	-0.968506
C	3.816190	0.422918	0.414922
H	4.479476	0.162915	-1.617295
H	4.783439	0.211160	0.853712
H	0.051139	1.809436	-2.095652
H	2.247031	0.575731	-2.595523
H	2.841412	0.812341	2.304935
H	-2.967434	-1.378439	1.049975
O	0.471273	1.320862	1.542977
O	0.029231	2.663451	1.298877
H	-0.855672	2.508917	0.927243

TS[PTE-OOH•] - Site 2a
Solvent = Water
E = -936.200679
G = -936.049656
ZPC = 0.193235
TCE = 0.207010
TCH = 0.207954
TCG = 0.151022
N_{imag} = 1, -464.44 cm^{-1}

Te	-0.283235	-1.692338	-0.086271
N	-0.082856	1.540878	-0.881848
C	1.126193	1.019880	-0.427608
C	1.334689	-0.401754	-0.287653
C	-3.990662	0.543121	0.322885
C	-3.667525	1.834073	-0.090262
C	-2.368186	2.141069	-0.464460
C	-1.364755	1.163517	-0.446663
C	-1.693333	-0.135994	-0.047405
C	-3.000288	-0.430867	0.344935
C	2.286716	1.828355	-0.717939
C	2.624203	-0.900753	-0.202543
H	-5.001048	0.295944	0.624694
H	-4.425877	2.607699	-0.114127
H	-2.109867	3.148086	-0.776657
C	3.545340	1.304829	-0.636397
C	3.729524	-0.066216	-0.359875
H	4.405648	1.941529	-0.803741
H	4.729045	-0.478030	-0.296278
H	-0.022615	2.531261	-1.089646
H	2.126286	2.877264	-0.944281
H	2.775282	-1.962024	-0.035156
H	-3.245554	-1.438932	0.663048
O	0.901950	1.465822	1.415707
O	1.962650	1.056577	2.223679
H	1.788638	0.117852	2.393971

Solvent = Pentyl Ethanoate
E = -936.195465
G = -936.045052
ZPC = 0.192994
TCE = 0.206864
TCH = 0.207808
TCG = 0.150412
N_{imag} = 1, -600.87 cm^{-1}

Te	-0.296620	-1.700244	-0.089503
N	-0.077396	1.495758	-0.928103
C	1.134502	0.998670	-0.427342
C	1.341348	-0.420751	-0.253779
C	-3.978369	0.575213	0.361305
C	-3.648612	1.851626	-0.086010
C	-2.351063	2.137423	-0.483716
C	-1.357112	1.151678	-0.462911
C	-1.696061	-0.134809	-0.031564
C	-2.996449	-0.407974	0.390694
C	2.300168	1.788610	-0.747224
C	2.624414	-0.915384	-0.119275
H	-4.986320	0.345955	0.685006
H	-4.399345	2.632747	-0.115405
H	-2.088769	3.136796	-0.817072
C	3.558064	1.269248	-0.620303
C	3.736681	-0.084547	-0.281324
H	4.421290	1.900195	-0.795540
H	4.734039	-0.493672	-0.176416
H	-0.013085	2.475339	-1.175512
H	2.147299	2.829309	-1.013435
H	2.776288	-1.970860	0.083002
H	-3.246885	-1.405345	0.738107
O	0.901258	1.518241	1.336242
O	1.953779	1.157904	2.156011
H	1.776147	0.228738	2.368807

TS[PTE-OOH•] - Site 2
Solvent = Water
E = -936.200960
G = -936.050028
ZPC = 0.193859
TCE = 0.207744
TCH = 0.208688
TCG = 0.150932
N_{imag} = 1, -506.57 cm^{-1}

Te	-0.576691	-1.642045	0.239696
N	0.003866	1.604780	0.117416
C	1.075602	0.866226	-0.325183
C	1.062018	-0.529150	-0.477363
C	-4.117001	0.867167	-0.271739
C	-3.617024	2.166187	-0.224657

C	-2.251794	2.382318	-0.108293
C	-1.363982	1.303921	-0.014462
C	-1.865899	-0.001463	-0.040545
C	-3.237402	-0.206918	-0.188963
C	2.306454	1.577896	-0.531242
C	2.175168	-1.172140	-0.995280
H	-5.180831	0.689282	-0.371421
H	-4.287567	3.014805	-0.288372
H	-1.854947	3.391953	-0.088349
C	3.389257	0.902558	-1.166176
C	3.336026	-0.451512	-1.358888
H	4.268300	1.475083	-1.435309
H	4.172450	-0.981003	-1.799044
H	0.189921	2.600218	0.176932
H	2.242765	2.657703	-0.622426
H	2.156525	-2.248261	-1.127572
H	-3.620992	-1.220797	-0.224106
O	2.883586	1.773808	1.266663
O	3.147209	0.520543	1.810554
H	2.280356	0.188542	2.092573

Solvent = Pentyl Ethanoate
E = -936.197989
G = -936.046869
ZPC = 0.193850
TCE = 0.207680
TCH = 0.208624
TCG = 0.151120
N_{imag} = 1, -601.87 cm^{-1}

Te	-0.580420	-1.638856	0.261612
N	0.016239	1.571028	0.193476
C	1.079254	0.843564	-0.311834
C	1.061243	-0.543385	-0.474083
C	-4.100183	0.886513	-0.334813
C	-3.591839	2.179820	-0.254539
C	-2.228606	2.384756	-0.097271
C	-1.351678	1.299477	0.011591
C	-1.865340	-0.000650	-0.046371
C	-3.231393	-0.195487	-0.241970
C	2.305327	1.558981	-0.534816
C	2.165842	-1.192573	-1.015291
H	-5.161964	0.719090	-0.469518
H	-4.254473	3.034207	-0.327154
H	-1.828092	3.392553	-0.058360
C	3.378812	0.879756	-1.183894
C	3.321737	-0.474670	-1.382276
H	4.258321	1.449701	-1.457126
H	4.155807	-1.001954	-1.829820
H	0.218107	2.557044	0.307161
H	2.233184	2.636016	-0.654968
H	2.138079	-2.267274	-1.158064
H	-3.621302	-1.205694	-0.306802
O	2.889162	1.795632	1.213848
O	3.115428	0.564601	1.799632
H	2.234736	0.271129	2.082076

TS[PTE-OOH$^{\bullet}$] - Site 3
Solvent = Water
E = -936.197260
G = -936.046069
ZPC = 0.193727
TCE = 0.207475
TCH = 0.208420
TCG = 0.151191
N_{imag} = 1, -578.37 cm^{-1}

Te	-0.636717	-1.611793	0.186973
N	-0.156989	1.597913	0.480547
C	0.940302	0.980930	-0.144400
C	0.963714	-0.415891	-0.402632
C	-4.225796	0.832797	-0.294184
C	-3.763356	2.133400	-0.105943
C	-2.414700	2.371320	0.119137
C	-1.503079	1.310548	0.186264
C	-1.972473	0.004226	0.012860
C	-3.324054	-0.224568	-0.244206
C	2.042608	1.743103	-0.471783
C	2.080914	-0.994636	-1.033878
H	-5.275618	0.641975	-0.480448
H	-4.451798	2.969390	-0.145176
H	-2.050773	3.385141	0.250056
C	3.244561	1.128529	-0.925572
C	3.190437	-0.243001	-1.330220
H	3.999320	1.766212	-1.371409
H	4.052289	-0.691932	-1.808150
H	-0.002843	2.592198	0.608145
H	2.025132	2.813064	-0.293833
H	2.054832	-2.048868	-1.286625
H	-3.673639	-1.240519	-0.392992
O	4.287039	0.983934	0.634270
O	3.589387	0.242304	1.574632
H	2.965430	0.870730	1.970159

Solvent = Pentyl Ethanoate
E = -936.194329
G = -936.043349
ZPC = 0.193537
TCE = 0.207316
TCH = 0.208261
TCG = 0.150979
N_{imag} = 1, -630.74 cm^{-1}

Te	-0.623913	-1.607476	0.189362
N	-0.173318	1.582233	0.517137
C	0.934808	0.990913	-0.116962
C	0.965273	-0.396269	-0.412949
C	-4.228392	0.815753	-0.319912
C	-3.776331	2.114402	-0.101689
C	-2.431822	2.355349	0.143610
C	-1.515477	1.298830	0.205685
C	-1.976094	-0.006976	0.004106
C	-3.321417	-0.237472	-0.277920
C	2.034280	1.766263	-0.423363
C	2.073657	-0.948092	-1.078296
H	-5.274637	0.623040	-0.524225
H	-4.469265	2.947109	-0.135416
H	-2.076053	3.369969	0.291825
C	3.241987	1.166520	-0.891667
C	3.180027	-0.184822	-1.357373
H	3.988242	1.822546	-1.325801
H	4.039518	-0.616073	-1.855362
H	-0.024281	2.567050	0.702564
H	2.015752	2.832310	-0.221908
H	2.048361	-1.992680	-1.369245
H	-3.664692	-1.251632	-0.452909

O	4.267679	0.977844	0.632051
O	3.611026	0.155828	1.522438
H	2.961091	0.735742	1.948188

TS[PTE-OOH$^{\bullet}$] - Site 4

Solvent = Water
E = -936.202090
G = -936.051211
ZPC = 0.193928
TCE = 0.207782
TCH = 0.208726
TCG = 0.150878
N_{imag} = 1, -433.68 cm^{-1}

Te	-0.482823	-1.579693	0.009730
N	-0.355037	1.753840	0.207937
C	0.827071	1.203175	-0.214394
C	1.027760	-0.184512	-0.438244
C	-4.342045	0.446751	0.004764
C	-4.026540	1.795794	0.151192
C	-2.700808	2.195819	0.206139
C	-1.664240	1.255235	0.132223
C	-1.978610	-0.101432	0.000267
C	-3.317142	-0.488588	-0.076012
C	1.906114	2.097612	-0.451856
C	2.252730	-0.636123	-0.866829
H	-5.374639	0.123927	-0.046846
H	-4.811512	2.539745	0.215003
H	-2.448417	3.246632	0.307018
C	3.127474	1.639771	-0.857690
C	3.374867	0.237051	-0.954724
H	3.932768	2.337705	-1.052304
H	4.253340	-0.103305	-1.489519
H	-0.316465	2.759866	0.330632
H	1.725826	3.161669	-0.335644
H	2.401396	-1.693083	-1.060508
H	-3.558981	-1.540125	-0.189575
O	4.283112	-0.079392	0.714207
O	3.395698	0.215770	1.749622
H	3.438480	1.179776	1.840562

Solvent = Pentyl Ethanoate
E = -936.197808
G = -936.047006
ZPC = 0.193789
TCE = 0.207667
TCH = 0.208611
TCG = 0.150801
N_{imag} = 1, -574.50 cm^{-1}

Te	-0.462051	-1.570624	0.053632
N	-0.373574	1.701624	0.419891
C	0.808636	1.211463	-0.100660
C	1.014781	-0.151319	-0.422354
C	-4.338090	0.428395	-0.166024
C	-4.038493	1.765327	0.081072
C	-2.721274	2.162307	0.255888
C	-1.679466	1.227347	0.211428
C	-1.981498	-0.119307	-0.018539
C	-3.306436	-0.501827	-0.223624
C	1.860098	2.133733	-0.331253
C	2.216060	-0.561152	-0.949021
H	-5.363262	0.111180	-0.313953
H	-4.829123	2.504998	0.127052
H	-2.483772	3.207048	0.430296
C	3.067018	1.719516	-0.827532
C	3.328252	0.328683	-1.024733
H	3.853589	2.439972	-1.018219
H	4.173135	0.042801	-1.640286
H	-0.342642	2.693348	0.622727
H	1.675050	3.187505	-0.144443
H	2.364126	-1.601097	-1.219485
H	-3.533973	-1.544642	-0.418776
O	4.301857	-0.094587	0.522230
O	3.477413	0.074081	1.620053
H	3.516838	1.025020	1.800854

TS[PTE-OOH$^{\bullet}$] - Site 5

Solvent = Water
E = -936.196525
G = -936.044923
ZPC = 0.193874
TCE = 0.207532
TCH = 0.208476
TCG = 0.151602
N_{imag} = 1, -583.97 cm^{-1}

Te	-0.231256	-1.519079	0.021548
N	-0.459733	1.616127	0.794641
C	0.739769	1.345170	0.104301
C	1.055617	0.082196	-0.369584
C	-4.240332	0.188977	-0.402089
C	-4.073954	1.506183	0.018133
C	-2.817472	1.972107	0.382531
C	-1.707987	1.119331	0.363122
C	-1.880935	-0.208995	-0.040707
C	-3.137918	-0.659214	-0.441360
C	1.663379	2.393307	-0.068388
C	2.338440	-0.167208	-0.952609
H	-5.216261	-0.175910	-0.698382
H	-4.921865	2.180226	0.051795
H	-2.684277	3.003673	0.692857
C	2.875822	2.186932	-0.743445
C	3.194339	0.946203	-1.237625
H	3.543384	3.026948	-0.893899
H	4.117749	0.772750	-1.776108
H	-0.523254	2.591881	1.062690
H	1.417547	3.378185	0.314955
H	2.454069	-1.062640	-1.554401
H	-3.257220	-1.685585	-0.770831
O	3.261470	-0.982209	0.437353
O	3.268912	-0.146783	1.540579
H	4.017914	0.451716	1.391222

Solvent = Pentyl Ethanoate
E = -936.194105
G = -936.043283
ZPC = 0.193607
TCE = 0.207425
TCH = 0.208369
TCG = 0.150821
N_{imag} = 1, -624.41 cm^{-1}

Te	-0.209967	-1.500890	0.019677
N	-0.477600	1.633577	0.756957
C	0.736070	1.366902	0.097646

C	1.057177	0.107874	-0.385749
C	-4.246709	0.147987	-0.393069
C	-4.096205	1.467208	0.024108
C	-2.843238	1.952174	0.374002
C	-1.720388	1.116919	0.345931
C	-1.878246	-0.215742	-0.052910
C	-3.131835	-0.683078	-0.441248
C	1.668613	2.410085	-0.039589
C	2.346530	-0.143483	-0.952326
H	-5.219862	-0.232080	-0.679627
H	-4.953802	2.128748	0.065098
H	-2.723939	2.987089	0.679737
C	2.895268	2.203128	-0.689586
C	3.214542	0.970043	-1.201623
H	3.571679	3.040898	-0.814601
H	4.145522	0.801544	-1.729071
H	-0.544712	2.596422	1.062694
H	1.421810	3.392475	0.349980
H	2.455291	-1.018989	-1.584905
H	-3.240278	-1.711347	-0.769593
O	3.217765	-1.014887	0.407043
O	3.205624	-0.228815	1.539286
H	3.953164	0.375979	1.414220

TS[PTE-OOH•] - Site 5a

Solvent = Water
E = -936.202298
G = -936.051571
ZPC = 0.193308
TCE = 0.207307
TCH = 0.208251
TCG = 0.150728
N_{imag} = 1, -396.03 cm^{-1}

Te	-0.234606	-1.638720	-0.215487
N	-0.119795	1.645019	-0.771129
C	1.118901	1.088037	-0.595623
C	1.310761	-0.211212	-0.018453
C	-4.002596	0.481786	0.327294
C	-3.697190	1.808881	0.028985
C	-2.402583	2.164041	-0.311932
C	-1.385647	1.199565	-0.374563
C	-1.692761	-0.134635	-0.079863
C	-2.998295	-0.476747	0.276540
C	2.244803	1.863930	-0.927937
C	2.641568	-0.729739	0.038934
H	-5.010899	0.196157	0.601002
H	-4.466385	2.570862	0.068476
H	-2.158028	3.196934	-0.537306
C	3.518204	1.363138	-0.767665
C	3.719465	0.049351	-0.283724
H	4.370781	1.978556	-1.028095
H	4.724317	-0.345334	-0.194671
H	-0.104280	2.609762	-1.083458
H	2.089438	2.864160	-1.318875
H	2.780244	-1.747090	0.388224
H	-3.229035	-1.509833	0.513879
O	0.941239	0.213928	1.845262
O	1.850793	1.169351	2.311984
H	1.457906	2.019518	2.064142

Solvent = Pentyl Ethanoate
E = -936.196876
G = -936.046761
ZPC = 0.192856
TCE = 0.206970
TCH = 0.207914
TCG = 0.150115
N_{imag} = 1, -546.33 cm^{-1}

Te	-0.229834	-1.631652	-0.234033
N	-0.129539	1.630917	-0.797811
C	1.120203	1.090086	-0.596331
C	1.315098	-0.200816	-0.003344
C	-4.000396	0.480050	0.359633
C	-3.700340	1.803089	0.045381
C	-2.408724	2.156470	-0.311800
C	-1.391945	1.193761	-0.382208
C	-1.695955	-0.137926	-0.073175
C	-2.993875	-0.477003	0.306613
C	2.239335	1.871756	-0.914563
C	2.646758	-0.711548	0.061290
H	-5.004691	0.195701	0.649432
H	-4.469689	2.565066	0.088156
H	-2.168505	3.189606	-0.542514
C	3.520007	1.379392	-0.742499
C	3.724774	0.072308	-0.256978
H	4.369433	2.002213	-0.996584
H	4.730840	-0.316059	-0.153572
H	-0.119490	2.581993	-1.144552
H	2.084583	2.869489	-1.313447
H	2.790633	-1.723072	0.425113
H	-3.220229	-1.506997	0.561912
O	0.929220	0.195022	1.825825
O	1.840124	1.115031	2.317905
H	1.460892	1.973394	2.078503

PTE-OOCH$_3$• - Site 2a

Solvent = Water
E = -975.500239
G = -975.321062
ZPC = 0.222965
TCE = 0.238033
TCH = 0.238977
TCG = 0.179178
N_{imag} = 0

Te	-0.360069	-1.700527	-0.164337
N	-0.157334	1.496955	-1.012347
C	1.036063	1.054929	-0.332228
C	1.267270	-0.430484	-0.423960
C	-4.049097	0.554491	0.309114
C	-3.734816	1.833982	-0.145609
C	-2.442691	2.130614	-0.553249
C	-1.439132	1.153570	-0.533529
C	-1.761145	-0.136364	-0.094784
C	-3.059302	-0.419697	0.336391
C	2.207895	1.853468	-0.822141
C	2.524864	-0.941887	-0.643405
H	-5.052709	0.317176	0.640352
H	-4.493709	2.607118	-0.172964
H	-2.189828	3.129831	-0.894017
C	3.419447	1.296297	-1.046722
C	3.615687	-0.108077	-0.921583
H	4.252460	1.923773	-1.342814
H	4.594638	-0.536974	-1.091801

H	-0.100130	2.489716	-1.216100
H	2.042701	2.922200	-0.920426
H	2.670446	-2.017705	-0.642641
H	-3.298227	-1.418919	0.685901
O	0.788237	1.428098	1.079697
O	1.947640	1.136152	1.852664
C	1.577963	0.184537	2.843166
H	2.470815	0.067339	3.458859
H	0.756725	0.567986	3.452304
H	1.306681	-0.769101	2.387640

Solvent = Pentyl Ethanoate
E = -975.499519
G = -975.321151
ZPC = 0.222684
TCE = 0.237867
TCH = 0.238811
TCG = 0.178367
N_{imag} = 0

Te	-0.359720	-1.689805	-0.155448
N	-0.150516	1.514065	-0.959762
C	1.045424	1.057110	-0.291562
C	1.269791	-0.428205	-0.436330
C	-4.063059	0.545486	0.269374
C	-3.745933	1.827131	-0.175509
C	-2.447777	2.131678	-0.554960
C	-1.437266	1.161665	-0.518611
C	-1.765159	-0.131999	-0.093923
C	-3.068867	-0.423280	0.310950
C	2.216491	1.860671	-0.781394
C	2.509779	-0.941209	-0.727920
H	-5.071668	0.302481	0.581002
H	-4.507949	2.596918	-0.214980
H	-2.194867	3.135021	-0.883616
C	3.412787	1.302484	-1.072110
C	3.597766	-0.106441	-1.016885
H	4.243456	1.935386	-1.364029
H	4.564568	-0.537407	-1.243456
H	-0.089017	2.499378	-1.188661
H	2.066272	2.935578	-0.816288
H	2.646863	-2.017297	-0.776743
H	-3.310888	-1.425158	0.651767
O	0.807472	1.396665	1.115286
O	1.974896	1.079525	1.865817
C	1.581329	0.175167	2.882229
H	2.482193	0.015465	3.477866
H	0.799971	0.612697	3.509191
H	1.239355	-0.772700	2.460633

PTE-OOCH$_3$$^•$ - Site 2
Solvent = Water
E = -975.503605
G = -975.325155
ZPC = 0.223463
TCE = 0.238744
TCH = 0.239688
TCG = 0.178451
N_{imag} = 0

Te	-0.809096	-1.652853	0.292448
N	-0.000688	1.527036	-0.145660
C	0.998941	0.659837	-0.510991
C	0.888180	-0.725011	-0.550151
C	-4.178399	1.077072	-0.226392
C	-3.585836	2.334302	-0.317905
C	-2.204207	2.455104	-0.298913
C	-1.389590	1.323126	-0.166740
C	-1.983664	0.060869	-0.054138
C	-3.372791	-0.050245	-0.103894
C	2.318479	1.328253	-0.791711
C	1.916834	-1.503100	-1.075999
H	-5.256291	0.972513	-0.248834
H	-4.197368	3.223492	-0.414029
H	-1.736126	3.430259	-0.386887
C	3.291916	0.449259	-1.498742
C	3.097558	-0.886749	-1.590867
H	4.187539	0.921893	-1.884874
H	3.843048	-1.512329	-2.069728
H	0.267273	2.505050	-0.163566
H	2.158016	2.267650	-1.333340
H	1.815314	-2.580731	-1.117136
H	-3.829426	-1.031432	-0.030023
O	2.891506	1.855901	0.448747
O	3.250438	0.761726	1.289983
C	2.388198	0.781235	2.421492
H	2.764994	-0.011877	3.068849
H	2.455440	1.741812	2.936160
H	1.356131	0.568046	2.135602

Solvent = Pentyl Ethanoate
E = -975.505694
G = -975.326959
ZPC = 0.223450
TCE = 0.238651
TCH = 0.239595
TCG = 0.178735
N_{imag} = 0

Te	-0.932615	-1.639769	0.417217
N	0.112279	1.414807	-0.124063
C	1.037615	0.465046	-0.504743
C	0.829857	-0.899939	-0.471592
C	-4.081296	1.290609	-0.381675
C	-3.390962	2.495099	-0.484670
C	-2.005872	2.512995	-0.408087
C	-1.287029	1.329662	-0.200520
C	-1.981745	0.121478	-0.069521
C	-3.370699	0.111106	-0.184736
C	2.360668	1.049302	-0.939437
C	1.802799	-1.785884	-0.943254
H	-5.162006	1.267053	-0.452758
H	-3.928499	3.423508	-0.638006
H	-1.463622	3.447718	-0.511338
C	3.330333	0.030975	-1.434950
C	3.048198	-1.292380	-1.428660
H	4.284317	0.405568	-1.788321
H	3.781539	-2.002533	-1.795089
H	0.466414	2.363913	-0.134127
H	2.179466	1.816834	-1.706882
H	1.616567	-2.852847	-0.930901
H	-3.904420	-0.829776	-0.102635
O	2.925030	1.877595	0.106258
O	3.323714	1.033707	1.184453
C	2.516715	1.362911	2.302608
H	2.925896	0.772156	3.124245

H	2.601383	2.426431	2.540749
H	1.471522	1.090026	2.137942

PTE-$OOCH_3^{\bullet}$ - Site 3

Solvent = Water
E = -975.499448
G = -975.320806
ZPC = 0.223553
TCE = 0.238687
TCH = 0.239631
TCG = 0.178642
N_{imag} = 0

Te	-1.040058	-1.605455	0.322719
N	-0.191315	1.534235	0.300554
C	0.805781	0.749131	-0.318906
C	0.653132	-0.669861	-0.430976
C	-4.350082	1.183953	-0.265651
C	-3.734858	2.433470	-0.225553
C	-2.358926	2.531820	-0.072618
C	-1.570424	1.382745	0.068799
C	-2.193135	0.129549	0.040410
C	-3.572480	0.037580	-0.142533
C	1.945649	1.350245	-0.762128
C	1.688714	-1.437293	-1.033127
H	-5.422334	1.100906	-0.394745
H	-4.325410	3.336782	-0.324029
H	-1.876065	3.503597	-0.053639
C	3.128054	0.579018	-1.231936
C	2.853118	-0.872696	-1.438228
H	3.551366	1.026975	-2.138988
H	3.634829	-1.472100	-1.890802
H	0.076514	2.511826	0.329593
H	2.044496	2.429863	-0.704656
H	1.531837	-2.502499	-1.168674
H	-4.042402	-0.939435	-0.177041
O	4.269088	0.752589	-0.329146
O	3.938976	0.180954	0.934216
C	3.874720	1.240360	1.879223
H	3.064151	1.930718	1.637010
H	3.676583	0.750886	2.833911
H	4.828563	1.770028	1.925545

Solvent = Pentyl Ethanoate
E = -975.500404
G = -975.322717
ZPC = 0.223134
TCE = 0.238399
TCH = 0.239343
TCG = 0.177686
N_{imag} = 0

Te	-1.068823	-1.599161	0.328038
N	-0.204802	1.523520	0.302480
C	0.796794	0.735892	-0.303974
C	0.633604	-0.680899	-0.427267
C	-4.363720	1.208387	-0.269996
C	-3.741493	2.452895	-0.212746
C	-2.365654	2.541140	-0.056222
C	-1.583323	1.386434	0.075136
C	-2.215119	0.137602	0.034673
C	-3.593249	0.056568	-0.156295
C	1.947918	1.328888	-0.727185
C	1.671060	-1.454587	-1.014705
H	-5.435900	1.133214	-0.405093
H	-4.326524	3.360846	-0.302832
H	-1.879719	3.511418	-0.028750
C	3.123553	0.557123	-1.220683
C	2.843610	-0.897850	-1.405437
H	3.495295	0.991212	-2.159107
H	3.630575	-1.505180	-1.838237
H	0.074799	2.494201	0.373192
H	2.053684	2.408124	-0.666429
H	1.513546	-2.520303	-1.146915
H	-4.070621	-0.916343	-0.205976
O	4.295556	0.755087	-0.385264
O	4.036748	0.177882	0.890973
C	4.041926	1.233047	1.832725
H	3.223995	1.934329	1.647141
H	3.899158	0.749441	2.801043
H	5.000239	1.759188	1.822795

PTE-$OOCH_3^{\bullet}$ - Site 4

Solvent = Water
E = -975.503044
G = -975.324452
ZPC = 0.223637
TCE = 0.238849
TCH = 0.239793
TCG = 0.178592
N_{imag} = 0

Te	-0.824063	-1.584390	0.083288
N	-0.544143	1.746726	0.076603
C	0.599971	1.111597	-0.357436
C	0.743566	-0.301243	-0.494358
C	-4.585997	0.607149	0.143820
C	-4.207643	1.947811	0.204513
C	-2.866592	2.293201	0.172770
C	-1.870750	1.307208	0.093446
C	-2.249149	-0.040376	0.050669
C	-3.604593	-0.373403	0.062937
C	1.705142	1.943087	-0.718786
C	1.909985	-0.844735	-0.932430
H	-5.632249	0.327497	0.159662
H	-4.957641	2.727167	0.268668
H	-2.565778	3.335379	0.208896
C	2.887450	1.432421	-1.132819
C	3.145327	-0.034517	-1.142712
H	3.697102	2.089814	-1.428154
H	3.678786	-0.345685	-2.047770
H	-0.455642	2.755707	0.117830
H	1.558535	3.019009	-0.684359
H	2.002203	-1.918367	-1.062486
H	-3.892825	-1.418569	0.018596
O	4.163622	-0.391947	-0.142982
O	3.657754	-0.082300	1.153968
C	4.492752	0.927984	1.702424
H	4.096369	1.106997	2.703008
H	5.524622	0.576735	1.770433
H	4.438963	1.842377	1.108358

Solvent = Pentyl Ethanoate
E = -975.503824
G = -975.325811
ZPC = 0.223252

TCE = 0.238552
TCH = 0.239496
TCG = 0.178014
N_{imag} = 0

Te	-0.799112	-1.567803	0.098347
N	-0.622285	1.733367	0.277678
C	0.544831	1.172701	-0.206836
C	0.715605	-0.219285	-0.468989
C	-4.632609	0.509470	0.005600
C	-4.294514	1.852078	0.162159
C	-2.963484	2.229419	0.235431
C	-1.939113	1.273264	0.175267
C	-2.279264	-0.078351	0.035634
C	-3.622336	-0.442212	-0.064158
C	1.638517	2.050446	-0.473821
C	1.875532	-0.698213	-0.985607
H	-5.670195	0.206039	-0.063295
H	-5.067526	2.609687	0.216044
H	-2.697011	3.276281	0.339661
C	2.820031	1.604189	-0.959917
C	3.085259	0.154743	-1.197599
H	3.621421	2.301272	-1.178157
H	3.505723	-0.007571	-2.199129
H	-0.560344	2.732867	0.428814
H	1.491721	3.113988	-0.303346
H	1.989929	-1.756662	-1.197213
H	-3.880105	-1.489146	-0.187655
O	4.200207	-0.325699	-0.395924
O	3.832275	-0.248423	0.978771
C	4.730632	0.653859	1.593689
H	4.458169	0.649518	2.650977
H	5.763495	0.313525	1.480277
H	4.620146	1.662711	1.187328

PTE-OOCH$_3^•$ - Site 5
Solvent = Water
E = -975.500366
G = -975.321736
ZPC = 0.223295
TCE = 0.238464
TCH = 0.239408
TCG = 0.178630
N_{imag} = 0

Te	-0.309820	-1.448119	0.023243
N	-0.876918	1.620398	0.880018
C	0.343713	1.495764	0.177387
C	0.792652	0.298756	-0.306293
C	-4.479233	-0.159859	-0.388194
C	-4.455306	1.155387	0.069350
C	-3.257190	1.739067	0.459303
C	-2.064042	1.007883	0.426816
C	-2.092969	-0.319898	-0.013656
C	-3.293698	-0.886450	-0.439049
C	1.134640	2.666966	0.028641
C	2.096205	0.170021	-1.030895
H	-5.409283	-0.617151	-0.703383
H	-5.369420	1.735847	0.112439
H	-3.233213	2.769321	0.799315
C	2.398158	2.604269	-0.602822
C	2.879679	1.439914	-1.110780
H	2.982422	3.513620	-0.686320
H	3.842484	1.391923	-1.606294
H	-1.041322	2.579640	1.163730
H	0.763816	3.606971	0.420395
H	1.929123	-0.230980	-2.041702
H	-3.303731	-1.910410	-0.796476
O	2.883503	-0.909520	-0.468421
O	3.190525	-0.583515	0.887426
C	4.604690	-0.476953	0.983070
H	4.797111	-0.272907	2.037218
H	5.079417	-1.416917	0.694538
H	4.975646	0.345535	0.368450

Solvent = Pentyl Ethanoate
E = -975.502109
G = -975.324151
ZPC = 0.222842
TCE = 0.238114
TCH = 0.239058
TCG = 0.177958
N_{imag} = 0

Te	-0.270418	-1.418771	0.020136
N	-0.920239	1.641595	0.843854
C	0.314962	1.538126	0.170459
C	0.791356	0.347731	-0.306176
C	-4.475918	-0.250795	-0.384247
C	-4.489799	1.061950	0.078507
C	-3.307279	1.682458	0.458154
C	-2.090717	0.991177	0.412306
C	-2.082682	-0.338339	-0.026657
C	-3.268313	-0.939002	-0.445376
C	1.098796	2.714438	0.044923
C	2.102702	0.236392	-1.020810
H	-5.393536	-0.736023	-0.694470
H	-5.421785	1.612661	0.132628
H	-3.315695	2.712797	0.799990
C	2.388869	2.660288	-0.534337
C	2.895500	1.503904	-1.033383
H	2.974618	3.571699	-0.581130
H	3.878373	1.465944	-1.489374
H	-1.102055	2.583225	1.167772
H	0.710887	3.652676	0.424514
H	1.931385	-0.100076	-2.056139
H	-3.249713	-1.961797	-0.806819
O	2.860777	-0.882991	-0.520025
O	3.152746	-0.640493	0.856712
C	4.562629	-0.621922	0.977934
H	4.751038	-0.487604	2.044679
H	4.994004	-1.569311	0.644903
H	4.997092	0.210154	0.418136

PTE-OOCH$_3^•$ - Site 5a
Solvent = Water
E = -975.542175
G = -975.363219
ZPC = 0.223782
TCE = 0.239370
TCH = 0.240315
TCG = 0.178957
N_{imag} = 0

Te	0.027621	-2.022351	0.272429
N	0.126032	0.799729	-1.563722

C	1.352756	0.779268	-0.898051
C	1.515550	1.006490	0.481449
C	-3.746134	0.113408	-0.076553
C	-3.489417	1.166306	-0.951972
C	-2.197785	1.392871	-1.405490
C	-1.141909	0.556459	-1.022422
C	-1.405195	-0.534899	-0.174949
C	-2.703222	-0.720022	0.313851
C	2.513773	0.549964	-1.651581
C	2.769531	0.934597	1.081739
H	-4.747106	-0.059830	0.299915
H	-4.290209	1.825629	-1.265977
H	-1.983704	2.229581	-2.062490
C	3.762777	0.494850	-1.055150
C	3.898040	0.661309	0.323929
H	4.635551	0.302988	-1.668103
H	4.870457	0.600331	0.795960
H	0.132002	1.293353	-2.447218
H	2.406256	0.387053	-2.718306
H	2.831972	1.108225	2.149800
H	-2.902698	-1.544386	0.989856
O	0.442399	1.277134	1.306919
O	-0.102455	2.562785	0.909424
C	-1.317410	2.682117	1.632041
H	-1.991012	1.855969	1.394973
H	-1.119911	2.724140	2.705663
H	-1.747794	3.625440	1.293888

Solvent = Pentyl Ethanoate
E = -975.545602
G = -975.366610
ZPC = 0.223799
TCE = 0.239372
TCH = 0.240316
TCG = 0.178992
N_{imag} = 0

Te	0.051743	-2.010125	0.269564
N	0.111877	0.820221	-1.549357
C	1.343099	0.796655	-0.891896
C	1.509071	1.027734	0.487643
C	-3.751513	0.068945	-0.073414
C	-3.509375	1.127937	-0.945384
C	-2.220587	1.376287	-1.394009
C	-1.152241	0.553363	-1.015151
C	-1.400368	-0.542338	-0.168948
C	-2.695951	-0.747280	0.317752
C	2.497842	0.556173	-1.650304
C	2.767628	0.950378	1.078199
H	-4.750516	-0.120988	0.300685
H	-4.319878	1.776116	-1.258545
H	-2.017938	2.223020	-2.042011
C	3.750217	0.497953	-1.061221
C	3.891420	0.669743	0.315659
H	4.619024	0.297075	-1.677228
H	4.865876	0.606661	0.783787
H	0.113599	1.301485	-2.438119
H	2.382884	0.379539	-2.714339
H	2.837150	1.127674	2.145226
H	-2.882495	-1.573171	0.995801
O	0.446782	1.293124	1.315058
O	-0.136754	2.550423	0.887577
C	-1.340303	2.651140	1.622700
H	-1.991082	1.796048	1.425814
H	-1.133363	2.735281	2.693254
H	-1.810890	3.567711	1.263002

TS[PTE-OOCH$_3^•$] - Site 2a
Solvent = Water
E = -975.489497
G = -975.312149
ZPC = 0.221398
TCE = 0.236548
TCH = 0.237493
TCG = 0.177348
N_{imag} = 1, -481.82 cm^{-1}

Te	-0.418073	-1.742181	-0.140198
N	-0.202260	1.367681	-1.219721
C	1.023929	0.909149	-0.729035
C	1.221527	-0.488879	-0.415839
C	-3.983203	0.676560	0.503075
C	-3.665255	1.908914	-0.063607
C	-2.406806	2.123265	-0.607090
C	-1.446418	1.105055	-0.611448
C	-1.772287	-0.137412	-0.059002
C	-3.032152	-0.336911	0.507105
C	2.182387	1.642851	-1.193517
C	2.508773	-0.987368	-0.289653
H	-4.959870	0.505034	0.938882
H	-4.393785	2.711000	-0.074038
H	-2.150490	3.086935	-1.035858
C	3.434960	1.116623	-1.080115
C	3.615411	-0.199158	-0.596144
H	4.295991	1.703493	-1.376581
H	4.613009	-0.609450	-0.500140
H	-0.139433	2.322988	-1.554508
H	2.023014	2.648333	-1.568608
H	2.654736	-2.017852	0.016622
H	-3.272179	-1.299570	0.946249
O	0.874866	1.639416	0.995995
O	2.006564	1.441365	1.782758
C	1.722644	0.450179	2.762340
H	2.639277	0.341879	3.343828
H	0.907937	0.782818	3.410224
H	1.464896	-0.499445	2.289795

Solvent = Pentyl Ethanoate
E = -975.486788
G = -975.309290
ZPC = 0.221463
TCE = 0.236575
TCH = 0.237519
TCG = 0.177498
N_{imag} = 1, -615.29 cm^{-1}

Te	-0.438860	-1.750598	-0.123216
N	-0.195116	1.311191	-1.255251
C	1.035307	0.873234	-0.734053
C	1.222838	-0.514828	-0.373719
C	-3.970093	0.722332	0.519269
C	-3.640461	1.935218	-0.079226
C	-2.383046	2.120252	-0.636127
C	-1.436246	1.090425	-0.628560
C	-1.777039	-0.133495	-0.043842
C	-3.031150	-0.302744	0.540416

C	2.200549	1.567677	-1.240655
C	2.502164	-1.008896	-0.194556
H	-4.944920	0.574860	0.968152
H	-4.358039	2.747175	-0.101678
H	-2.117715	3.073270	-1.082846
C	3.450144	1.045871	-1.077283
C	3.619004	-0.236346	-0.514302
H	4.317475	1.614432	-1.391449
H	4.613545	-0.641417	-0.372452
H	-0.126751	2.246650	-1.637850
H	2.052797	2.553897	-1.667946
H	2.642057	-2.024244	0.162603
H	-3.279870	-1.250124	1.007689
O	0.901411	1.681040	0.905865
O	2.025023	1.497812	1.687179
C	1.711892	0.583657	2.726088
H	2.638511	0.443773	3.285754
H	0.941663	1.000571	3.380859
H	1.373160	-0.371034	2.318080

TS[PTE-OOCH$_3$$^•$] - Site 2

Solvent = Water
E = -975.492206
G = -975.314715
ZPC = 0.222088
TCE = 0.237246
TCH = 0.238190
TCG = 0.177492
N_{imag} = 1, -501.40 cm^{-1}

Te	-0.804175	-1.836427	0.071191
N	0.044329	0.981657	-1.381431
C	1.141792	0.300825	-0.893735
C	1.065629	-0.901677	-0.180497
C	-3.733942	1.390343	0.421735
C	-3.172812	2.429895	-0.316388
C	-1.920477	2.278075	-0.893622
C	-1.218244	1.074005	-0.762224
C	-1.787979	0.022329	-0.037397
C	-3.033655	0.197374	0.565336
C	2.424156	0.926028	-1.086507
C	2.228792	-1.509190	0.269845
H	-4.707115	1.505705	0.883231
H	-3.704259	3.366563	-0.435176
H	-1.469685	3.092202	-1.451803
C	3.599162	0.176883	-0.778387
C	3.499187	-0.982513	-0.057574
H	4.562400	0.580619	-1.064583
H	4.388445	-1.523677	0.243382
H	0.295299	1.828686	-1.880998
H	2.483859	1.686844	-1.859871
H	2.166108	-2.429168	0.840147
H	-3.465160	-0.614694	1.140392
O	2.376511	2.283310	0.199543
O	2.363997	1.744257	1.479787
C	1.048915	1.827696	2.016396
H	1.139590	1.507144	3.055027
H	0.689346	2.857969	1.974124
H	0.360760	1.163418	1.487671

Solvent = Pentyl Ethanoate
E = -975.491200
G = -975.313915
ZPC = 0.222009
TCE = 0.237172
TCH = 0.238116
TCG = 0.177285
N_{imag} = 1, -600.12 cm^{-1}

Te	-0.842018	-1.834553	0.068047
N	0.054609	0.948239	-1.384181
C	1.146592	0.255645	-0.884830
C	1.046073	-0.933857	-0.160559
C	-3.700153	1.456509	0.443092
C	-3.120515	2.479026	-0.303453
C	-1.875642	2.296498	-0.887442
C	-1.199738	1.077100	-0.759722
C	-1.790461	0.042529	-0.026150
C	-3.025656	0.248977	0.586724
C	2.439601	0.862842	-1.071524
C	2.193656	-1.552546	0.320761
H	-4.666791	1.596597	0.911737
H	-3.630808	3.427734	-0.421484
H	-1.409512	3.100756	-1.447959
C	3.597406	0.098334	-0.733692
C	3.472233	-1.046858	0.006502
H	4.571037	0.485652	-1.007409
H	4.351461	-1.589577	0.333709
H	0.315302	1.778399	-1.903346
H	2.524064	1.582798	-1.881805
H	2.108204	-2.461250	0.906312
H	-3.470424	-0.549276	1.171460
O	2.413353	2.265347	0.116568
O	2.418538	1.820404	1.420682
C	1.102431	1.884161	1.949268
H	1.209231	1.694098	3.018693
H	0.674998	2.876990	1.789301
H	0.455811	1.119970	1.509758

TS[PTE-OOCH$_3$$^•$] - Site 3

Solvent = Water
E = -975.486532
G = -975.309103
ZPC = 0.221953
TCE = 0.237018
TCH = 0.237962
TCG = 0.177428
N_{imag} = 1, -582.34 cm^{-1}

Te	-0.864657	-1.613895	0.292655
N	-0.196866	1.565145	0.231261
C	0.812102	0.831123	-0.418478
C	0.746952	-0.579889	-0.533471
C	-4.342765	0.984306	-0.223843
C	-3.799052	2.266543	-0.190628
C	-2.427490	2.442265	-0.069676
C	-1.574268	1.338559	0.047892
C	-2.123948	0.052297	0.031911
C	-3.499467	-0.117027	-0.123428
C	1.918228	1.497252	-0.906699
C	1.780427	-1.279469	-1.190503
H	-5.411295	0.841059	-0.328776
H	-4.442172	3.135079	-0.270463
H	-1.999759	3.439571	-0.060080
C	3.058157	0.775535	-1.366718
C	2.900013	-0.626190	-1.633789

H	3.798796	1.322387	-1.939643
H	3.699593	-1.166513	-2.125273
H	0.022035	2.554974	0.260499
H	1.963458	2.579493	-0.845483
H	1.679566	-2.348860	-1.340022
H	-3.913830	-1.118975	-0.151427
O	4.211984	0.722029	0.091288
O	3.543149	0.149537	1.159851
C	3.167641	1.169974	2.078107
H	2.538286	1.916070	1.588884
H	2.607708	0.662893	2.864762
H	4.057584	1.645144	2.496939

Solvent = Pentyl Ethanoate
E = -975.486180
G = -975.308914
ZPC = 0.221866
TCE = 0.236957
TCH = 0.237901
TCG = 0.177266
N_{imag} = 1, -649.71 cm^{-1}

Te	-0.829239	-1.609755	0.265891
N	-0.225638	1.566458	0.263389
C	0.800383	0.868174	-0.401903
C	0.755302	-0.534883	-0.565988
C	-4.360429	0.928561	-0.213598
C	-3.840072	2.218155	-0.146399
C	-2.472110	2.414959	-0.018823
C	-1.598640	1.324990	0.075475
C	-2.126631	0.029796	0.027033
C	-3.497362	-0.159378	-0.139131
C	1.903956	1.564353	-0.854340
C	1.790738	-1.195355	-1.256575
H	-5.426032	0.769143	-0.325954
H	-4.498558	3.076914	-0.206988
H	-2.065393	3.420732	0.011777
C	3.060039	0.870381	-1.323416
C	2.906529	-0.515339	-1.666855
H	3.795717	1.448459	-1.872423
H	3.713027	-1.028281	-2.176085
H	-0.019545	2.555127	0.341952
H	1.939069	2.644292	-0.752215
H	1.699050	-2.258125	-1.453822
H	-3.894492	-1.167238	-0.197111
O	4.177012	0.775508	0.118697
O	3.546533	0.079656	1.125000
C	3.120016	0.990698	2.125781
H	2.428339	1.729220	1.713618
H	2.609783	0.384755	2.876246
H	3.980272	1.495083	2.573834

TS[PTE-OOCH$_3^•$] - Site 4
Solvent = Water
E = -975.490542
G = -975.313061
ZPC = 0.222212
TCE = 0.237366
TCH = 0.238310
TCG = 0.177480
N_{imag} = 1, -443.60 cm^{-1}

Te	-0.799520	-1.599980	0.109317
N	-0.433754	1.720808	-0.077021
C	0.674189	1.038110	-0.510574
C	0.766078	-0.378121	-0.580677
C	-4.498417	0.694503	0.200181
C	-4.084886	2.024795	0.170309
C	-2.736792	2.330644	0.070782
C	-1.773168	1.314147	0.013137
C	-2.185702	-0.021927	0.058168
C	-3.547111	-0.317000	0.138626
C	1.786724	1.813370	-0.929155
C	1.922587	-0.966363	-1.028006
H	-5.550147	0.445382	0.270887
H	-4.811569	2.826978	0.217277
H	-2.408206	3.364413	0.036217
C	2.945679	1.222747	-1.348370
C	3.096967	-0.195882	-1.285544
H	3.777270	1.832489	-1.680448
H	3.900855	-0.658574	-1.845714
H	-0.322169	2.728582	-0.090531
H	1.685643	2.894073	-0.932804
H	1.984771	-2.046575	-1.105302
H	-3.863480	-1.354454	0.163412
O	4.099417	-0.448823	0.319156
O	3.361353	0.096563	1.368584
C	3.989484	1.298127	1.793996
H	3.351043	1.704208	2.580054
H	4.983591	1.084185	2.194752
H	4.063748	2.007746	0.967689

Solvent = Pentyl Ethanoate
E = -975.488621
G = -975.311735
ZPC = 0.221771
TCE = 0.236927
TCH = 0.237871
TCG = 0.176886
N_{imag} = 1, -596.11 cm^{-1}

Te	-0.759880	-1.588077	0.136771
N	-0.459594	1.679645	0.251779
C	0.645923	1.088332	-0.335212
C	0.750217	-0.305111	-0.566687
C	-4.522210	0.626301	-0.006432
C	-4.128946	1.956737	0.112030
C	-2.782019	2.281454	0.176266
C	-1.802791	1.280690	0.150725
C	-2.197989	-0.057557	0.045652
C	-3.553281	-0.369894	-0.050298
C	1.712554	1.926029	-0.732823
C	1.875610	-0.822503	-1.157592
H	-5.571776	0.365208	-0.067983
H	-4.870230	2.746674	0.141843
H	-2.471342	3.319370	0.245358
C	2.851780	1.406894	-1.289521
C	3.026935	-0.007077	-1.393282
H	3.651324	2.065394	-1.607844
H	3.785978	-0.382332	-2.070377
H	-0.353868	2.677641	0.387811
H	1.596818	2.999534	-0.617836
H	1.945168	-1.885905	-1.359508
H	-3.854016	-1.407789	-0.149288
O	4.114434	-0.434382	0.055267
O	3.448696	-0.081532	1.212852

C	4.075020	1.062891	1.766308
H	3.493680	1.320138	2.653802
H	5.105783	0.834323	2.051761
H	4.063524	1.894433	1.057933

TS[PTE-OOCH$_3^•$] - Site 5

Solvent = Water
E = -975.485241
G = -975.307804
ZPC = 0.221948
TCE = 0.237042
TCH = 0.237986
TCG = 0.177437
N_{imag} = 1, -601.39 cm^{-1}

Te	0.485097	-1.523064	0.011357
N	0.627863	1.622134	-0.742807
C	-0.520583	1.328641	0.020526
C	-0.789667	0.061450	0.501555
C	4.494992	0.230504	0.187182
C	4.285877	1.549339	-0.208134
C	3.002632	2.002596	-0.484714
C	1.907343	1.135556	-0.400820
C	2.121765	-0.194270	-0.022643
C	3.407811	-0.631890	0.289912
C	-1.441934	2.369064	0.265597
C	-2.030107	-0.210726	1.168218
H	5.492580	-0.125111	0.414683
H	5.121626	2.234295	-0.290365
H	2.835697	3.035189	-0.775056
C	-2.599581	2.144384	1.018485
C	-2.874293	0.893112	1.519228
H	-3.264405	2.975191	1.223045
H	-3.755169	0.707619	2.121228
H	0.662696	2.601301	-1.003886
H	-1.229752	3.359904	-0.122038
H	-2.081914	-1.100730	1.787278
H	3.561153	-1.659770	0.600079
O	-2.999091	-1.077746	-0.138317
O	-3.153983	-0.237555	-1.225284
C	-4.504716	0.212489	-1.274930
H	-4.760011	0.756366	-0.363763
H	-4.557172	0.877030	-2.137824
H	-5.178069	-0.635985	-1.412258

Solvent = Pentyl Ethanoate
E = -975.485480
G = -975.308084
ZPC = 0.221847
TCE = 0.236971
TCH = 0.237915
TCG = 0.177396
N_{imag} = 1, -657.02 cm^{-1}

Te	0.436705	-1.497034	0.024713
N	0.671680	1.631868	-0.736355
C	-0.496725	1.372863	0.004389
C	-0.790218	0.121749	0.511624
C	4.499094	0.139330	0.190649
C	4.328012	1.456671	-0.224612
C	3.057705	1.943741	-0.502034
C	1.936184	1.112330	-0.401485
C	2.113329	-0.219281	-0.006662
C	3.386625	-0.687874	0.309231
C	-1.410137	2.428278	0.205385
C	-2.037013	-0.123488	1.177422
H	5.486469	-0.242447	0.420870
H	5.183588	2.115134	-0.320419
H	2.923045	2.977491	-0.805413
C	-2.584947	2.231663	0.938346
C	-2.878591	0.998661	1.473492
H	-3.247293	3.072961	1.107558
H	-3.767971	0.842233	2.071711
H	0.723595	2.594111	-1.046925
H	-1.180749	3.407593	-0.202120
H	-2.082109	-0.972330	1.853492
H	3.511272	-1.714908	0.635704
O	-2.963328	-1.088977	-0.056275
O	-3.111516	-0.350714	-1.207397
C	-4.456518	0.095081	-1.296419
H	-4.710284	0.732175	-0.446252
H	-4.514086	0.667990	-2.223200
H	-5.137938	-0.758118	-1.342163

TS[PTE-OOCH$_3^•$] - Site 5a

Solvent = Water
E = -975.491700
G = -975.314681
ZPC = 0.221550
TCE = 0.236866
TCH = 0.237810
TCG = 0.177019
N_{imag} = 1, -428.53 cm^{-1}

Te	-0.396862	-1.782716	0.019498
N	-0.109842	1.244319	-1.280051
C	1.104623	0.701889	-0.946238
C	1.229321	-0.431455	-0.073508
C	-3.968597	0.710329	0.289704
C	-3.594177	1.904392	-0.324275
C	-2.306925	2.061599	-0.812760
C	-1.370747	1.022808	-0.711586
C	-1.748072	-0.179454	-0.100304
C	-3.041301	-0.318460	0.404523
C	2.266735	1.325498	-1.435156
C	2.533306	-0.996653	0.103221
H	-4.970888	0.582114	0.679859
H	-4.303025	2.718466	-0.417350
H	-2.007259	2.991668	-1.284600
C	3.513115	0.810331	-1.153646
C	3.646847	-0.371078	-0.385435
H	4.395987	1.306424	-1.538107
H	4.629977	-0.789540	-0.206676
H	-0.043850	2.116083	-1.795023
H	2.160612	2.216435	-2.045431
H	2.620050	-1.912448	0.677496
H	-3.323658	-1.246938	0.889038
O	0.893438	0.370087	1.641196
O	1.841651	1.360631	1.906534
C	1.242012	2.637647	1.731535
H	0.918054	2.781947	0.698569
H	0.394312	2.753294	2.411135
H	2.017830	3.363397	1.979260

Solvent = Pentyl Ethanoate
E = -975.489063

G = -975.312054
ZPC = 0.221346
TCE = 0.236651
TCH = 0.237595
TCG = 0.177009
N_{imag} = 1, -574.73 cm^{-1}

Te	-0.366413	-1.778194	0.005998
N	-0.120404	1.232936	-1.290670
C	1.108812	0.713570	-0.945930
C	1.246510	-0.399637	-0.052290
C	-3.971141	0.672308	0.294443
C	-3.613825	1.862930	-0.333027
C	-2.329012	2.030920	-0.826227
C	-1.379829	1.004987	-0.722819
C	-1.743294	-0.196512	-0.101106
C	-3.029902	-0.344041	0.414113
C	2.259832	1.340240	-1.444467
C	2.556313	-0.941798	0.136547
H	-4.969472	0.536709	0.692539
H	-4.332121	2.668597	-0.430022
H	-2.043621	2.963986	-1.301718
C	3.517167	0.845322	-1.155530
C	3.664876	-0.313257	-0.364424
H	4.392431	1.345143	-1.553024
H	4.652644	-0.713845	-0.170105
H	-0.069592	2.077031	-1.847896
H	2.146254	2.216292	-2.075474
H	2.656052	-1.838085	0.738883
H	-3.299154	-1.268871	0.913260
O	0.863880	0.356618	1.641083
O	1.765943	1.352600	1.958076
C	1.157172	2.615410	1.744356
H	0.948868	2.780914	0.684385
H	0.233362	2.696485	2.322868
H	1.879980	3.354777	2.094026

Single Electron Transfer

PS•+
Solvent = Water
E = -915.381844
$E_{VER.EX.}$ = -915.363183
G = -915.238493
ZPC = 0.180074
TCE = 0.190297
TCH = 0.191241
TCG = 0.143351
N_{imag} = 0

S	-1.712738	-0.000084	0.000000
N	1.371449	-0.000082	-0.000000
C	0.767746	-0.000028	-1.226263
C	-0.636163	-0.000004	-1.362447
C	-0.402697	0.000074	3.758109
C	0.998130	0.000046	3.623339
C	1.578089	-0.000004	2.378979
C	0.767746	-0.000028	1.226263
C	-0.636163	-0.000004	1.362447
C	-1.210583	0.000048	2.642276
C	1.578089	-0.000004	-2.378979
C	-1.210583	0.000048	-2.642276
H	-0.850958	0.000115	4.743478
H	1.624612	0.000065	4.506361
H	2.655247	-0.000021	2.255935
C	0.998130	0.000046	-3.623339
C	-0.402697	0.000074	-3.758109
H	1.624612	0.000065	-4.506361
H	-0.850958	0.000115	-4.743478
H	2.390859	-0.000119	-0.000000
H	2.655247	-0.000021	-2.255935
H	-2.289622	0.000067	-2.744482
H	-2.289622	0.000067	2.744482

Solvent = Pentyl Ethanoate
E = -915.376322
$E_{VER.EX.}$ = -915.358854
G = -915.232580
ZPC = 0.180181
TCE = 0.190324
TCH = 0.191268
TCG = 0.143742
N_{imag} = 0

S	-1.710695	-0.000020	0.000000
N	1.371405	-0.000031	-0.000000
C	0.768749	-0.000007	-1.227643
C	-0.635695	-0.000001	-1.362992
C	-0.404023	0.000017	3.758957
C	0.996834	0.000014	3.625043
C	1.577589	0.000002	2.381077
C	0.768749	-0.000007	1.227643
C	-0.635695	-0.000001	1.362992
C	-1.210846	0.000011	2.643016
C	1.577589	0.000002	-2.381077
C	-1.210846	0.000011	-2.643016
H	-0.852548	0.000025	4.744348
H	1.622460	0.000023	4.508988
H	2.655682	0.000004	2.263089
C	0.996834	0.000014	-3.625043
C	-0.404023	0.000017	-3.758957
H	1.622460	0.000023	-4.508988
H	-0.852548	0.000025	-4.744348
H	2.388808	-0.000048	-0.000000
H	2.655682	0.000004	-2.263089
H	-2.289998	0.000017	-2.744022
H	-2.289998	0.000017	2.744022

PSE•+
Solvent = Water
E = -2918.820979
$E_{VER.EX.}$ = -2918.802289
G = -2918.679369
ZPC = 0.179361
TCE = 0.189901
TCH = 0.190845
TCG = 0.141609
N_{imag} = 0

Se	1.579302	0.000025	0.000000
N	-1.611885	0.000048	-0.000000
C	-1.025468	0.000015	1.237848
C	0.371705	-0.000003	1.424487
C	0.059668	-0.000044	-3.816542
C	-1.335166	-0.000025	-3.631963
C	-1.871153	0.000004	-2.367669

C	-1.025468	0.000015	-1.237848
C	0.371705	-0.000003	-1.424487
C	0.899872	-0.000033	-2.725540
C	-1.871153	0.000004	2.367669
C	0.899872	-0.000033	2.725540
H	0.474558	-0.000065	-4.816556
H	-1.993656	-0.000034	-4.491694
H	-2.944720	0.000017	-2.214580
C	-1.335166	-0.000025	3.631963
C	0.059668	-0.000044	3.816542
H	-1.993656	-0.000034	4.491694
H	0.474558	-0.000065	4.816556
H	-2.630064	0.000114	-0.000000
H	-2.944720	0.000017	2.214580
H	1.975566	-0.000049	2.857509
H	1.975566	-0.000049	-2.857509

Solvent = Pentyl Ethanoate
E = -2918.812597
$E_{VER.EX.}$ = -2918.795276
G = -2918.671311
ZPC = 0.179457
TCE = 0.190016
TCH = 0.190960
TCG = 0.141286
N_{imag} = 0

Se	1.579535	0.000034	-0.000000
N	-1.608012	0.000047	0.000000
C	-1.024149	0.000017	1.239435
C	0.373402	-0.000004	1.427396
C	0.057272	-0.000057	-3.818461
C	-1.337110	-0.000026	-3.632871
C	-1.871035	0.000010	-2.368183
C	-1.024149	0.000017	-1.239435
C	0.373402	-0.000004	-1.427396
C	0.899138	-0.000047	-2.728951
C	-1.871035	0.000010	2.368183
C	0.899138	-0.000047	2.728951
H	0.471043	-0.000087	-4.819074
H	-1.996522	-0.000033	-4.492152
H	-2.945148	0.000028	-2.216322
C	-1.337110	-0.000026	3.632871
C	0.057272	-0.000057	3.818461
H	-1.996522	-0.000033	4.492152
H	0.471043	-0.000087	4.819074
H	-2.624876	0.000112	0.000000
H	-2.945148	0.000028	2.216322
H	1.973912	-0.000072	2.867567
H	1.973912	-0.000072	-2.867567

PTE$^{\bullet+}$
Solvent = Water
E = -785.139070
$E_{VER.EX.}$ = -785.129320
G = -784.999225
ZPC = 0.178291
TCE = 0.189121
TCH = 0.190065
TCG = 0.139846
N_{imag} = 0

Te	1.516664	0.000049	-0.000000
N	-1.836235	0.000185	0.000000
C	-1.265882	0.000072	1.255069
C	0.116916	0.000005	1.508172
C	-0.276838	-0.000154	-3.895341
C	-1.657654	-0.000087	-3.647774
C	-2.143981	0.000019	-2.359304
C	-1.265882	0.000072	-1.255069
C	0.116916	0.000005	-1.508172
C	0.597221	-0.000119	-2.829562
C	-2.143981	0.000019	2.359304
C	0.597221	-0.000119	2.829562
H	0.099581	-0.000243	-4.910241
H	-2.355128	-0.000120	-4.476914
H	-3.213033	0.000062	-2.174806
C	-1.657654	-0.000087	3.647774
C	-0.276838	-0.000154	3.895341
H	-2.355128	-0.000120	4.476914
H	0.099581	-0.000243	4.910241
H	-2.850646	0.000292	0.000000
H	-3.213033	0.000062	2.174806
H	1.668778	-0.000179	3.000401
H	1.668778	-0.000179	-3.000401

Solvent = Pentyl Ethanoate
E = -785.128353
$E_{VER.EX.}$ = -785.117022
G = -784.987815
ZPC = 0.178801
TCE = 0.189570
TCH = 0.190515
TCG = 0.140538
N_{imag} = 0

Te	1.511919	0.000055	0.000000
N	-1.829951	0.000139	-0.000000
C	-1.263600	0.000056	1.256036
C	0.119909	-0.000015	1.509924
C	-0.274205	-0.000149	-3.895391
C	-1.655686	-0.000066	-3.647580
C	-2.142052	0.000032	-2.360363
C	-1.263600	0.000056	-1.256036
C	0.119909	-0.000015	-1.509924
C	0.600396	-0.000128	-2.832032
C	-2.142052	0.000032	2.360363
C	0.600396	-0.000128	2.832032
H	0.101062	-0.000244	-4.910746
H	-2.352853	-0.000072	-4.477034
H	-3.211735	0.000090	-2.177781
C	-1.655686	-0.000066	3.647580
C	-0.274205	-0.000149	3.895391
H	-2.352853	-0.000072	4.477034
H	0.101062	-0.000244	4.910746
H	-2.843966	0.000274	-0.000000
H	-3.211735	0.000090	2.177781
H	1.671872	-0.000192	3.005349
H	1.671872	-0.000192	-3.005349

HO^-
Solvent = Water
E = -75.939618
$E_{VER.EX.}$ = -75.939444
G = -75.947023
ZPC = 0.008840

TCE = 0.011200
TCH = 0.012144
TCG = -0.007406
N_{imag} = 0

O	-0.000000	0.000000	0.106787
H	0.000000	0.000000	-0.854293

Solvent = Pentyl Ethanoate
E = -75.877637
$E_{VER.EX.}$ = -75.877475
G = -75.885101
ZPC = 0.008782
TCE = 0.011142
TCH = 0.012087
TCG = -0.007464
N_{imag} = 0

O	-0.000000	0.000000	0.106844
H	0.000000	0.000000	-0.854751

HOO^-
Solvent = Water
E = -151.070397
$E_{VER.EX.}$ = -151.052652
G = -151.078318
ZPC = 0.013783
TCE = 0.016675
TCH = 0.017619
TCG = -0.007921
N_{imag} = 0

O	0.055126	0.788637	0.000000
O	0.055126	-0.676069	-0.000000
H	-0.882008	-0.900539	0.000000

Solvent = Pentyl Ethanoate
E = -151.014558
$E_{VER.EX.}$ = -150.997101
G = -151.022624
ZPC = 0.013652
TCE = 0.016551
TCH = 0.017496
TCG = -0.008066
N_{imag} = 0

O	0.055302	0.790971	-0.000000
O	0.055302	-0.681407	0.000000
H	-0.884830	-0.876517	0.000000

$CH3OO^-$
Solvent = Water
E = -190.360430
$E_{VER.EX.}$ = -190.342665
G = -190.341842
ZPC = 0.043327
TCE = 0.046963
TCH = 0.047907
TCG = 0.018588
N_{imag} = 0

C	-1.070393	-0.214773	0.000002
H	-1.934059	0.455608	-0.000255
H	-1.102316	-0.849507	-0.893072
H	-1.102575	-0.849191	0.893292
O	0.080881	0.594400	0.000004
O	1.239283	-0.277934	-0.000001

Solvent = Pentyl Ethanoate
E = -190.309094
$E_{VER.EX.}$ = -190.291383
G = -190.291392
ZPC = 0.042410
TCE = 0.046046
TCH = 0.046991
TCG = 0.017703
N_{imag} = 0

C	-1.047567	-0.213877	-0.000007
H	-1.939385	0.427489	-0.000522
H	-1.077859	-0.864786	-0.889222
H	-1.078498	-0.864160	0.889685
O	0.069935	0.599494	0.000012
O	1.227708	-0.276404	0.000000

DIRECT OXIDATION

$CH_3O^•$
Solvent = Water
E = -115.035165
G = -115.021858
ZPC = 0.036306
TCE = 0.039432
TCH = 0.040377
TCG = 0.013308
N_{imag} = 0

C	0.586134	-0.000385	-0.013885
H	0.997557	0.905517	-0.469167
H	0.872641	0.013629	1.054990
H	0.998271	-0.916054	-0.448034
O	-0.798159	-0.000098	-0.006810

Solvent = Pentyl Ethanoate
E = -115.034981
G = -115.021732
ZPC = 0.036258
TCE = 0.039429
TCH = 0.040373
TCG = 0.013249
N_{imag} = 0

C	0.575188	-0.000315	-0.013430
H	0.998425	0.904009	-0.467494
H	0.873530	0.011305	1.052580
H	0.999006	-0.912787	-0.449930
O	-0.790261	-0.000080	-0.006822

PS=O
Solvent = Water
E = -990.749365
G = -990.603189
ZPC = 0.183541
TCE = 0.194742
TCH = 0.195686
TCG = 0.146177

N_{imag} = 0

S	1.605208	0.058569	0.000000
N	-1.429638	0.551454	-0.000000
C	-0.880657	0.198255	-1.216448
C	0.462843	-0.179201	-1.336796
C	0.179483	-0.622397	3.688088
C	-1.156686	-0.218335	3.579293
C	-1.685245	0.182418	2.367082
C	-0.880657	0.198255	1.216448
C	0.462843	-0.179201	1.336796
C	0.986170	-0.593869	2.565414
C	-1.685245	0.182418	-2.367082
C	0.986170	-0.593869	-2.565414
H	0.580486	-0.943474	4.640774
H	-1.795390	-0.229432	4.454680
H	-2.726650	0.473161	2.282141
C	-1.156686	-0.218335	-3.579293
C	0.179483	-0.622397	-3.688088
H	-1.795390	-0.229432	-4.454680
H	0.580486	-0.943474	-4.640774
H	-2.424735	0.744849	-0.000000
H	-2.726650	0.473161	-2.282141
H	2.030738	-0.880083	-2.628200
H	2.030738	-0.880083	2.628200
O	1.962453	1.551885	0.000000

Solvent = Pentyl Ethanoate
E = -990.745293
G = -990.599314
ZPC = 0.183394
TCE = 0.194635
TCH = 0.195579
TCG = 0.145980
N_{imag} = 0

S	1.617288	0.072722	0.000000
N	-1.427376	0.550828	-0.000000
C	-0.880241	0.199565	-1.218143
C	0.461117	-0.186191	-1.334254
C	0.171371	-0.631036	3.687187
C	-1.160308	-0.216861	3.580864
C	-1.685157	0.188542	2.367952
C	-0.880241	0.199565	1.218143
C	0.461117	-0.186191	1.334254
C	0.977523	-0.603861	2.562682
C	-1.685157	0.188542	-2.367952
C	0.977523	-0.603861	-2.562682
H	0.571486	-0.955049	4.639521
H	-1.798576	-0.222352	4.456959
H	-2.724412	0.489176	2.286456
C	-1.160308	-0.216861	-3.580864
C	0.171371	-0.631036	-3.687187
H	-1.798576	-0.222352	-4.456959
H	0.571486	-0.955049	-4.639521
H	-2.413416	0.778302	-0.000000
H	-2.724412	0.489176	-2.286456
H	2.021593	-0.893083	-2.625608
H	2.021593	-0.893083	2.625608
O	1.972075	1.545384	0.000000

PSE=O
Solvent = Water
E = -2994.189907
G = -2994.046818
ZPC = 0.181684
TCE = 0.193486
TCH = 0.194430
TCG = 0.143089
N_{imag} = 0

Se	-1.499916	-0.027082	0.000000
N	1.638444	0.534902	-0.000000
C	1.111932	0.184759	1.230756
C	-0.224087	-0.194334	1.409380
C	0.146818	-0.577577	-3.763583
C	1.474859	-0.170674	-3.597185
C	1.954855	0.200870	-2.355484
C	1.111932	0.184759	-1.230756
C	-0.224087	-0.194334	-1.409380
C	-0.696466	-0.583613	-2.666215
C	1.954855	0.200870	2.355484
C	-0.696466	-0.583613	2.666215
H	-0.219954	-0.876018	-4.737362
H	2.145670	-0.154785	-4.448254
H	2.991238	0.496002	-2.229299
C	1.474859	-0.170674	3.597185
C	0.146818	-0.577577	3.763583
H	2.145670	-0.154785	4.448254
H	-0.219954	-0.876018	4.737362
H	2.629756	0.744855	-0.000000
H	2.991238	0.496002	2.229299
H	-1.735746	-0.876248	2.771393
H	-1.735746	-0.876248	-2.771393
O	-1.834881	1.617568	0.000000

Solvent = Pentyl Ethanoate
E = -2994.180796
G = -2994.037738
ZPC = 0.181703
TCE = 0.193515
TCH = 0.194459
TCG = 0.143058
N_{imag} = 0

Se	-1.506332	-0.025253	-0.000000
N	1.644250	0.525964	0.000000
C	1.118211	0.183583	1.233149
C	-0.216796	-0.199713	1.409061
C	0.156212	-0.572081	-3.767181
C	1.481612	-0.160811	-3.601638
C	1.960292	0.207004	-2.357969
C	1.118211	0.183583	-1.233149
C	-0.216796	-0.199713	-1.409061
C	-0.685056	-0.584352	-2.667286
C	1.960292	0.207004	2.357969
C	-0.685056	-0.584352	2.667286
H	-0.211784	-0.866644	-4.741905
H	2.151457	-0.137597	-4.453588
H	2.995653	0.507732	-2.233329
C	1.481612	-0.160811	3.601638
C	0.156212	-0.572081	3.767181
H	2.151457	-0.137597	4.453588
H	-0.211784	-0.866644	4.741905
H	2.625849	0.771181	0.000000
H	2.995653	0.507732	2.233329

H	-1.724396	-0.877601	2.776203
H	-1.724396	-0.877601	-2.776203
O	-1.889484	1.583792	-0.000000

PTE=O

Solvent = Water
E = -860.511530
G = -860.370849
ZPC = 0.180555
TCE = 0.192805
TCH = 0.193749
TCG = 0.140680
N_{imag} = 0

Te	-1.454403	-0.091354	0.000000
N	1.856161	0.463229	-0.000000
C	1.342608	0.156216	1.251176
C	0.004139	-0.182299	1.496378
C	0.471594	-0.467676	-3.857853
C	1.800482	-0.105191	-3.618511
C	2.232343	0.197788	-2.341532
C	1.342608	0.156216	-1.251176
C	0.004139	-0.182299	-1.496378
C	-0.413822	-0.502855	-2.794117
C	2.232343	0.197788	2.341532
C	-0.413822	-0.502855	2.794117
H	0.137436	-0.711943	-4.858276
H	2.508760	-0.068615	-4.438135
H	3.269727	0.461556	-2.161785
C	1.800482	-0.105191	3.618511
C	0.471594	-0.467676	3.857853
H	2.508760	-0.068615	4.438135
H	0.137436	-0.711943	4.858276
H	2.847504	0.670135	-0.000000
H	3.269727	0.461556	2.161785
H	-1.451889	-0.770245	2.964300
H	-1.451889	-0.770245	-2.964300
O	-1.798486	1.733048	0.000000

Solvent = Pentyl Ethanoate
E = -860.497250
G = -860.357001
ZPC = 0.180498
TCE = 0.192799
TCH = 0.193743
TCG = 0.140249
N_{imag} = 0

Te	-1.460347	-0.082594	-0.000000
N	1.853801	0.474513	0.000000
C	1.346848	0.162128	1.252834
C	0.013084	-0.195788	1.495115
C	0.488794	-0.484218	-3.856533
C	1.810492	-0.099960	-3.619277
C	2.236275	0.213271	-2.342436
C	1.346848	0.162128	-1.252834
C	0.013084	-0.195788	-1.495115
C	-0.396020	-0.526460	-2.791625
C	2.236275	0.213271	2.342436
C	-0.396020	-0.526460	2.791625
H	0.157732	-0.735966	-4.856345
H	2.518169	-0.053456	-4.439162
H	3.269589	0.495043	-2.164774
C	1.810492	-0.099960	3.619277
C	0.488794	-0.484218	3.856533
H	2.518169	-0.053456	4.439162
H	0.157732	-0.735966	4.856345
H	2.831992	0.731548	0.000000
H	3.269589	0.495043	2.164774
H	-1.430831	-0.805735	2.965861
H	-1.430831	-0.805735	-2.965861
O	-1.861690	1.701784	-0.000000

TS[PS-OOH•]

Solvent = Water
E = -1066.436861
G = -1066.284982
ZPC = 0.193919
TCE = 0.207627
TCH = 0.208571
TCG = 0.151879
N_{imag} = 1, -1038.57 cm^{-1}

S	0.049702	-1.395264	-0.649486
N	0.087709	1.391325	0.604243
C	-1.131907	0.966101	0.114154
C	-1.280505	-0.251676	-0.562485
C	3.817034	-0.061813	-0.646038
C	3.707449	1.160200	0.026744
C	2.476947	1.636156	0.440558
C	1.311692	0.893347	0.202155
C	1.432301	-0.338245	-0.456168
C	2.678440	-0.808970	-0.884607
C	-2.266568	1.773650	0.280988
C	-2.517406	-0.647628	-1.077695
H	4.782425	-0.424604	-0.974321
H	4.594798	1.750563	0.221628
H	2.390073	2.590314	0.948742
C	-3.491842	1.372146	-0.218682
C	-3.626075	0.162669	-0.907886
H	-4.354809	2.011843	-0.077341
H	-4.586847	-0.143412	-1.301068
H	0.100156	2.314974	1.021498
H	-2.159488	2.718375	0.802926
H	-2.600401	-1.595631	-1.597649
H	2.740819	-1.764103	-1.394427
O	-0.724222	-1.475509	2.478838
O	-0.193733	-2.116780	1.076148
H	-1.629636	-1.270674	2.205971

Solvent = Pentyl Ethanoate
E = -1066.430575
G = -1066.278384
ZPC = 0.193858
TCE = 0.207553
TCH = 0.208497
TCG = 0.152191
N_{imag} = 1, -1055.51 cm^{-1}

S	0.043526	-1.396104	-0.639241
N	0.082864	1.409063	0.588985
C	-1.139864	0.974886	0.118959
C	-1.288904	-0.248530	-0.547593
C	3.813077	-0.051026	-0.645247
C	3.705844	1.168949	0.027318
C	2.473280	1.645331	0.437491

C	1.307961	0.904621	0.198423
C	1.425251	-0.327596	-0.459031
C	2.671278	-0.795677	-0.884236
C	-2.275738	1.779439	0.289694
C	-2.528403	-0.644841	-1.052412
H	4.778159	-0.417776	-0.970742
H	4.593761	1.757328	0.226636
H	2.389558	2.599216	0.947233
C	-3.505697	1.373473	-0.196735
C	-3.640635	0.162095	-0.879031
H	-4.369320	2.011401	-0.049868
H	-4.603032	-0.149584	-1.264358
H	0.093770	2.314548	1.039601
H	-2.169096	2.727234	0.806336
H	-2.612793	-1.596065	-1.566680
H	2.734015	-1.753128	-1.390020
O	-0.658996	-1.500904	2.435984
O	-0.167332	-2.144630	1.018618
H	-1.605567	-1.501406	2.244408

TS[PS-OOCH$_3^•$]

Solvent = Water
E = -1105.726270
G = -1105.546734
ZPC = 0.222868
TCE = 0.237779
TCH = 0.238723
TCG = 0.179536
N_{imag} = 1, -1004.70 cm^{-1}

S	-0.170670	0.948314	-1.192255
N	-0.291394	-1.219974	0.950187
C	0.904127	-1.113241	0.264779
C	1.068665	-0.233954	-0.812808
C	-4.009048	0.064602	-0.502742
C	-3.924419	-0.860378	0.543436
C	-2.697447	-1.283007	1.021493
C	-1.510086	-0.779760	0.471107
C	-1.603475	0.158998	-0.565656
C	-2.847215	0.573415	-1.053936
C	1.996795	-1.904892	0.645693
C	2.278133	-0.154591	-1.506163
H	-4.972599	0.386128	-0.876376
H	-4.829362	-1.261790	0.983670
H	-2.632348	-2.010879	1.822918
C	3.200003	-1.809312	-0.030363
C	3.348189	-0.941242	-1.116709
H	4.032821	-2.426430	0.285224
H	4.290053	-0.877579	-1.646080
H	-0.328292	-1.934234	1.668093
H	1.878513	-2.589618	1.478194
H	2.373399	0.539145	-2.334218
H	-2.889403	1.299854	-1.858092
O	0.871076	2.092234	1.686101
O	0.263733	2.178921	0.170735
C	2.269188	1.982877	1.547010
H	2.685991	2.064064	2.556987
H	2.569538	1.017680	1.126093
H	2.673241	2.794102	0.932817

Solvent = Pentyl Ethanoate
E = -1105.721763
G = -1105.542883
ZPC = 0.222160
TCE = 0.237102
TCH = 0.238046
TCG = 0.178879
N_{imag} = 1, -1052.73 cm^{-1}

S	-0.165606	0.994492	-1.160269
N	-0.280440	-1.206288	0.941032
C	0.916139	-1.088950	0.262274
C	1.072734	-0.206245	-0.814251
C	-4.004803	0.040887	-0.523638
C	-3.917767	-0.879701	0.523619
C	-2.687320	-1.287418	1.008474
C	-1.502648	-0.770734	0.466879
C	-1.598391	0.169266	-0.568530
C	-2.842845	0.563636	-1.065122
C	2.012599	-1.878792	0.634908
C	2.271580	-0.139280	-1.523463
H	-4.969117	0.352441	-0.904348
H	-4.820753	-1.289886	0.960298
H	-2.622150	-2.013556	1.811680
C	3.209253	-1.790600	-0.055673
C	3.344763	-0.931175	-1.148343
H	4.044624	-2.407640	0.254136
H	4.279405	-0.873541	-1.691501
H	-0.305698	-1.884738	1.690694
H	1.905753	-2.561143	1.471465
H	2.360290	0.554991	-2.352010
H	-2.887795	1.292346	-1.867228
O	0.844843	1.926390	1.710261
O	0.248190	2.147635	0.202591
C	2.232794	1.990460	1.571365
H	2.651314	1.950122	2.585355
H	2.641802	1.144747	1.003896
H	2.554303	2.927683	1.100839

TS[PSE-OOH$^•$]

Solvent = Water
E = -3069.884451
G = -3069.734474
ZPC = 0.192609
TCE = 0.206774
TCH = 0.207718
TCG = 0.149977
N_{imag} = 1, -991.26 cm^{-1}

Se	0.029523	-1.404110	-0.462355
N	0.043680	1.674782	0.353478
C	-1.192837	1.160866	0.008522
C	-1.387504	-0.152498	-0.438872
C	3.854315	0.148033	-0.529493
C	3.679281	1.463684	-0.089904
C	2.418296	1.952297	0.197214
C	1.283312	1.135050	0.062502
C	1.469850	-0.186327	-0.363769
C	2.746864	-0.670461	-0.664106
C	-2.316940	1.998802	0.105274
C	-2.659012	-0.608386	-0.797698
H	4.842238	-0.230783	-0.758065
H	4.536873	2.116436	0.022069
H	2.285035	2.977470	0.526173
C	-3.573812	1.537861	-0.239814
C	-3.755522	0.230704	-0.700772

H	-4.422662	2.206192	-0.156525
H	-4.740045	-0.126062	-0.974957
H	0.048187	2.652218	0.619572
H	-2.178009	3.017984	0.450368
H	-2.775787	-1.629923	-1.142461
H	2.857759	-1.697242	-0.994910
O	-0.370879	-0.817858	2.755411
O	-0.118095	-1.787062	1.474773
H	-1.249095	-0.488400	2.518496

Solvent = Pentyl Ethanoate
E = -3069.874578
G = -3069.725232
ZPC = 0.192487
TCE = 0.206726
TCH = 0.207670
TCG = 0.149347
N_{imag} = 1, -1003.52 cm^{-1}

Se	0.038855	-1.393112	-0.460009
N	0.066607	1.683433	0.366425
C	-1.171796	1.181773	0.015769
C	-1.371651	-0.126120	-0.444694
C	3.875743	0.140505	-0.488759
C	3.707572	1.447914	-0.027140
C	2.446107	1.940933	0.253132
C	1.306677	1.136435	0.092540
C	1.486294	-0.180030	-0.350779
C	2.761906	-0.666292	-0.646779
C	-2.291661	2.024479	0.114673
C	-2.642805	-0.567952	-0.816300
H	4.862935	-0.243915	-0.711888
H	4.569354	2.090686	0.109253
H	2.319424	2.960675	0.601643
C	-3.550540	1.575730	-0.241533
C	-3.736877	0.276174	-0.717658
H	-4.395865	2.248340	-0.154314
H	-4.721758	-0.072308	-1.001755
H	0.074523	2.641800	0.689211
H	-2.150374	3.038727	0.473915
H	-2.765654	-1.585703	-1.171698
H	2.870844	-1.689883	-0.989310
O	-0.569890	-0.907216	2.653594
O	-0.161402	-1.850899	1.385677
H	-1.514212	-0.843017	2.461275

TS[PSE-OOCH$_3$•]
Solvent = Water
E = -3109.173029
G = -3108.996502
ZPC = 0.221426
TCE = 0.236944
TCH = 0.237888
TCG = 0.176527
N_{imag} = 1, -939.30 cm^{-1}

Se	0.010034	-1.111069	-1.007169
N	-0.030548	1.850920	0.255442
C	-1.264125	1.278663	0.019194
C	-1.437735	0.012027	-0.553174
C	3.825706	0.331324	-0.408084
C	3.623674	1.596348	0.151785
C	2.346379	2.079138	0.363930
C	1.219087	1.310918	0.023814
C	1.431039	0.044730	-0.536051
C	2.726487	-0.436615	-0.749810
C	-2.413004	2.010165	0.366669
C	-2.719235	-0.506066	-0.769102
H	4.826621	-0.046062	-0.573660
H	4.473285	2.211117	0.424391
H	2.191398	3.060975	0.798531
C	-3.675657	1.491730	0.149791
C	-3.840367	0.226564	-0.422582
H	-4.543187	2.077990	0.428662
H	-4.829721	-0.178531	-0.592227
H	-0.043140	2.772047	0.676391
H	-2.286692	2.991317	0.811797
H	-2.821645	-1.492292	-1.209128
H	2.858076	-1.421805	-1.184319
O	0.485184	-2.019867	2.196890
O	0.089430	-2.224062	0.631441
C	-0.318666	-0.962986	2.665365
H	-0.169879	-0.911664	3.749189
H	-0.020670	-0.003877	2.227870
H	-1.380157	-1.143512	2.461053

Solvent = Pentyl Ethanoate
E = -3109.164961
G = -3108.988199
ZPC = 0.220355
TCE = 0.235137
TCH = 0.236081
TCG = 0.176761
N_{imag} = 1, -976.33 cm^{-1}

Se	-0.037676	-1.138380	-0.954484
N	-0.115559	1.638888	0.627086
C	-1.348281	1.157599	0.221119
C	-1.503168	-0.017313	-0.526496
C	3.696194	0.630483	-0.815534
C	3.490844	1.776866	-0.046414
C	2.228223	2.097046	0.419294
C	1.128281	1.271740	0.138451
C	1.345495	0.116675	-0.620813
C	2.618760	-0.192835	-1.099755
C	-2.500799	1.874826	0.579779
C	-2.775365	-0.446877	-0.916950
H	4.683983	0.380985	-1.181791
H	4.323085	2.430187	0.188245
H	2.070682	2.994167	1.008753
C	-3.755689	1.436881	0.197015
C	-3.902695	0.273199	-0.561809
H	-4.628526	2.010006	0.487054
H	-4.884449	-0.067368	-0.865801
H	-0.139976	2.513347	1.135273
H	-2.390088	2.785192	1.159798
H	-2.868550	-1.359769	-1.495910
H	2.758633	-1.094281	-1.686736
O	1.040997	-1.907651	2.035183
O	0.270266	-2.174823	0.624053
C	0.116633	-1.235432	2.834754
H	0.564996	-1.148131	3.832861
H	-0.093148	-0.222714	2.466595
H	-0.827460	-1.786279	2.924757

TS[PTE-OOH•]

Solvent = Water
E = -936.214670
G = -936.066611
ZPC = 0.191786
TCE = 0.206296
TCH = 0.207240
TCG = 0.148059
N_{imag} = 1, -984.62 cm^{-1}

Te	0.061624	-1.425943	-0.332527
N	0.087549	1.845776	0.312993
C	-1.159504	1.361537	-0.056142
C	-1.418541	0.037316	-0.438071
C	3.974664	0.405081	-0.391750
C	3.735164	1.734244	-0.032439
C	2.447145	2.184168	0.189702
C	1.347020	1.315230	0.070938
C	1.594200	-0.022492	-0.268641
C	2.901759	-0.462308	-0.506870
C	-2.236013	2.267599	-0.029202
C	-2.710034	-0.352347	-0.808990
H	4.983019	0.053994	-0.570904
H	4.561774	2.427923	0.067364
H	2.267252	3.221084	0.454901
C	-3.510678	1.866680	-0.384025
C	-3.759583	0.551864	-0.785045
H	-4.318893	2.588123	-0.354812
H	-4.756722	0.239760	-1.068788
H	0.094046	2.831472	0.545044
H	-2.046996	3.294056	0.268658
H	-2.887441	-1.380142	-1.108217
H	3.070230	-1.500457	-0.774020
O	-0.983093	-0.446645	2.773409
O	-0.298434	-1.509836	1.730620
H	-1.764961	-0.214787	2.252178

Solvent = Pentyl Ethanoate
E = -936.201585
G = -936.054278
ZPC = 0.191504
TCE = 0.206185
TCH = 0.207129
TCG = 0.147307
N_{imag} = 1, -1000.39 cm^{-1}

Te	0.053749	-1.408887	-0.358739
N	0.092013	1.881183	0.263512
C	-1.163977	1.388908	-0.051222
C	-1.430354	0.062840	-0.420802
C	3.980247	0.402668	-0.340005
C	3.743984	1.734074	0.009215
C	2.454862	2.192972	0.202227
C	1.349354	1.333567	0.063507
C	1.592550	-0.007950	-0.264871
C	2.902042	-0.455529	-0.472479
C	-2.242662	2.291057	0.007980
C	-2.732370	-0.328394	-0.747599
H	4.989247	0.041870	-0.495392
H	4.573472	2.421956	0.125511
H	2.279176	3.232184	0.462020
C	-3.527798	1.886636	-0.301735
C	-3.785456	0.571058	-0.691076
H	-4.336880	2.605750	-0.245294
H	-4.790457	0.254235	-0.940287
H	0.100913	2.854054	0.538853
H	-2.049252	3.318547	0.300128
H	-2.920837	-1.357526	-1.037823
H	3.071967	-1.496684	-0.728553
O	-0.893237	-0.562838	2.705439
O	-0.283950	-1.650961	1.612251
H	-1.781407	-0.501575	2.330313

TS[PTE-OOCH$_3^•$]
Solvent = Water
E = -975.503485
G = -975.327714
ZPC = 0.220891
TCE = 0.236558
TCH = 0.237502
TCG = 0.175771
N_{imag} = 1, -965.87 cm^{-1}

Te	-0.002697	-1.235900	-0.866241
N	-0.080615	2.072943	-0.319063
C	-1.317924	1.451510	-0.196015
C	-1.542904	0.081534	-0.385204
C	3.837358	0.564991	-0.033525
C	3.557792	1.924337	0.123271
C	2.260018	2.393142	0.030610
C	1.191674	1.517576	-0.234313
C	1.479924	0.158073	-0.408373
C	2.793989	-0.307931	-0.297808
C	-2.419686	2.266627	0.120204
C	-2.828959	-0.450892	-0.240394
H	4.852218	0.196179	0.045435
H	4.359475	2.624524	0.326926
H	2.047200	3.448243	0.168949
C	-3.688538	1.731732	0.247842
C	-3.905219	0.363064	0.072899
H	-4.516352	2.386585	0.493427
H	-4.896952	-0.058342	0.178119
H	-0.103217	3.067841	-0.131130
H	-2.255144	3.328490	0.271172
H	-2.978886	-1.516829	-0.379769
H	2.992818	-1.367242	-0.427368
O	0.654743	-1.573107	2.513743
O	0.172643	-2.093268	1.030531
C	-0.145848	-0.453581	2.808602
H	0.087780	-0.165418	3.839494
H	0.080166	0.393122	2.150402
H	-1.213711	-0.691034	2.741340

Solvent = Pentyl Ethanoate
E = -975.493707
G = -975.318571
ZPC = 0.220255
TCE = 0.235867
TCH = 0.236811
TCG = 0.175135
N_{imag} = 1, -973.17 cm^{-1}

Te	-0.013188	-1.053970	-1.050956
N	-0.352101	2.137591	-0.504694
C	-1.494874	1.428019	-0.109886
C	-1.587625	0.033326	-0.199026
C	3.598969	0.941767	0.332784

C	3.221359	2.281947	0.406266
C	1.916282	2.664715	0.135979
C	0.961332	1.712706	-0.244156
C	1.355997	0.375600	-0.352449
C	2.659271	-0.010138	-0.041163
C	-2.600394	2.141197	0.372964
C	-2.736456	-0.630214	0.232997
H	4.613623	0.642633	0.565554
H	3.945000	3.034996	0.696554
H	1.617464	3.703682	0.231147
C	-3.751698	1.477581	0.767671
C	-3.824070	0.086208	0.713850
H	-4.594082	2.051555	1.136180
H	-4.718059	-0.432322	1.037594
H	-0.463091	3.139357	-0.406596
H	-2.539294	3.222464	0.443513
H	-2.779178	-1.713499	0.181820
H	2.926452	-1.059835	-0.083259
O	1.350330	-2.154437	1.819155
O	0.490723	-2.335239	0.407059
C	0.525210	-1.385588	2.637548
H	1.016744	-1.329852	3.617641
H	0.400817	-0.360656	2.261253
H	-0.464179	-1.840583	2.771205
